# Metamaterial-enabled battery-free wireless sensor networks for unencumbered human-machine interactions

**DOI:** 10.1038/s41467-026-77600-2

**Published:** 2026-09-14

**Authors:** Yimeng Zhang, Fengman He, Tianyiyi He, Qianrui Luo, Juerui Lin, Qinghao Xu, Changqing Guo, Xinyue Chai, Yuchen Wang, Tong Wu, Yiru Jiang, Chun Jin, Xiping Hu, Wenbo Ding, Xi Tian

**Affiliations:** 1 Institute of Data and Information, Tsinghua Shenzhen International Graduate School, Tsinghua University, Shenzhen, China; 2 Artificial Intelligence Research Institute, Shenzhen MSU-BIT University, Shenzhen, China; 3 Center of Double Helix, Tsinghua Shenzhen International Graduate School, Tsinghua University, Shenzhen, China; 4 Shenzhen X-Institute, Shenzhen, China; 5 School of Medical Technology, Beijing Institute of Technology, Beijing, China

**Keywords:** Electrical and electronic engineering, Electronics, photonics and device physics

## Abstract

Wireless body sensor networks could enable unencumbered human-machine interfaces for robotics, assistive healthcare, and immersive computing. However, their widespread adoption remains constrained by bulky batteries and inefficient wireless communication. Here, we report a battery-free wireless body sensor network enabled by a dual-mode metamaterial textile for continuous and unobtrusive human-machine interactions. The textile, digitally embroidered with liquid-metal fibers, establishes ultra-wideband electromagnetic pathways to simultaneously support efficient near-field power transfer and Bluetooth communication across the entire human body. This architecture enhances data throughput by 37.8 times and doubles wireless power transfer efficiency compared with emerging near-field clothing. When worn, the metamaterial-integrated clothing allows an NFC-enabled smartphone to serve as a central hub, wirelessly delivering power to and acquiring multimodal signals from distributed battery-free HMI sensors. We demonstrate that this battery-free body sensor network remains robust during motion and enables intuitive, unencumbered interactions in robotic control, virtual reality, and human digital twins.

## Abstract (web summary)

A battery-free wireless body sensor network enabled by metamaterial textiles is reported. Using a smartphone as a hub, it wirelessly powers distributed sensors for unencumbered human-machine interactions in robotics, VR, and digital twins.

## Introduction

Human–machine interfaces (HMIs) that enable seamless communication between humans and machines have been transforming fields such as robotics, healthcare, and immersive virtual and augmented environments, fundamentally redefining how people interact with technology in daily life^1–6^. An important future of HMIs lies in achieving unencumbered, seamless, and continuous interaction across diverse scenarios, with the integration of wearable sensor networks that deliver dense, multimodal data streams playing a pivotal role toward this vision^7–10^. The practical deployment of such sensor networks, however, poses significant challenges, as distributed sensors must be interconnected across the body in a lightweight, unobtrusive, and reliable manner. Existing approaches often rely on wired architectures^11,12^, which could ensure stable power and data transmission but severely restrict natural movement. Portable wireless modules^13–15^ have been shown to alleviate the constraints of tethered connections, yet their reliance on rigid, bulky batteries adds weight, compromises comfort, and necessitates frequent replacement. Consequently, achieving seamless power delivery and data communication in fully unobtrusive wearable sensor networks remains a central challenge toward realizing unencumbered HMIs.

To overcome these limitations, wireless technologies that enable battery-free wireless body sensor networks (WBSNs) are highly desired. Although both near-field^16^ and far-field^17,18^ wireless power transfer techniques have been explored for WBSNs, they are often hindered by significant distance-dependent power loss and require restrictive proximity to a transmitter coil, again limiting user mobility. Recent works have shown the potential to mitigate this challenge by leveraging the ubiquitous near-field communication (NFC)-enabled smartphone to serve as a wireless power source^19,20^. This strategy has inspired the development of specialized metamaterial^21–23^ and NFC^19,20,24,25^ textiles that can power and interconnect battery-free wearable sensors across the body via NFC or bluetooth low energy (BLE), enabling continuous vital sign and activity monitoring with unrestricted movement. However, these systems operate in a single functional mode, inevitably favoring either high-speed communication or efficient energy transfer. For instance, early metamaterial textiles^21^ working in the BLE band demonstrated over 30 dB enhancement in wireless communication, yet their capacity for long-range or high-efficiency power transfer remained limited. Conversely, NFC-based textiles^19,20,24^ provide stable energy delivery but are intrinsically restricted by narrow bandwidth and low data rates, making them unsuitable for HMI applications which require rapid, multimodal information exchange. These limitations highlight the urgent need for an integrated textile platform that can simultaneously support high-throughput data transmission and efficient wireless power delivery across the human body, paving the way for untethered and seamless HMIs.

**Fig. 1:**
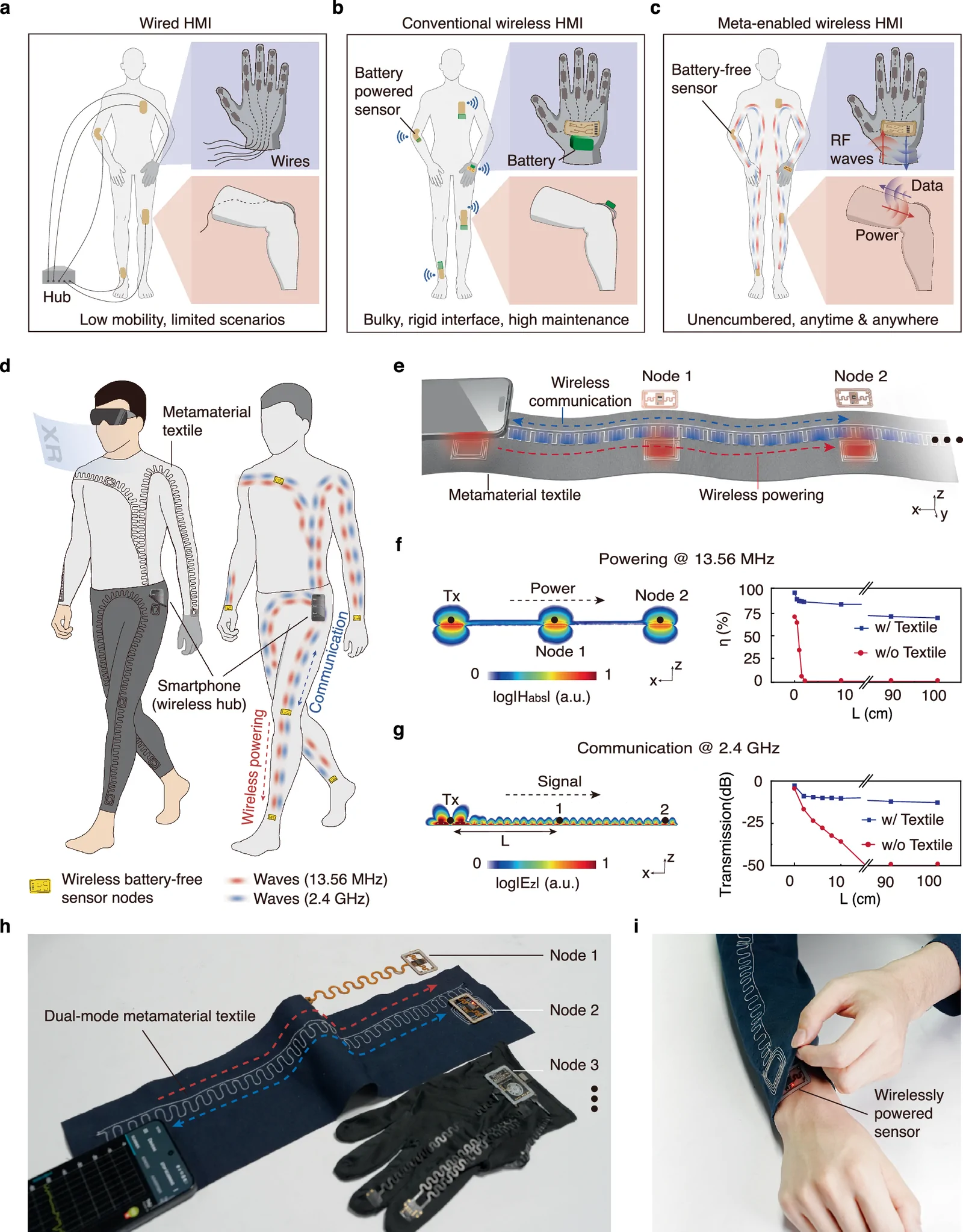
Metamaterial-enabled battery-free WBSN for unencumbered human-machine interactions. **a** Schematic of a conventional wired human-machine interface (HMI) system, in which extensive wiring restricts user mobility. **b** Schematic of conventional wireless HMI system designs that rely on distributed battery-powered sensor nodes, adding bulk and limiting wearability. **c** Schematic of the proposed metamaterial-enabled wireless HMI system, which eliminates tethered connections or bulky batteries at individual sensor nodes. **d** Design concept of the battery-free WBSN, where an NFC-enabled smartphone acts as the central hub to construct body-scale wireless powering and communication networks through dual-mode metamaterial textiles. **e** The operating principle of the dual-mode metamaterial textile, which simultaneously supports the propagation of near-field (13.56 MHz) and Bluetooth (2.4 GHz) electromagnetic waves for power delivery and data communication, respectively. **f** Simulated electric field distribution at 13.56 MHz along the metamaterial textile, and the corresponding power transfer efficiency as a function of distance between the transmitter and receiver, with and without the metamaterial structure. **g** Simulated electric field distribution at 2.4 GHz along the metamaterial textile, and the corresponding wireless signal transmission as a function of distance between the transmitter and receiver, with and without the metamaterial structure. **h** Photograph showing the metamaterial textile enabling a smartphone to wirelessly power a wearable sensor node while simultaneously receiving sensory data. Sensor nodes in various forms are positioned near the receiver, demonstrating the scalability and configurational flexibility of the system. **i** Photograph of an epidermal sensor node attached to the wrist and wirelessly powered by the metamaterial textile integrated into the sleeve, confirming operational reliability both above and below the metamaterial textile.

In this article, we report a battery-free wireless sensor network built upon dual-mode metamaterial textiles for untethered, intuitive, and continuous human-machine interactions. This sensor network is realized by creating dual-mode metamaterial textiles—patterned conductive fabrics with electromagnetic (EM) properties—that form ultra-wideband EM pathways capable of guiding the propagation of both powering and communication radio waves (Supplementary Fig. 1 and Supplementary Table 1). With this approach, we simultaneously achieve high-efficiency wireless power delivery (13.56 MHz) and high-throughput data transmission (2.4 GHz) through metamaterial pathways across the full body. Unlike wired HMI systems^11,12^ that restrict user mobility (Fig. 1a) or conventional wireless systems^13^ reliant on bulky batteries (Fig. 1b), our design allows a smartphone to serve as a central hub, wirelessly supplying power to and acquiring multimodal signals from distributed battery-free sensors without compromising natural movement or wearing comfort (Fig. 1c). This metamaterial-enabled HMI system provides simultaneous advantages in power delivery efficiency, network scalability, communication robustness, data privacy, and wearable comfort (Supplementary Note A). We fabricate the metamaterial textiles using computer-aided digital embroidery of liquid metal fibers and show that they enhance wireless data throughput by over one order (37.8 times) while simultaneously doubling power-transfer efficiency compared with emerging near-field clothing^19,20,24^. The resulting HMI system supports battery-free, miniaturized, and heterogeneous sensors distributed across the body, operating cooperatively with minimal interference to natural motion. This enables seamless acquisition of full-body signals across diverse environments. To validate the system, we demonstrate a series of intuitive and unobtrusive human-machine interactions, including intelligent hand gesture recognition for human–robot collaboration and immersive control in virtual and augmented reality. Furthermore, by wirelessly bridging the metamaterial textiles on the shirt and trousers, we realize a full-body WBSN capable of generating dynamic digital-twin representations of human motion, paving the way toward wearable, lightweight, battery-free, and unencumbered HMI systems that integrate seamlessly with the human body.

## Results

### Metamaterial-enabled battery-free wireless body sensor networks

Figure 1d presents the concept of the proposed battery-free WBSN, which enables untethered, intuitive, and versatile human-machine interactions by simultaneously supporting high-efficiency wireless power delivery and high-throughput data transmission across the full body. This capability is achieved using dual-mode metamaterial textiles that form such ultra-wideband EM pathways between a central hub (e.g., smartphone) and spatially distributed battery-free sensor nodes. To ensure compatibility with consumer electronics without requiring hardware modification, we employ the NFC band (13.56 MHz) for wireless power transfer and the BLE band (2.4 GHz) for data communication. To simultaneously guide radio waves spanning two widely separated frequency regimes, the dual-mode metamaterial textile incorporates spoof surface plasmon (SSP) waveguides^26,27^ alongside a network of interconnected planar inductor patterns (Supplementary Fig. 2). This architecture enables a unified, high-efficiency wireless power-and-data network across the entire body.

The operating principle is illustrated in Fig. 1e and Supplementary Fig. 3. In the powering mode, the SSP structure functions as a conductive pathway interlinking the planar inductors to form the necessary current loops. This effectively extends the smartphone’s wireless powering range to distant sensor nodes. Specifically, the 13.56 MHz quasi-static magnetic field generated by the NFC coil (Supplementary Fig. 4) does not excite propagating waves along the SSP structure due to the extremely long wavelength at this frequency. Consequently, the near-field energy transfer remains confined to the coil region without coupling into the SSP waveguide. In the communication mode, the SSP waveguide—composed of subwavelength comb-shaped units—confines and guides 2.4 GHz surface plasmon-like waves, enabling high-speed data transfer between distributed sensor nodes. The surface wave supported by the SSP waveguide exhibits a pronounced evanescent field extending beyond the conductor, thereby enabling practical near-field coupling with surrounding RF antennas (Supplementary Note B and Supplementary Fig. 5). In contrast to conventional wireless protocols that radiate energy omnidirectionally into free space, leading to significant energy loss and potential interception, the SSP structure localizes electromagnetic propagation within close proximity to the body, thereby improving both transmission efficiency and BLE communication security^21^. Because time–harmonic fields at different frequencies are orthogonal in a linear system, the proposed SSP structure can simultaneously support these two widely separated frequency regimes without mutual interference. This dual-mode operation enables sensor nodes at different body locations to be wirelessly powered by a smartphone via NFC while transmitting data back through BLE.

The superior performance of the dual-mode metamaterial textile is illustrated through electromagnetic simulations presented in Fig. 1f, g. The results confirm that the SSP waveguide enables highly efficient propagation of 2.4 GHz surface waves, while the interconnected inductor network facilitates 13.56 MHz power relaying across spatially separated regions. This dual-mode structure allows both power and data signals to be guided seamlessly to multiple sensor nodes distributed along the textile pathways. In contrast, in the absence of the metamaterial architecture, both power transfer efficiency and communication signal strength decrease sharply with increasing transmitter-receiver distance. These results highlight the critical role of the dual-mode metamaterial design in establishing a robust, battery-free WBSN capable of simultaneously supporting wireless power transfer and data communication.

The metamaterial network clothing is fabricated using computer-aided digital embroidery technology, which enables the precise integration of conductive fibers into customizable patterns on textile substrates (Supplementary Fig. 6). These fibers are formed by injecting the liquid metal Galinstan (a gallium-indium-tin alloy) into perfluoroalkoxy alkane (PFA) tubing (Supplementary Fig. 7). The PFA tubing provides excellent chemical and thermal resistance, while Galinstan offers high electrical conductivity at room temperature. Owing to these combined advantages, the resulting liquid metal fibers exhibit stable electrical performance, conformability to the curved surfaces of the human body, and durability against mechanical deformation, while also demonstrating preliminary washability (Supplementary Figs. 8–10, Supplementary Table 2). As shown in Fig. 1h, the dual-mode metamaterial pattern is practically integrated into the fabric, enabling diversified sensor nodes to be interconnected and wirelessly powered through the textile network, with data transmission to a central hub (e.g., a smartphone). Remarkably, the embedded metamaterial textile not only connects devices located on the garment surface but also wirelessly powers epidermal sensor nodes attached beneath it on the skin (Fig. 1i). Consequently, the proposed metamaterial network clothing establishes a unified communication and power delivery framework for heterogeneous sensors distributed across both textile and skin interfaces, forming a comprehensive, battery-free WBSN capable of monitoring diverse motion and physiological signals.

### Metamaterial design and characterization

**Fig. 2:**
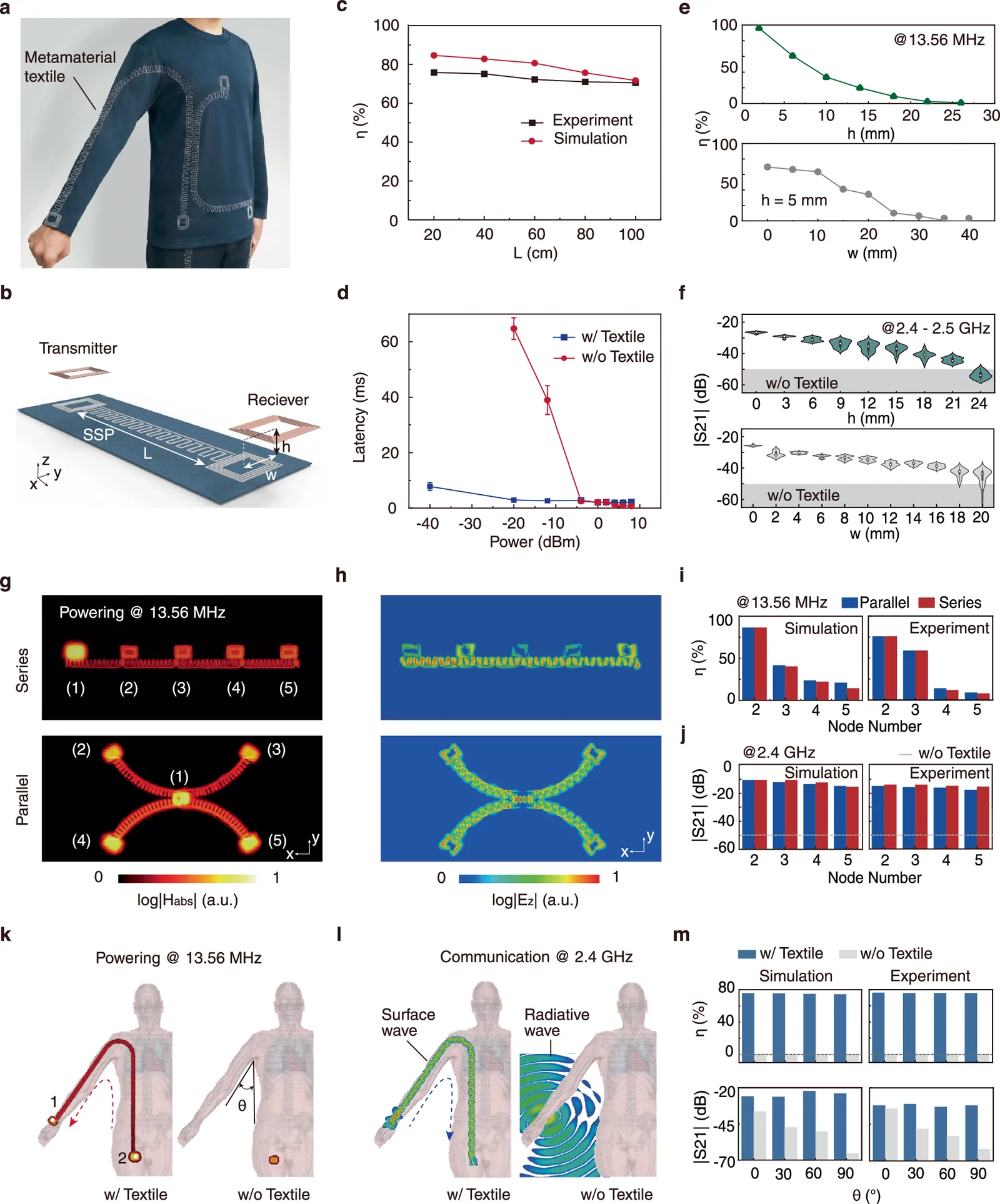
Battery-free metamaterial networks design and characterization. **a** Photograph of battery-free metamaterial networks integrated into a long-sleeved shirt and trousers. **b** Schematic of the metamaterial textile interconnecting a transmitter and receiver node, where *L* is the relay length, *w* is the lateral offset, and *h* is the vertical separation. **c** Simulated and measured power transfer efficiency as a function of relay length *L* (*h* = 0.5 cm and *w* = 0 cm). **d** Experimentally measured wireless transmission (BLE) latency as a function of transmit power, with and without the metamaterial textile. Error bars show mean ± s.d. (*n* = 5 measurements). **e** Experimentally measured wireless power transfer efficiency at 13.56 MHz under varying *w* and *h* configurations of the receiver coil in (**b**). **f** Violin plots for comparison of transmission coefficient (∣*S*_21_∣) measured under different *h* and *w* configurations of the receiver antenna with and without the metamaterial textile, across the 2.4–2.5 GHz ISM band. The box plots inside the violins indicate the quartiles of corresponding transmission spectra. The end-points show the minimum and maximum values. The white dots represent median values. The whiskers denote 1.5 times the interquartile range. The gray shaded region represents the baseline (∣*S*_21_∣) range for the condition without textile. **g** Normalized magnetic fields (∣*H*_abs_∣) at 13.56 MHz, showing the wireless interconnection between the source (node 1) and receivers through the metamaterial network. **h** Simulated electric field distribution at 2.45 GHz, showing BLE communication among interconnected nodes. Simulated and experimental results of power transfer efficiency (**i**) and transmission coefficient (**j**) as functions of node number under parallel and series configurations. Comparisons between full-wave simulation and experimental results of wireless powering (**k**) and data transmission (**l**) in a computational human body model. **m** Simulated and on-body measured power transfer efficiency and transmission coefficient as functions of arm-raising angle, with and without the metamaterial textile, demonstrating angular stability and robustness to body movement. The data are plotted based on single measurements.

We designed and fabricated metamaterial-enabled clothing capable of guiding wireless power and communication signals across the entire body, including the torso and limbs (Fig. 2a). The functionality of this system was validated using an NFC-enabled Android smartphone as a wireless power source and communication hub, together with battery-free BLE sensor nodes integrated with light-emitting diodes (LEDs) to indicate their operational status. When placed on the metamaterial textiles, the sensor nodes exhibited robust LED activation (Supplementary Movie 1), confirming the establishment of a dual-mode wireless powering and communication network. Measurements of the rectified voltage and BLE received signal strength indicator (RSSI) on the sensor side further verified the efficient coupling of power and data through the metamaterial textile (Supplementary Figs. 11 and 12). To evaluate the system’s spatial operating margin, we characterized the RSSI stability as a function of the distance between the smartphone and the metamaterial textile (Supplementary Fig. 13). The results demonstrate that a reliable communication link is maintained even as the device is moved away from the textile, highlighting the system’s tolerance to practical, non-ideal positioning. Moreover, experiments demonstrated that the metamaterial textiles can enable reliable propagation of wireless signals and power across separate garment pieces (Supplementary Figs. 14 and 15 and Supplementary Movie 1), an essential capability for constructing body-scale WBSNs in HMI applications.

The metamaterial textile integrates an SSP structure and planar inductor patterns that respectively enable 2.4 GHz signal transmission and 13.56 MHz wireless power delivery. To achieve seamless integration with powering inductor coils on the clothing, we designed a metamaterial structure that consists of hollowed comb shapes with conductive wires. This hybrid structure simultaneously functions as a transmission line for the 13.56 MHz power transfer mode and a high-speed surface-wave waveguide in the 2.4 GHz communication mode. As shown in Supplementary Fig. 16, the metamaterial structure supports surface-wave propagation with a wavelength compressed to approximately one-third of the free-space wavelength (Supplementary Fig. 17), confirming its strong electromagnetic confinement and efficient signal guidance.

As shown in Fig. 2b and Supplementary Fig. 18, a two-port wireless sensor network was used to characterize the electromagnetic performance of the dual-mode metamaterial textile, where *h* denotes the vertical distance, and w represents the lateral displacement. The power transfer efficiency *η* was measured as a function of relay length (*L*) under fixed geometric parameters (*h* = 0.5 cm, *w* = 0 cm) (Fig. 2c). The results reveal that the metamaterial enables high power transfer efficiency, maintaining around 70% efficiency even when two nodes are separated by up to 1 m. In the communication mode, low latency is essential for real-time continuous data exchange. Figure 2d shows the experimentally measured BLE latency as a function of transmit power. The metamaterial textile enables reliable communication even at a low transmit power of −40 dBm, maintaining latency below 10 ms. In contrast, conventional radiative BLE requires transmit power exceeding −10 dBm to prevent connection failure. Further measurement of wireless data transmission speed demonstrates that our proposed metamaterial-enabled network achieves a total transmission time of merely 5.58 s at a transmit power of −40 dBm, whereas the conventional BLE system necessitates 15.98 s under the same test conditions at a higher transmit power of −20 dBm (Supplementary Fig. 19). These results confirm that the metamaterial textile maintains superior communication quality while substantially reducing transmit power (Supplementary Movie 2), which is essential for battery-free wireless systems.

To assess the robustness of the metamaterial textile in both power transfer and communication modes, we evaluated its performance under various misalignment conditions. At the operating frequency of 13.56 MHz, the power transfer efficiency (*η*) was measured across different lateral displacements (*w*) and vertical separations (*h*) of the receiver coil. As shown in Fig. 2e, the efficiency gradually decreases with increasing misalignment, dropping to zero when *w* exceeds 30 mm or *h* exceeds 26 mm. These tolerable ranges of *w* and *h* are design-adjustable by modifying the metamaterial structural parameters (Supplementary Fig. 20). For the communication mode at 2.4 GHz, Fig. 2f presents violin plots of the benchtop-measured transmission coefficient (2.4–2.5 GHz) under varying *w* and *h* (Supplementary Figs. 21 and 22). The results show that when *h* ≤ 20 mm and *w* ≤ 16 mm, the transmission coefficient remains above −50 dB, ensuring reliable data transmission. During daily motion, relative displacement between clothing and the human body is inevitable; nevertheless, the metamaterial textile maintains stable power delivery and communication performance within the tolerable range, demonstrating strong robustness to real-world positional deviations.

Simultaneous power supply and communication across multiple sensor nodes can be achieved using metamaterial networks with multiple terminals. When interconnected either in series or in parallel (Supplementary Figs. 23 and 24), excitation of a single terminal induces multiple magnetic hotspots (Fig. 2g) and launches surface waves (Fig. 2h) that propagate toward the sensor nodes. These magnetic hotspots enable multimodal battery-free sensor operation, while the surface waves facilitate high-speed data transmission along the textile network. Figure 2g illustrates the magnetic field distribution generated when a power source is placed at Node 1 in the powering mode. The results confirm that the metamaterial network effectively distributes magnetic energy along its structure, inducing strong magnetic coupling at the receiver nodes. In the 2.4 GHz communication mode, the field distribution generated by a source at Node 1 is shown in Fig. 2h. The field is tightly confined to the surface of the metamaterial network, propagating as surface waves along the textile. The simulated field confinement (Fig. 2h) indicates that the electromagnetic energy is strongly localized within the evanescent region of the SSP structure. By suppressing radiation into the far-field, this guided-wave mechanism ensures efficient power delivery across the distributed network. This physical behavior is consistent with the experimentally measured transmission performance in the multi-node configuration (Fig. 2j). Figure 2i, j shows the power transfer efficiency and transmission performance as functions of the number of interconnected nodes for both series and parallel topologies. In both configurations, *η* is inversely proportional to the number of terminals due to the distribution of energy over multiple terminal points. For a parallel relay network with five terminals, the power transfer efficiency to each node remains around 18%, sufficient to operate a BLE device under a transmitted power of 100 mW from the smartphone. Meanwhile, the transmission coefficient at 2.4 GHz remains consistently above −20 dB. This represents a more than three-order-of-magnitude enhancement in received signal strength compared to conventional radiative propagation. Such a substantial improvement provides experimental validation of the field confinement effect, demonstrating the system’s ability to bypass the high path loss typically associated with body-centric wireless links.

Furthermore, full-wave electromagnetic simulations were performed to evaluate the powering and communication performance of the metamaterial textiles on a human body model (Supplementary Figs. 25–28). Figure 2k compares the magnetic field distributions during wireless powering. Without the metamaterial textiles, 13.56 MHz magnetic fields experience severe attenuation in air and biological tissue, preventing effective energy delivery to battery-free nodes. In contrast, the metamaterial textile guides magnetic energy along its structure, extending the effective powering range to over 1 m (from abdomen to wrist) while maintaining *η* above the threshold required for reliable sensor operation. Figure 2l compares wireless communication performance between conventional radiative electromagnetic waves and the metamaterial-enabled surface waves. The results show that radiative waves are strongly scattered and absorbed by biological tissues, resulting in high signal loss and potential privacy leakage. By contrast, the metamaterial textile confines wireless signals as surface waves that propagate around the body, enabling highly localized and energy-efficient data transmission. The simulation and measurement results in Supplementary Figs. 27 and28 further demonstrate that a conductive trace without SSP structures cannot effectively support surface wave propagation and exhibits significantly weaker transmission efficiency. As shown in Fig. 2m, the stable power transfer efficiency *η* and communication transmission ∣S21∣ across varying limb angles verify the robustness of the system for real-time powering and communication during natural body movements. As the bending angle increases, the transmission coefficient without the textile (radiative propagation) decreases markedly due to increased path loss, whereas the surface-wave-guided transmission remains relatively stable. At a bending angle of 90^∘^, the difference exceeds 30 dB, corresponding to an improvement of over three orders of magnitude in transmission efficiency under this condition.

### Battery-free wireless sensor node design

**Fig. 3:**
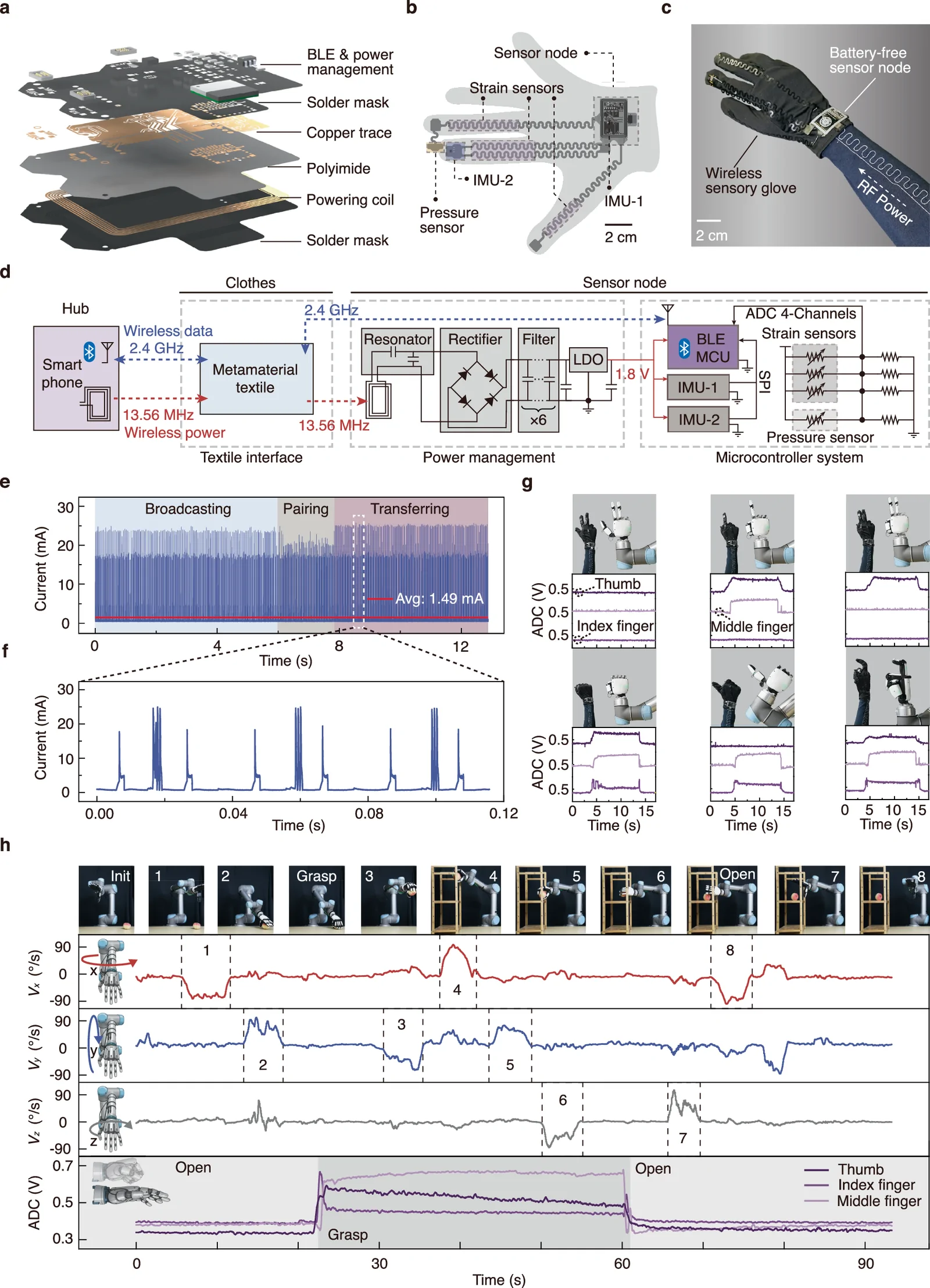
Battery-free sensor node design and human–robot collaboration application. **a** Exploded schematic illustration of a wireless sensor node, showing its core modules: the copper coil and power management elements for wireless power harvesting; the BLE module for data processing and wireless communication. **b** Components illustration of the sensory glove. This sensor node was configured with three strain sensors, two IMUs, and a pressure sensor. **c** Photograph of the wireless sensory glove integrated with a battery-free sensor node. **d** Block diagram of system operation, showing the sensor node components and system-level wireless powering and data transmission units. SPI serial peripheral interface, LDO low dropout regulator, IMU inertial measurement unit, BLE MCU bluetooth low energy microcontroller unit, ADC analog-to-digital converter. **e** The current consumption across different BLE communication phases, with an average of 1.49 mA. **f** Peak current during BLE packet transmission at 20 ms interval. **g** Robot gesture control via battery-free sensory glove. Resistance variations of the strain sensors are transduced into voltage changes, which are recorded by the ADC. In each sub-figure, the three data lines correspond to strain sensors on the index finger, middle finger, and thumb, shown from bottom to top. **h** Data recorded during human–robot collaboration, including grasping, moving, and placing. The robotic arm and hand are wirelessly controlled by a battery-free sensory glove that collects strain signals from each finger and the triaxial angular velocity from the dorsal IMU.

To realize an unencumbered wearable HMI, the sensing nodes are desired to be lightweight, compact, and capable of fully battery-free and wireless operation. Achieving this function requires the seamless integration of three essential modules—wireless energy harvesting, signal processing, and data transmission—within a single miniaturized platform. Guided by these requirements, we developed a compact battery-free wireless sensor node that integrates all components onto a flexible circuit. Figure 3a illustrates an exploded view of the battery-free sensor node, with the key elements highlighted: a powering coil, a power-management circuit, and a BLE communication module. This lightweight and flexible node supports wireless energy harvesting, multimodal data acquisition, and wireless data transmission (Supplementary Fig. 29). To harvest energy from the 13.56 MHz NFC signal emitted by a smartphone, this battery-free sensor node employs a six-turn coil. The received signal is rectified by a full-wave bridge rectifier composed of low-junction-capacitance Schottky diodes, followed by multi-stage filtering capacitors to suppress power supply fluctuations. The filtered voltage is then regulated to 1.8 V through a low-dropout regulator (LDO) to power the low-power inertial measurement unit (IMU) and the BLE microcontroller. Meanwhile, to maximize practical deployability and compatibility with existing wireless hardware, we directly utilized the antenna integrated within the BLE MCU for 2.4 GHz signal transmission. The corresponding reflection and transmission characteristics are presented in Supplementary Figs. 30–32. In this configuration, the generated source excites surface waves on the textile SSP waveguide, which then guide the transmitted signals to a smartphone receiver.

In an unencumbered HMI system, the battery-free sensor node can be configured and deployed on different body sites to achieve specific sensing functions, such as intelligent hand-gesture recognition. To demonstrate the human-machine interaction capabilities of metamaterial-enabled WBSN, we designed and fabricated a prototype of a battery-free sensory glove. As shown in Fig. 3b, c, the glove integrates a flexible sensor node on the dorsum of the hand, three strain sensors across the finger joints, one pressure sensor on the inner side of the middle finger, and two six-axis IMUs located at the index fingertip and the dorsum of the hand. All sensing elements are interconnected via flexible printed circuits with serpentine traces, ensuring stretchability and mechanical compliance during grasping and bending (Supplementary Figs. 33 and 34). The strain sensors are fabricated from stretchable multi-walled carbon nanotube (MWCNT) fibers for detecting finger bending (Supplementary Fig. 35), while the pressure sensors use PEDOT:PSS-coated fabric for touch sensing (Supplementary Fig. 36). The main control board of this battery-free sensory glove weighs only 0.836 g, while the complete battery-free sensory glove weighs only 13.832 g.

Figure 3d illustrates the block diagram of the system operation, outlining the components of the battery-free sensor node and its interface with the WBSN for power transfer and signal exchange. The wireless sensing system operates through a dual-mode metamaterial body network, which simultaneously transfers wireless power and data between an NFC-enabled Android smartphone and distributed sensor nodes. When the smartphone’s NFC coil emits a 13.56 MHz electromagnetic field, the metamaterial network captures and guides the field, enabling efficient wireless energy transfer to the flexible sensor node. The received alternating current signal is then rectified, filtered, and regulated to a stable 1.8 V output that powers the entire sensing circuit. At the same time, the metamaterial guides 2.4 GHz Bluetooth signals along the textile through the SSP pathway, establishing data transmission between the sensor nodes and the smartphone. The low-power characteristics of this proposed wireless battery-free sensor node are shown in Fig. 3e, f. Quantitative evaluation of power transfer performance under realistic smartphone operation is provided in Supplementary Fig. 37, demonstrating a substantial operating margin under practical conditions and further supporting the feasibility of using a commercial NFC-enabled Android smartphone as a universal hub. The rectified output voltage of sensor nodes was measured, demonstrating that sufficient power can be harvested from different smartphones to satisfy the input-voltage requirement of the LDO and support stable sensor-node operation. During BLE packet transmission at 20 ms intervals with −20 dBm transmit power, the average current consumption is 1.49 mA with a peak of approximately 24 mA. The stable communication behavior, evidenced by the absence of packet retransmissions and negligible variation in the transmission interval (20 ± 1.13 ms), provides strong evidence that the power delivered through the metamaterial network is sufficient to sustain continuous sensing and communication. The long-term operational stability enabled by this energy budget is further validated through an extended continuous operation test (Supplementary Fig. 38). This integrated architecture enables fully battery-free, untethered sensor-node operation with continuous low-power sensing and stable data communication, supporting seamless and natural human-machine interactions.

### Human–robot collaboration

With the fully battery-free sensory glove seamlessly powered and connected through the metamaterial-enabled WBSN, we demonstrate its capability for seamless human–robot collaboration by achieving real-time and intuitive robotic hand control. Since the glove operates through the WBSN, the sensor node continuously harvests energy and transmits motion data to the smartphone, which serves as the communication gateway and command processor for the robotic system; the robotic arm and anthropomorphic hand then replicate the detected gestures in real time.

The glove’s gesture-sensing capability is enabled by three strain sensors positioned at the finger joints of the thumb, index, and middle fingers (Fig. 3g). As a finger bends, the corresponding sensor stretches, inducing a proportional resistance change. This resistance change is converted into a voltage signal through a voltage division circuit and digitized by the ADC, then wirelessly transmitted to a smartphone. The raw voltage readings are normalized and mapped to specific finger bending angles. The combined signals from the strain sensors enable decoding of different hand postures. The processed voltage data are subsequently transmitted from the smartphone to the robotic hand’s control system, which drives the robotic fingers to reproduce the user’s finger movements in real time. The resulting robot motions faithfully mirror the user’s gestures, demonstrating a closed-loop realization of seamless, wireless, and energy-autonomous human–robot interaction.

The functionalities of the sensory glove can be further extended by integrating additional sensors of various forms and modalities. For example, miniature IMUs can be incorporated to complement the strain sensors, enabling three-dimensional trajectory tracking of the hand and providing richer control capabilities for human–robot collaboration. To demonstrate the extensibility of the battery-free sensor node and the versatility of the HMI systems, we integrated both strain sensors and an IMU on the back of the hand to control a robotic arm with three degrees of freedom. The operational details of the HMI system are illustrated in Fig. 3h and Supplementary Movies 3 and 4. Since the robotic arm provides an interface for angular velocity control, to achieve intuitive control, we directly map the three-axis angular velocity data from the IMU to the three degrees of freedom of the robotic arm after mean filtering, and then perform closed-loop control through the robotic arm’s back-end controller. Ultimately, this allows the robotic arm to smoothly follow the user’s hand movements. Meanwhile, the strain sensors monitor the finger bending, as demonstrated previously. A coordinated sequence of tasks was carried out by the robotic arm and hand, successfully grasping a peach from a desk and placing it at a designated target location on a shelf. The complete operation includes horizontal and vertical movements in three-dimensional space (steps 1, 2), grasping and transferring the object toward the shelf (steps 3–6), releasing it, and returning to the initial position (steps 7, 8). Throughout this process, the user and robotic system operate synergistically in a smooth and seamless manner. Notably, during dynamic operation, the sensor node maintained continuous connectivity and consistent motion correspondence, confirming the WBSN’s capability to provide stable power delivery and high-rate data transmission for real-time robotic control. Beyond stable operation, this architecture highlights the intrinsic advantages of the metamaterial-enabled WBSN, enabling continuous and battery-free sensing and communication without compromising mobility or comfort. Moreover, the design is inherently scalable, allowing flexible expansion of the sensor node’s function as well as its number, form, and placement across the body.

### Unencumbered and intuitive virtual reality interaction

Building upon its scalable architecture, the battery-free WBSN can be further extended from physical teleoperation to immersive virtual environments, enabling intuitive control through wireless and battery-free sensing. It should be noted that existing virtual reality (VR) or augmented reality (AR) interaction technologies primarily rely on optical and camera-based tracking^9,28^ or handheld controllers^29^. Camera-based systems, while widely adopted, suffer from privacy concerns, environmental dependency, and limited spatial resolution. Accurate tracking requires the user’s hands to remain within the camera’s field of view, making interaction unreliable under occlusion or dynamic lighting conditions. Wearable solutions such as electromyography (EMG) armbands^3,4,30^, IMUs^10,31^, and sensor-equipped gloves^2,8^ offer improved signal accessibility but often compromise wearing comfort and practicality. Many of these systems are dependent on wired connections or bulky batteries, which limit portability and battery life, restricting their suitability for immersive VR/AR applications. In contrast, the proposed battery-free WBSN overcomes these limitations by providing continuous sensing and communication through a lightweight, energy-autonomous network, offering a truly untethered and seamless platform for next-generation immersive interaction.

**Fig. 4:**
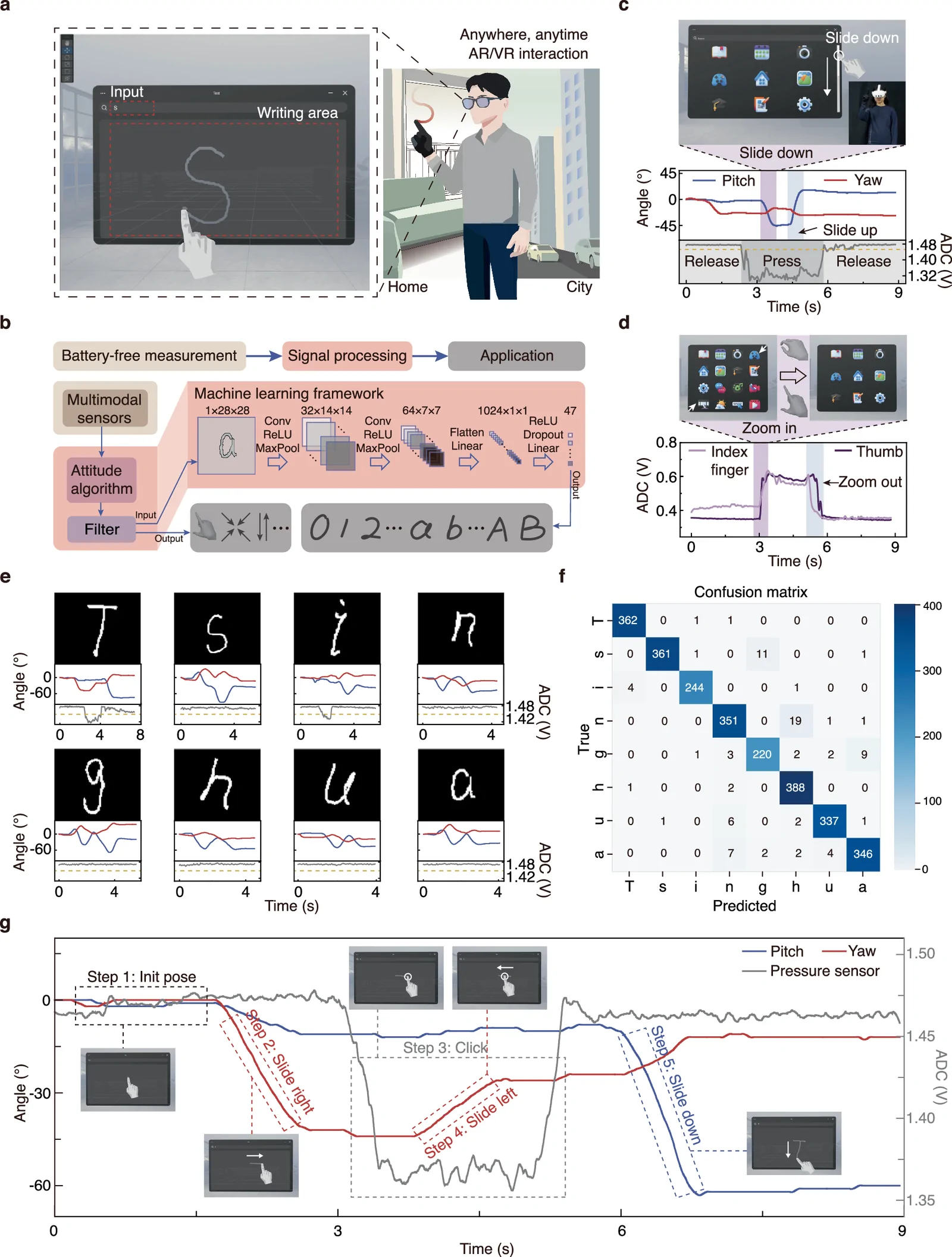
Unencumbered and intuitive virtual reality interaction. **a** Schematics of a user equipped with the battery-free sensory glove engaging in VR interaction across different environments. **b** Flowchart of the intuitive VR interaction framework, divided into three sections: battery-free measurement, signal processing, and application. Real-time manipulation of the VR interface, demonstrating panel sliding (**c**) and window zooming (**d**). During sliding, voltage signals from the pressure sensor and angular data from the fingertip IMU are recorded. During zooming, voltage signals from the strain sensors on the index finger and thumb are captured. **e** Measured voltage and angular signals corresponding to handwriting of each character in “Tsinghua.” **f** Confusion matrix for CNN-based recognition of handwritten characters (400 samples per character). Complete results are provided in Supplementary Fig. 40. **g** Real-time handwriting of the character “T.” Angular data from the fingertip IMU and voltage from the pressure sensor were captured and transmitted by the battery-free sensory glove throughout the process.

To demonstrate this capability, the sensory glove was enhanced for intuitive control in immersive virtual environments (Fig. 4a). Instead of relying on external controllers or power modules, the WBSN transmits both power and sensor data through the metamaterial network, allowing natural gestures to directly control virtual objects in real time. This configuration transforms the human body into an active interface for immersive interaction, free from movement constraints and adaptable to diverse environments. The system workflow is illustrated in Fig. 4b. To enrich the interaction experience, an IMU was mounted on the index fingertip, and a pressure sensor was added to the side of the middle finger, complementing the strain sensors on the thumb and index finger. The IMU tracks three-dimensional fingertip trajectories for position control, the pressure sensor functions as a virtual click, and the strain sensors capture finger bending. With the integration of a machine learning framework, freehand characters written in the air can also be recognized and interpreted. As shown in Fig. 4c, scrolling is achieved by positioning the fingertip over a virtual scrollbar and gently pressing the lateral pressure sensor (Supplementary Fig. 39). The corresponding voltage drop, caused by a decrease in resistance upon light pressing, is detected as a click event. The IMU simultaneously provides direction and velocity information for smooth scrolling. For instance, moving the fingertip upward scrolls the panel up, with speed intuitively modulated by finger movement. Rescaling is realized through pinching motions between the thumb and index finger, where bending triggers zoom-out and extension triggers zoom-in (Fig. 4d). The multimodal sensor data is transmitted via BLE to the VR engine, which decodes the gestures and triggers the corresponding virtual operations.

To evaluate fine-grained control enabled by the battery-free WBSN, we implemented handwriting recognition as a representative intelligent interaction task. The fingertip-mounted IMU continuously captures motion trajectories, while the pressure sensor identifies pen-down and pen-up states for stroke segmentation. This combination allows users to perform freehand writing in the air, with each stroke accurately recorded and distinguished. As shown in Fig. 4e, the sensory glove successfully captured handwritten trajectories of characters such as “Tsinghua.” Each character consists of multiple strokes separated by the pressure signal: when the voltage output exceeds a defined threshold, the trajectory is recorded as an active stroke; when it falls below the threshold, the system registers a pen-up state and pauses recording. The open-source dataset EMNIST^32^ was used for training and validating the model, which includes numbers, uppercase letters, and lowercase letters. A lightweight convolutional neural network (CNN) was employed to classify the handwritten trajectories (Fig. 4f and Supplementary Fig. 40). Testing on the held-out dataset achieved a recognition accuracy of 82.8%. This result suggests that the captured motion features contain discriminative information for character-level recognition, although the performance is affected by several practical factors, including relative displacement between the textile glove and the fingers during motion, residual IMU drift despite short-term calibration, and error amplification when reconstructing 2D trajectories from 3D in-air handwriting signals. For multi-stroke letters such as “T” and “i,” users activate the pressure sensor to mark a pen-up state between strokes (Fig. 4g). The time intervals between strokes are automatically detected, enabling segmentation of continuous writing sequences. A video demonstration of the intuitive VR interaction is provided in Supplementary Movie 5. This demonstration verifies that the WBSN not only supports dynamic and expressive gesture control but also enables real-time intelligent input within virtual environments. Together, these results establish a foundation for future integration of on-device learning, semantic interpretation, and adaptive multimodal interaction, paving the way toward embodied and energy-autonomous interfaces for next-generation immersive systems.

### Untethered full-body motion digital twin

**Fig. 5:**
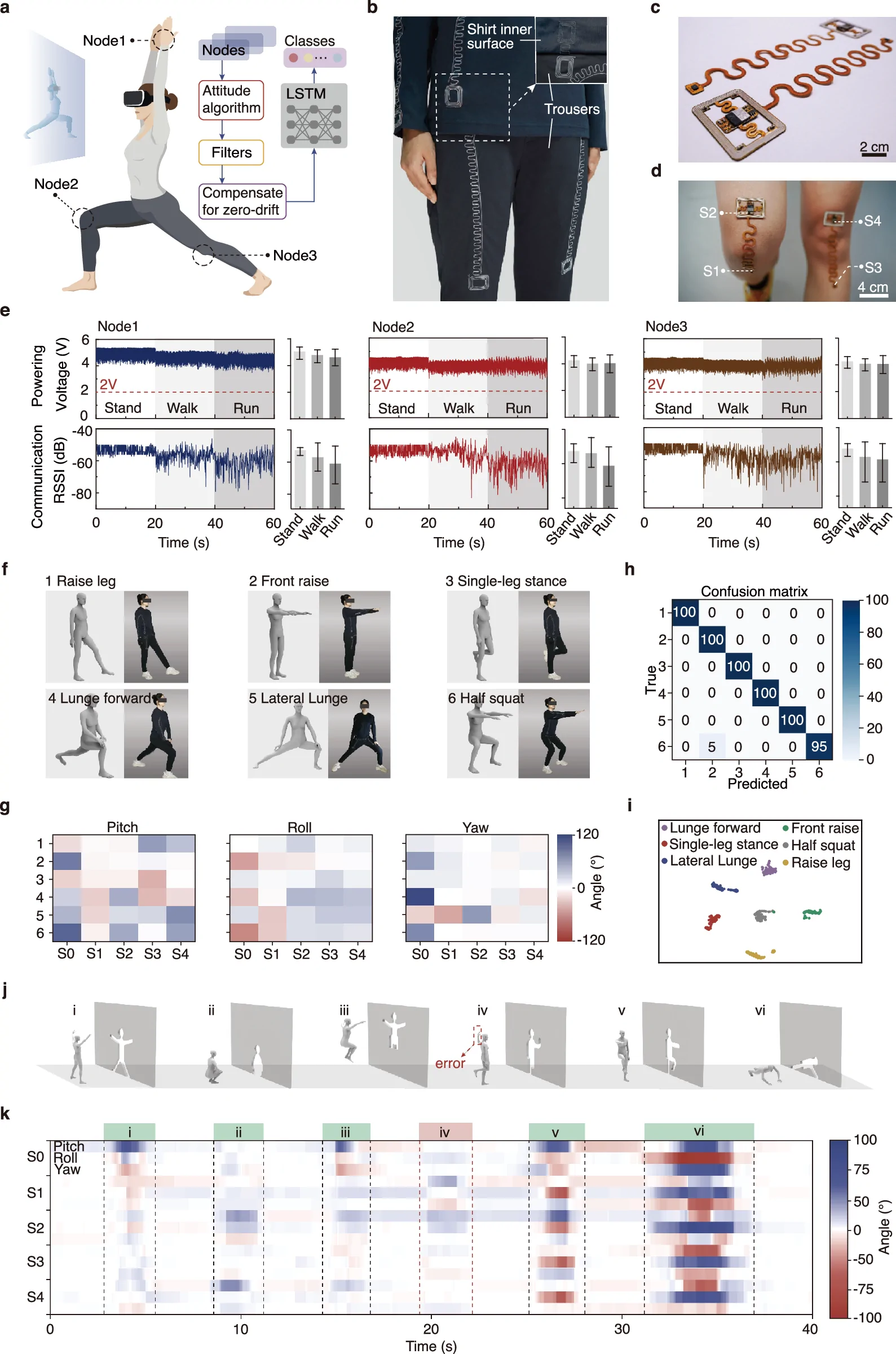
Untethered full-body motion digital twin. **a** Schematic of the full-body motion digital twin based on the battery-free WBSN, with a block diagram illustrating the data processing flow. **b** Images of the metamaterial-integrated shirt and trousers for guiding wireless power and signals across the whole body. The inset shows the overlapping area between shirt and trousers, where wireless coupling enables power and signal transmission across the gap. **c** Photograph of battery-free epidermal sensor nodes, each configured with two IMUs. **d** Photograph of the epidermal sensors worn on the legs, where S1–S4 indicate the positions of the four IMU sensors. **e** Wireless powering and communication performance during physiological activity. Rectified voltage and RSSI from each node were recorded, with average values compared across different activities. Error bars show mean ± s.d. of voltage and RSSI values calculated separately across the indicated period. The red dashed line indicates the minimum input voltage required for stable operation of the LDO chip. Comparison of various poses recorded by the camera and the same poses recovered by our model (**f**) from the triaxial angle frames (**g**) of battery-free WBSN. S0 is located on the hand, and the positions of S1–S4 are shown in (**d**). **h** Confusion matrix for LSTM recognition of the six poses. **i** *t*-SNE plot from the pose dataset recorded by the battery-free WBSN. Distinct clusters corresponding to each pose illustrate the discriminative capability of the WBSN. **j** Demonstration of full-body motion digital twin for VR gaming. **k** Tri-axial angular data collected from battery-free sensor nodes during VR gaming, where green and red indicate correct and incorrect poses, respectively.

Extending beyond hand-based interaction, we further demonstrate the scalability of the WBSN for full-body motion sensing and digital twin reconstruction, enabling holistic and immersive representation of human movement (Fig. 5a). The system aims to reconstruct distributed body kinematics in real time, forming a digital twin that mirrors physical movements within virtual space. To validate the scalability and robustness of the WBSN architecture across the whole-body region, we developed a distributed sensing network capable of capturing motion signals from multiple body segments simultaneously. The system integrates three wireless sensor nodes powered and connected through dual-mode metamaterial textiles that extend from the upper to the lower body, enabling synchronized, untethered, and continuous motion capture.

The overall configuration of the full-body WBSN is shown in Supplementary Fig. 41. Constructing a dual-channel WBSN across the entire body presents the challenge of bridging electromagnetic signals at different frequencies across discrete garments. As illustrated in Fig. 5b, the dual-mode metamaterial textiles integrated into the shirt and trousers feature overlapping coils in the pocket regions, enabling simultaneous NFC excitation of the two subsystems and seamless BLE signal propagation along both garments. When a smartphone is placed in the pocket, the metamaterial pathways guide 13.56 MHz power and 2.4 GHz data signals between nodes, forming a unified framework for wireless power delivery and communication. The upper garment supports the glove node for upper-limb motion capture, while the lower garment integrates two epidermal sensor nodes (Fig. 5c, d), each equipped with two IMUs on the thighs and calves, to track lower-limb movements (Supplementary Fig. 42). Figure 5a shows the algorithm processing flow of the IMU. We used the Mahony algorithm to calculate the Euler angles of the IMU and performed low-pass filtering on the result. In order to reduce the zero drift of the IMU sensor output, we recorded the zero drift of the IMU for 1 s during the initialization process and compensated for it in the subsequent output results. A complete epidermal sensor node weighs only 1.048 g. Once activated by the smartphone, all nodes operate synchronously, achieving full-body connectivity without additional transceivers or batteries. This architecture enables real-time collection and processing of motion data from multiple body segments, establishing a robust foundation for large-scale motion reconstruction and digital embodiment applications.

To evaluate the power delivery and communication stability under dynamic conditions, the full-body WBSN was tested using three active sensor nodes located on the hand and both legs, incorporating five IMUs on the hand, right calf, right thigh, left calf, and left thigh (S0–S4). Each node continuously monitored its supply voltage and signal quality during three representative motion states: standing, walking, and running. As shown in Fig. 5e, the rectified voltages at all nodes remained consistently above the 2.0 V input threshold across all activities, satisfying the requirement for stable circuit operation. During high-intensity motion such as running, minor voltage fluctuations were observed due to transient coil misalignment. The minimum voltages measured at Node 1, Node 2, and Node 3 were 3.48, 2.89, and 2.80 V, respectively, all of which exceed the input requirement of the LDO chip. Moreover, the regulated outputs of all nodes maintained sufficient margins to ensure uninterrupted operation. Under more severe motion conditions, this voltage margin may be further reduced. No disconnection or restart events occurred during continuous testing, confirming robust near-field power coupling through the metamaterial relay network. Compared with voltage stability, body motion exerted a slightly greater influence on the Bluetooth RSSI. The RSSI exhibited moderate fluctuations across different motion states but remained well within the margin required for reliable communication. These variations primarily affected transmission latency rather than link continuity, and the observed delay increase remained acceptable for real-time motion analysis. Quantitative analysis of transmission latency during high-intensity motion (Supplementary Fig. 43) further shows that the delay remains consistently below 20 ms across all nodes, supporting reliable real-time data transfer under dynamic conditions. Importantly, during all activities, BLE connections among all nodes were maintained without packet loss or communication dropouts. These results verify that the WBSN can stably sustain wireless power delivery and continuous data transmission across multiple moving nodes, demonstrating the intrinsic robustness of the dual-mode metamaterial network for large-scale, battery-free sensing in dynamic environments.

The robust performance of the full-body WBSN enables comprehensive motion capture, leveraging five distributed IMUs across the upper and lower body to reconstruct full-body dynamics in real time. Each IMU generated tri-axial orientation signals (roll, pitch, and yaw), resulting in a total of 15 continuous data channels. The WBSN enabled all sensors to operate synchronously, allowing time-aligned acquisition of multi-segment kinematic information under fully battery-free conditions. Six representative postures were selected to evaluate the system’s recognition performance (Fig. 5f): leg raise, front raise, single-leg stance, forward lunge, lateral lunge, and half squat. Distinct signal patterns were observed across these actions, as illustrated in Fig. 5g, where the thigh and calf IMUs exhibit coordinated angular trajectories corresponding to the degree and direction of limb extension, confirming that the distributed IMUs capture the characteristic kinematic correlations between body parts. A long short-term memory (LSTM) neural network was employed to classify these multi-channel temporal sequences (Fig. 5h, i). The model was trained using the 15-dimensional sensor data as sequential inputs and optimized for six-class posture recognition. We collected 30, 34, and 36 sets of data for each action from three subjects (100 samples per class in total). The dataset was divided into training and test sets with a 4:1 ratio. The test set contained samples from all participants, providing an initial evaluation under mixed-subject conditions. The trained LSTM model achieved an average recognition accuracy of 99.5% in three rounds of test set validation. The high recognition accuracy can be partly attributed to the pronounced morphological distinctiveness of the limb postures across the tested movements. The confusion matrix in Fig. 5g displays minimal misclassification among adjacent motion types, while the performance summary in Fig. 5i indicates high discrimination capability and precision. However, due to the limited number of participants, definitive conclusions regarding the model’s generalization ability cannot be drawn from this experiment.

To further validate the practicality and versatility of the proposed WBSN, an interactive VR game was developed, in which task progression is determined by recognizing users’ completion of predefined movements, thereby highlighting the system’s effectiveness for full-body motion capture and real-time human-machine interaction (Fig. 5j). The avatar’s pose is continuously updated using the data streams from the five distributed IMUs, enabling intuitive body control without the need for handheld devices. A game-based evaluation task was designed to demonstrate the functional utility and real-time tracking capabilities of the WBSN in a dynamic application context. During gameplay, the user was required to replicate target postures to pass through incoming virtual barriers. When the user’s actual pose matched the predefined virtual pose, a green indicator appeared; otherwise, a red signal denoted a miss. Representative examples are shown in Fig. 5j (Supplementary Movie 6), where a misaligned hand direction results in detection failure, as seen in panel (iv). The corresponding temporal variations of IMU signals throughout the game sequence are illustrated in Fig. 5k (Supplementary Fig. 44), each exhibiting distinct motion signatures correlated with specific postures. This interactive demonstration highlights the capability of the WBSN to achieve real-time digital embodiment, where physical body movements are directly mirrored within virtual environments by fully battery-free sensors. The results confirm that the developed WBSN enables robust, multi-node motion reconstruction and seamless mapping between physical and virtual domains, laying the technological foundation for untethered full-body digital twins and next-generation immersive HMIs.

## Discussion

We have reported a wireless sensor network based on dual-mode metamaterial textiles that can wirelessly power and interconnect heterogeneous battery-free sensors distributed throughout the body, enabling unencumbered and continuous human-machine interactions. Leveraging computer-aided digital embroidery, we fabricated metamaterial textiles with liquid metal fibers that can be seamlessly integrated into everyday clothing, forming a body-scale wireless communication and power network. We demonstrated that the dual-mode metamaterial design supports simultaneous high-efficiency data transmission and wireless power transfer, achieving more than an order of magnitude (37.8 times) enhancement in data throughput and a twofold improvement in power transfer efficiency compared with emerging near-field clothing. These capabilities eliminate the need for wired connections or bulky distributed batteries, allowing free and natural movement during continuous HMI operation in diverse environments. We have validated the system through demonstrations of intelligent hand-gesture recognition for human–robot collaboration and immersive control in VR/AR environments. Moreover, the metamaterial textile maintains robust propagation of dual-channel electromagnetic waves even across discontinuities in clothing, enabling a full-body WBSN capable of generating dynamic digital-twin representations of human motion. The present system operates directly with off-the-shelf antennas embedded in commercial BLE modules and smartphones, eliminating the need for antenna redesign. Future studies may investigate custom antenna designs with optimized current distributions and impedance matching to further improve coupling efficiency and communication robustness in particularly challenging deployment scenarios. This technology paves the way for unconstrained, anytime-anywhere human-machine interactions, with the potential to fundamentally transform how we perceive, engage with, and navigate the digital and physical worlds.

## Methods

### Dual-mode metamaterial textile design

The metamaterial textile is designed by optimizing its geometrical parameters to simultaneously enable powering and communication modes. The metamaterial textile consists of an SSP structure and planar coil patterns. The SSP exhibits the desired wavenumber characteristics over the 2.4–2.5 GHz industrial, scientific and medical (ISM) frequency band, supporting surface-plasmon-like dispersion relations with wavenumber *β* of the form

1 $$\beta={k}_{0}\sqrt{{\varepsilon }_{1}{\varepsilon }_{2}/({\varepsilon }_{1}+{\varepsilon }_{2})}$$

where *k*_0_ = *ω*/*c* is the free-space wavenumber, *ε*_1_ is the dielectric permittivity, and *ε*_2_ is the permittivity of the metal. To evaluate the influence of the substrate material, the fabric substrate is assigned a dielectric constant of *ε*_polyester_ = 1.9. The SSP waveguide is formed by periodically arranging rectangular structural units (14 mm in height and 6 mm in width) at intervals of 6 mm. The SSP waveguide serves as a transmission line for 13.56 MHz power transfer and supports high-speed 2.4 GHz signal transmission in the form of wireless surface waves. The planar coil patterns are integrated with the SSP structure that enable 13.56 MHz power distribution between physically separated locations.

### Fabrication of the metamaterial textile

The patterns of the metamaterial were designed using commercial software (PE-DESIGN 10, Brother) and imported into a digital embroidery machine (NV180, Brother). Liquid metal fibers-fabricated by filling galinstan (a gallium-indium-tin alloy) into perfluoroalkoxy alkane (PFA) tubes-were loaded into the embroidery machine.

### Numerical simulations

Electromagnetic simulations were carried out with CST Microwave Studio (Dassault Systèmes). The frequency domain solver was used to calculate the field distributions at 13.56 MHz, while the time domain solver was employed to compute the field distributions at 2.4 GHz. Field distributions were calculated using the finite integration technique by applying excitation under three scenarios: with the metamaterial structure alone, with the metamaterial structure placed above human tissue, and without the metamaterial structure placed above human tissue. The computations utilized a computational voxel body model (Gustav, CST Voxel Family) with a resolution of 2 × 2 × 2 mm^3^.

### Design of battery-free sensor node

The sensory glove incorporates four sections of flexible PCB circuits: a wireless sensor node, a thumb unit, an index finger unit, and a middle finger unit. The wireless sensor node integrates an onboard NFC coil(dimensions: 38.4 mm × 25.7 mm, gap: 0.254 mm, number of turns: 6), rectification (CDBQC0130L-HF, Comchip), filtering, and voltage regulation (AP7312-1833, DIODES) circuits, an IMU (LSM6DSOTR, ST) sensor, and an MCU (ISP1807, Insight-Sip) capable of BLE data transmission and reception (Supplementary Figs. 45 and 46). The thumb unit is equipped with a strain sensor. The index finger unit contains an IMU sensor and a strain sensor. The middle finger unit includes a pressure sensor and a strain sensor. The connecting traces for the three finger units all use serpentine wiring to ensure joint flexibility. These three units are connected to the wireless sensor node via micro board-to-board connectors (X0400FVS-10-LPV01, XKB Connection). During assembly, the flexible PCBs are bonded to the glove surface using a heat-activated adhesive film (UltraHold, HeatnBond). The NFC output power of commercial smartphones is governed by international standards, typically ranging between 100 and 300 mW (ISO/IEC 14443-2:2020). To ensure a robust design, we adopted the lower bound of this range (i.e., 100 mW) as our primary consideration. Given that the power transfer efficiency of the metamaterial textile under three-node loading is approximately 20%, the harvested power at each node is approximately 20 mW. Accounting for potential efficiency drops due to coil misalignment during high-intensity motions, we optimized our sensor nodes to operate at a significantly lower power profile (i.e., <10 mW). This ensures a substantial “power buffer,” maintaining continuous operation even under suboptimal coupling conditions. More detailed component elements are listed in Supplementary Figs. 47 and 48.

### Fabrication of the stretchable strain sensor

Strain sensors were fabricated by coating a stretchable fiber with a 1:1 (base:curing agent, w/w) mixture of Ecoflex^TM^ 00-30 (Smooth-On). The coated fibers were cured in an oven at 60 ℃. Subsequently, MWCNTs were uniformly applied onto the outer surface of the cured Ecoflex layer by manual rubbing to form a conductive network. Two copper wires (100 μm in diameter) were then connected and fixed to both ends of the conductive fiber using silver paste, followed by curing at 70 ℃ for 30 min to ensure stable electrical connections. Finally, the conductive fiber was encapsulated with an additional layer of Ecoflex^TM^ 00-30 to provide mechanical protection and enhance durability.

### Fabrication of the fabric-based pressure sensor

The as-received PEDOT:PSS solution was modified by adding 5 wt% DMSO to enhance its electrical conductivity. The fabric-based pressure sensor consists of two main components: an electrode layer and a pressure-sensitive fabric layer. For the electrode layer, pristine cotton textile was immersed in the modified PEDOT:PSS solution for 5 min, followed by curing in an oven at 70 ℃ for 20 min. For the pressure-sensitive fabric, the PEDOT:PSS solution was diluted with deionized (DI) water at a 1:1 ratio (v/v) before undergoing the same dip-coating and curing procedures. Two square textile electrodes were then affixed to a textile substrate using double-sided adhesive tape. Copper wires were connected to each electrode with silver paste and cured at 70 ℃ for 30 min to ensure stable electrical connections. Finally, the pressure-sensitive fabric was placed over the electrode-embedded textile, and a top textile layer was aligned and stitched along the edges to secure the structure. The resulting assembly was fully sealed, completing the fabrication of the fabric-based pressure sensor.

### Materials

MWCNTs (diameter: 10–20 nm, length: 10–30 μm) were purchased from Jiangsu Xianfeng Nanomaterials Science and Technology Co., Ltd. PEDOT:PSS aqueous dispersion (1.5% in water, FY-800) was purchased from Shenzhen Yilai Technology Co., Ltd. DMSO was purchased from Shanghai Aladdin Biochemistry Science and Technology Co., Ltd. (Aladdin, Shanghai, China).

### Wireless measurements

The transmitter was a rectangular copper planar antenna (dimensions: 44 mm × 33 mm, gap: 1.2 mm, number of turns: 4), and the receiver was a rectangular copper planar antenna (dimensions: 37 mm × 27 mm, gap: 0.6 mm, number of turns: 4). A rectangular spiral liquid metal antenna with a metamaterial network structure (dimensions: 40 mm × 30 mm, gap: 1.6 mm, number of turns: 4) was used as the relay network. Both the transmitter and receiver were tuned to a resonant frequency of 13.56 MHz via a matching network. During the experiment, a signal generator was used to input a continuous sinusoidal wave of 13.56 MHz (input power: 20 dBm) to the transmitter, while an oscilloscope (Rigol DS7014, RIGOL Technologies) was employed to measure the output voltage of the receiver. In this study, the wireless power transfer efficiency is defined as the coil-to-coil coupling efficiency between the transmitter and receiver coils, measured in a benchtop setup to provide a consistent and comparable metric for evaluating metamaterial-enabled wireless power transfer systems^20,25,33^.

### Design of the epidermal motion sensor

The epidermal motion sensor is fabricated using a flexible PCB. The rigid electronic components are positioned at the center of the NFC coil (dimensions: 38.4 mm × 25.7 mm, gap: 0.254 mm, number of turns: 6) and connected to the coil via serpentine traces. One of the two IMUs (LSM6DSOTR, ST) is placed at the center of the coil, while the other is mounted 125 mm away from the coil through serpentine wiring. The coil section adheres to the thigh above the knee, and the distal end of the serpentine trace attaches to the shank below the knee. More detailed component elements are listed in Supplementary Figs. 47 and 48.

### Preliminary washability assessment

Washing tests were performed in accordance with the ISO 6330 standard for domestic laundering to evaluate the preliminary durability and functional integrity of the metamaterial textile over repeated wash cycles. The test specimen was placed in a household washing machine together with additional garments to achieve a total load exceeding 2 kg, while the specimen itself was maintained below 1 kg to ensure representative conditions. Each washing cycle lasted 45 min (Supplementary Movie 7). Upon completion, the specimen was retrieved and air-dried under ambient conditions. Its electrical performance and transmission loss were subsequently characterized, and the pre and post-wash variations are summarized in Supplementary Table 2. The results provide a preliminary verification that the textile retained its functionality after three washing cycles, with only limited performance degradation.

### Bluetooth configuration parameters

This work adopts the BLE protocol. The relevant configuration parameters of the protocol stack are presented as follows: The advertising interval: 40 ms; The advertising duration: 180 s; Minimum acceptable connection interval: 20 ms; Maximum acceptable connection interval: 75 ms; Connection supervisory timeout: 4 s; TX power set: −20 dBm.

## Supplementary information

Supplementary Information

Description of Additional Supplementary Files

Supplementary Video 1

Supplementary Video 2

Supplementary Video 3

Supplementary Video 4

Supplementary Video 5

Supplementary Video 6

Supplementary Video 7

Transparent Peer Review file

## Source data

Source data

## Data Availability

Source data are provided with this paper. The other data that support the findings of this study are available from the corresponding authors upon request. Source data are provided with this paper.

## Code availability

The code used in this study is available via GitHub at https://github.com/thu-wbg-zym/WBSN-project.
