# Supplementary material for: Metamaterial-enabled battery-free wireless sensor networks for unencumbered human-machine interactions
Source: Nat Commun. 2026 Sep 14;17:10778. doi: 10.1038/s41467-026-77600-2 (PMC13661596; doi:10.1038/s41467-026-77600-2)
Supplement: Supplementary file 1 [file 41467_2026_77600_MOESM10_ESM.pdf]

nature portfolio

Peer Review File

Metamaterial-enabled battery-free wireless sensor networks for unencumbered human-machine interactions

Corresponding Author: Professor Xi Tian

This file contains all reviewer reports in order by version, followed by all author rebuttals in order by version.

Version 0:

Reviewer comments:

Reviewer #1

(Remarks to the Author)
This manuscript reports a battery-free wireless body sensor network enabled by a dual-mode metamaterial textile that supports 13.56 MHz wireless powering and 2.4 GHz BLE communication on the same embroidered textile pathway. The most noteworthy results are the reported high power-transfer efficiency over relay lengths approaching 1 m, low-latency BLE communication at very low transmit power when the textile is used, multi-node operation, and end-to-end demonstrations in robotic control, VR interaction, and full-body motion sensing. The paper is timely and potentially important for wearable electronics, smart textiles, wireless body networks, and human-machine interaction.

In my view, the work is original mainly at the system architecture level rather than through a fundamentally new electromagnetic effect. Relative to prior single-mode body networks summarized by the authors, the advance here is the integration of efficient near-field power relaying and high-rate SSP-guided BLE communication into one embroidered textile platform, with smartphone-centered battery-free sensing as the target application. That is a meaningful contribution and should interest both this field and related communities.

At the same time, several of the manuscript's strongest practical claims are not yet fully supported by the evidence presented. I therefore do not recommend acceptance in the current form, but I do think the paper has a realistic path to publication after substantial revision. My main comments are below.

Major comments
1. The central claim that a consumer smartphone can practically act as the universal hub is not yet experimentally closed.

The paper is framed around the idea that an unmodified smartphone can both power distributed battery-free nodes and receive their data across the body. However, the most quantitative power-transfer characterization is not performed with a real phone, but with a benchtop setup using a signal generator and matched planar copper antennas. The smartphone demonstrations are compelling qualitatively, but they do not yet establish an end-to-end power/data budget under realistic phone operation, across different phone models, or under simultaneous multi-node loading. Because this point is central to the manuscript’s significance, I think the authors should either add substantially stronger real-smartphone characterization or narrow the practical claim. At minimum, the paper should report phone model(s), NFC operating conditions, node operating margin, and multi-node behavior under real phone excitation.

2. The 2.4 GHz coupling mechanism and antenna architecture are insufficiently described.

The manuscript states that the BLE antenna couples to the textile SSP waveguide, but the node-side antenna geometry, matching, placement, and polarization are not described in enough detail for the reader to assess the RF mechanism. Supplementary Fig. S25 identifies the coil, power-management module, IMU, BLE MCU, and connectors, but it does not clearly isolate the 2.4 GHz coupling structure. The smartphone side is even less clear. Since a conventional smartphone BLE antenna is not designed as a mode-matched coupler to a textile SSP waveguide, the mechanism by which practical coupling is achieved needs more explicit support. This is not a fatal flaw, because the demonstrations suggest the system works, but it is a substantial gap between the claimed mechanism and the reported evidence. I would strongly encourage the authors to provide antenna/coupler details, phone-position tolerance, device dependence, and, if possible, S-parameter or

field-mapping data.

3. Several practical claims are currently stronger than the data presented.

The supplementary comparison table presents this work as machine-washable and highly user-friendly, and the main text also argues that surface-wave confinement improves security by reducing loss and interception risk. However, in the material provided, I could not find dedicated washing data such as post-wash power-transfer efficiency, RSSI, sensor performance, leakage, connector fatigue, or abrasion/sweat robustness. Likewise, the security argument is presently supported mainly by physical intuition and simulation rather than by direct leakage or eavesdropping measurements. These issues do not necessarily prohibit publication, but the authors should either provide supporting experiments or moderate the wording so that the paper remains clearly framed as a proof-of-concept systems study rather than a validated practical platform.

4. Reproducibility and reporting transparency need improvement.

The Methods are good for an interdisciplinary systems paper: the manuscript gives textile geometry, substrate dielectric constant, embroidery machine, sensor-node ICs, and fabrication routes for the strain and pressure sensors. Still, full reproduction would remain difficult because the paper does not provide embroidery pattern files/CAD, Gerbers, matching-network details, firmware, BLE configuration, phone-side software, or ML code and preprocessing. The Data Availability statement is limited to data being available on reasonable request. For a paper whose contribution depends heavily on integrated RF/electronics/textile/ML implementation, I think the transparency bar should be higher.

5. There are several reporting inconsistencies that should be corrected before publication.

In Fig. 2b, h is defined as the vertical distance between the transmitter and receiver. However, in the main Results text, the measurement condition for Fig. 2c, where the relay length L is varied, is described as h = 5 cm, w = 0 cm. In contrast, the caption of Fig. 2 gives the condition for the same Fig. 2c as h = 0.5 cm, w = 0 cm. Moreover, the same section states that the efficiency drops to zero when h > 26 mm, which is not consistent with the high efficiency shown in Fig. 2c if h = 5 cm were correct. Thus, there is at least one typo or description error somewhere in this part.

A further point of confusion is that the lower panel of Fig. 2e on page 28 appears to indicate "h = 5 mm." However, this refers to the fixed condition for a different panel, namely the plot showing the dependence on w. The issue here is specifically the discrepancy between the main text and the caption regarding the relay-length experiment in Fig. 2c, and is separate from the 5 mm label in Fig. 2e.

The second issue is an incorrect reference to the supplementary figures. In the Results text for the VR handwriting recognition experiment, the CNN classification results are said to correspond to Fig. 4f and Supplementary Fig. 32. However, the caption of Fig. 4 states that the complete results are shown in Supplementary Fig. 33. In the actual Supplementary Information, Fig. S32 is the confusion matrix for 47-character recognition, whereas Fig. S33 shows the full-body WBSN architecture. Therefore, this seems to be not a discrepancy in the experimental results themselves, but rather a figure-numbering error, most likely an off-by-one mistake.

The third issue is that the claim of ">100-fold improvement" does not appear to align directly with the numbers shown in the table. The Abstract states that the communication throughput is improved by "over two orders of magnitude," and the Introduction similarly says ">100 times." However, in Supplementary Table I, the data rate of this work is listed as up to 1 Mbps, while the clothing-based near-field coupling system used for comparison is listed as 26.48 kbps. This corresponds to a ratio of only about 37.8x, which would naturally be described as tens-fold, not >100-fold. Please clarify the baseline used for this claim and revise the wording so that it is quantitatively consistent.

Minor comments

1. The manuscript would benefit from clearer separation between simulation results, benchtop characterization, and real on-body experiments, especially in places where the text may currently encourage the reader to overinterpret simulated robustness as experimental proof.

2. For the ML sections, please report participant numbers, number of trials per class, data balancing, cross-subject validation protocol, and uncertainty/statistical variation across runs.

Reviewer #2

(Remarks to the Author)
This study proposes a dual-mode metamaterial network for distributed body sensor systems. It provides a significant contribution to the field of wearable metamaterial clothing by enabling both power transfer and data communication through its design. While the demonstration of a single-hand glove alone might not be entirely unique, the whole-body batteryless sensor network provides a clear advantage to this system. However, I have a few minor concerns related to the clarification and logical support for some arguments included in this paper, though these issues do not limit the overall value of this work.
1. At the top of page 8, the phrase "wireless transmission speed" is ambiguous since the transmission speed in RF refers to the speed of light. A more accurate term would be "wireless data transmission speed".
2. The statement "the received BLE signal strength remains above -50 dB" contains an incorrect unit. It should be expressed

in dBm rather than dB.
3. Regarding the sentence, "Meanwhile, the 2.4 GHz transmission coefficient exceeds -20 dB, indicating that the BLE signal strength is enhanced by more than three orders of magnitude," I recommend rephrasing this to clarify that this is a comparison of efficiency (metamaterial enhanced vs. radiation). Additionally, claiming an enhancement of three orders of magnitude seems rather arbitrary and is accurate only under specific conditions. As seen in Figure 2m, the efficiency S21 with the textile is not universally three orders of magnitude higher than without it.
4. Figure 2f is somewhat confusing. Although a legend is provided, the gray area- which presumably represents the efficiency level without the textile- is not clearly defined in the figure or the caption.
5. "These results confirm that the power delivered through the metamaterial network is sufficient to sustain continuous sensing and communication without noticeable fluctuation or packet loss." The authors need to clarify how they reached this conclusion. It appears the authors concluded this because no packet retransmission occurred on the Bluetooth device and the 20 ms interval was accurately maintained. Adding this explanation would better guide the readers. A stronger statement would include the average transmission interval ± standard error.
6. On page 13, the authors mention that the device is lightweight, but no specific weight values (numbers) are provided.
7. "During high intensity motion such as running, minor voltage fluctuations were observed due to transient coil misalignment; however, the regulated outputs of all nodes maintained sufficient margins to ensure uninterrupted operation." While Figure 5e provides data on voltage and RSSI fluctuations during these activities, the claim of uninterrupted operation is not fully supported by these metrics alone. It is highly recommended to include data on data transmission packet loss (%) and latency during high-intensity motions to support this argument.
8. "A game-based evaluation task was designed to assess the responsiveness and accuracy of the WBSN." This argument is not supported by the results. The responsiveness and accuracy of the WBSN are not demonstrated in this task (only angular data is provided). The authors should either rephrase this sentence or provide the supporting results.

Reviewer #3

(Remarks to the Author)
The manuscript reports a battery-free wireless body sensor network based on dual-mode metamaterial textiles for continuous and unobtrusive human-machine interaction. By digitally embroidering liquid-metal fibers into conductive textile patterns, the authors construct ultra-wideband electromagnetic pathways that simultaneously support wireless power transfer (13.56 MHz) and wireless communication (2.4 GHz), enabling distributed battery-free sensors to operate across the full body. The system integrates near-field power delivery with low-latency wireless data transmission and demonstrates applications in gesture recognition, robotic control, virtual/augmented reality interaction, and human digital twins. Overall, the manuscript presents a well-designed system with convincing demonstrations. The work is relevant to the development of wearable electronics and next-generation human–machine interfaces. Moreover, the manuscript presents a relatively complete system-level demonstration, and overall the work is well organized and clearly presented. However, the manuscript could be further strengthened by addressing the following points for improved technical clarity and rigor:

1. Regarding the operating principle, the dual-mode scheme integrating 13.56 MHz inductive coupling and 2.4 GHz surface-wave guiding is interesting. However, a clearer explanation of how the SSP structure simultaneously supports these two widely separated frequency regimes without mutual interference would strengthen the manuscript. In addition, the claims regarding higher efficiency and enhanced security appear largely qualitative and would benefit from clearer quantitative justification.
2. The manuscript presents electromagnetic field simulations to explain energy distribution across multiple nodes. However, the manuscript presents electromagnetic simulations to illustrate energy distribution across multiple nodes. However, the connection between the simulated field confinement and the experimentally measured transmission performance could be discussed more explicitly.
3. The system architecture is clearly illustrated. However, a more explicit discussion of the relationship between the harvested power and the circuit power consumption would help clarify the energy budget for battery-free operation. In addition, the claim of continuous low-power sensing and stable data communication would benefit from a clearer discussion of long-term operational stability.
4. The human-robot interaction demonstration is interesting. However, the manuscript could more clearly highlight how the proposed metamaterial-enabled WBSN improves system-level performance compared with conventional wireless wearable control approaches.
5. The description of the IMU integration is relatively brief. Additional clarification on the IMU signal processing and its specific role in the control strategy would improve technical clarity.
6. In Fig. 5g, some text annotations overlap with the heatmap regions, making local variations difficult to distinguish. The figure layout could be refined for improved readability.
7. The formatting of numerical values and units (e.g., “2.4 GHz”, “13.56 MHz”, and angle symbols such as “90°”) is not fully consistent across several figures and axis labels. A thorough check of unit formatting throughout the figures (e.g., Figs. 3 and 4, Supplementary Figs. S10, S13, S17, and S23, among others) is recommended according to SI conventions.

Version 1:

Reviewer comments:

Reviewer #1

(Remarks to the Author)
Many of the individual technical elements, including metamaterial-assisted surface-wave communication on textiles, have been previously demonstrated. The present work appears primarily as a large-scale system integration effort rather than a fundamentally new wireless or metamaterial concept. Precisely because the contribution lies in systems integration, I had hoped the manuscript would more clearly disclose the practical key techniques that allow others to reproduce and build upon the integrated platform.
In particular, the previously requested S-parameter and field-mapping data characterizing the node-side coupling to the textile SSP waveguide are addressed largely through theoretical reasoning. The authors invoke the TM nature of the surface mode and the general current distribution of an inverted-F antenna, but do not provide measured S21 or impedance-matching data between the node antenna and the textile. The manuscript also indicates that the chip antenna integrated into the BLE MCU is used as-is. Such chip antennas are typically designed and matched for free-space radiation, and one would expect substantial detuning when placed against a textile SSP waveguide. The system clearly functions in the demonstrations, but as a systems integration study it remains somewhat unclear what design takeaway the reader should carry forward regarding how to engineer the node-side coupling for similar platforms.
I recognize that this regime is one in which device-level implementation know-how plays an outsized role, and I do not wish to press this point too strongly. The comment is offered as a suggestion for sharpening the practical value of the contribution rather than as a barrier to publication.

Reviewer #2

(Remarks to the Author)
I would like to thank the authors for their thorough revisions. The authors have satisfactorily addressed all of my minor concerns raised. I appreciate the addition of quantitative data regarding transmission latency and packet loss. I believe the manuscript has been significantly improved in terms of scientific soundness and clarity.

Reviewer #3

(Remarks to the Author)
The revision shows substantial effort with new data and figures. However, several first-round issues remain partially resolved, and new concerns have emerged.

[Major concerns]

1. Only 3 Chinese-market Android phones tested. No iPhone or Samsung included despite fundamentally different NFC implementations. Fig. S32 reports rectified voltage only, not power transfer efficiency. Please test iPhone/Samsung, or narrow claim to "NFC-enabled Android smartphones."

2. BLE connection parameters (advertising interval, TX power, etc.) and power management component values still missing. These are essential for reproducing the core system-level contribution. Please add BLE parameters and circuit values to Methods/SI.

3. 3 wash cycles cannot support "machine washable" claim (consumer standard: 50+ cycles). No quantified pre/post-wash performance data provided. Please report explicit degradation values, or reframe as "preliminary washability assessment."

4. NFC coil radiates omnidirectionally and is not confined by the SSP structure. Security advantage applies only to 2.4 GHz BLE, not the entire system. Please limit security claim to BLE channel explicitly.

5. 20% efficiency figure from benchtop, not smartphone excitation. No worst-case analysis despite visible voltage fluctuations during running (Fig. 5e). Please report minimum rectified voltage during motion and confirm it exceeds LDO dropout threshold.

6. N=3 subjects; random train/test split (not LOSO) inflates 99.5% accuracy; 6-class pose task is trivially separable; handwriting accuracy (82.8%) is low but undiscussed. Please acknowledge N=3 limitation, clarify that cross-subject generalization was not assessed, discuss handwriting accuracy limitations.

7. Comparison is against free-space propagation around a bent arm (naturally extreme path loss), not a fair baseline. A simple conductive trace would be more informative. Please add conductive trace comparison or moderate the language.

8. Phone battery drain dominated by screen/OS, not NFC; RSSI stability not quantified; IMU gyroscope drift over 6 hours not characterized. Please report RSSI mean±std, acknowledge IMU drift, remove or qualify "12 hours" projection.

[Minor comments]

9. Grammar: "excite" → "excites" in Section 2C.

10. "More than an order of magnitude" repeated 3x without stating actual value — include in Abstract.

11. Fig. 2m: clarify whether data points have error bars or are single measurements.

Version 2:

Reviewer comments:

Reviewer #3

(Remarks to the Author)
The authors have revised the manuscript NCOMMS-26-013708, titled "Metamaterial-enabled battery-free wireless sensor networks for unencumbered human-machine interactions," to address the second-round comments. The revision includes new experimental data such as the Samsung S23 measurement (Supplementary Fig. 37), straight-line and bent-arm conductive-trace baselines (Supplementary Figs. 27-28), the minimum rectified voltage during running, the quantified RSSI and IMU drift over 6 hours, and a preliminary washability table. The BLE configuration parameters and the power management circuit schematic are also newly disclosed, and the previously overclaimed expressions have been appropriately moderated. In this regard, I am satisfied with the overall direction of the revision, and most of my prior concerns seem to be adequately addressed. Only a couple of points may need to be mentioned further, none of which are critical. My assessment of the specific responses is as follows.

1. Regarding the universal smartphone hub claim, the addition of the Samsung S23 measurement and the consistent replacement with "NFC-enabled Android smartphone" throughout the manuscript is a reasonable resolution. The explanation that iOS integration is mainly a product-engineering task rather than a scientific limitation appears acceptable. I accept this response.

2. The BLE configuration parameters (advertising interval, connection interval range, supervision timeout, TX power) and the power management circuit schematic in Supplementary Fig. 48 provide sufficient detail for the system to be reproduced. This point is resolved.

3. The washability claim is now framed as a "preliminary washability assessment," and the pre- and post-wash performance is quantified in Supplementary Table 2. I appreciate that the authors chose to downgrade the language rather than defend the original expression. It would be beneficial to add one summary row showing the raw percentage change to Table 2 for easier interpretation.

4. The security claim is now explicitly limited to the BLE channel, and the manuscript clarifies that the NFC link carries only power. This is acceptable.

5. Reporting the minimum rectified voltages during running (3.48, 2.89, and 2.80 V) with the LDO threshold marked on Fig. 5e directly answers the worst-case concern. The margin at Node 3, about 0.8 V above the 2.0 V threshold, seems adequate but not large. A brief note in the Discussion that this margin may narrow under more extreme motion regimes would be helpful for the readers.

6. For the ML validation, the authors have added the acknowledgement that N = 3 does not permit conclusions regarding cross-subject generalization, and the mechanisms behind the lower handwriting accuracy are discussed. The leave-one-subject-out evaluation was not performed, and instead the ML demonstrations are reframed as end-to-end functional showcases rather than ML claims. Given that the central contribution of the paper is the metamaterial-textile system, I think this reframing is reasonable. One additional sentence noting that the 99.5% posture accuracy also reflects the fact that the six poses involve distinct limb configurations would improve the calibration further. This is only a suggestion.

7. The new Supplementary Figs. 27 and 28 comparing the SSP metamaterial with a simple conductive trace, under both straight and bent-arm configurations, directly address the previous concern about the "three orders of magnitude" comparison. The language in the main text has also been appropriately hedged. This concern is now handled.

8. The RSSI is reported as -54.34 +/- 4.72 dB over the 6-hour window, the static IMU drift is quantified per axis (0.69, 0.12, and 0.22 degrees for roll, pitch, and yaw), and the "12 hours of continuous operation" projection has been removed. All three sub-points are addressed.

9. The grammatical correction in Section 2C, the inclusion of the 37.8x figure in the Abstract, and the clarification that the Fig. 2m data points are single measurements are all reflected in the revised text. These minor points are resolved.

Overall, the manuscript appears to have reached the level appropriate for Nature Communications. The engineering achievement is substantial, the claims are now proportional to the evidence, and the reproducibility gaps have been closed. I have no further major concerns and recommend acceptance, subject to the minor editorial suggestions above.

Open Access This Peer Review File is licensed under a Creative Commons Attribution 4.0 International License, which permits use, sharing, adaptation, distribution and reproduction in any medium or format, as long as you give appropriate credit to the original author(s) and the source, provide a link to the Creative Commons license, and indicate if changes were made.
In cases where reviewers are anonymous, credit should be given to 'Anonymous Referee' and the source.
The images or other third party material in this Peer Review File are included in the article's Creative Commons license, unless indicated otherwise in a credit line to the material. If material is not included in the article's Creative Commons license and your intended use is not permitted by statutory regulation or exceeds the permitted use, you will need to obtain permission directly from the copyright holder.
To view a copy of this license, visit https://creativecommons.org/licenses/by/4.0/

Dr. Xi Tian
Tsinghua University
Institute of Data and Information
Information Building 1108
Shenzhen 518055, China
Email: tianxi@sz.tsinghua.edu.cn

March 25, 2026

Re: Nature communications manuscript NCOMMS-26-013708, Yimeng, et al "Metamaterial-enabled battery-free wireless sensor networks for unencumbered human-machine interactions"

To the reviewers,

We sincerely appreciate the reviewer's thorough evaluation of our manuscript and the constructive feedback provided. Your comments have been invaluable in helping us refine and strengthen our work. The main text has been extensively revised to address the raised concerns, which are now highlighted in blue in the revised manuscript. We firmly believe that these revisions have significantly enhanced the quality of the manuscript. As detailed below, we address each of your concerns in detail.

Reviewer #1

This manuscript reports a battery-free wireless body sensor network enabled by a dual-mode metamaterial textile that supports 13.56 MHz wireless powering and 2.4 GHz BLE communication on the same embroidered textile pathway. The most noteworthy results are the reported high power-transfer efficiency over relay lengths approaching 1 m, low-latency BLE communication at very low transmit power when the textile is used, multi-node operation, and end-to-end demonstrations in robotic control, VR interaction, and full-body motion sensing. The paper is timely and potentially important for wearable electronics, smart textiles, wireless body networks, and human-machine interaction.

In my view, the work is original mainly at the system architecture level rather than through a fundamentally new electromagnetic effect. Relative to prior single-mode body networks summarized by the authors, the advance here is the integration of efficient near-field power relaying and high-rate SSP-guided BLE communication into one embroidered textile platform, with smartphone centered battery-free sensing as the target application. That is a meaningful contribution and should interest both this field and related communities.

At the same time, several of the manuscript's strongest practical claims are not yet fully supported by the evidence presented. I therefore do not recommend acceptance in the current form, but I do think the paper has a realistic path to publication after substantial revision. My main comments are below:

Reply: We thank the reviewer for the positive comments and for recognizing the broad interest and potential impact of this work. We acknowledge the reviewer's concern that some claims were not sufficiently supported in the original manuscript. In response, we have substantially revised the manuscript by adding new experimental results, strengthening the quantitative analysis, and expanding the technical descriptions and discussions throughout the main text.

We believe these revisions have addressed the reviewer's concerns and significantly improved the rigor and clarity of the manuscript. Detailed point-by-point responses are provided below.

Major comments:

1. *The central claim that a consumer smartphone can practically act as the universal hub is not yet experimentally closed.*

The paper is framed around the idea that an unmodified smartphone can both power distributed battery-free nodes and receive their data across the body. However, the most quantitative power-transfer characterization is not performed with a real phone, but with a

benchtop setup using a signal generator and matched planar copper antennas. The smartphone demonstrations are compelling qualitatively, but they do not yet establish an end-to-end power/data budget under realistic phone operation, across different phone models, or under simultaneous multi-node loading. Because this point is central to the manuscript's significance, I think the authors should either add substantially stronger real-smartphone characterization or narrow the practical claim. At minimum, the paper should report phone model(s), NFC operating conditions, node operating margin, and multi-node behavior under real phone excitation.

Reply: We thank the reviewer for recognizing the compelling qualitative smartphone demonstrations, and for highlighting the need to more rigorously substantiate the role of the smartphone as a universal hub.

First, we clarify the rationale for employing a benchtop setup in the quantitative power-transfer characterization. Commercial smartphones exhibit variability in form factor, internal circuitry, and NFC output power (typical ~100-300mW) [R1], which can lead to measurement variability across different phones. By contrast, the benchtop setup provides well-defined and controlled excitation conditions, thereby enabling an accurate evaluation of the intrinsic power-transfer characteristics of the proposed system. This approach is widely adopted in prior research on textile-based metamaterials [R2-R4].

We fully agree that providing quantitative results under real-phone excitation would strengthen the practical claim of the smartphone acting as a universal hub. We have added new experimental results in Supplementary Fig. S32 to test the power transfer performance under realistic phone operation. Supplementary Fig. S32e presents the rectified voltage of a batter-free sensor node powered by three different phone models (OnePlus, Huawei, Xiaomi). These rectified voltages all exceed 8 V, which is well above the minimum operating voltage of 1.8 V required by the sensor nodes. We further assessed multi-node operation under different smartphone excitation conditions (Supplementary Fig. 32f). The rectified voltages remain sufficiently high to support stable concurrent operation across multiple nodes. These results demonstrate a robust operating margin under practical, non-ideal smartphone operation conditions and further substantiate the feasibility of using a consumer smartphone as a universal hub. We have updated the Results section of the manuscript to include these quantitative findings:

"Quantitative evaluation of power transfer performance under realistic smartphone operation is provided in Supplementary Fig. 32, demonstrating a substantial operating margin under practical, non-ideal conditions and further supporting the feasibility of using a consumer smartphone as a universal hub"

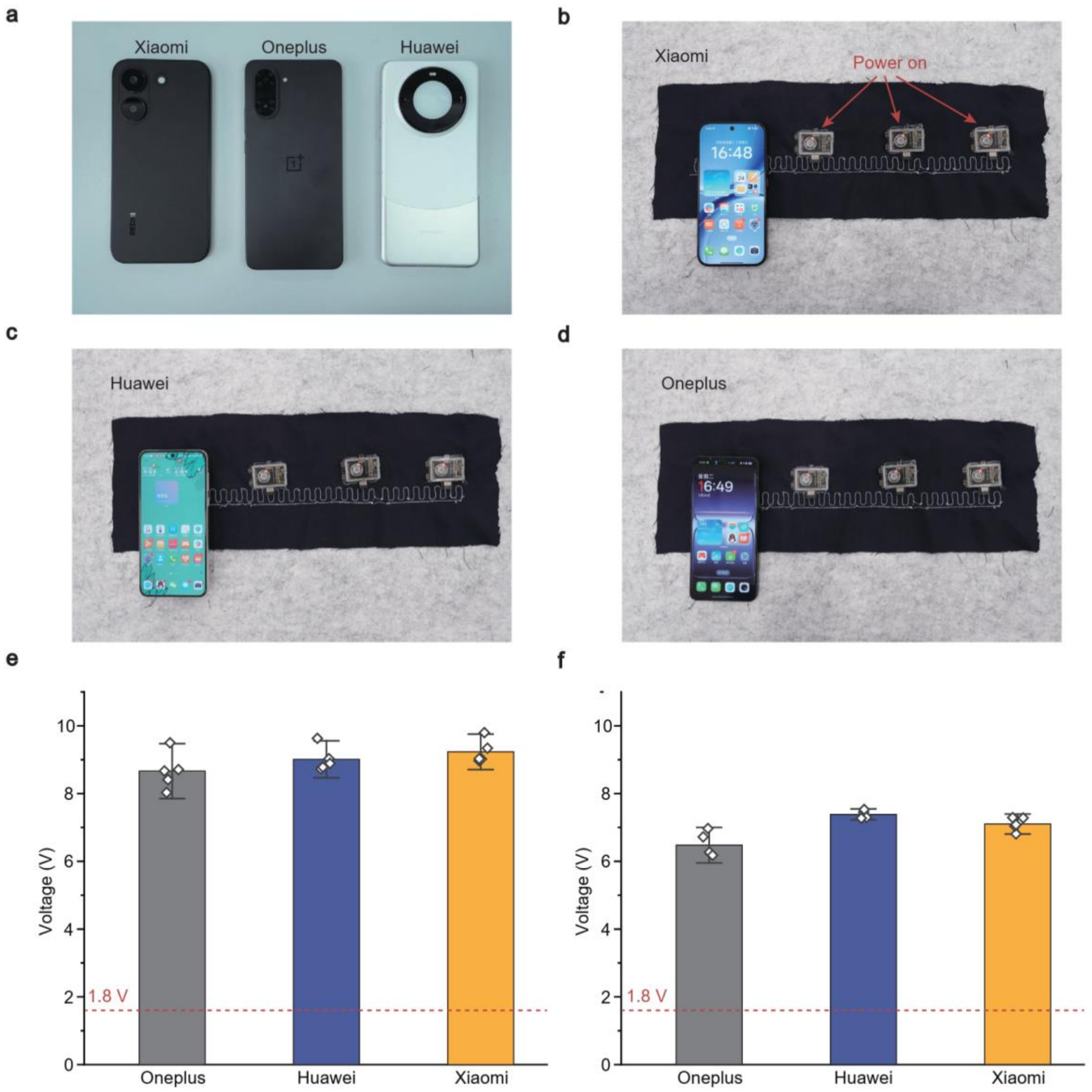

Fig. S32. Quantitative characterization of end-to-end power transfer using commercial smartphones. (a) Photographs of the three commercial smartphone models utilized for validation (OnePlus Ace5, Huawei Mate60, and Xiaomi Redmi turbo5). (b-d) Experimental demonstrations of the smartphone-powered WBSN under a simultaneous three-node loading condition. (e) Measured rectified voltage of a single batter-free sensor node powered by three different smartphone models. (f) Average rectified voltage across three concurrent battery-free sensor nodes powered by three different smartphone models.

[R1] ISO/IEC 14443-2:2020 Cards and security devices for personal identification — Contactless proximity objects — Part 2: Radio frequency power and signal interface (ISO, 2020).

[R2] Lin, Rongzhou, et al. "Wireless battery-free body sensor networks using near-field-enabled clothing." *Nature communications* 11.1 (2020): 444.

[R3] Lin, Rongzhou, et al. "Digitally-embroidered liquid metal electronic textiles for wearable wireless systems." *Nature communications* 13.1 (2022): 2190.

[R4] Zhu, Xia, et al. "A robust near-field body area network based on coaxially-shielded textile metamaterial." *Nature Communications* 15.1 (2024): 6589.

2. *The 2.4 GHz coupling mechanism and antenna architecture are insufficiently described.*

The manuscript states that the BLE antenna couples to the textile SSP waveguide, but the node-side antenna geometry, matching, placement, and polarization are not described in enough detail for the reader to assess the RF mechanism. Supplementary Fig. S25 identifies the coil, power-management module, IMU, BLE MCU, and connectors, but it does not clearly isolate the 2.4 GHz coupling structure. The smartphone side is even less clear. Since a conventional smartphone BLE antenna is not designed as a mode-matched coupler to a textile SSP waveguide, the mechanism by which practical coupling is achieved needs more explicit support. This is not a fatal flaw, because the demonstrations suggest the system works, but it is a substantial gap between the claimed mechanism and the reported evidence. I would strongly encourage the authors to provide antenna/coupler details, phone-position tolerance, device dependence, and, if possible, S-parameter or field-mapping data.

Reply: We thank the reviewer for raising this important point for elaboration. We agree that a rigorous description of the 2.4 GHz coupling mechanism is essential for a complete understanding of the system's performance. We have included a new supplementary note (B. Surface wave excitation) and supplementary figure (Fig. S5) to describe the physics of surface mode excitation by a nearby radio-frequency source (antenna) as requested by the reviewer. The key result is Eq. (5), which shows that the excitation of surface modes depends only on the overlap in the current density supported by the antenna and the electric field of the mode. Because the supported surface plasmon-like mode is transverse magnetic (TM) in nature, excitation can occur as long as the antenna has a nonzero source-current component along either the longitudinal (x) or vertical (z) direction.

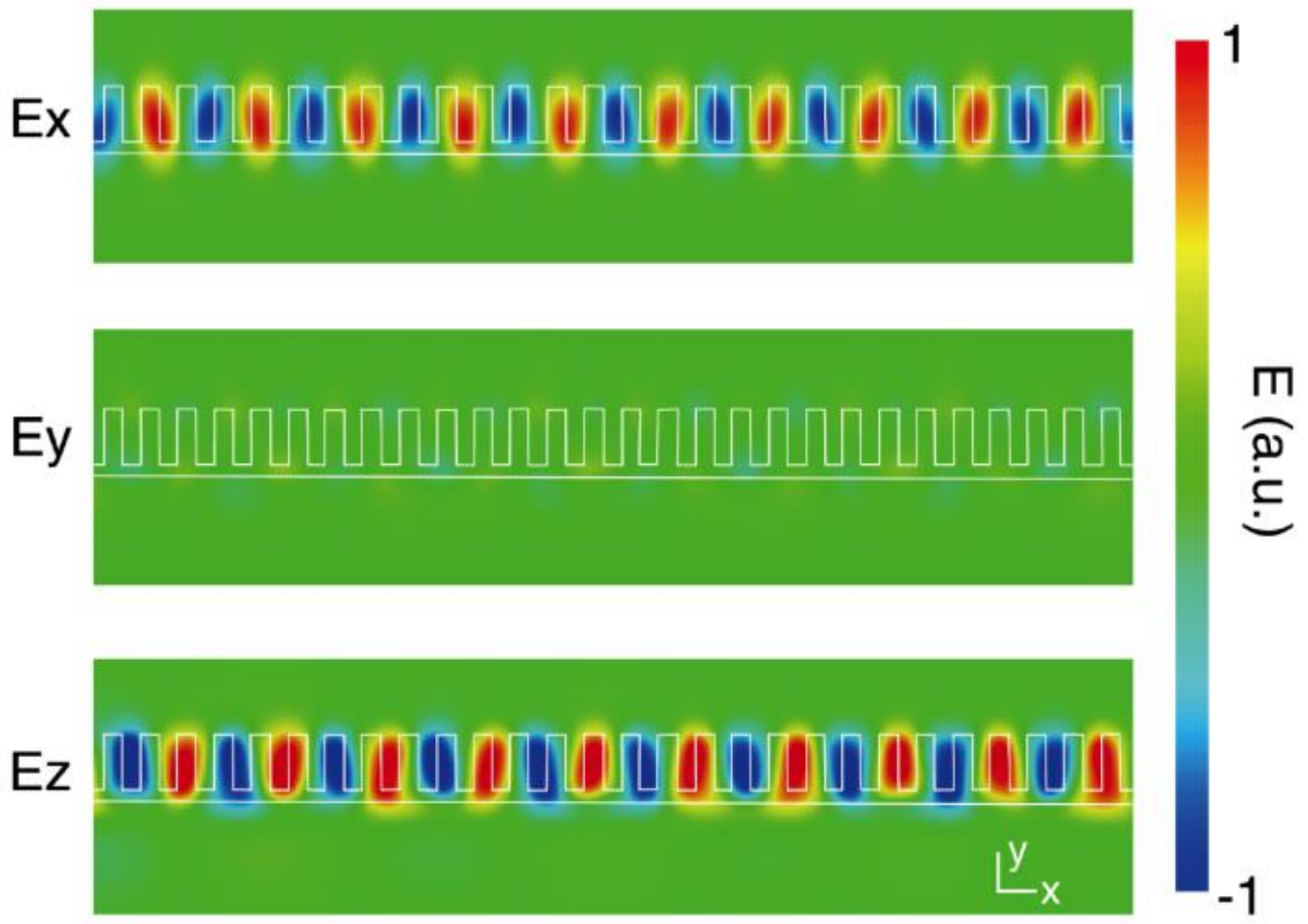

Fig. S5. Electric field of the mode in the xy plane at 2.4 GHz. Full-wave simulations of the electric field (xy plane) at 2.4 GHz for the SSP metamaterial textiles, the electric field expressions indicate that the SSP modes exhibit transverse magnetic characteristics and are highly confined near the structure.

This property is fundamentally different from that of conventional GHz-frequency guided structures, such as microstrip or coaxial waveguides, whose fields are largely confined and thus generally require dedicated coupler structures for efficient interaction with nearby wireless devices. By contrast, the surface plasmon-like mode supported by our textile exhibits a pronounced evanescent field outside the conductor, which enables practical near-field coupling with surrounding RF antennas. To clarify this point, we have revised the Section 2A as follows:

"The surface wave supported by the SSP waveguide exhibits a pronounced evanescent field extending beyond the conductor, thereby enabling practical near-field coupling with surrounding RF antennas (Supplementary Note B and Supplementary Fig.5). …"

Regarding the antenna/coupler details, on the smartphone side, one of the most widely used BLE antenna types is the inverted-F antenna (IFA). Its current distribution generally includes components that satisfy the above excitation condition, which explains why practical coupling between a commercial smartphone and the textile SSP waveguide can be achieved in our demonstrations. On the node side, to simplify the system design and demonstrate compatibility with existing wireless wearable HMI sensors, we directly used the antenna integrated into the BLE MCU. We agree that a purpose-designed antenna, with its source current predominantly oriented along the x or z direction, could further improve the coupling efficiency. However, such a design would increase system complexity and require additional modifications to existing wireless wearable HMI sensors, thereby reducing the compatibility of our approach. We have include a new description in the revised Section 2C of manuscript to clarify this point:

"Meanwhile, to simplify the system design, we directly used the antenna integrated into the BLE MCU to transmit the 2.4 GHz signals. In this configuration, the generated source excite surface waves on the textile SSP waveguide, which then guide the transmitted signals to a smartphone receiver."

The reviewer also correctly points out that the phone-position is essential in determining the surface wave excitation efficiency. This effect has been studied in previous studies [R1-R2]. And we have now included a new supplementary figure (Fig. S13) to illustrate the real phone position tolerance. The results show that when the lateral displacement is within 2 cm, the RSSI remains above −80 dB, indicating that stable wireless communication can be reliably maintained within this tolerable range. We have incorporated these results into the revised Section 2B of manuscript :

"To evaluate the system's spatial operating margin, we characterized the RSSI stability as a function of the distance between the smartphone and the metamaterial textile (Supplementary Fig. 13). The results demonstrate that a reliable communication link is maintained even as the device is moved away from the textile, highlighting the system's tolerance to practical, non-ideal positioning."

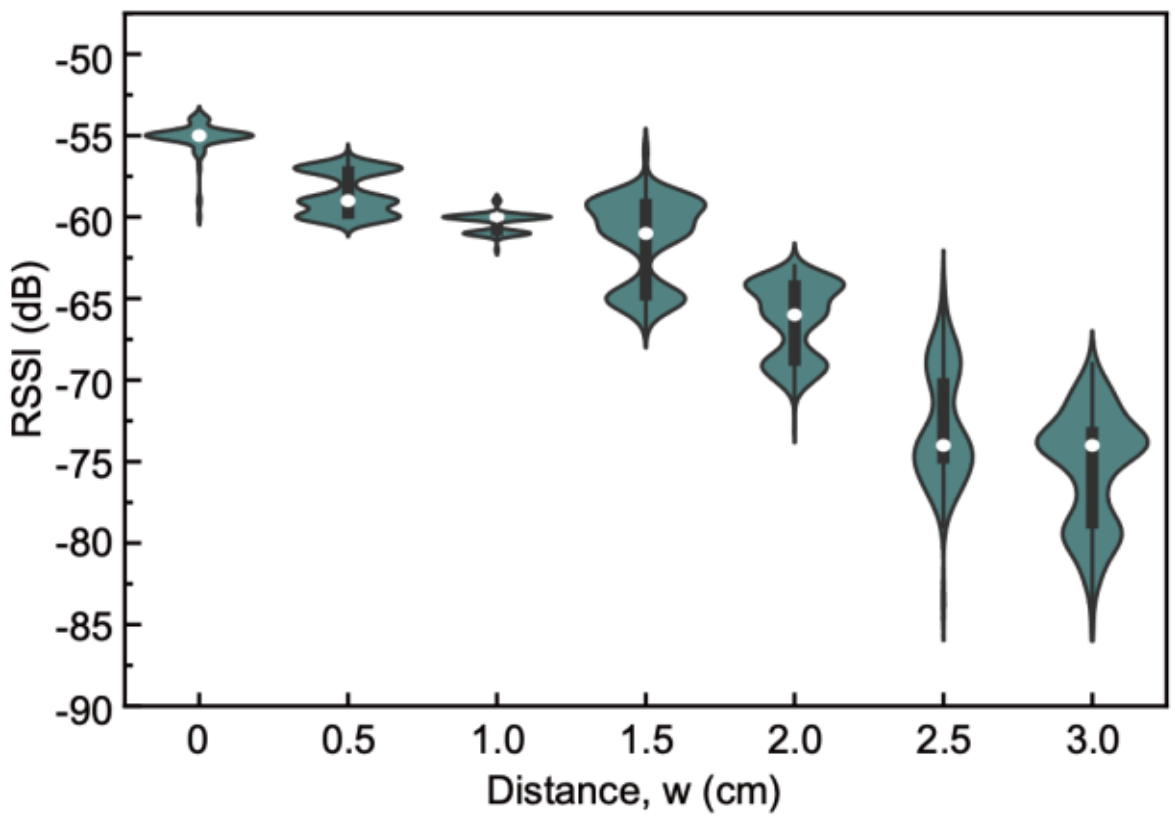

Fig. S13. Phone position tolerance of the metamaterial textile. The phone was placed on the SSP, and the RSSI of a single node was recorded for 1 min at each position as the phone was incrementally displaced perpendicular to the SSP structure (i.e., increasing w). The position defined as w = 0 corresponds to the location yielding the maximum RSSI, representing the optimal coupling condition.

[R1] Tian, Xi, et al. "Wireless body sensor networks based on metamaterial textiles." *Nature Electronics* 2.6 (2019): 243-251.

[R2] Tian, Xi, et al. "Conformal propagation and near-omnidirectional radiation with surface plasmonic clothing." *IEEE Transactions on Antennas and Propagation* 68.11 (2020): 7309-7319.

3. *Several practical claims are currently stronger than the data presented.*

The supplementary comparison table presents this work as machine-washable and highly user-friendly, and the main text also argues that surface-wave confinement improves security by reducing loss and interception risk. However, in the material provided, I could not find dedicated washing data such as post-wash power-transfer efficiency, RSSI, sensor performance, leakage, connector fatigue, or abrasion/sweat robustness. Likewise, the security argument is presently supported mainly by physical intuition and simulation rather than by direct leakage or eavesdropping measurements. These issues do not necessarily prohibit publication, but the authors should either provide supporting experiments or moderate the wording so that the paper remains clearly framed as a proof-of-concept systems study rather than a validated practical platform.

Reply: We thank the reviewer for this insightful and constructive comment. We fully agree that the claims regarding machine washability require dedicated experimental validation. We conducted washing tests in accordance with the ISO 6330:2012 protocols, which are widely adopted for assessing the impact of repeated laundering[R1]. In our tests, the textile was washed together with a heavy load of clothing (over 2.5 kg) to simulate harsh real-world conditions. After three consecutive wash cycles without any protective measures, the textile retained

comparable performance in both power transmission and data communication (Supplementary Table 2), confirming that it continues to meet functional requirements even after three repeated washing. We have also included a video (Supplementary Video 7) that illustrates the wash test process and demonstrates the textile's functionality after washing.

Supplementary Table 2. Washing test of the metamaterial textile.

Cycles	Transmission coefficient at 2.4 GHz (dB)	Power Efficiency at 13.56 MHz (%)	Rectified Voltage (v)	Duration (min)
0	-18.4	75.86	8.2	0
1	-18.9	74.01	8.0	45
2	-19.3	73.08	7.9	45
3	-20.1	72.16	7.2	45

We have included a new paragraph in the Methods section accordingly.

*"**Washing durability evaluation**. Washing tests were performed in accordance with the ISO 6330 standard for domestic laundering to evaluate the durability and functional integrity of the metamaterial textile over repeated wash cycles. The test specimen was placed in a household washing machine together with additional garments to achieve a total load exceeding 2 kg, while the specimen itself was maintained below 1 kg to ensure representative conditions. Each washing cycle lasted 45 minutes (Supplementary Video 7). Upon completion, the specimen was retrieved and air-dried under ambient conditions. Its electrical performance and transmission loss were subsequently characterized, confirming that the textile retained its functionality after three washing cycles (Supplementary Table 2)."*

Regarding the concern on system security, we would like to clarify that this aspect has been systematically investigated and experimentally validated in our prior work published in *Nature Electronics* (2019, 2(6), 243–251), where the metamaterial textile supporting spoof surface plasmon (SSP) mode was first proposed. In that study, comprehensive experiments were conducted to quantify the physical-layer security characteristics of the wireless body sensor network (WBSN) enabled by SSP textiles. Specifically, conventional radiative wireless transmission exhibits an eavesdropping range on the order of ~22 m, whereas the SSP-enabled system confines electromagnetic energy along the textile surface, resulting in a highly localized signal distribution with an effective propagation distance of ~10 cm from the body. In addition, prior measurements of wireless signal strength during Bluetooth interconnection between on-body sensor nodes demonstrated fundamentally different spatial decay behaviors. Without SSP textiles, the radiative signal is partially obstructed by the body and can even exhibit increased strength at short distances (up to ~20 cm), followed by gradual decay, with comparable signal levels observed even at distances up to several meters. In contrast, SSP-enabled transmission

shows a clear exponential attenuation with distance, with the signal rapidly falling below communication thresholds beyond ~10 cm while maintaining stable on-body connectivity. These results provide direct experimental evidence supporting reduced signal leakage and a significantly diminished eavesdropping range. Importantly, this form of physical-layer security complements conventional cryptographic and protocol-level approaches, as it inherently limits signal exposure without requiring additional computational overhead or hardware modification.

In response, we have revised the main text to explicitly cite this work and clarify the underlying rationale:

"... In contrast to conventional wireless protocols that radiate energy omnidirectionally into free space, leading to significant energy loss and potential interception, the SSP structure localizes electromagnetic propagation within close proximity to the body, thereby improving both transmission efficiency and communication security[21]. ..."

[R1] S. Rotzler, von M. Krshiwoblozki and M. Schneider-Ramelow. Washability of e-textiles: current testing practices and the need for standardization. Text. Res. J. 91, 2401-2417 (2021).

4. *Reproducibility and reporting transparency need improvement.*

The Methods are good for an interdisciplinary systems paper: the manuscript gives textile geometry, substrate dielectric constant, embroidery machine, sensor-node ICs, and fabrication routes for the strain and pressure sensors. Still, full reproduction would remain difficult because the paper does not provide embroidery pattern files/CAD, Gerbers, matching-network details, firmware, BLE configuration, phone-side software, or ML code and preprocessing. The Data Availability statement is limited to data being available on reasonable request. For a paper whose contribution depends heavily on integrated RF/electronics/textile/ML implementation, I think the transparency bar should be higher.

Reply: We thank the reviewer for this thoughtful comment regarding reproducibility and reporting transparency. We fully agree that clear and sufficient methodological disclosure is essential, particularly for interdisciplinary system-level studies. At the same time, we would like to respectfully clarify the primary scope and contribution of this work. The central novelty lies in the proposed architecture of a battery-free wireless body sensor network (WBSN) enabled by the dual-mode metamaterial textile, and the concept of unencumbered HMI interaction. These aspects constitute the core scientific and engineering contributions of the manuscript, which are not limited to the integration of discrete components.

In contrast, components such as firmware implementation, BLE configuration, mobile-side software, and machine learning pipelines are primarily employed to demonstrate system-level

feasibility and application potential. They can be implemented using standard, widely accessible approaches in the field. As such, we believe that providing exhaustive implementation-level resources is not strictly necessary for reproducing the key innovations and validating the main conclusions of this study. Importantly, the essential information required to reproduce the core contributions (particularly the design parameters, material composition, and fabrication details of the dual-mode metamaterial textile) has already been provided in the manuscript and supplementary materials.

Nevertheless, in response to the reviewer's suggestion and to further improve transparency, we have taken additional steps to improve transparency and reproducibility. Specifically, we have revised Supplementary Fig. 2 to include detailed structural parameters of the dual-mode metamaterial textile (including coil and SSP waveguide geometries), from which the embroidery pattern can be directly reconstructed.

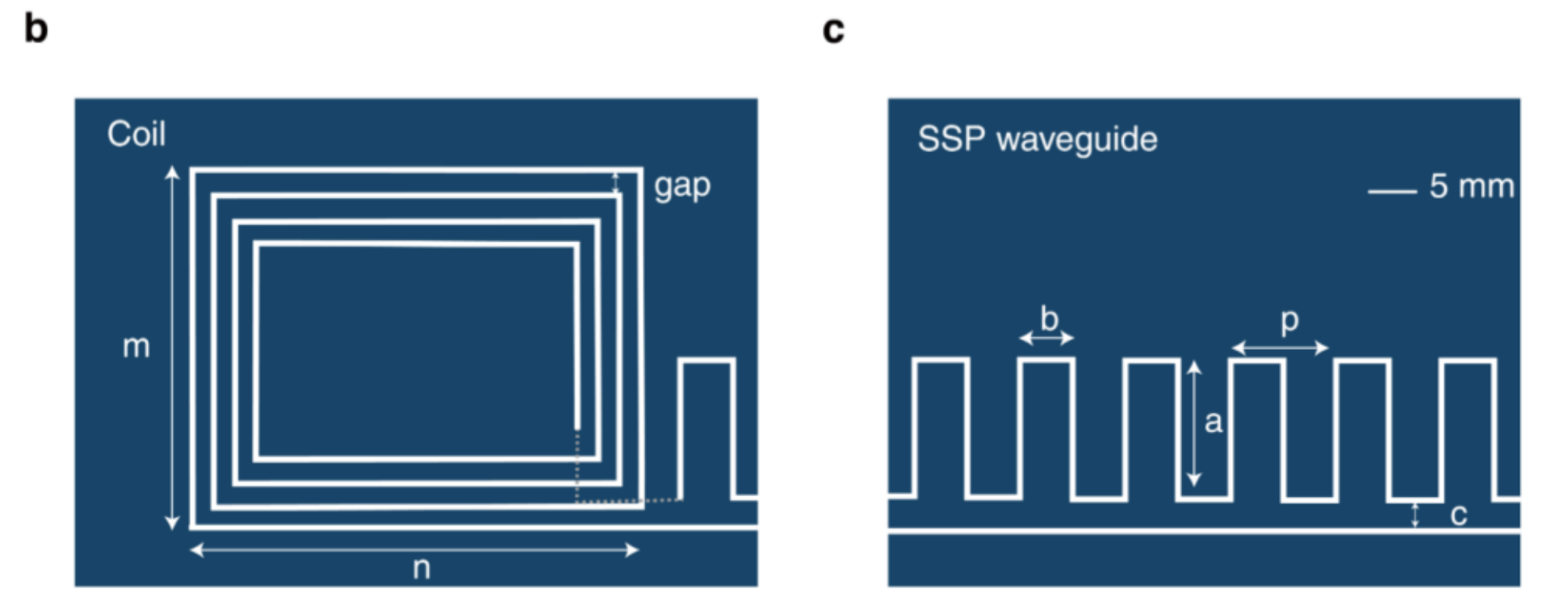

Fig. S2. (b,c) Structure of dual-mode metamaterial textiles including coils (m = 40 mm, n = 30 mm, gap = 1.6 mm) and SSP waveguides (a = 14 mm, b = 6 mm, c = 2 mm, p = 12 mm) for powering supply and data transmission.

We have also added a new supplementary figure (Fig. S42) that provides the detailed circuit schematic and bill of materials (BOM) for the sensor node.

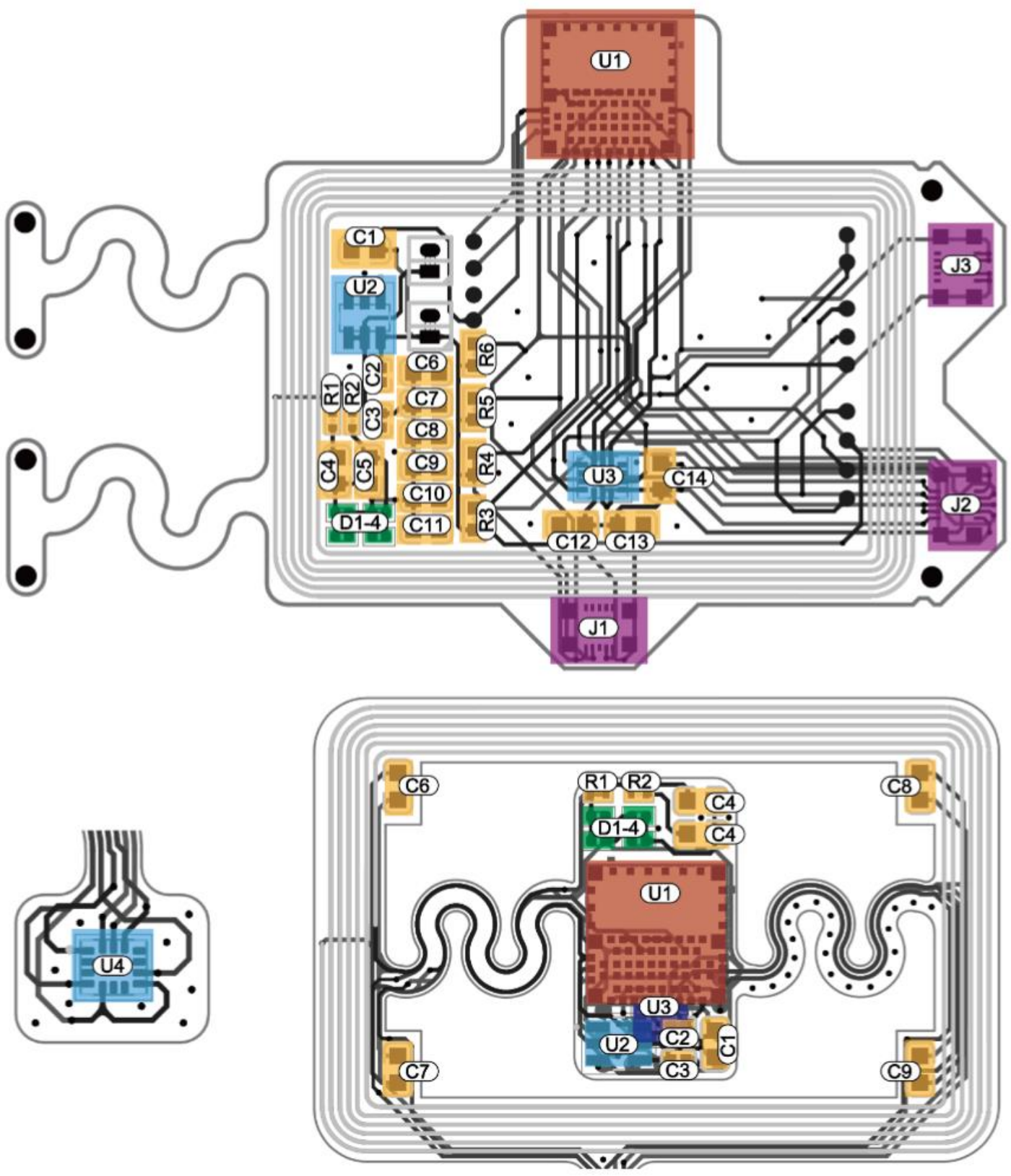

U1	ISP1807	C1, C12	100nF (0603)
U2	AP7312-1833	C2, C3	1uF (0402)
U3, U4	LSM6DSOTR	C4, C5	100pF (0603)
D1-4	CDBQC0130L-HF	C6-C11	22uF (0603)
J1-3	X0400FVS-10-LPV01	C13	2.2uF (0603)
R1, R2	0R (0402)	C14	10nF (0603)
R3-5	100K (0603)		

Fig. S42. Sensor nodes schematic. Battery-free sensory glove and epidermal sensor node circuitry with components identified.

In addition, we have made the machine learning and data processing code publicly available via an open-access GitHub repository, and updated the Data and Code Availability statement accordingly. These additions collectively enhance the reproducibility of the work while maintaining focus on the core scientific and engineering contributions.

DATA AVAILABILITY

"Source data are provided with this paper. The other data that support the findings of this study are available from the corresponding authors upon request. "

CODE AVAILABILITY

"The code used in this study is available via GitHub at https://github.com/thu-wbg-zym/WBSN-project."

***5.** There are several reporting inconsistencies that should be corrected before publication.*

In Fig. 2b, h is defined as the vertical distance between the transmitter and receiver. However, in the main Results text, the measurement condition for Fig. 2c, where the relay length L is varied, is described as h = 5 cm, w = 0 cm. In contrast, the caption of Fig. 2 gives the condition for the same Fig. 2c as h = 0.5 cm, w = 0 cm. Moreover, the same section states that the efficiency drops to zero when h > 26 mm, which is not consistent with the high efficiency shown in Fig. 2c if h = 5 cm were correct. Thus, there is at least one typo or description error somewhere in this part.

A further point of confusion is that the lower panel of Fig. 2e on page 28 appears to indicate "h = 5 mm." However, this refers to the fixed condition for a different panel, namely the plot showing the dependence on w. The issue here is specifically the discrepancy between the main text and the caption regarding the relay-length experiment in Fig. 2c, and is separate from the 5 mm label in Fig. 2e.

Reply: We thank the reviewer for the very careful reading of the manuscript and for identifying these inconsistencies. Upon careful verification, we confirm that the value "h = 5 cm" stated in the main Results text is a typographical error. The correct experimental condition is h = 5 mm, w = 0 cm, which is consistent with the description provided in the Fig. 2 caption. This correction also resolves the apparent contradiction noted by the reviewer. Specifically, the high transmission efficiency observed in Fig. 2c is physically consistent with a separation of h = 5 mm, and aligns with the statement that the efficiency rapidly decays to zero when h > 26 mm. We also note that the "h=5 mm" label in Fig. 2e refers to a separate measurement condition and is correct as originally presented.

We have corrected this in the main text as follows:

"The power transfer efficiency η was measured as a function of relay length (L) under fixed geometric parameters (h = 0.5 cm, w = 0 cm)(Fig. 2c)."

The second issue is an incorrect reference to the supplementary figures. In the Results text for the VR handwriting recognition experiment, the CNN classification results are said to correspond to Fig. 4f and Supplementary Fig. 32. However, the caption of Fig. 4 states that the complete results are shown in Supplementary Fig. 33. In the actual Supplementary Information, Fig. S32 is the confusion matrix for 47-character recognition, whereas Fig. S33 shows the full-body WBSN architecture. Therefore, this seems to be not a discrepancy in the experimental results themselves, but rather a figure numbering error, most likely an off-by-one mistake.

Reply: We thank the reviewer for pointing out the indexing inconsistency of original Supplementary Fig. 32 . We confirm that this was a clerical error in figure referencing. We have corrected the relevant citation in the Fig. 4 caption to refer to new Supplementary Fig. 35. In addition, we have conducted a comprehensive cross-check of all figure and table references in both the main text and Supplementary Information to ensure consistency throughout.

"*... Complete results are provided in Supplementary Fig. 35. ...*"

The third issue is that the claim of ">100-fold improvement" does not appear to align directly with the numbers shown in the table. The Abstract states that the communication throughput is improved by "over two orders of magnitude," and the Introduction similarly says ">100 times." However, in Supplementary Table I, the data rate of this work is listed as up to 1 Mbps, while the clothing based near-field coupling system used for comparison is listed as 26.48 kbps. This corresponds to a ratio of only about 37.8x, which would naturally be described as tens-fold, not >100-fold. Please clarify the baseline used for this claim and revise the wording so that it is quantitatively consistent.

Reply: We thank the reviewer for this important observation regarding the performance improvement metrics. The previously stated ">100-fold improvement" refers to the enhancement in transmission efficiency (i.e., transmission coefficient) when comparing surface-wave-guided propagation enabled by the metamaterial textile with conventional radiative transmission. We agree that this statement could be confused with the data throughput comparison presented in Supplementary Table I. For the comparison between the proposed dual-mode metamaterial textile and near-field clothing systems, the improvement in data throughput is approximately 37.8 times, as correctly noted by the reviewer.

To avoid ambiguity and ensure quantitative consistency, we have revised the descriptions in the Abstract, Introduction, and Conclusion accordingly:

Abstract:

"This architecture enhances data throughput by over an order of magnitude and doubles wireless power transfer efficiency compared with emerging near-field clothing."

Introduction:

"We fabricate the metamaterial textiles using computer-aided digital embroidery of liquid metal and show that they enhance wireless data throughput by over one order (37.8 times) while simultaneously doubling power-transfer efficiency compared with emerging near-field clothing[19,20,24]"

Conclusion:

"We demonstrated that the dual-mode metamaterial design supports simultaneous high-efficiency data transmission and wireless power transfer, achieving more than an order of magnitude enhancement in data throughput and a twofold improvement in power transfer efficiency compared with emerging near-field clothing."

Minor comments

1. *The manuscript would benefit from clearer separation between simulation results, benchtop characterization, and real on-body experiments, especially in places where the text may currently encourage the reader to overinterpret simulated robustness as experimental proof.*

Reply: We thank the reviewer for this insightful observation. We agree that maintaining a rigorous distinction between idealized simulations, controlled benchtop characterizations, and dynamic on-body experiments is essential to avoid overinterpretation. In response, we have systematically revised the manuscript to explicitly delineate these different data sources throughout the text. Simulation results, benchtop measurements, and on-body experiments are now clearly labeled and discussed in their respective contexts. In addition, we have incorporated new experimentally measured data in Fig. 2i–j and Fig. 2m to provide direct comparison with the corresponding simulation results, thereby strengthening the experimental validation and ensuring a more balanced interpretation.

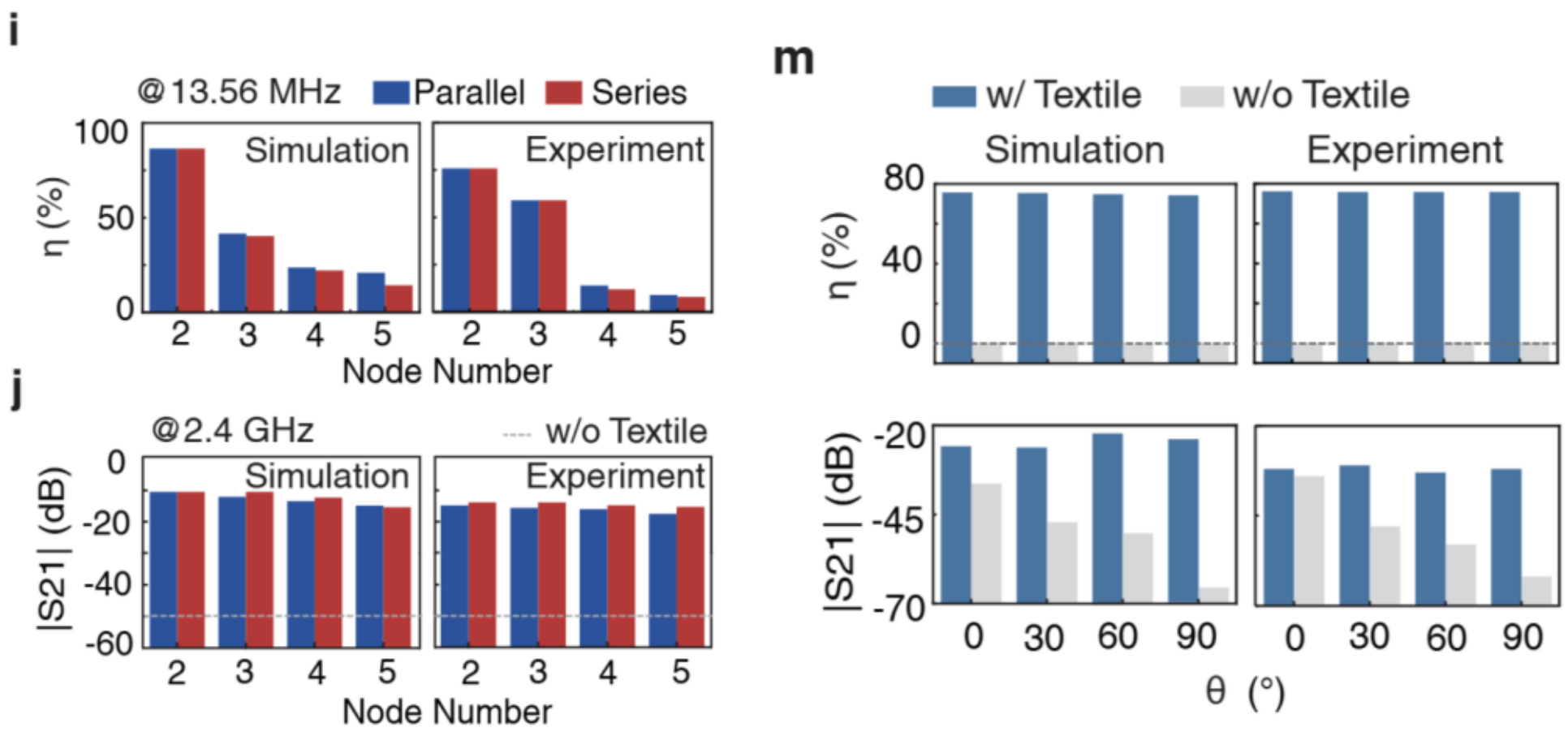

Fig. 2i,j, Simulated and experimental results of power transfer efficiency (i) and transmission coefficient (j) as a functions of node number under parallel and series configurations. **m,** Simulated and on-body measured power transfer efficiency and transmission coefficient as functions of arm-raising angle, with and without the metamaterial textile, demonstrating angular stability and robustness to body movement.

***2.** For the ML sections, please report participant numbers, number of trials per class, data balancing, cross-subject validation protocol, and uncertainty/statistical variation across runs.*

Reply:

We thank the reviewer for this valuable suggestion regarding the reporting of machine learning details. In the action classification task, data were collected from three participants, with 100 valid samples acquired for each action class. The dataset is balanced across classes. All samples were randomly partitioned into training and test sets using a 4:1 split. The test set contains mixed data from all three participants, thereby providing an initial assessment of cross-subject generalization. The model achieved overall classification accuracies of 99.2%, 99.3%, and 100% in the three rounds on the test set, respectively.

Following the reviewer's suggestion, we have now clarified these details in the revised manuscript.

"... The open-source dataset EMNIST[32] was used for training and validating the model, which includes numbers, uppercase letters, and lowercase letters. ..."

"... We collected 30, 34, and 36 sets of data for each action from three subjects (100 samples per class in total). The dataset was divided into training and test sets with a 4:1 ratio. The test set contained samples from all participants, providing an initial evaluation under mixed-subject conditions. The trained LSTM model achieved an average recognition accuracy of 99.5% in three rounds of test set validation. ..."

Reviewer #2

This study proposes a dual-mode metamaterial network for distributed body sensor systems. It provides a significant contribution to the field of wearable metamaterial clothing by enabling both power transfer and data communication through its design. While the demonstration of a single-hand glove alone might not be entirely unique, the whole-body batteryless sensor network provides a clear advantage to this system. However, I have a few minor concerns related to the clarification and logical support for some arguments included in this paper, though these issues do not limit the overall value of this work.

Reply: We sincerely thank the reviewer for the positive assessment and for recognizing the significance our work. We particularly appreciate the acknowledgment of the advantages offered by our whole-body, battery-free sensor system. Following the reviewer's constructive feedback, we have carefully revised the manuscript to strengthen the logical support and improve the clarity of the arguments mentioned. Detailed responses to each specific point are delineated below.

1. *At the top of page 8, the phrase "wireless transmission speed" is ambiguous since the transmission speed in RF refers to the speed of light. A more accurate term would be "wireless data transmission speed".*

Reply: We thank the reviewer for pointing out this ambiguity. To distinguish the data throughput from the propagation speed of electromagnetic waves, we have revised the phrase to "wireless data transmission speed" as suggested:

"*... Further measurement of wireless data transmission speed demonstrates that ...*"

2. *The statement "the received BLE signal strength remains above -50 dB" contains an incorrect unit. It should be expressed in dBm rather than dB.*

Reply: We thank the reviewer for pointing out this imprecise wording. Here, we intended to describe the transmission coefficient in the 2.4–2.5 GHz range, rather than the received BLE signal power. We agree that the original wording could be misleading because the unit dB refers to the transmission coefficient, whereas dBm would be appropriate for received power. We have therefore revised the description in Section 2B of the manuscript as follows:

"*... the transmission coefficient remains above -50 dB ...*"

3. *Regarding the sentence, "Meanwhile, the 2.4 GHz transmission coefficient exceeds -20 dB, indicating that the BLE signal strength is enhanced by more than three orders of magnitude," I recommend rephrasing this to clarify that this is a comparison of efficiency (metamaterial enhanced vs. radiation). Additionally, claiming an enhancement of three orders of magnitude seems rather arbitrary and is accurate only under specific conditions. As seen in Figure 2m, the efficiency S21 with the textile is not universally three orders of magnitude higher than without it.*

Reply: We thank the reviewer for raising this important point. We agree that the reported enhancement should be explicitly framed as a comparison of transmission efficiency between surface-wave-guided propagation enabled by the metamaterial textile and conventional radiative transmission. Accordingly, we have revised the statement in the Section 2B of main text to:

"Meanwhile, the transmission coefficient at 2.4 GHz remains consistently above -20 dB. This represents a more than three-order-of-magnitude enhancement in received signal strength compared to conventional radiative propagation. Such a substantial improvement provides experimental validation of the field confinement effect, demonstrating the system's ability to bypass the high path loss typically associated with body-centric wireless links."

Second, we acknowledge that the previously stated "more than three orders of magnitude" enhancement is condition-dependent and not universally applicable across all configurations. To address this, we have revised the manuscript to (i) explicitly specify the geometric conditions under which such enhancement is observed, and (ii) provide a more nuanced interpretation of the transmission behavior in Fig. 2m:

"As the bending angle increases, the transmission coefficient without the textile (radiative propagation) decreases markedly due to increased path loss, whereas the surface-wave-guided transmission remains relatively stable. At a bending angle of 90°, the difference exceeds 30 dB, corresponding to an improvement of over three orders of magnitude in transmission efficiency under this condition. "

4. *Figure 2f is somewhat confusing. Although a legend is provided, the gray area- which presumably represents the efficiency level without the textile- is not clearly defined in the figure or the caption.*

Reply: We thank the reviewer for this careful observation. In the original figure, the gray shaded region was intended to represent the baseline transmission level measured without the metamaterial textile, which was labeled as "w/o Textile". To improve clarity, we have revised the caption of Fig. 2f to explicitly state that the gray region denotes the reference $|S_{21}|$ range

obtained under the "without textile" condition across the 2.4–2.5 GHz band. This revision improves the clarity and interpretability of the figure.

"**FIG. 2. *f***, *Violin plots comparing benchtop-measured transmission coefficients* $|S_{21}|$ *under various w and h configurations, with and without the metamaterial textile, across the 2.4–2.5 GHz ISM band. Box plots within the violins indicate the quartiles of the corresponding spectra. The gray shaded region represents the baseline* $|S_{21}|$ *range for the condition without textile.* "

5. *"These results confirm that the power delivered through the metamaterial network is sufficient to sustain continuous sensing and communication without noticeable fluctuation or packet loss." The authors need to clarify how they reached this conclusion. It appears the authors concluded this because no packet retransmission occurred on the Bluetooth device and the 20 ms interval was accurately maintained. Adding this explanation would better guide the readers. A stronger statement would include the average transmission interval ± standard error.*

Reply: We thank the reviewer for raising this point for clarification. The conclusion was based on the stable Bluetooth communication performance, specifically the absence of packet retransmission events and the consistent maintenance of the transmission interval throughout the measurement. Following the reviewer's suggestion, we have included a quantitative characterization of the transmission stability by reporting the average transmission interval with its standard error (20 ± 1.13 ms) and revised the manuscript to explicitly state these criteria:

"The stable communication behavior, evidenced by the absence of packet retransmissions and negligible variation in the transmission interval (20 ± 1.13 ms), provides strong evidence that the power delivered through the metamaterial network is sufficient to sustain continuous sensing and communication. ..."

6. *On page 13, the authors mention that the device is lightweight, but no specific weight values (numbers) are provided.*

Reply: We thank the reviewer for this valuable suggestion. Providing quantitative weight information indeed better substantiates the lightweight advantage of the proposed battery-free system. We have revised the manuscript to include specific weight values for each wearable device, thereby offering a clearer and more objective evaluation of their form factor:

Section 2C: "... The main control board of this battery-free sensory glove weighs only 0.836 g, while the complete battery-free sensory glove weighs only 13.832 g. ..."

Section 2F: "... A complete epidermal sensor node weighs only 1.048 g. ..."

7. *"During high intensity motion such as running, minor voltage fluctuations were observed due to transient coil misalignment; however, the regulated outputs of all nodes maintained sufficient margins to ensure uninterrupted operation." While Figure 5e provides data on voltage and RSSI fluctuations during these activities, the claim of uninterrupted operation is not fully supported by these metrics alone. It is highly recommended to include data on data transmission packet loss (%) and latency during high-intensity motions to support this argument.*

Reply: We thank the reviewer for this constructive suggestion. The transmission packet loss for all three nodes remains 0% throughout the entire testing period, which is clarified in the main text. In addition, we have included new quantitative results in Supplementary Fig. 38 to directly evaluate wireless communication performance under high-intensity motion. The measured communication latency for each node consistently remains below 20 ms during running. These results provide direct quantitative evidence that, despite transient fluctuations induced by coil misalignment, the system sustains stable and uninterrupted data transmission under dynamic conditions. The manuscript has been revised accordingly to include these results.

"Quantitative analysis of transmission latency during high-intensity motion (Supplementary Fig. 38) further shows that the delay remains consistently below 20 ms across all nodes, supporting reliable real-time data transfer under dynamic conditions."

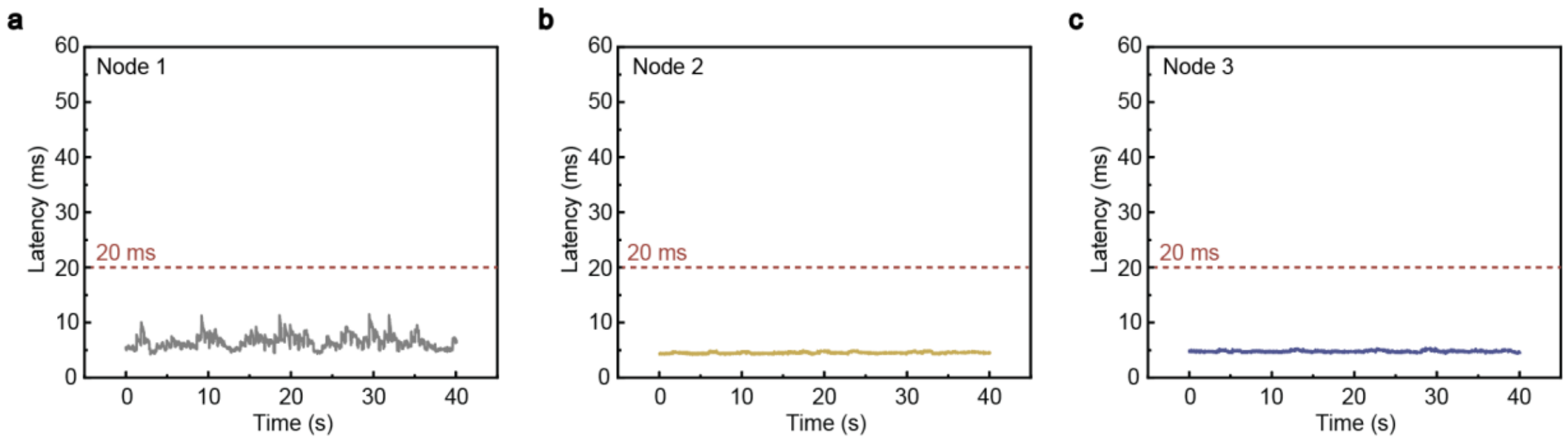

Fig. S38. Transmission latency during high-intensity motion. The communication latency of three nodes was measured during the subject's running activity. Node 1 corresponds to the battery-free glove, while Nodes 2 and 3 are battery-free epidermal sensor nodes. Throughout the motion, the data transmission latency of all nodes remains below 20 ms, indicating stable wireless communication under dynamic conditions.

8. *"A game-based evaluation task was designed to assess the responsiveness and accuracy of the WBSN." This argument is not supported by the results. The responsiveness and accuracy of the WBSN are not demonstrated in this task (only angular data is provided). The authors should either rephrase this sentence or provide the supporting results.*

Reply: We thank the reviewer for this insightful suggestion. We agree that this task was intended to demonstrate the practical functionality and real-time application of the WBSN rather than to provide a quantitative performance validation. To clarify this point, we have rephrased the sentence in the Section 2F of manuscript:

"*... A game-based evaluation task was designed to demonstrate the functional utility and real-time tracking capabilities of the WBSN in a dynamic application context. ...*"

Reviewer #3

The manuscript reports a battery-free wireless body sensor network based on dual-mode metamaterial textiles for continuous and unobtrusive human machine interaction. By digitally embroidering liquid-metal fibers into conductive textile patterns, the authors construct ultra-wideband electromagnetic pathways that simultaneously support wireless power transfer (13.56 MHz) and wireless communication (2.4 GHz), enabling distributed battery-free sensors to operate across the full body. The system integrates near-field power delivery with low-latency wireless data transmission and demonstrates applications in gesture recognition, robotic control, virtual/augmented reality interaction, and human digital twins. Overall, the manuscript presents a well-designed system with convincing demonstrations. The work is relevant to the development of wearable electronics and next generation human–machine interfaces. Moreover, the manuscript presents a relatively complete system-level demonstration, and overall the work is well organized and clearly presented. However, the manuscript could be further strengthened by addressing the following points for improved technical clarity and rigor:

Reply: We sincerely thank the reviewer for the positive and constructive assessment of our work. We particularly appreciate the reviewer's recognition of the system-level innovation, the clarity of the manuscript's organization, and its high relevance to the development of next-generation human-machine interfaces. Following the reviewer's insightful suggestions, we have carefully revised the manuscript to enhance its technical clarity and rigor. Detailed responses to the specific points raised are provided below.

1. *Regarding the operating principle, the dual-mode scheme integrating 13.56 MHz inductive coupling and 2.4 GHz surface-wave guiding is interesting. However, a clearer explanation of how the SSP structure simultaneously supports these two widely separated frequency regimes without mutual interference would strengthen the manuscript. In addition, the claims regarding higher efficiency and enhanced security appear largely qualitative and would benefit from clearer quantitative justification.*

Reply: We thank the reviewer for this valuable comment. We would like to clarify that, because Maxwell's equations are linear, time-harmonic fields at different frequencies are orthogonal, meaning that electromagnetic waves at distinct frequencies propagate independently in linear media. In conventional waveguides, mutual interference is generally a concern when two modes operate at the same or closely spaced frequencies and possess similar propagation constants. In the present case, however, the two modes are widely separated in frequency. Consequently, the proposed SSP structure can simultaneously support these two

widely separated frequency regimes without mutual interference. To make this point clearer, we have revised the main text as follows:

"In the powering mode, ... This effectively extends the smartphone's wireless powering range to distant sensor nodes. Specifically, the 13.56 MHz quasi-static magnetic field generated by the NFC coil (Supplementary Fig. 4) does not excite propagating waves along the SSP structure due to the extremely long wavelength at this frequency. Consequently, the near-field energy transfer remains confined to the coil region without coupling into the SSP waveguide.

In the communication mode, ... Because time-harmonic fields at different frequencies are orthogonal in a linear system, the proposed SSP structure can simultaneously support these two widely separated frequency regimes without mutual interference. ......"

We also thank the reviewer for highlighting the need for quantitative support regarding efficiency and security. In the original manuscript, Fig. 2 provides detailed quantitative results, including both simulations and experimental measurements, to substantiate the high efficiency of the proposed metamaterial textile. Regarding communication security, this aspect has been systematically investigated and experimentally validated in our prior work (*Nature Electronics* 2019, 2(6), 243–251), where SSP-based metamaterial textiles were first introduced. In response, we have revised the main text to explicitly cite this work and clarify the underlying rationale:

"... In contrast to conventional wireless protocols that radiate energy omnidirectionally into free space, leading to significant energy loss and potential interception, the SSP structure localizes electromagnetic propagation within close proximity to the body, thereby improving both transmission efficiency and communication security[21]. ..."

***2.** The manuscript presents electromagnetic field simulations to explain energy distribution across multiple nodes. However, the manuscript presents electromagnetic simulations to illustrate energy distribution across multiple nodes. However, the connection between the simulated field confinement and the experimentally measured transmission performance could be discussed more explicitly.*

Reply: We thank the reviewer for this insightful comment. We agree that the connection between the simulated field confinement and the experimentally measured transmission performance could be stated more explicitly. As shown in Fig. 2h, the simulated field confinement indicates that electromagnetic energy is strongly localized and guided along the SSP structure, which suppresses radiation leakage and promotes efficient signal delivery to distributed nodes. This behavior is consistent with the experimentally measured transmission performance in the multi-node configuration shown in Fig. 2j. We have revised the manuscript as follows:

"... The simulated field confinement (Fig. 2h) indicates that the electromagnetic energy is strongly localized within the evanescent region of the SSP structure. By suppressing radiation into the far-field, this guided-wave mechanism ensures efficient power delivery across the distributed network. This physical behavior is consistent with the experimentally measured transmission performance in the multi-node configuration (Fig. 2j)."

"... Meanwhile, the transmission coefficient at 2.4 GHz remains consistently above -20 dB. This represents a more than three-order-of-magnitude enhancement in received signal strength compared to conventional radiative propagation. Such a substantial improvement provides experimental validation of the field confinement effect, demonstrating the system's ability to bypass the high path loss typically associated with body-centric wireless links."

We have also revised Fig. 2i-j and Fig. 2m to include the comparison of simulated and experimentally measured results:

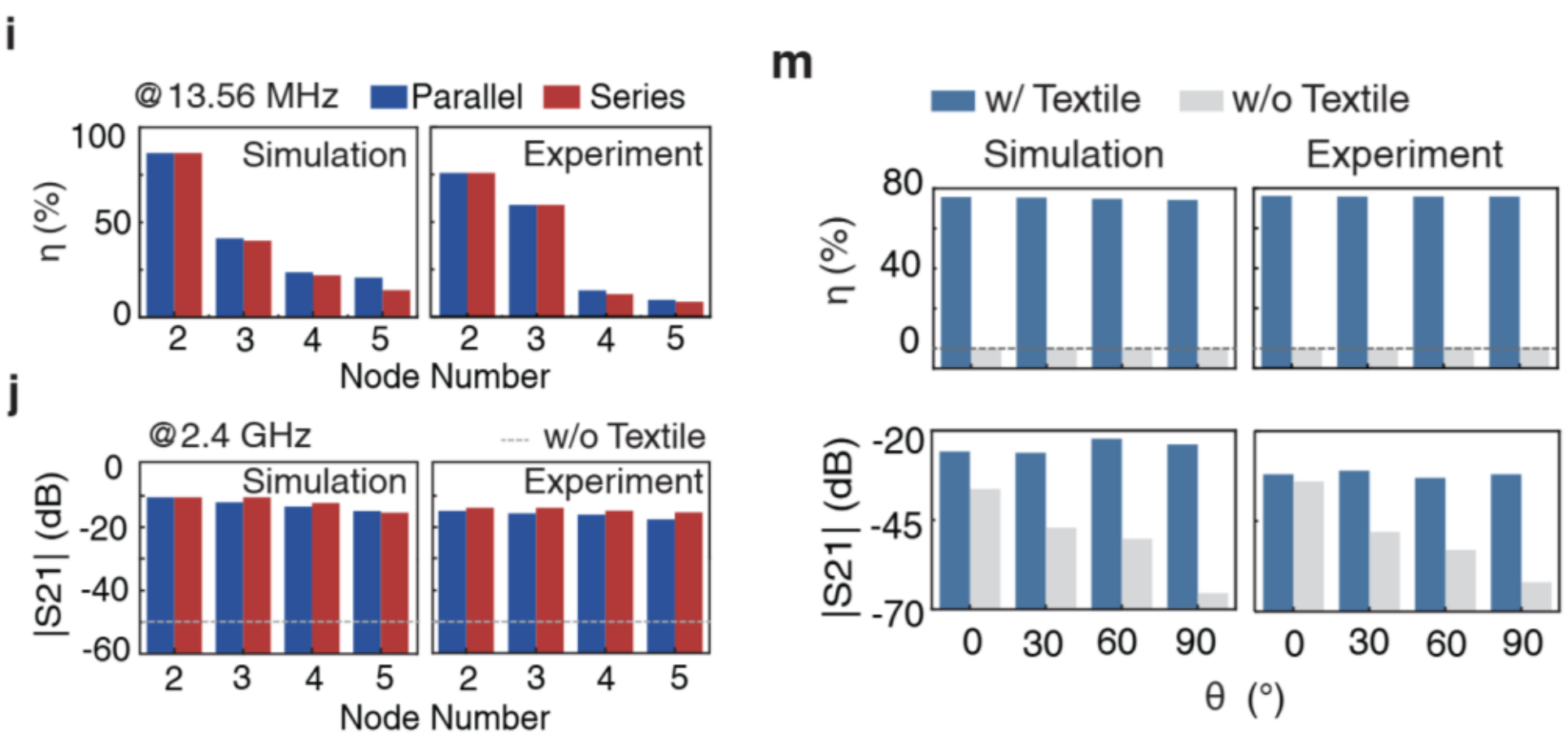

Fig. 2i,j, Simulated and experimental results of power transfer efficiency (i) and transmission coefficient (j) as a functions of node number under parallel and series configurations. **m,** Simulated and on-body measured power transfer efficiency and transmission coefficient as functions of arm-raising angle, with and without the metamaterial textile, demonstrating angular stability and robustness to body movement.

3. *The system architecture is clearly illustrated. However, a more explicit discussion of the relationship between the harvested power and the circuit power consumption would help clarify the energy budget for battery-free operation. In addition, the claim of continuous low-power sensing and stable data communication would benefit from a clearer discussion of long-term operational stability.*

Reply: We thank the reviewer for the constructive suggestion. To clarify the energy budget for battery-free operation, we have added an explicit discussion in the Methods section describing the relationship between harvested power and circuit power consumption:

Design of battery-free sensor node*: "... The NFC output power of commercial smartphones is governed by international standards, typically ranging between 100 mW and 300 mW (ISO/IEC 14443-2:2020). To ensure a robust design, we adopted the lower bound of this range (i.e., 100 mW) as our primary consideration. Given that the power transfer efficiency of the metamaterial textile under three-node loading is approximately 20%, the harvested power at each node is approximately 20 mW. Accounting for potential efficiency drops due to coil misalignment during high intensity motions, we optimized our sensor nodes to operate at a significantly lower power profile (i.e., <10 mW). This ensures a substantial "power buffer", maintaining continuous operation even under suboptimal coupling conditions. "*

To further substantiate the long-term operational stability of the system, we have conducted a 6-hour continuous operation test using a smartphone-powered configuration (Supplementary Fig. 33). During this period, the RSSI of a sensor node was continuously monitored to evaluate the stability of both sensing and wireless communication. In parallel, the smartphone battery level was recorded to provide a practical indication of the overall energy consumption associated with sustained operation.

The results show that the RSSI remains within a stable and comparable range throughout the entire 6-hour period, indicating reliable sensing and communication performance. Meanwhile, the smartphone battery level decreased from 100% to 53%, reflecting the combined power consumption of NFC-based wireless powering, screen usage, and background processes. Based on this measured consumption profile, the system demonstrates a projected continuous operation time of approximately 12 hours under comparable conditions. These results provide direct evidence of both stable long-term operation and a sustainable energy budget for battery-free sensing. The Section 2C of manuscript has been revised accordingly to include these results:

"... The long-term operational stability enabled by this energy budget is further validated through an extended continuous operation test (Supplementary Fig.33)"

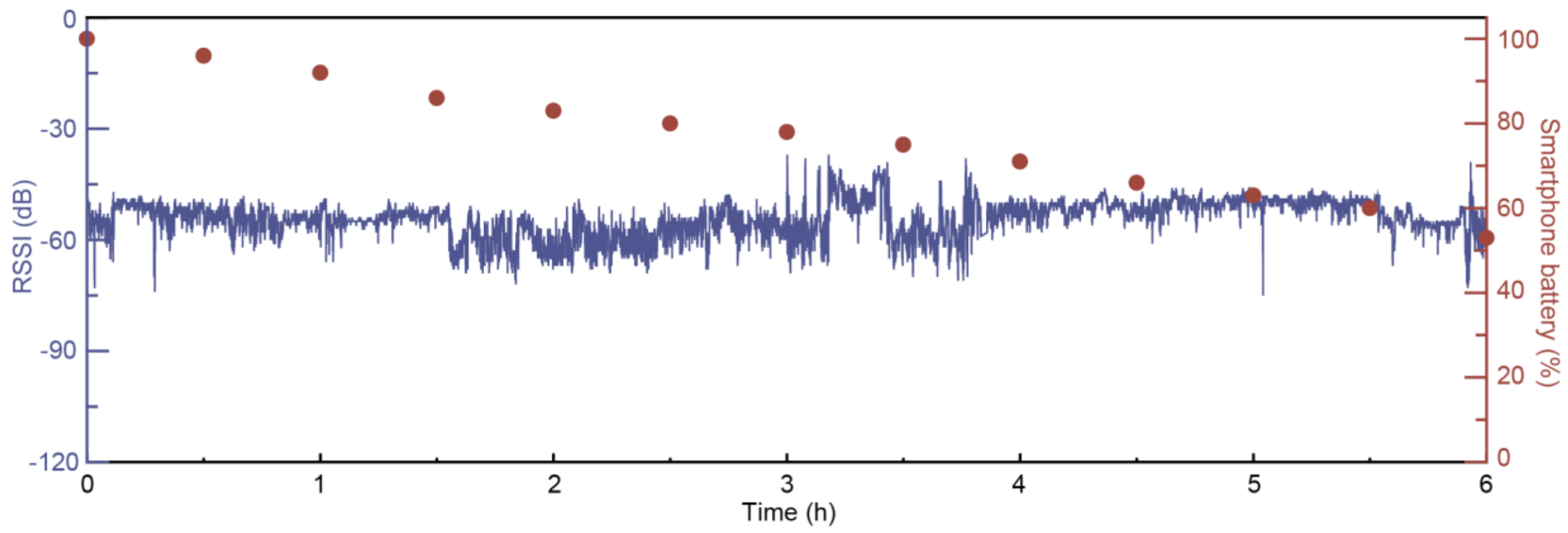

Fig. S33. Long-term continuous operation of the smartphone-powered WBSN system. The RSSI of a sensor node remained within a stable range throughout the 6-hour continuous operation, demonstrating consistent sensing and communication performance. Over the same period, the smartphone battery level decreased from 100% to 53%, accounting for power consumption from wireless powering (NFC), display activity, and background processes. This consumption profile suggests a feasible continuous operation duration of approximately 12 hours under similar conditions.

***4.** The human-robot interaction demonstration is interesting. However, the manuscript could more clearly highlight how the proposed metamaterial enabled WBSN improves system-level performance compared with conventional wireless wearable control approaches.*

Reply: We thank the reviewer for this insightful suggestion. In the original manuscript, Fig. 1a-c compare the proposed metamaterial-enabled wireless HMI with traditional wired and conventional wireless systems to illustrate its overall concept and qualitative advantages. We agree that the system-level performance improvements over conventional wireless wearable approaches could be more explicitly articulated.

In response, we have revised the main text to clearly highlight the key system-level advantages of the proposed approach, including power delivery, scalability, communication efficiency, data privacy, and wearable comfort. The following statement has been added:

"This metamaterial-enabled HMI system provides simultaneous advantages in power delivery efficiency, network scalability, communication robustness, data privacy, and wearable comfort (Supplementary Note A)."

Furthermore, we have added Supplementary Note A to provide a structured and more in-depth comparison with conventional wireless wearable systems.

A. *HMI system comparison*:

"Wired human–machine interface (HMI) systems have historically provided reliable power delivery and stable data transmission[1,2]; however, their reliance on physical interconnections fundamentally constrains user mobility and limits their applicability in wearable and dynamic scenarios. Conventional wireless wearable systems address these limitations by adopting radiative electromagnetic (EM) communication, yet this approach introduces new system-level challenges associated with energy consumption, scalability, and signal security.

In conventional wireless systems, radiative EM propagation leads to significant path loss due to omnidirectional energy dispersion, requiring continuous power supply from onboard batteries to sustain communication[3,4]. Consequently, each sensing node must integrate its own power source, increasing device weight and form factor while imposing repeated maintenance requirements such as charging or replacement. As the number of nodes increases, the total system weight, energy demand, and maintenance burden scale proportionally, limiting the practicality of multi-node wearable deployments.

In contrast, the proposed metamaterial-enabled WBSN employs a surface-wave-guided transmission mechanism along the body. By confining electromagnetic energy to the body interface, this approach suppresses radiative losses and enables more efficient energy transfer between system components. This improved transmission efficiency reduces the communication power required at individual sensor nodes, thereby lowering the overall system power consumption, particularly in networks comprising multiple nodes. At the same time, the system supports centralized wireless power delivery, allowing multiple sensing nodes to operate without individual batteries. This fundamentally decouples power supply from node count, significantly enhancing scalability and reducing system complexity.

The confined nature of surface-wave propagation also leads to distinct advantages in communication characteristics[5]. Unlike radiative systems, where signals propagate into the surrounding environment and are susceptible to interference, surface waves remain localized along the body, improving communication stability while minimizing unintended radiation. This spatial confinement intrinsically limits signal leakage, making external interception more difficult and thereby enhancing physical-layer data security.

Overall, the proposed metamaterial-enabled WBSN establishes a system architecture that directly addresses the key bottlenecks limiting conventional wireless HMI systems. By integrating surface-wave-guided communication with centralized wireless power delivery, the system enables battery-free, distributed sensing with reduced power consumption, and enhanced communication reliability. These features collectively support scalable, low-latency, and unobtrusive multimodal sensing, allowing seamless data acquisition and interaction without restricting natural movement. As a result, the proposed WBSN provides a robust and

efficient platform for realizing continuous, unencumbered human–machine interfaces across diverse application scenarios."

5. *The description of the IMU integration is relatively brief. Additional clarification on the IMU signal processing and its specific role in the control strategy would improve technical clarity.*

Reply: We thank the reviewer for this constructive suggestion regarding technical clarity. In this work, the IMU is integrated as a representative sensing node to demonstrate the capability of the proposed WBSN to support heterogeneous wearable sensors within a battery-free framework. Accordingly, the original manuscript primarily emphasizes the system architecture and the key enabling component, namely the dual-mode metamaterial textile. That said, we agree that further clarification of the IMU signal processing and its role in the control strategy would enhance the technical completeness. In response, we have expanded the results to include a more detailed description of IMU data processing procedure, and its specific function in providing feedback for the control scheme.

"Since the robotic arm provides an interface for angular velocity control, to achieve intuitive control, we directly map the three-axis angular velocity data from the IMU to the three degrees of freedom of the robotic arm after mean filtering, and then perform closed-loop control through the robotic arm's back-end controller. Ultimately, this allows the robotic arm to smoothly follow the user's hand movements."

"... Fig. 5a shows the algorithm processing flow of the IMU. We used the Mahony algorithm to calculate the Euler angles of the IMU and performed low-pass filtering on the result. In order to reduce the zero drift of the IMU sensor output, we recorded the zero drift of the IMU for one second during the initialization process and compensated for it in the subsequent output results. ..."

6. *In Fig. 5g, some text annotations overlap with the heatmap regions, making local variations difficult to distinguish. The figure layout could be refined for improved readability.*

Reply:

We thank the reviewer for this helpful comment regarding figure clarity. In response, we have revised Fig. 5g-i to improve readability by adjusting the layout and repositioning the text annotations to avoid any potential visual overlap with the data points. These modifications

ensure that local variations can be more clearly distinguished. We appreciate the reviewer's careful reading of the manuscript.

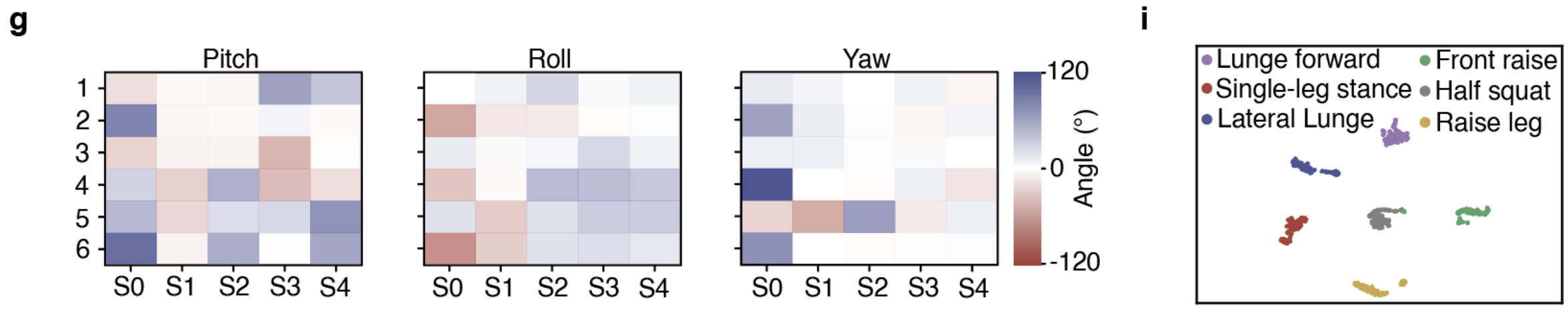

Fig. 5. **g**, Comparison of various poses recorded by camera and the same poses recovered by our model from the triaxial angle frames of battery-free WBSN. **i**, *t-SNE* plot from the pose dataset recorded by the battery-free WBSN. Distinct clusters corresponding to each pose illustrate the discriminative capability of the WBSN.

7. *The formatting of numerical values and units (e.g., "2.4 GHz", "13.56 MHz", and angle symbols such as "90°") is not fully consistent across several figures and axis labels. A thorough check of unit formatting throughout the figures (e.g., Figs. 3 and 4, Supplementary Figs. S10, S13, S17, and S23, among others) is recommended according to SI conventions.*

Reply: We thank the reviewer for this careful observation regarding unit consistency. In response, we have conducted a thorough review of all figures and axis labels, including Figs. 3 and 4 and Supplementary Figs. S10, S13, S17, and S23, among others. The formatting of numerical values, units (e.g., GHz, MHz), and angle notations (e.g., °) has been standardized throughout in accordance with SI conventions to ensure consistency and clarity.

Regarding the use of the degree symbol (°), we note that although the radian (rad) is the SI unit for plane angle, the degree is an SI-accepted non-SI unit that is widely adopted in scientific literature for expressing geometric and physical angles. In the context of this work, angular quantities are primarily used for descriptive purposes (e.g., bending and orientation), where the use of degrees enhances readability and aligns with common practice in dynamic motion sensing studies (e.g., *Nature Communications* 17, 991 (2026)). Accordingly, all angular quantities have been consistently expressed in degrees throughout the revised manuscript.

Again, we thank all the reviewers for their constructive feedback. We believe that the manuscript is much improved as a result.

Sincerely,

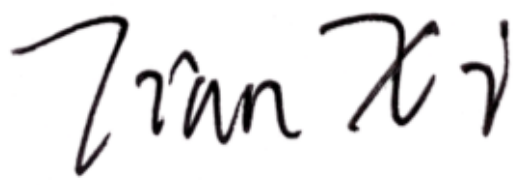

Xi Tian
Assistant Professor
Tsinghua Wireless Bioelectronics Group (https://tian-xi.cc)
Institute of Data and Information
Tsinghua University

Dr. Xi Tian
Tsinghua University
Institute of Data and Information
Information Building 1108
Shenzhen 518055, China
Email: tianxi@sz.tsinghua.edu.cn

June 13, 2026

Re: Nature communications manuscript NCOMMS-26-013708A, Yimeng, et al "Metamaterial-enabled battery-free wireless sensor networks for unencumbered human-machine interactions"

To the reviewers,

We sincerely appreciate the reviewer's thorough evaluation of our manuscript and the constructive feedback provided. Your comments have been invaluable in helping us refine and strengthen our work. The main text has been extensively revised to address the raised concerns, which are now highlighted in blue in the revised manuscript. We firmly believe that these revisions have significantly enhanced the quality of the manuscript. As detailed below, we address each of your concerns in detail.

Reviewer #1

Many of the individual technical elements, including metamaterial-assisted surface-wave communication on textiles, have been previously demonstrated. The present work appears primarily as a large-scale system integration effort rather than a fundamentally new wireless or metamaterial concept. Precisely because the contribution lies in systems integration, I had hoped the manuscript would more clearly disclose the practical key techniques that allow others to reproduce and build upon the integrated platform.

In particular, the previously requested S-parameter and field-mapping data characterizing the node-side coupling to the textile SSP waveguide are addressed largely through theoretical reasoning. The authors invoke the TM nature of the surface mode and the general current distribution of an inverted-F antenna, but do not provide measured S21 or impedance-matching data between the node antenna and the textile. The manuscript also indicates that the chip antenna integrated into the BLE MCU is used as-is. Such chip antennas are typically designed and matched for free-space radiation, and one would expect substantial detuning when placed against a textile SSP waveguide. The system clearly functions in the demonstrations, but as a systems integration study it remains somewhat unclear what design takeaway the reader should carry forward regarding how to engineer the node-side coupling for similar platforms.

I recognize that this regime is one in which device-level implementation know-how plays an outsized role, and I do not wish to press this point too strongly. The comment is offered as a suggestion for sharpening the practical value of the contribution rather than as a barrier to publication.

Reply:

We sincerely thank the reviewer for recognizing the value of our work in enabling large-scale system integration. We fully agree that providing additional details regarding node-side coupling would facilitate reproducibility and support future research in this area. In response to the reviewer's suggestion, we have added two new supplementary figures (Supplementary Fig. 30, 31) presenting measured S-parameters (S11 and S21) to characterize the node-side coupling behavior. Specifically, we investigated several antenna types that are commonly employed in wearable devices, including planar inverted-F antenna (PIFA), monopole antenna, and ceramic antenna, and demonstrated the gain afforded by metamaterial textiles for wireless communication.

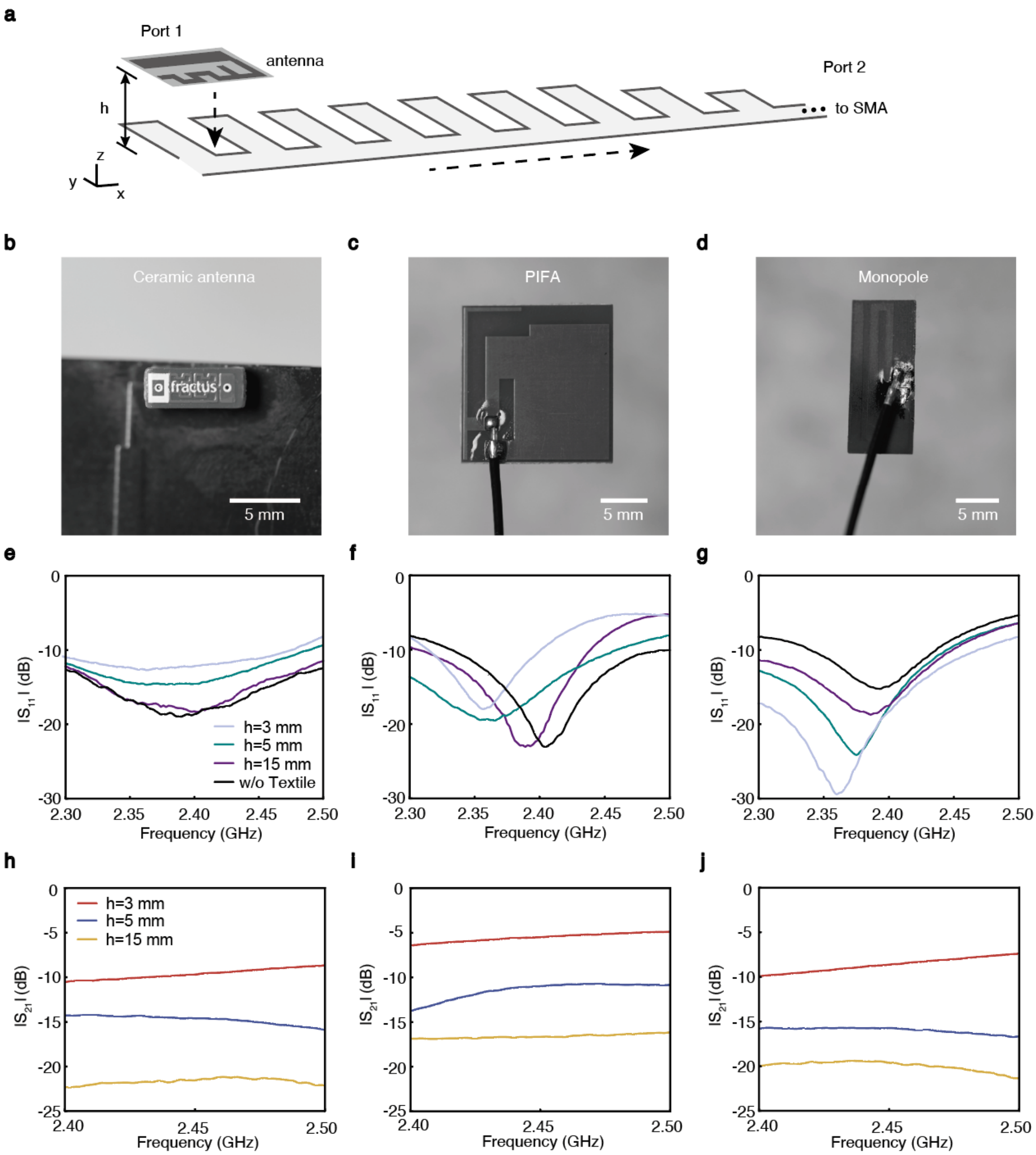

Fig. S30. Measured reflection and transmission characteristics of different commercial antennas in proximity to an SSP metamaterial textile. (a) Schematic illustration of the measurement setup, where h denotes the vertical separation between the antenna and the textile. (b–d) Photographs of the three transmitting antennas evaluated in this study: (b) ceramic antenna, (c) planar inverted-F antenna (PIFA), and (d) monopole antenna. (e–g) Measured reflection coefficient |S11| spectra of the three antennas under different separation distances (h = 3 mm, 5 mm, and 15 mm), together with the corresponding reference measurements obtained without the metamaterial textile. (h–j) Measured transmission coefficient |S21| spectra over the 2.4–2.5 GHz ISM band, characterizing the coupling efficiency between the antennas and the SSP waveguide, as well as the subsequent guided-wave transmission to the waveguide port.

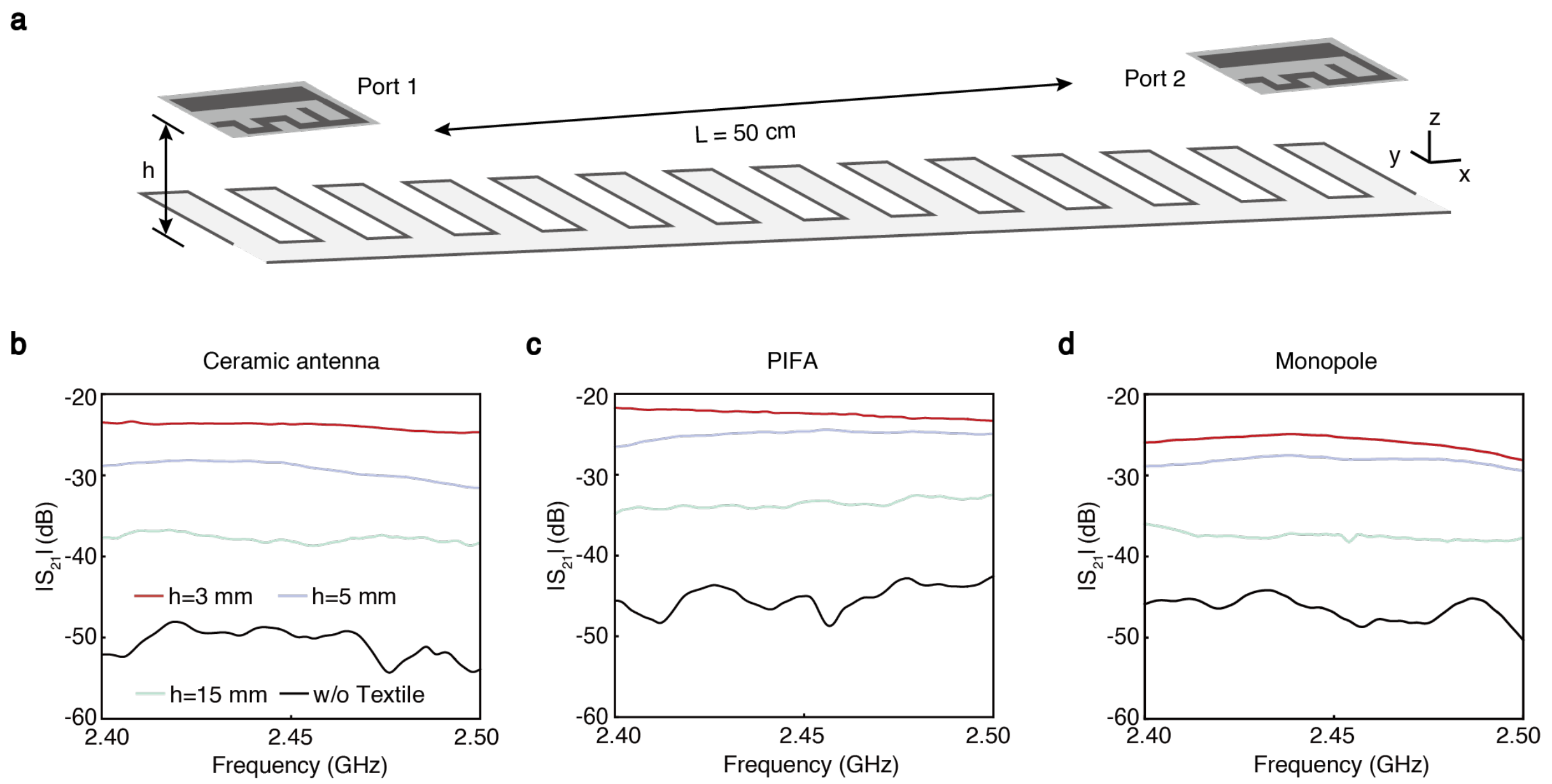

Fig. S31. Metamaterial-enhanced wireless transmission characteristics using different commercial antennas. (a) Schematic illustration of the measurement setup. Two antennas are placed above the metamaterial textile with a fixed separation distance of L = 50 cm, while the antenna-to-textile separation h is varied. (b-d) Measured transmission coefficient |S21| across the 2.40–2.50 GHz ISM band for (b) the ceramic antenna, (c) the PIFA, and (d) the monopole antenna.

First, we thank the reviewer for correctly pointing out that these antennas are typically designed and matched for free-space radiation, and therefore some degree of detuning is expected when they are placed in proximity to a textile SSP waveguide. As shown in Supplementary Fig. 30, the resonance frequency indeed shifts as the antenna approaches the textile waveguide, with a maximum shift of approximately 0.05 GHz. Nevertheless, even under these detuned conditions, the S11 value at 2.4 GHz remains below −8 dB for all tested antennas. More importantly, compared with conventional radiative communication, the metamaterial textile still provides more than ~20 dB improvement in transmission performance, as demonstrated in the new Supplementary Fig. 31. These results indicate that effective node-side coupling in the proposed system can be achieved using off-the-shelf antennas embedded in commercial BLE modules and smartphones, eliminating the need for antenna redesign or additional impedance matching. This provides a practical design guideline for future SSP-enabled systems and highlights a major practical advantage of our platform, allowing existing smartphones, sensors, and other HMI devices to be readily integrated without hardware redesign. Consequently, this drastically simplifies system implementation while maintaining compatibility with current wireless wearable devices. To clarify these points, we have revised the manuscript:

"... Meanwhile, to maximize practical deployability and compatibility with existing wireless hardware, we directly utilized the antenna integrated within the BLE MCU for 2.4 GHz signal

transmission. The corresponding reflection and transmission characteristics are presented in Supplementary Figs. 30–32."

To further validate the theoretical analysis presented in Supplementary Note B regarding the influence of antenna current distribution on coupling performance, we have added a new Supplementary Fig. 32 showing the current distributions of the antennas presented in Supplementary Fig. 30. It can be observed that the PIFA exhibits a strong unidirectional current component along the x-axis, whereas the monopole antenna contains noticeable counter-propagating current components. As detailed in our theoretical analysis (Supplementary Note B), such reverse current components tend to suppress the excitation efficiency of SSP modes. This prediction is further validated by the superior S21 performance of the PIFA observed in Supplementary Fig. 30. Therefore, while effective node-side coupling can be achieved using existing commercial antennas, antennas with source currents predominantly oriented along the x or z direction can further enhance SSP excitation efficiency and coupling performance.

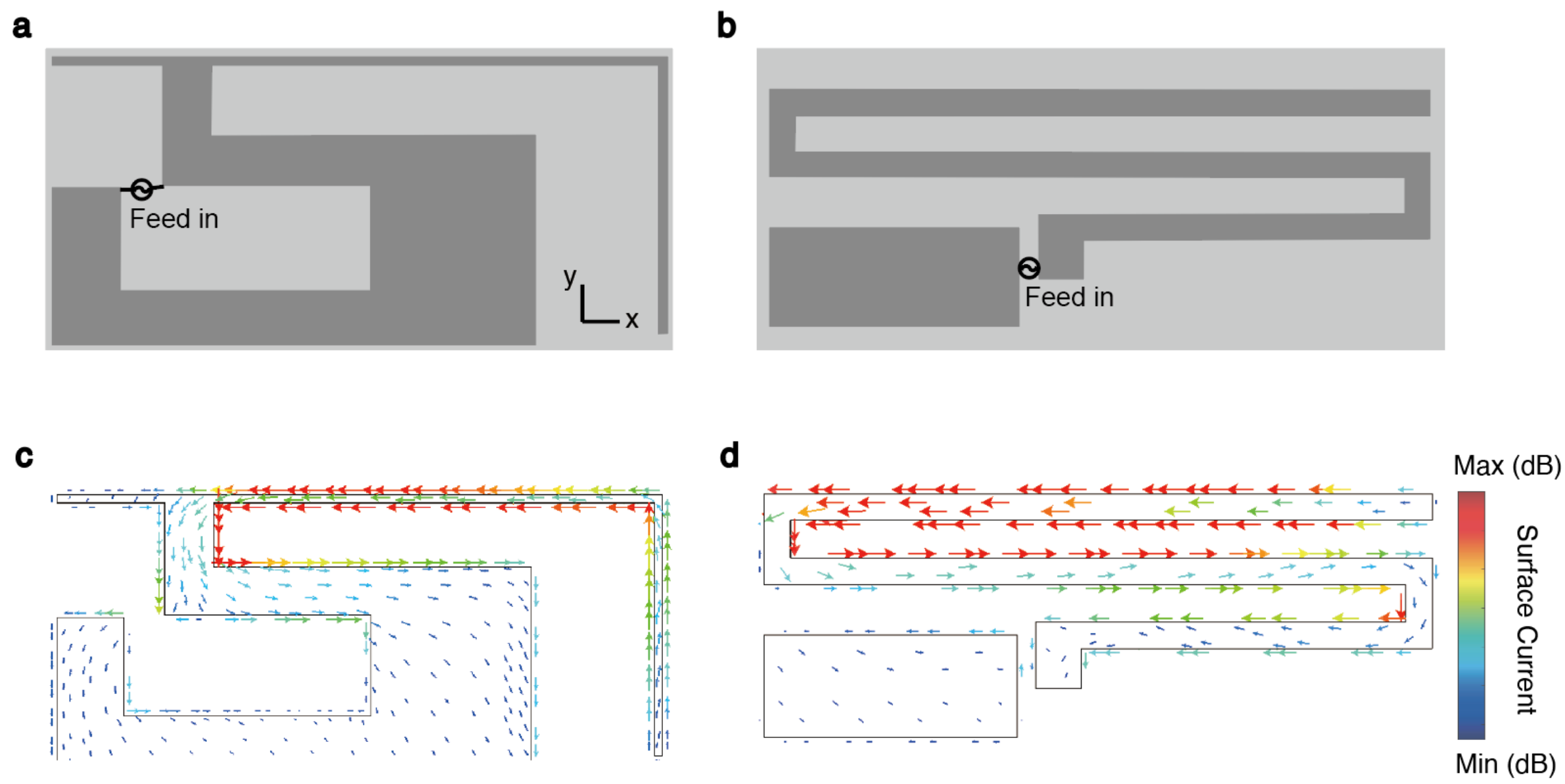

Fig. S32. Geometric structures and simulated surface current distributions of PIFA and monopole antenna. (a) Geometric structure of the PIFA. (b) Geometric structure of the monopole antenna. (c) Simulated surface current distribution on the PIFA at 2.4 GHz. (d) Simulated surface current distribution on the monopole antenna at 2.4 GHz.

On the other hand, we fully agree with the reviewer that custom-designed antennas with an optimized current distribution and improved impedance matching could further improve the coupling efficiency between the antenna and the textile SSP waveguide. While the present work deliberately employs off-the-shelf antennas integrated within commercial BLE modules and smartphones to maximize practicality and ease of deployment, we recognize antenna co-design as a promising direction for future research. Accordingly, we have added the following discussion to the Conclusion section:

"The present system operates directly with off-the-shelf antennas embedded in commercial BLE modules and smartphones, eliminating the need for antenna redesign. Future studies may investigate custom antenna designs with optimized current distributions and impedance matching to further improve coupling efficiency and communication robustness in particularly challenging deployment scenarios."

In addition, to facilitate the broader adoption of our technology in existing HMI devices, we have further enriched the implementation details by adding the circuit schematic of the NFC power-management module (new Supplementary Fig. 48) alongside the existing sensor-node schematic (Supplementary Fig. 47). Furthermore, we have included the detailed BLE configuration parameters used in our implementation.

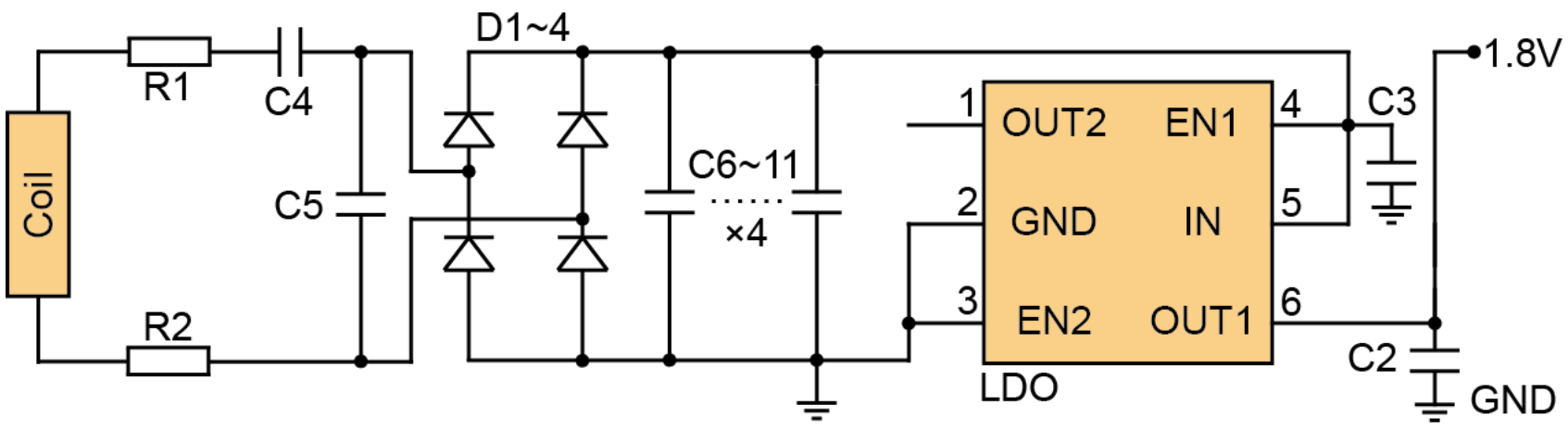

R1, R2	0 Ω	C6~11	22 uF
C2, C3	1 uF	C4, C5	100 pF

LDO	AP7312-1833
D1~4	CDBQC0130L-HF

Fig. S48. Power management circuit. Schematic diagram and component parameters of power management circuit.

"BLE configuration parameters. *This work adopts the BLE protocol. The relevant configuration parameters of the protocol stack are presented as follows: The advertising interval: 40 ms; The advertising duration: 180 s; Minimum acceptable connection interval: 20 ms; Maximum acceptable connection interval: 75 ms; Connection supervisory timeout: 4 s; TX power set: -20 dBm."*

We sincerely thank the reviewer for the constructive suggestions regarding the disclosure of additional implementation details for node-side coupling. The additional characterizations, design guidelines, and implementation details incorporated in the revised manuscript have substantially improved the reproducibility of the integrated platform and helped sharpen the practical value of the contribution. We believe these additions will enable other researchers to more readily reproduce, adapt, and build upon the proposed metamaterial-enabled HMI system.

Reviewer #2

I would like to thank the authors for their thorough revisions. The authors have satisfactorily addressed all of my minor concerns raised. I appreciate the addition of quantitative data regarding transmission latency and packet loss. I believe the manuscript has been significantly improved in terms of scientific soundness and clarity.

Reply:

We thank the reviewer for accepting our responses and the contribution on reviewing the paper. The reviewer's constructive comments have greatly enhanced the manuscript's quality.

Reviewer #3

The revision shows substantial effort with new data and figures. However, several first-round issues remain partially resolved, and new concerns have emerged.

[Major concerns]

***1.** Only 3 Chinese-market Android phones tested. No iPhone or Samsung included despite fundamentally different NFC implementations. Fig. S32 reports rectified voltage only, not power transfer efficiency. Please test iPhone/Samsung, or narrow claim to "NFC-enabled Android smartphones."*

Reply:

We thank the reviewer for the suggestion regarding the evaluation across different smartphone platforms. In response, we have performed additional tests using a Samsung smartphone and included the new results in the revised Supplementary Fig. 37. The results show that the rectified output voltage obtained from the Samsung smartphone is well above the input requirement of the LDO (2V), confirming that this Samsung phone can serve as a viable hub device for powering the proposed system.

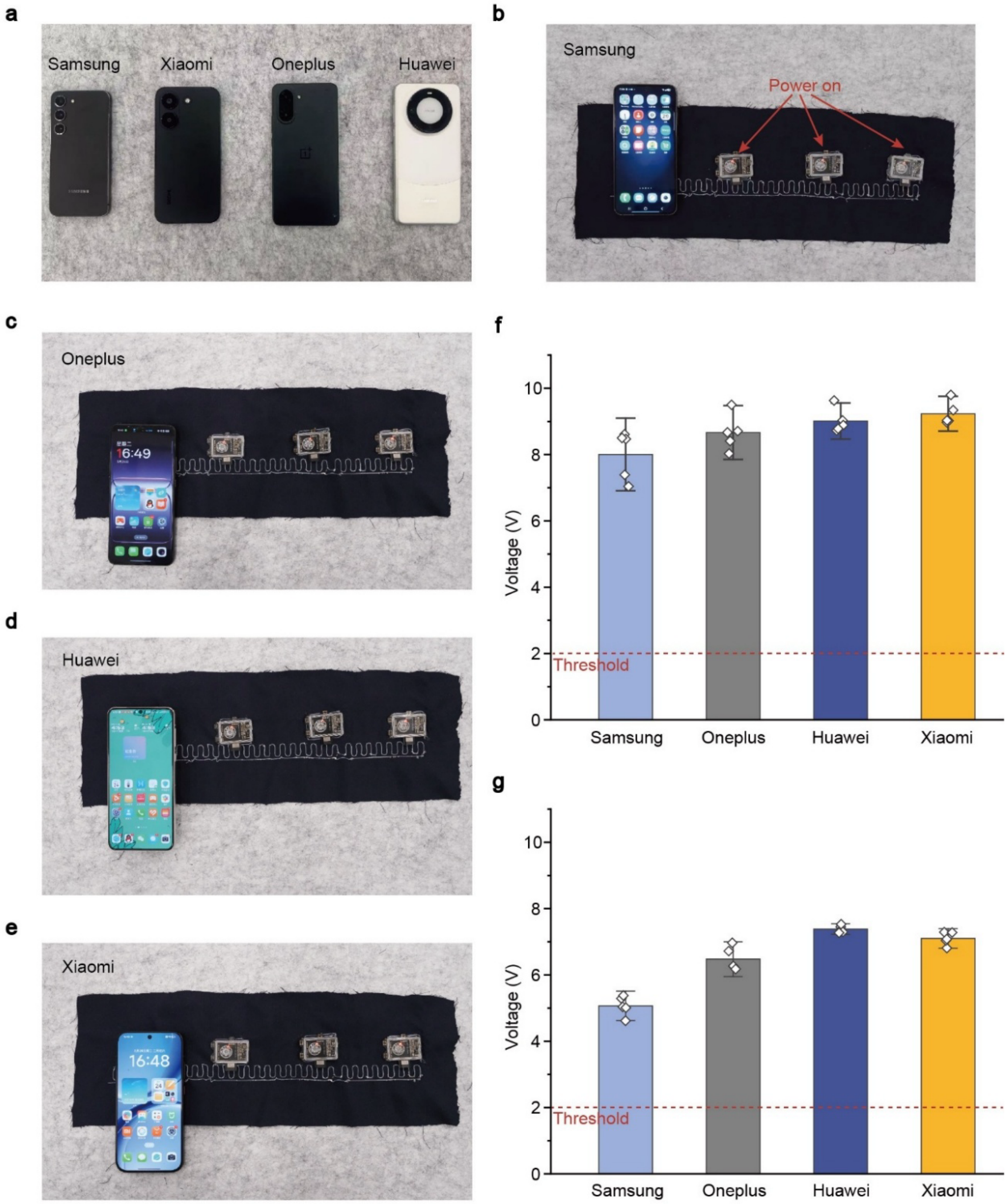

Fig. S37. Quantitative characterization of end-to-end power transfer using commercial smartphones. (a) Photographs of the four commercial smartphone models utilized for validation (Samsung S23, Xiaomi Redmi turbo5, OnePlus Ace5 and Huawei Mate60). (b-e) Experimental demonstrations of the smartphone-powered WBSN under a simultaneous three-node loading condition. (f) Measured rectified voltage of a single batter-free sensor node powered by four different smartphone models. (g) Average rectified voltage across three concurrent battery-free sensor nodes powered by different smartphone models.

We agree with the reviewer that smartphones from different manufacturers may adopt different NFC hardware architectures, antenna designs, and software-level access policies, which could potentially affect NFC powering performance. Nevertheless, as shown in Supplementary Fig. 37, the rectified voltages obtained from all tested smartphones are above the operating threshold of the LDO, confirming the feasibility of using commercially available NFC-enabled smartphones as hub devices for the proposed battery-free sensor network. The newly added Samsung result further supports the compatibility of the proposed system with mainstream Android smartphones beyond the originally tested Chinese-market models.

An iPhone-based implementation was not included in the current study because enabling the required NFC operation on iOS would require platform-specific development permissions and low-level system access that are not generally available through standard iOS NFC interfaces. Importantly, this does not reflect a fundamental limitation of the proposed approach. Rather, full iOS integration is primarily a product engineering task involving platform-specific software implementation and optimization, which is beyond the scope of this scientific proof-of-concept study.

To avoid overgeneralization, we have revised the relevant descriptions throughout the manuscript and now use "NFC-enabled Android smartphones" where appropriate.

Abstract*: "... When worn, the metamaterial-integrated clothing allows an <u>NFC-enabled smartphone</u> to serve as a central hub, ..."*

Introduction*: "... Recent works have shown the potential to mitigate this challenge by leveraging the ubiquitous <u>NFC-enabled smartphone</u> to serve as a wireless power source ..."*

Section 2B*: "... The functionality of this system was first validated using an <u>NFC-enabled Android smartphone</u> as a wireless power source and communication hub ..."*

Section 2C*: "... The wireless sensing system operates through a dual-mode metamaterial body network, which simultaneously transfers wireless power and data between an <u>NFC-enabled Android smartphone</u> and distributed sensor nodes ..."*

Section 2C*: "... Quantitative evaluation of power transfer performance under realistic smartphone operation is provided in Supplementary Fig. 37, demonstrating a substantial operating margin under practical, conditions and further supporting the feasibility of using a <u>commercial NFC-enabled Android smartphone</u> as a universal hub. ..."*

Figure 1*: "... d, Design concept of the battery-free WBSN, where an <u>NFC-enabled smartphone</u> acts as the central hub to construct body-scale wireless powering and communication networks through dual-mode metamaterial textiles. ..."*

We also thank the reviewer for this constructive comment, which allows us to clarify the efficiency metrics reported in Supplementary Fig. 37. The efficiency specified in the main manuscript refers to the coil-to-coil wireless power transfer efficiency from the transmitter coil to the receiver coil of the sensor node, as characterized in Fig. 2. This metric is distinct from a full end-to-end 'battery-to-BLE' system efficiency, which is challenging to define and reliably measure in real smartphone-based implementations.

In particular, commercial smartphones do not provide direct access to the power consumption of individual subsystems (e.g., the NFC module), and the overall system power usage is strongly influenced by dynamic OS scheduling and concurrent background processes, leading to significant measurement variability across devices and trials. Therefore, in the practical smartphone implementation, Supplementary Fig. 37 reports the rectified output voltage rather than the full end-to-end power transfer efficiency. This measurement directly verifies that sufficient power can be harvested from different smartphones to satisfy the input-voltage requirement of the LDO and support stable sensor-node operation.

Given that the core innovation of this work centres on the metamaterial-enabled coupling design, the coil-to-coil coupling efficiency provides a fair and physically meaningful metric for evaluating the wireless power transfer performance of the proposed textile-based metamaterial system. This approach is also consistent with prior studies on textile-based metamaterial and wireless power transfer systems [R1–R3].

To clarify this point, we have revised the manuscript as follows:

Section 2C*: "The rectified output voltage of sensor nodes was measured, demonstrating that sufficient power can be harvested from different smartphones to satisfy the input-voltage requirement of the LDO and support stable sensor-node operation."*

Methods*: "... In this study, the wireless power transfer efficiency is defined as the coil-to-coil coupling efficiency between the transmitter and receiver coils, measured in a benchtop setup to provide a consistent and comparable metric for evaluating metamaterial-enabled wireless power transfer systems[20,25,33]."*

[**R1**] Hajiaghajani, A., Afandizadeh Zargari, A.H., Dautta, M. et al. Textile-integrated metamaterials for near-field multibody area networks. Nat. Electron. 4, 808–817 (2021).

[**R2**] Zhu, X., Wu, K., Xie, X. et al. A robust near-field body area network based on coaxially-shielded textile metamaterial. Nat. Commun. 15, 6589 (2024).

[**R3**] Lin, R., Kim, HJ., Achavananthadith, S. et al. Digitally-embroidered liquid metal electronic textiles for wearable wireless systems. Nat. Commun. 13, 2190 (2022).

2. BLE connection parameters (advertising interval, TX power, etc.) and power management component values still missing. These are essential for reproducing the core system-level contribution. Please add BLE parameters and circuit values to Methods/SI.

Reply:

We thank the reviewer for raising this important point. These parameters are indeed crucial for reproducibility of the system-level implementation. In the revised manuscript, we have added the BLE configuration parameters in the Methods section as follows:

"BLE configuration parameters. *This work adopts the BLE protocol. The relevant configuration parameters of the protocol stack are presented as follows: The advertising interval: 40 ms; The advertising duration: 180 s; Minimum acceptable connection interval: 20 ms; Maximum acceptable connection interval: 75 ms; Connection supervisory timeout: 4 s; TX power set: -20 dBm."*

In addition, all circuit component values were previously included in the Supplementary Information (Fig. S47). To further improve clarity of the power management design, we have included the circuit schematic of the power management module in a new supplementary figure (Supplementary Fig. 48).

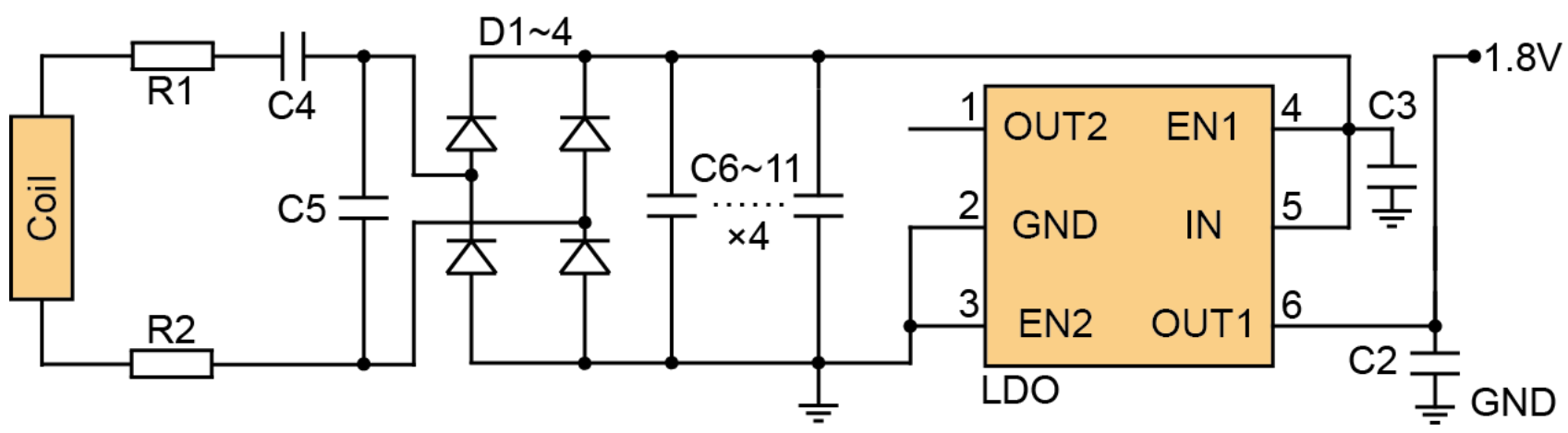

R1, R2	0 Ω	C6~11	22 uF	LDO	AP7312-1833
C2, C3	1 uF	C4, C5	100 pF	D1~4	CDBQC0130L-HF

Fig. S48. Power management circuit. Schematic diagram and component parameters of power management circuit.

3. 3 wash cycles cannot support "machine washable" claim (consumer standard: 50+ cycles). No quantified pre/post-wash performance data provided. Please report explicit degradation values, or reframe as "preliminary washability assessment."

Reply:

We thank reviewer for this constructive suggestion regarding the washability claim. We agree that a three-cycle test constitutes a preliminary evaluation rather than commercial certification. In response, we have added quantified pre- and post-wash performance data in Supplementary Table 2, including the explicit degradation values after repeated washing. We also clarify that our tests involve 3 washing cycles following the ISO 6330 standard for domestic laundering, which is intended to evaluate preliminary durability rather than certification-level "machine washable" performance.

Supplementary Table 2. Washing test of the metamaterial textile.

Cycles	Transmission coefficient at 2.4 GHz (dB)	Power Efficiency at 13.56 MHz (%)	Rectified Voltage (v)	Duration (min)
0	-18.4	75.86	8.2	0
1	-18.9	74.01	8.0	45
2	-19.3	73.08	7.9	45
3	-20.1	72.16	7.2	45

Accordingly, the manuscript has been revised to avoid overstatement, and the term "machine washable" has been replaced with "preliminary washability assessment". The updated description now reads:

Methods:

*"**Preliminary washability assessment.** Washing tests were performed in accordance with the ISO 6330 standard for domestic laundering to evaluate the <u>preliminary durability</u> and functional integrity of the metamaterial textile over repeated wash cycles. ..."*

"... Its electrical performance and transmission loss were subsequently characterized, and the pre- and post-wash variations are summarized in Supplementary Table 2. The results provide a <u>preliminary verification</u> that the textile retained its functionality after three washing cycles, with only limited performance degradation."

Section 2A:

"... Owing to these combined advantages, the resulting liquid metal fibers exhibit stable electrical performance, conformability to the curved surfaces of human body, and durability against mechanical deformation, <u>while also demonstrating preliminary washability</u>..."

4. *NFC coil radiates omnidirectionally and is not confined by the SSP structure. Security advantage applies only to 2.4 GHz BLE, not the entire system. Please limit security claim to BLE channel explicitly.*

Reply:

We thank the reviewer for the comment on communication security. In the proposed system, NFC is used exclusively for wireless power transfer and does not carry any communication data. Therefore, the security-related discussion in the manuscript is intended to apply only to the BLE communication channel rather than the entire system. We agree that this distinction was not sufficiently clear in the original manuscript. Accordingly, we have revised the relevant description in the manuscript to explicitly attribute the security advantage to BLE communication.

"... the SSP structure localizes electromagnetic propagation within close proximity to the body, thereby improving both transmission efficiency and BLE communication security ..."

5. *20% efficiency figure from benchtop, not smartphone excitation. No worst-case analysis despite visible voltage fluctuations during running (Fig. 5e). Please report minimum rectified voltage during motion and confirm it exceeds LDO dropout threshold.*

Reply:

We thank the reviewer for raising these points for clarification. As discussed in our **response to Question 1**, it is intrinsically challenging to quantify the overall power-transfer efficiency under smartphone excitation because commercial smartphones do not provide direct access to the real-time power consumption of individual subsystems, such as the NFC module. Therefore, the efficiency reported in the manuscript refers to the coil-to-coil coupling efficiency, which provides a standardized and physically meaningful metric to evaluate the wireless power transfer performance of the proposed metamaterial-enabled system. This evaluation approach is also widely adopted in prior studies on textile-based metamaterials. To clarify this point, we have revised the Section 2C and Methods of the manuscript.

To address the reviewer's concern regarding worst-case operating conditions, we further analyzed the rectified voltage data during motion. During running, the minimum transient voltages recorded at Node 1, Node 2, and Node 3 were 3.48 V, 2.89 V, and 2.80 V, respectively. All of these values remain above the 2.0 V input threshold of the LDO regulator, confirming stable circuit operation even during peak motion fluctuations. We have revised Figure 5e, its corresponding caption, and the relevant descriptions in the main manuscript accordingly.

"... As shown in Fig. 5e, the rectified voltages at all nodes remained consistently above the 2.0 V input threshold across all activities, satisfying the requirement for stable circuit operation. During high-intensity motion such as running, minor voltage fluctuations were observed due to transient coil misalignment. The minimum voltages measured at Node 1, Node 2 and Node 3 were 3.48 V, 2.89 V and 2.80 V respectively, all of which exceed the input requirement of the LDO chip. ..."

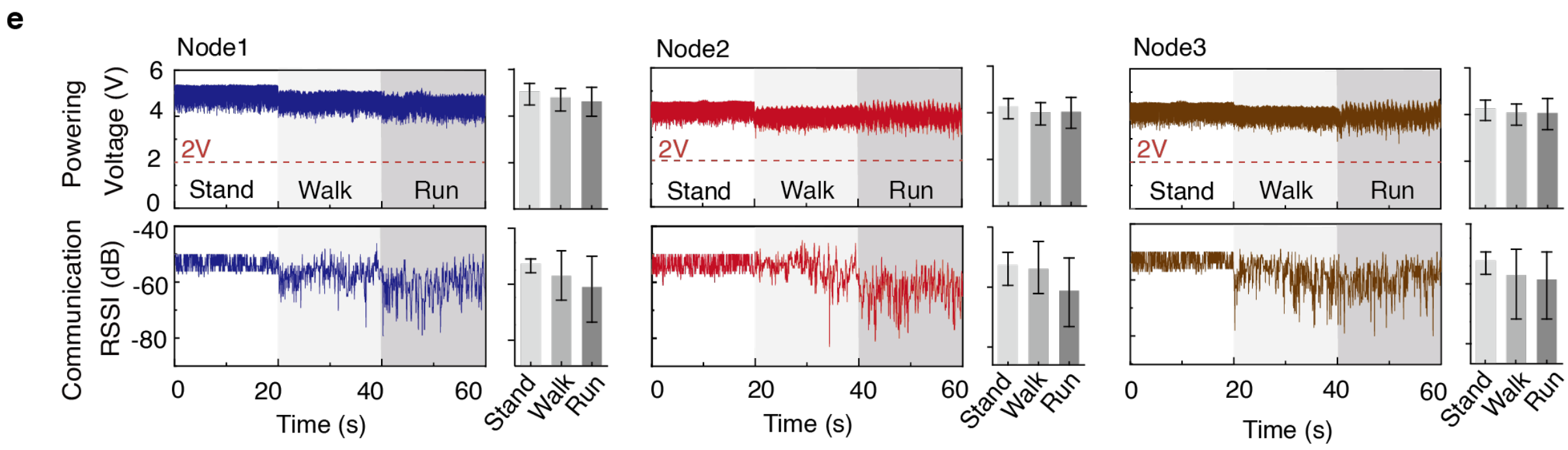

Fig. 5e, Wireless powering and communication performance during physiological activity. Rectified voltage and RSSI from each node were recorded, with average values compared across different activities. Error bars show mean ± s.d. of voltage and RSSI values calculated separately across the indicated period. The red dashed line indicates the minimum input voltage required for stable operation of the LDO chip.

***6.** N=3 subjects; random train/test split (not LOSO) inflates 99.5% accuracy; 6-class pose task is trivially separable; handwriting accuracy (82.8%) is low but undiscussed. Please acknowledge N=3 limitation, clarify that cross-subject generalization was not assessed, discuss handwriting accuracy limitations.*

Reply:

We thank the reviewer for these very insightful comments. We would like to clarify that the central contribution of this work lies in the proposed architecture of a battery-free wireless body sensor network (WBSN) enabled by the dual-mode metamaterial textile, and the concept of unencumbered HMI interaction. The posture and handwriting recognition tasks were designed as application-level, end-to-end functional demonstrations to showcase the practical utility of the proposed textile platform, rather than focusing on optimizing the sensing or machine-learning performance. Nevertheless, we agree that the limited number of participants (N = 3) represents a constraint of the current demonstration and does not allow a rigorous assessment of cross-subject generalization. We have therefore revised the manuscript to explicitly acknowledge this limitation as the reviewer suggested.

"... However, due to the limited number of participants, definitive conclusions regarding the model's generalization ability cannot be drawn from this experiment."

Regarding the handwriting recognition results, we agree that the achieved accuracy (82.8%) is noticeably lower than that of the posture recognition task and can be further discussed. The reduced performance mainly arises from three factors. First, relative displacement between the textile glove and the fingers during hand movements introduces motion artifacts and sensing errors. Second, the current drift-compensation strategy only corrects IMU zero drift using short-duration calibration data and cannot fully suppress accumulated measurement errors during dynamic handwriting. Third, reconstructing 2D trajectories from 3D in-air handwriting motions amplifies the influence of sensor noise and tracking inaccuracies. These factors collectively limit the current handwriting recognition performance.

We have added a corresponding discussion in the revised manuscript. Future improvements may be achieved through improved glove fit and conformability, more advanced drift-compensation and filtering algorithms, and user-specific training data that better capture individual handwriting characteristics.

"Testing on the held-out dataset achieved a recognition accuracy of 82.8%. This result suggests that the captured motion features contain discriminative information for character-level recognition, although the performance is affected by several practical factors, including relative displacement between the textile glove and the fingers during motion, residual IMU drift despite short-term calibration, and error amplification when reconstructing 2D trajectories from 3D in-air handwriting signals."

7. *Comparison is against free-space propagation around a bent arm (naturally extreme path loss), not a fair baseline. A simple conductive trace would be more informative. Please add conductive trace comparison or moderate the language.*

Reply:

We thank the reviewer for raising the comparison with simple conductive trace. To provide a more informative baseline, we have included new simulation and experimental results comparing the SSP metamaterial textile with simple conductive traces under both straight and bent-arm configurations (new Supplementary Fig. 27 and 28).

First, we have included a straight-line comparison in the new Supplementary Fig. 27. The results demonstrate that the SSP metamaterial pattern plays a critical role in enhancing the transmission performance. In contrast, the conductive trace without SSP structures exhibits

significantly weaker transmission efficiency, indicating that efficient guided-wave propagation cannot be established in the absence of the SSP pattern.

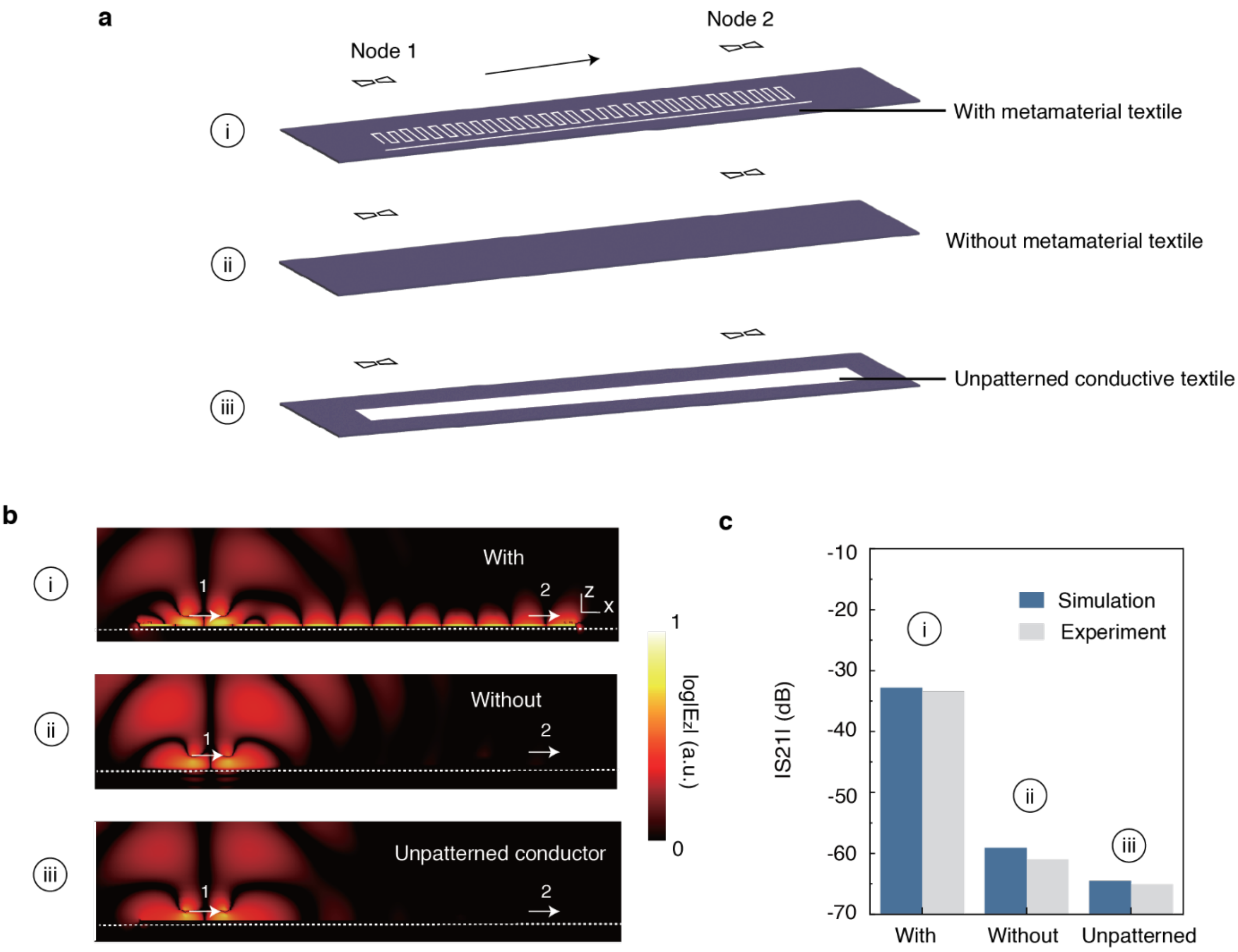

Fig. S27. Comparison of wireless communication between different textiles. (a) Schematic illustration of comparison setup. Two antennas are placed 5mm above the textiles with a fixed separation distance of L = 50 cm. (b) Simulated illustration of electric field distribution emitted by a dipole above a metamaterial textile (top), normal textile (centre) and unpatterned conductor (bottom). The textile is placed on an air–body half-space. Only metamaterial textile supports the surface wave propagation. (c) Measured and simulated results of transmission coefficient between different textiles.

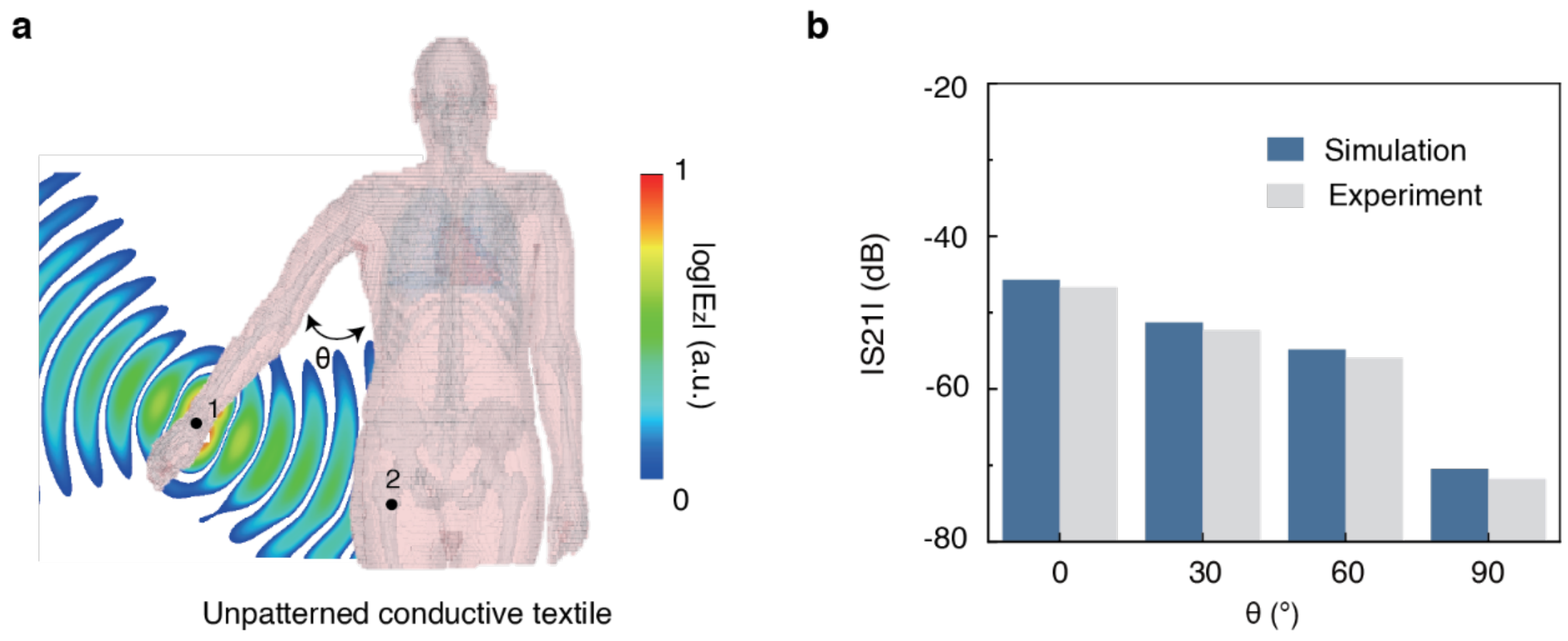

Fig. S28. Wireless communication performance of the unpatterned conductive textile on the human body. (a) Simulated electric field distribution. (b) Simulated and on-body measured transmission coefficient as functions of arm-raising angle, with the unpatterned conductive textile. The data are plotted based on single measurements.

In addition, we measured the transmission performance of the conductive trace in the bent-arm scenario and included the results in the new Supplementary Fig. 28. Consistent with the straight-line comparison, the conductive trace shows substantially lower transmission efficiency than the SSP metamaterial reported in main Fig. 2, further confirming the effectiveness of the SSP-enabled waveguiding mechanism.

To clarify this point, we have revised the corresponding discussion in the manuscript and moderated the language where appropriate:

"The simulation and measurement results in Supplementary Fig. 27, 28 further demonstrate that a conductive trace without SSP structures cannot effectively support surface wave propagation and exhibits significantly weaker transmission efficiency."

8. *Phone battery drain dominated by screen/OS, not NFC; RSSI stability not quantified; IMU gyroscope drift over 6 hours not characterized. Please report RSSI mean ±std, acknowledge IMU drift, remove or qualify "12 hours" projection.*

Reply:

We thank the reviewer for the valuable and professional comments. In response to the reviewer's suggestion, we have added quantitative RSSI data (−54.34±4.72 dB) in the caption of Supplementary Fig. 38.

"FIG. S38. Long-term continuous operation of the smartphone-powered WBSN system. The RSSI of a sensor node remained stable throughout the 6-hour continuous operation, with an average value of -54.34±4.72 dB, demonstrating stable wireless communication performance under extended operation."

We also acknowledge that drift is an inherent issue in IMU-based sensing systems. This drift originates from multiple factors, including manufacturing offset of the chip, temperature-induced drift, structural stress and other factors, which is a well-known limitation of IMUs. Since angular data from the gyroscope is obtained via integration, zero drift will accumulate during this process. To further characterize this effect in our system, we measured the static IMU drift over a six-hour period. The accumulated angular drift was 0.69°, 0.12°, and 0.22° for the roll, pitch, and yaw axes, respectively. These results have now been explicitly reported in the Supplementary Fig. 38 caption.

"... It is noted that IMU measurements inherently suffer from time-dependent drift; after 6 hours of static operation, the accumulated angular drift reached 0.69°, 0.12°, and 0.22° for the roll, pitch, and yaw axes, respectively."

Regarding power consumption, we agree with the reviewer that it is not rigorous to assume a linear extension of system endurance to 12 hours based solely on six-hour measurements. Therefore, the corresponding statement has been removed from the revised manuscript.

[Minor comments]

9. *Grammar: "excite" → "excites" in Section 2C.*

Reply:

We thank the reviewer for carefully pointing out the grammatical issue. The grammar in Section 2C has been revised accordingly. We have also carefully checked the entire manuscript to avoid similar issues.

"... this configuration, the generated source excites surface waves on the textile SSP waveguide, which then guide the transmitted signals to a smartphone receiver. ..."

10. *"More than an order of magnitude" repeated 3x without stating actual value — include in Abstract.*

Reply:

We thank the reviewer for the very careful review of the manuscript. We have revised these descriptions in the manuscript:

Abstract:

"This architecture enhances data throughput by over an order of magnitude (37.8 times) and doubles wireless power transfer efficiency compared with emerging near-field clothing."

Introduction:

"We fabricate the metamaterial textiles using computer-aided digital embroidery of liquid metal and show that they enhance wireless data throughput by over one order (37.8 times) while simultaneously doubling power-transfer efficiency compared with emerging near-field clothing[19,20,24]"

Conclusion:

"We demonstrated that the dual-mode metamaterial design supports simultaneous high-efficiency data transmission and wireless power transfer, achieving more than an order of magnitude (37.8 times) enhancement in data throughput and a twofold improvement in power transfer efficiency compared with emerging near-field clothing."

11. *Fig. 2m: clarify whether data points have error bars or are single measurements.*

Reply:

We thank the reviewer for raising this point for clarification. The data points in Fig. 2m represent single measurements and therefore do not include error bars. This has now been clarified in the figure caption:

*"... **m**, Simulated and on-body measured power transfer efficiency and transmission coefficient as functions of arm-raising angle, with and without the metamaterial textile, demonstrating angular stability and robustness to body movement. The data are plotted based on single measurements."*

Again, we thank all the reviewers for their constructive feedback. We believe that the manuscript is much improved as a result.

Sincerely,

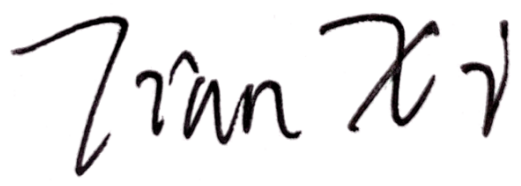

Xi Tian
Assistant Professor
Institute of Data and Information
Tsinghua University

Dr. Xi Tian
Tsinghua University
Institute of Data and Information
Information Building 1108
Shenzhen 518055, China
Email: tianxi@sz.tsinghua.edu.cn

July 30, 2026

Re: Final revisions for Nature Communications manuscript NCOMMS-26-013708B, Yimeng, et al “Metamaterial-enabled battery-free wireless sensor networks for unencumbered human-machine interactions”

To the reviewers,

We thank all the reviewers for their insightful and constructive comments on our manuscript. Please find below a point-by-point response to each reviewer.

Reviewer #3

The authors have revised the manuscript NCOMMS-26-013708, titled "Metamaterial-enabled battery-free wireless sensor networks for unencumbered human-machine interactions," to address the second-round comments. The revision includes new experimental data such as the Samsung S23 measurement (Supplementary Fig. 37), straight-line and bent-arm conductive-trace baselines (Supplementary Figs. 27-28), the minimum rectified voltage during running, the quantified RSSI and IMU drift over 6 hours, and a preliminary washability table. The BLE configuration parameters and the power management circuit schematic are also newly disclosed, and the previously overclaimed expressions have been appropriately moderated. In this regard, I am satisfied with the overall direction of the revision, and most of my prior concerns seem to be adequately addressed. Only a couple of points may need to be mentioned further, none of which are critical. My assessment of the specific responses is as follows.

Reply: We thank the reviewer for accepting our responses and the contribution on reviewing the paper.

***1.** Regarding the universal smartphone hub claim, the addition of the Samsung S23 measurement and the consistent replacement with "NFC-enabled Android smartphone" throughout the manuscript is a reasonable resolution. The explanation that iOS integration is mainly a product-engineering task rather than a scientific limitation appears acceptable. I accept this response.*

Reply: We thank the reviewer for accepting our responses.

***2.** The BLE configuration parameters (advertising interval, connection interval range, supervision timeout, TX power) and the power management circuit schematic in Supplementary Fig. 48 provide sufficient detail for the system to be reproduced. This point is resolved.*

Reply: We thank the reviewer's confirmation.

***3.** The washability claim is now framed as a "preliminary washability assessment," and the pre- and post-wash performance is quantified in Supplementary Table 2. I appreciate that the authors chose to downgrade the language rather than defend the original expression. It would be beneficial to add one summary row showing the raw percentage change to Table 2 for easier interpretation.*

Reply: We thank the reviewer for this constructive suggestion. We have added a summary row of raw percentage changes in Supplementary Table 2 to simplify data reading.

TABLE S2. Washing test of the metamaterial textile

Cycles	Transmission coefficient at 2.4 GHz (dB)	Power Efficiency at 13.56 MHz (%)	Rectified Voltage (v)	Duration (min)
0	-18.4	75.86	8.2	0
1	-18.9	74.01	8.0	45
2	-19.3	73.08	7.9	45
3	-20.1	72.16	7.2	45
Percentage change after 3 cycles	-9.24%	-4.88%	-12.20%	—

***4.** The security claim is now explicitly limited to the BLE channel, and the manuscript clarifies that the NFC link carries only power. This is acceptable.*

Reply: We thank the reviewer for accepting our responses.

***5.** Reporting the minimum rectified voltages during running (3.48, 2.89, and 2.80 V) with the LDO threshold marked on Fig. 5e directly answers the worst-case concern. The margin at Node 3, about 0.8 V above the 2.0 V threshold, seems adequate but not large. A brief note in the Discussion that this margin may narrow under more extreme motion regimes would be helpful for the readers.*

Reply: We thank the reviewer for this valuable suggestion. We have added a brief description in the manuscript to clarify this point.

"Moreover, the regulated outputs of all nodes maintained sufficient margins to ensure uninterrupted operation. Under more severe motion conditions, this voltage margin may be further reduced."

***6.** For the ML validation, the authors have added the acknowledgement that N = 3 does not permit conclusions regarding cross-subject generalization, and the mechanisms behind the lower handwriting accuracy are discussed. The leave-one-subject-out evaluation was not performed, and instead the ML demonstrations are reframed as end-to-end functional showcases rather than ML claims. Given that the central contribution of the paper is the metamaterial-textile system, I think this reframing is reasonable. One additional sentence noting that the 99.5% posture accuracy also reflects the fact that the six poses involve distinct limb configurations would improve the calibration further. This is only a suggestion.*

Reply: We thank the reviewer's constructive suggestion. We have added a sentence in the manuscript to explain this point.

"The trained LSTM model achieved an average recognition accuracy of 99.5% in three rounds of test set validation. The high recognition accuracy can be partly attributed to the pronounced morphological distinctiveness of the limb postures across the tested movements."

***7.** The new Supplementary Figs. 27 and 28 comparing the SSP metamaterial with a simple conductive trace, under both straight and bent-arm configurations, directly address the*

previous concern about the "three orders of magnitude" comparison. The language in the main text has also been appropriately hedged. This concern is now handled.

Reply: We thank the reviewer for accepting our responses.

8. *The RSSI is reported as -54.34 +/- 4.72 dB over the 6-hour window, the static IMU drift is quantified per axis (0.69, 0.12, and 0.22 degrees for roll, pitch, and yaw), and the "12 hours of continuous operation" projection has been removed. All three sub-points are addressed.*

Reply: We are grateful to the reviewer for the positive reception of our earlier replies.

9. *The grammatical correction in Section 2C, the inclusion of the 37.8x figure in the Abstract, and the clarification that the Fig. 2m data points are single measurements are all reflected in the revised text. These minor points are resolved.*

Reply: We thank the reviewer for accepting all minor textual and figure description revisions.

Overall, the manuscript appears to have reached the level appropriate for Nature Communications. The engineering achievement is substantial, the claims are now proportional to the evidence, and the reproducibility gaps have been closed. I have no further major concerns and recommend acceptance, subject to the minor editorial suggestions above.

Reply: We thank the reviewer for positive feedback and recommendation of our work.

Sincerely,

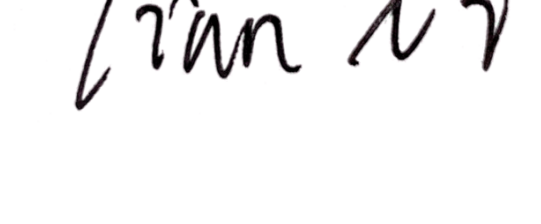

Xi Tian
Assistant Professor
Institute of Data and Information
Tsinghua University
