# Supplementary material for: Metamaterial-enabled battery-free wireless sensor networks for unencumbered human-machine interactions
Source: Nat Commun. 2026 Sep 14;17:10778. doi: 10.1038/s41467-026-77600-2 (PMC13661596; doi:10.1038/s41467-026-77600-2)
Supplement: Supplementary file 2 [file 41467_2026_77600_MOESM1_ESM.pdf]

Supplementary materials: Metamaterial-enabled battery-free wireless sensor networks for unencumbered human-machine interactions

Yimeng Zhang*,[1,2] Fengman He*,[1,3,4] Tianyiyi He*,[2,a)] Qianrui Luo,[1] Juerui Lin,[1] Qinghao Xu,[1] Changqing Guo,[1] Xinyue Chai,[1] Yuchen Wang,[1] Tong Wu,[1] Yiru Jiang,[2] Chun Jin,[1] Xiping Hu,[2,5,b)] Wenbo Ding,[1,c)] and Xi Tian[1,3,d)]

[1)] *Institute of Data and Information, Tsinghua Shenzhen International Graduate School, Tsinghua University, Shenzhen 518055, China*

[2)] *Artificial Intelligence Research Institute, Shenzhen MSU-BIT University, Shenzhen 518172, China*

[3)] *Center of Double Helix, Tsinghua Shenzhen International Graduate School, Tsinghua University, Shenzhen 518055, China*

[4)] *Shenzhen X-Institute, Nanshan District, Shenzhen 518055, China*

[5)] *School of Medical Technology, Beijing Institute of Technology, Beijing, 100081, China*

(Dated: 14 August 2026)

[a)]Electronic mail: tianyiyi@smbu.edu.cn

[b)]Electronic mail: huxp@bit.edu.cn

[c)]Electronic mail: ding.wenbo@sz.tsinghua.edu.cn

[d)]Electronic mail: tianxi@sz.tsinghua.edu.cn

I. SUPPLEMENTARY NOTE

A. HMI system comparison

Wired human–machine interface (HMI) systems have historically provided reliable power delivery and stable data transmission[1,2]; however, their reliance on physical interconnections fundamentally constrains user mobility and limits their applicability in wearable and dynamic scenarios. Conventional wireless wearable systems address these limitations by adopting radiative electromagnetic (EM) communication, yet this approach introduces new system-level challenges associated with energy consumption, scalability, and signal security.

In conventional wireless systems, radiative EM propagation leads to significant path loss due to omnidirectional energy dispersion, requiring continuous power supply from onboard batteries to sustain communication[3,4]. Consequently, each sensing node must integrate its own power source, increasing device weight and form factor while imposing repeated maintenance requirements such as charging or replacement. As the number of nodes increases, the total system weight, energy demand, and maintenance burden scale proportionally, limiting the practicality of multi-node wearable deployments.

In contrast, the proposed metamaterial-enabled WBSN employs a surface-wave-guided transmission mechanism along the body. By confining electromagnetic energy to the body interface, this approach suppresses radiative losses and enables more efficient energy transfer between system components. This improved transmission efficiency reduces the communication power required at individual sensor nodes, thereby lowering the overall system power consumption, particularly in networks comprising multiple nodes. At the same time, the system supports centralized wireless power delivery, allowing multiple sensing nodes to operate without individual batteries. This fundamentally decouples power supply from node count, significantly enhancing scalability and reducing system complexity.

The confined nature of surface-wave propagation also leads to distinct advantages in communication characteristics[5]. Unlike radiative systems, where signals propagate into the surrounding environment and are susceptible to interference, surface waves remain localized along the body, improving communication stability while minimizing unintended radiation. This spatial confinement intrinsically limits signal leakage, making external interception more difficult and thereby enhancing physical-layer data security.

Overall, the proposed metamaterial-enabled WBSN establishes a system architecture that directly addresses the key bottlenecks limiting conventional wireless HMI systems. By integrating surface-wave-guided communication with centralized wireless power delivery, the system enables battery-free, distributed sensing with reduced power consumption, and enhanced communication reliability. These features collectively support scalable, low-latency, and unobtrusive multimodal sensing, allowing seamless data acquisition and interaction without restricting natural movement. As a result, the proposed WBSN provides a robust and efficient platform for realizing continuous, unencumbered human–machine interfaces across diverse application scenarios.

B. Surface wave excitation

In this section, we derive the relationship between the surface wave amplitude and the source current on the antenna to provide a theoretical foundation for understanding the excitation of surface wave on the metamaterial textile[5,6].

We consider an interface model between air and the body: the upper half-space ($z > 0$) is free space, and the lower half-space ($z < 0$) is filled with biological tissue of permittivity ϵ_{body}. A thin planar metamaterial placed on the interface $z = 0$ supports surface-plasmon-like modes propagating in the $\pm x$-directions.

Although the field distribution depends on the detailed structure, the electric field can be approximated as

$$\mathbf{E}_{\pm}(\mathbf{r}) = p(y) \begin{pmatrix} i\alpha_n/\beta \\ 0 \\ 1 \end{pmatrix} e^{\pm i\beta x - \alpha_n |z|} \tag{1}$$

where $p(y)$ describes the field profile in the y-direction, α_n are the decay constants in the upper ($n = 1$) and lower ($n = 2$) regions, β is the propagation constant, and a time dependence $e^{-i\omega t}$ is assumed. The propagation constant β follows the surface-plasmon-like dispersion relation

$$\beta = k_0 \sqrt{\frac{\epsilon_1 \epsilon_2}{\epsilon_1 + \epsilon_2}} \tag{2}$$

where $k_0 = \omega/c$, $\epsilon_2 = 1 - \omega_p^2/\omega^2$, and ϵ_1 and ω_p are fitting parameters.

The electric field expressions indicate that the SSP modes exhibit transverse magnetic characteristics and are highly confined near the structure, which are also verified by sim-

ulation results (Supplementary Figure 5). Due to the evanescent field extending from the surface of the SSP textile, surface modes can be wirelessly excited by nearby antennas. The surface excitation effect can be described by separating the electric field of the surface mode into forward (+) and backward (−) propagating components:

$$\mathbf{E}(\mathbf{r}) = a_+\mathbf{E}_+(\mathbf{r}) + a_-\mathbf{E}_-(\mathbf{r}) \tag{3}$$

where $a_\pm$ are the mode amplitudes and $\mathbf{E}_\pm$ are normalized such that

$$\frac{1}{2}\int |p(y)|^2 \, dy = \frac{\alpha_1\alpha_2}{\alpha_1 + \alpha_2} \tag{4}$$

When a source with current density $\mathbf{J}(\mathbf{r})$ is placed above the SSP clothing, the surface modes are excited with amplitude

$$a_\pm = -\frac{\beta}{2\omega\epsilon_0}\int \mathbf{J}(\mathbf{r}) \cdot \mathbf{E}_\mp(\mathbf{r}) \, d\mathbf{r} \tag{5}$$

Equation (5) shows that excitation depends only on the overlap between the source current density and the evanescent field above the surface. Since excitation occurs in the near-field of the antenna, far-field properties such as gain and directivity do not directly determine the amplitude of the excited mode.

II. SUPPLEMENTARY FIGURES

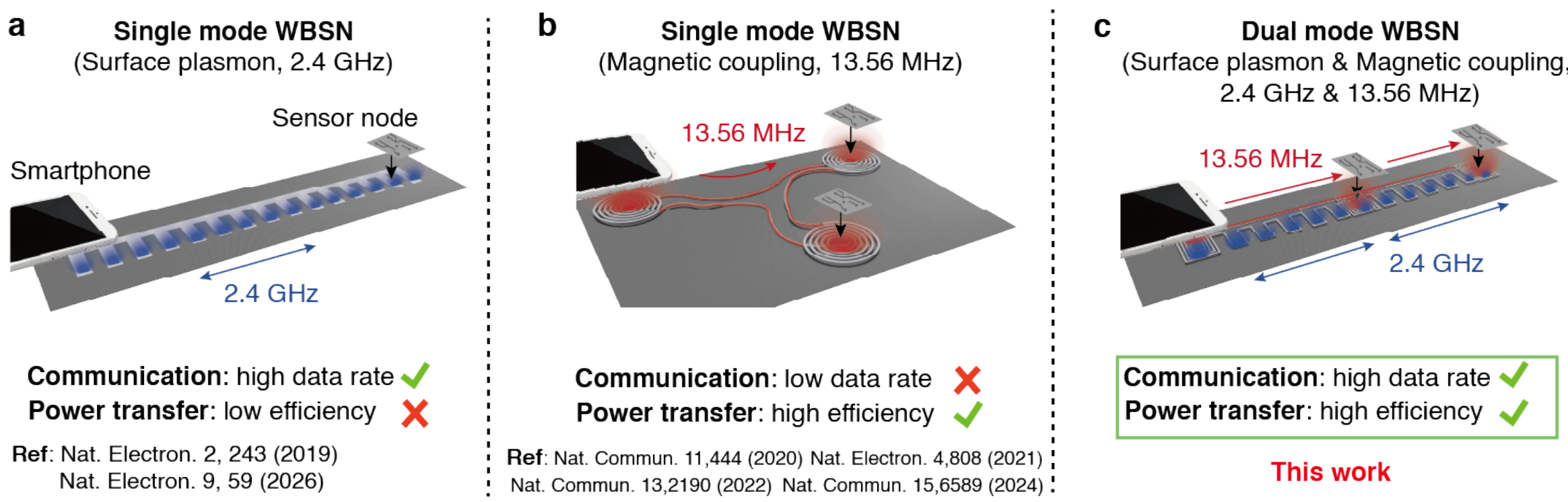

Supplementary Figure 1. **Comparison of key performance characteristics of metamaterial-based wireless body sensor network (WBSN).** (**a**) Single mode WBSN based on surface plasmon. (**b**) Single mode WBSN based on magnetic coupling. (**c**) Dual mode WBSN.

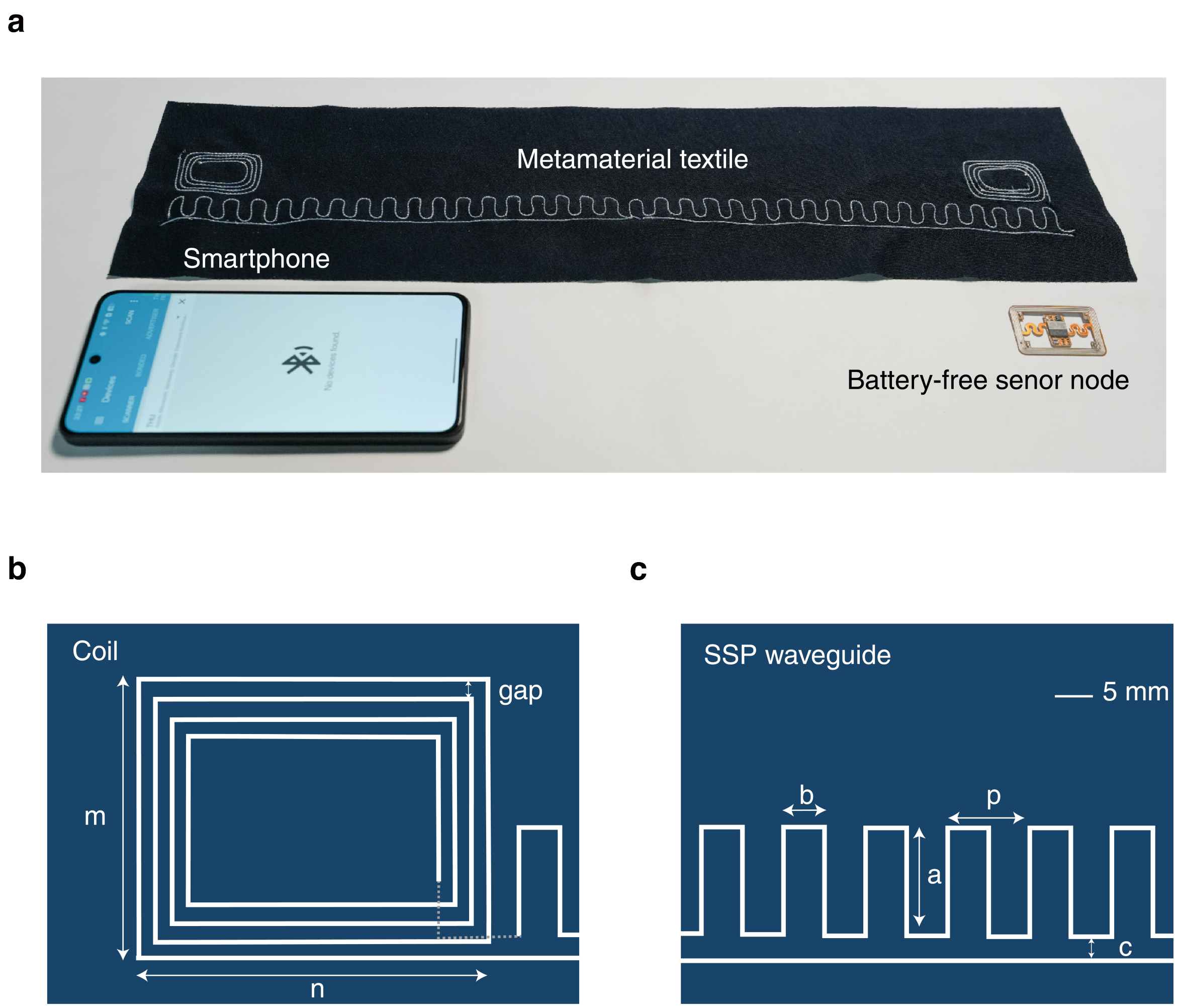

Supplementary Figure 2. **Design of dual-mode metamaterial textiles. (a)** Metamaterial enabled battery-free wireless sensor networks for powering and communication between a smartphone and a sensor node. **(b,c)** Structure of dual-mode metamaterial textiles including coils (m = 40 mm, n = 30 mm, gap = 1.6 mm) and SSP waveguides (a = 14 mm, b = 6 mm, c = 2 mm, p = 12 mm) for powering supply and data transmission.

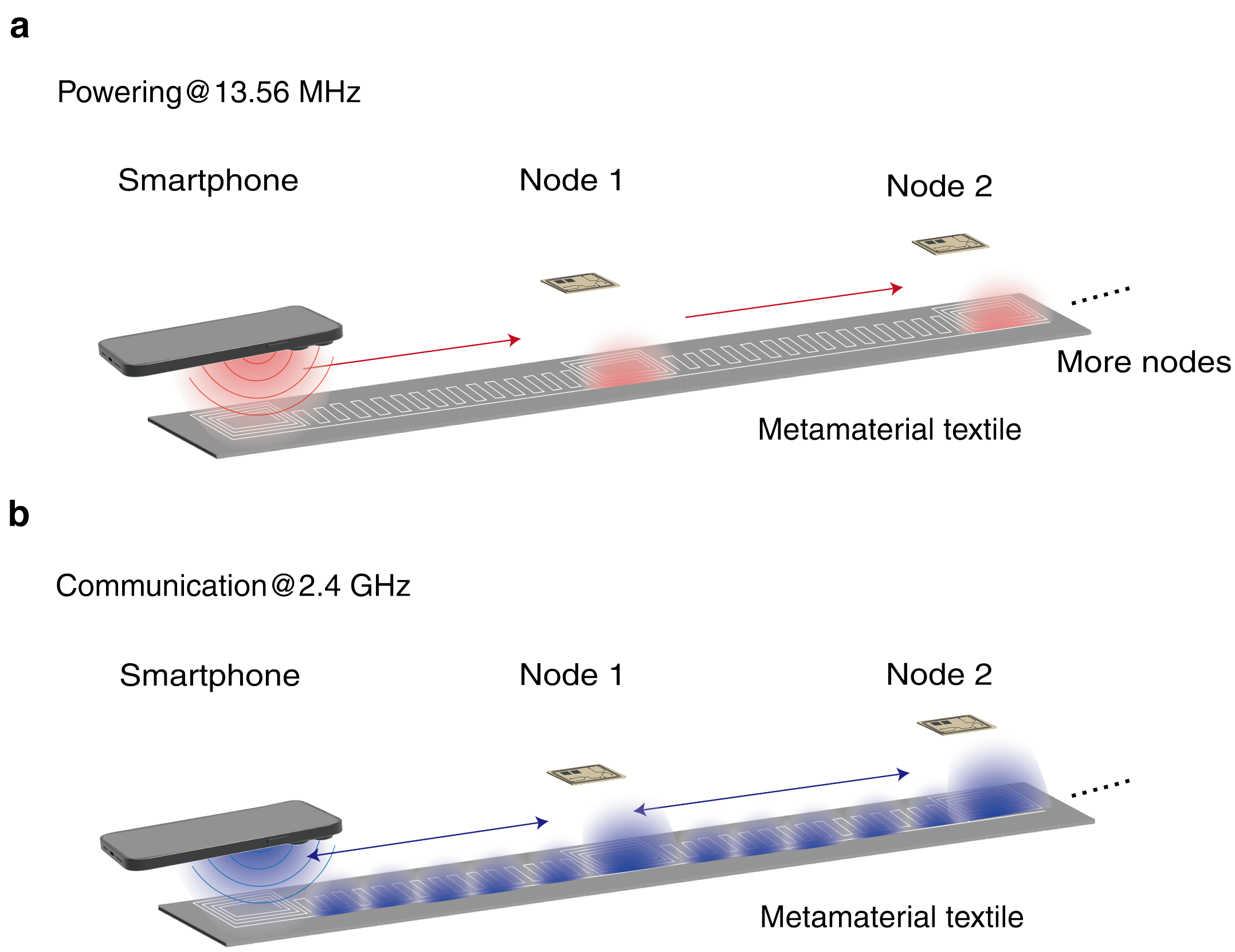

Supplementary Figure 3. **Working mechanism of dual-mode metamaterial textiles.** (**a**) llustration of the wireless powering transfer using near-field 13.56 MHz for wireless sensor network. (**b**) llustration of the data communication using bluetooth 2.4 GHz for wireless sensor network.

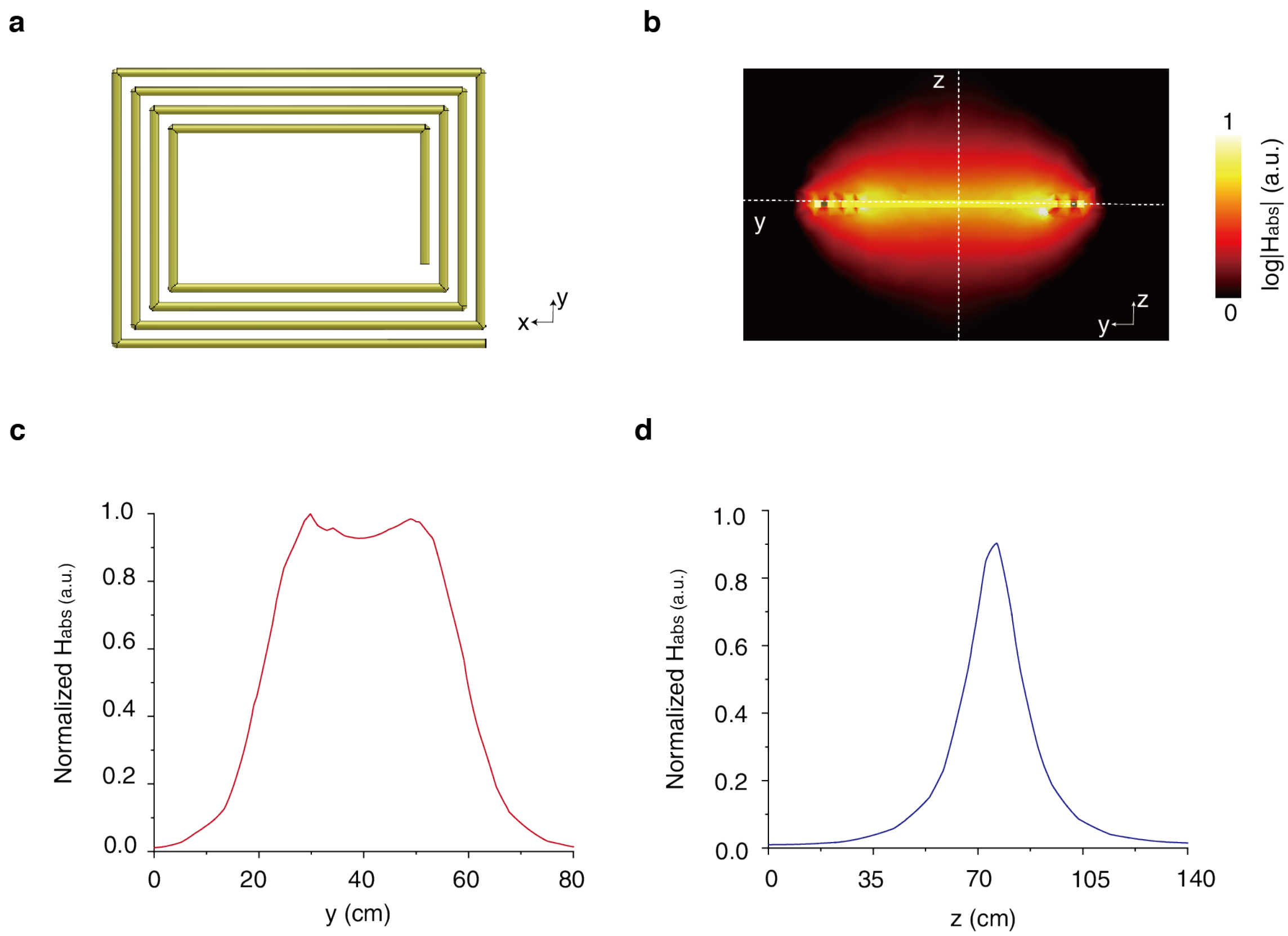

Supplementary Figure 4. **Magnetic field distribution emitted by a coil.** (**a**) Simulation model of a coil. (**b**) Magnetic field distribution emitted by the coil. (**c,d**) The variation in the magnitude of the normalized magnetic field along the y-axis and z-axis.

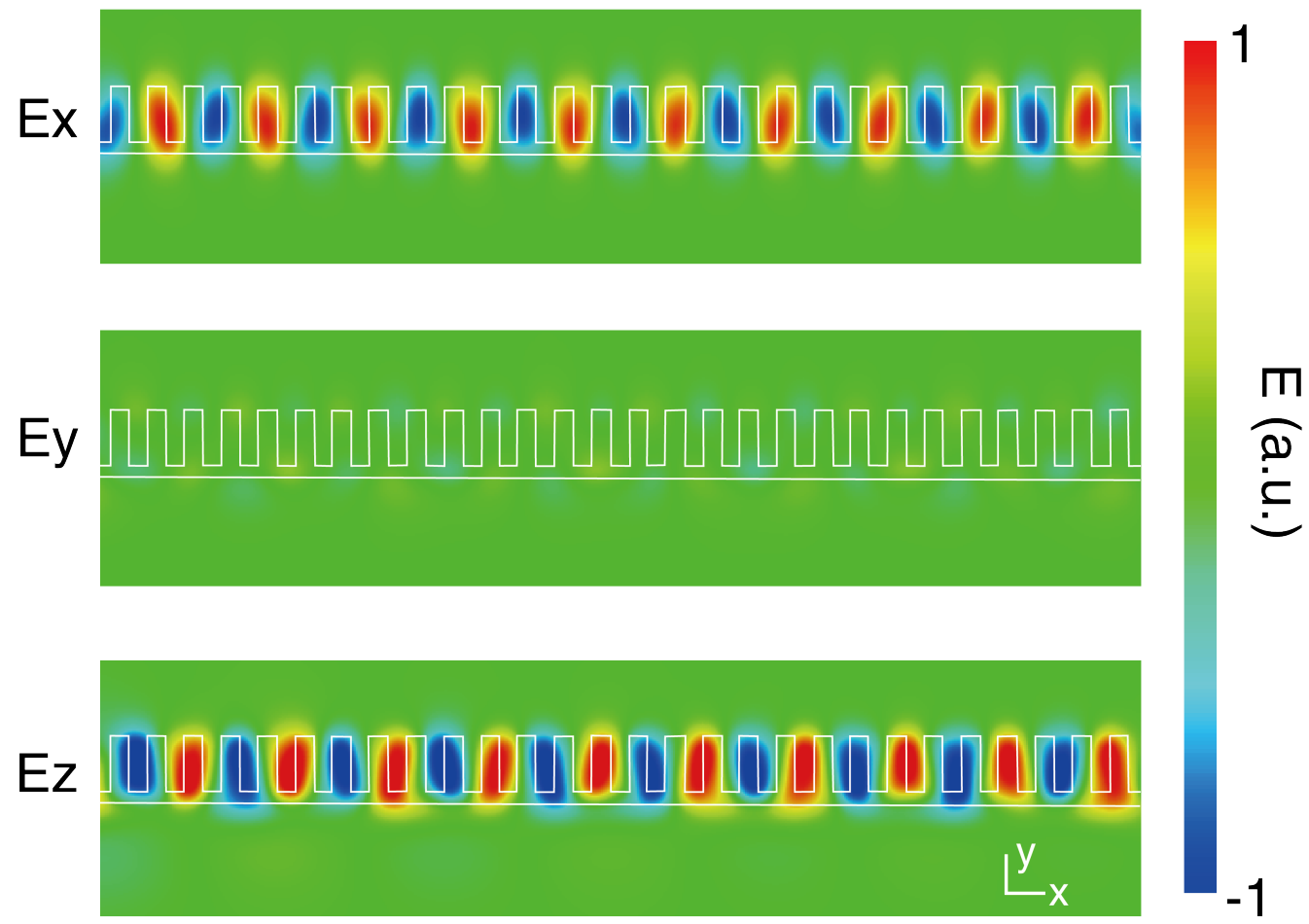

Supplementary Figure 5. **Electric field of the mode in the xy plane at 2.4 GHz.** Full-wave simulations of the electric field (xy plane) at 2.4 GHz for the SSP metamaterial textiles, the electric field expressions indicate that the SSP modes exhibit transverse magnetic characteristics and are highly confined near the structure.

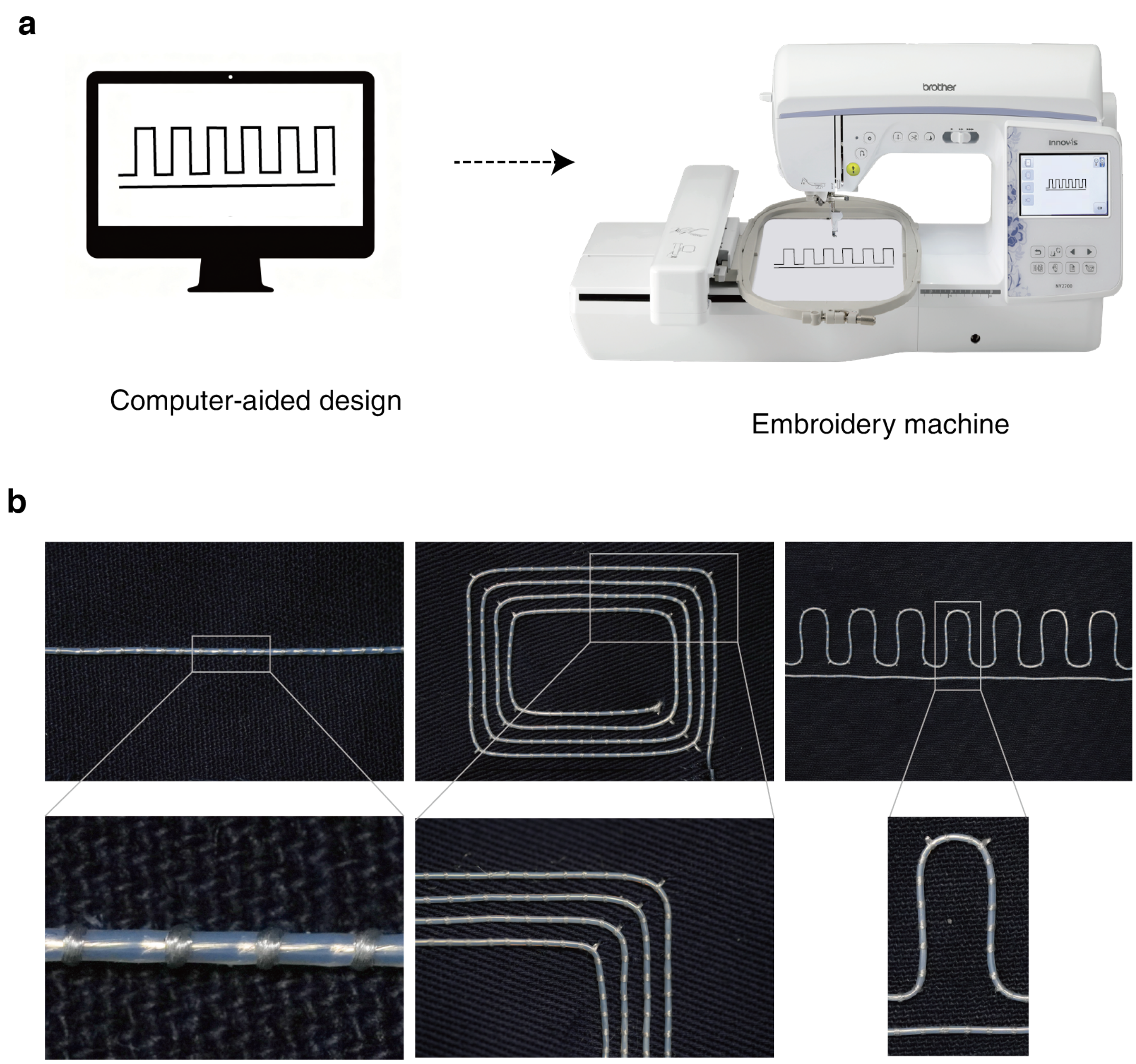

Supplementary Figure 6. **Illustration of the metamaterial textile fabrication process.** (**a**) Illustration of the digital embroidery fabrication process. (**b**) An image of the line and a detailed image of the local area of line, an image of the coil and a detailed image of the local area of coil, an image of the SSP and a detailed image of the local area of SSP.

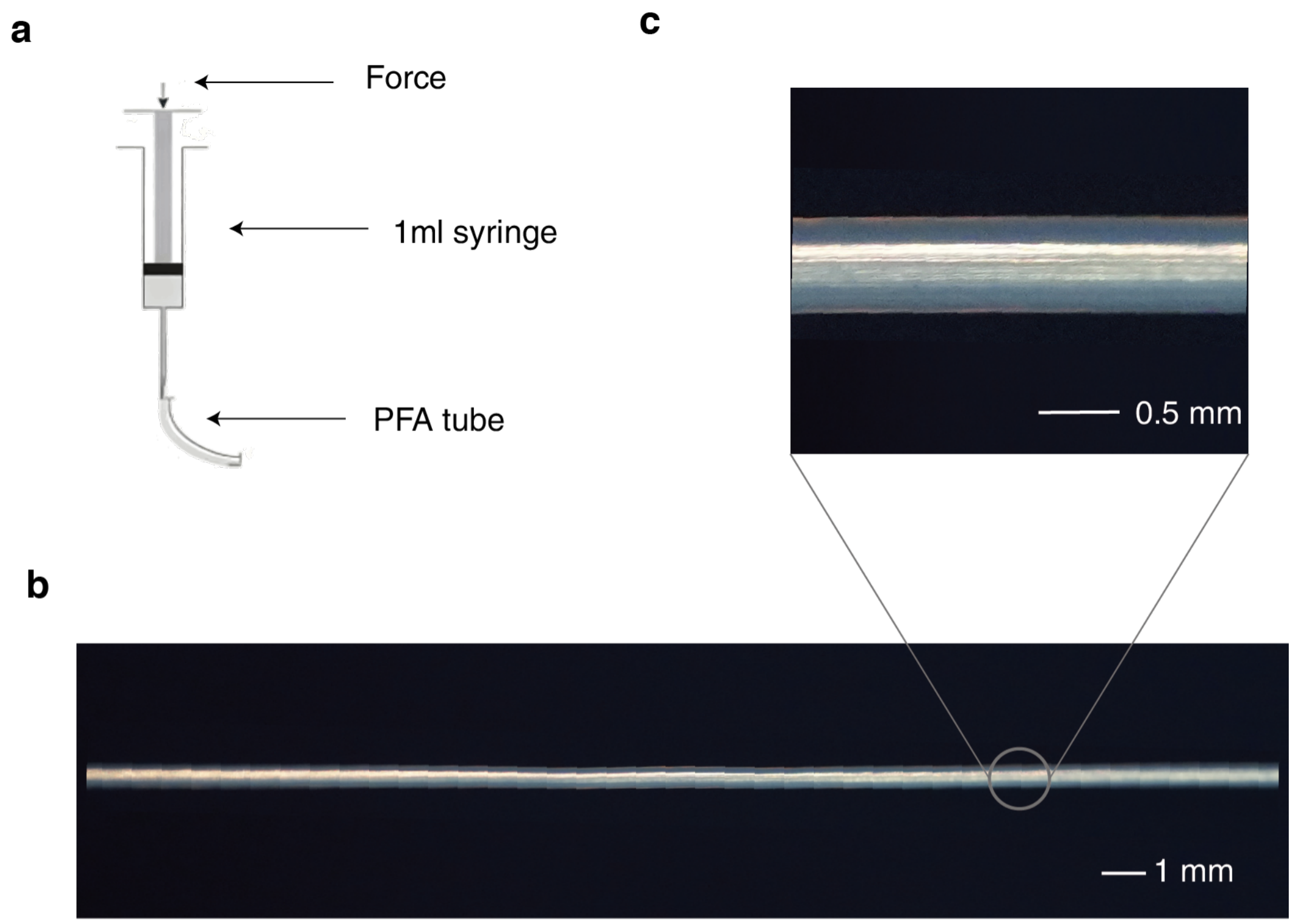

Supplementary Figure 7. **Fabrication process of Liquid Metal Fibers.** (**a**) The liquid metal fibers are fabricated by injecting gallium-tin compound through a syringe into a perfluoroalkoxyane tubing. (**b,c**) An image of the liquid metal fiber and the detailed image of the local area of liquid metal fibers.

a b

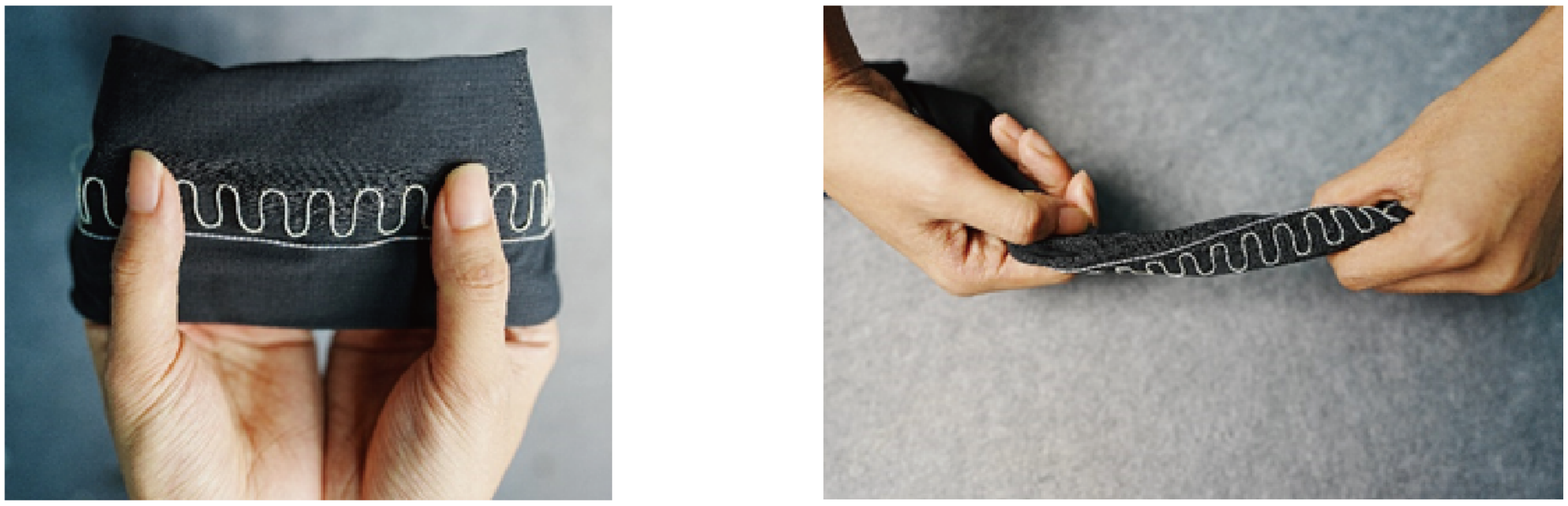

Supplementary Figure 8. **Images of embroidered dual-mode metamaterial textile subjected to pristine and twisting.** (**a,b**) The images are for demonstrating the stretchability and mechanical properties of the metamaterial textile.

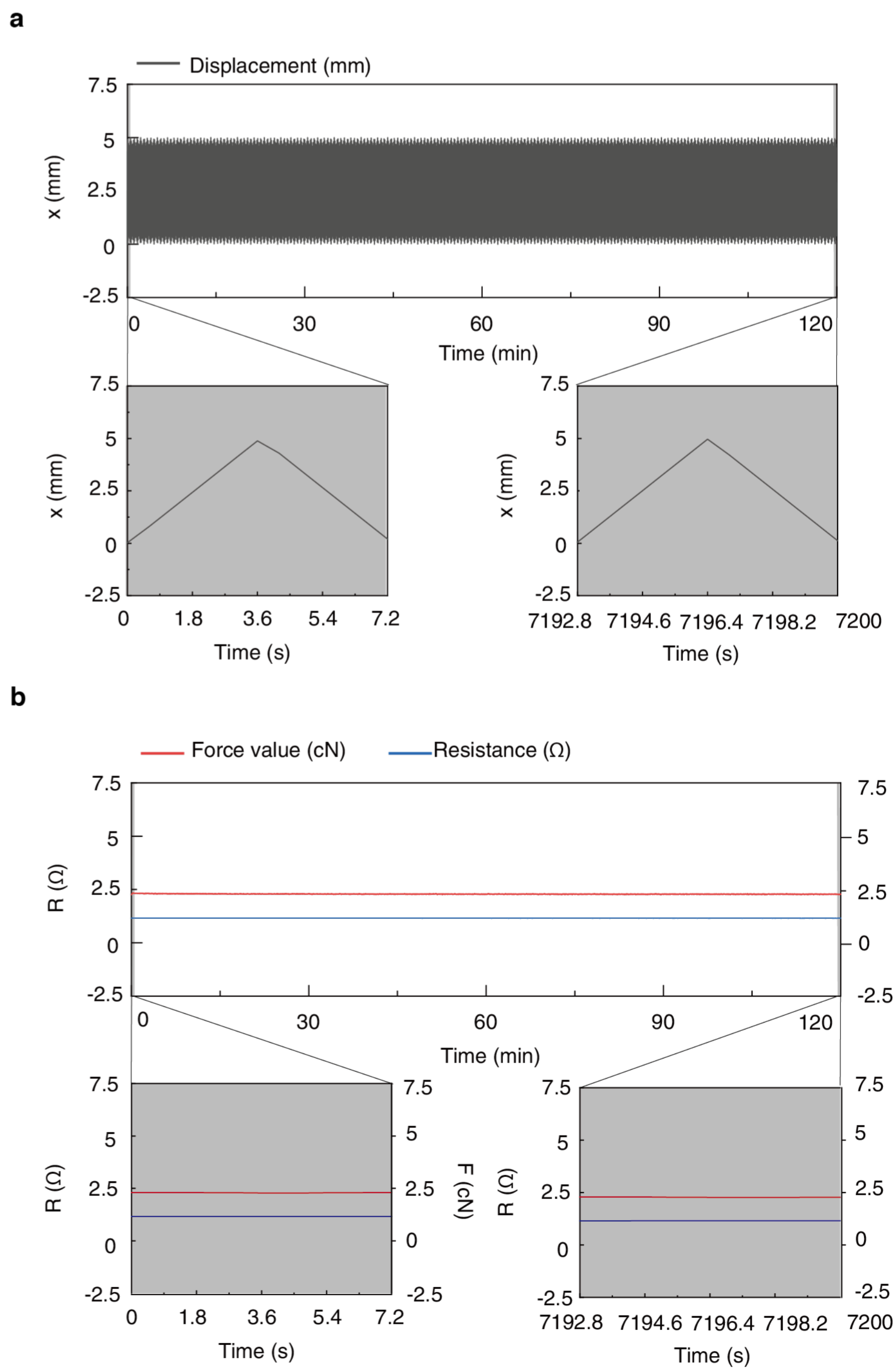

Supplementary Figure 9. **Resistance of the liquid metal fiber as a function of number of stretching time.** (**a**) The variation of displacement over time. (**b**) The variations of force and resistance over time.

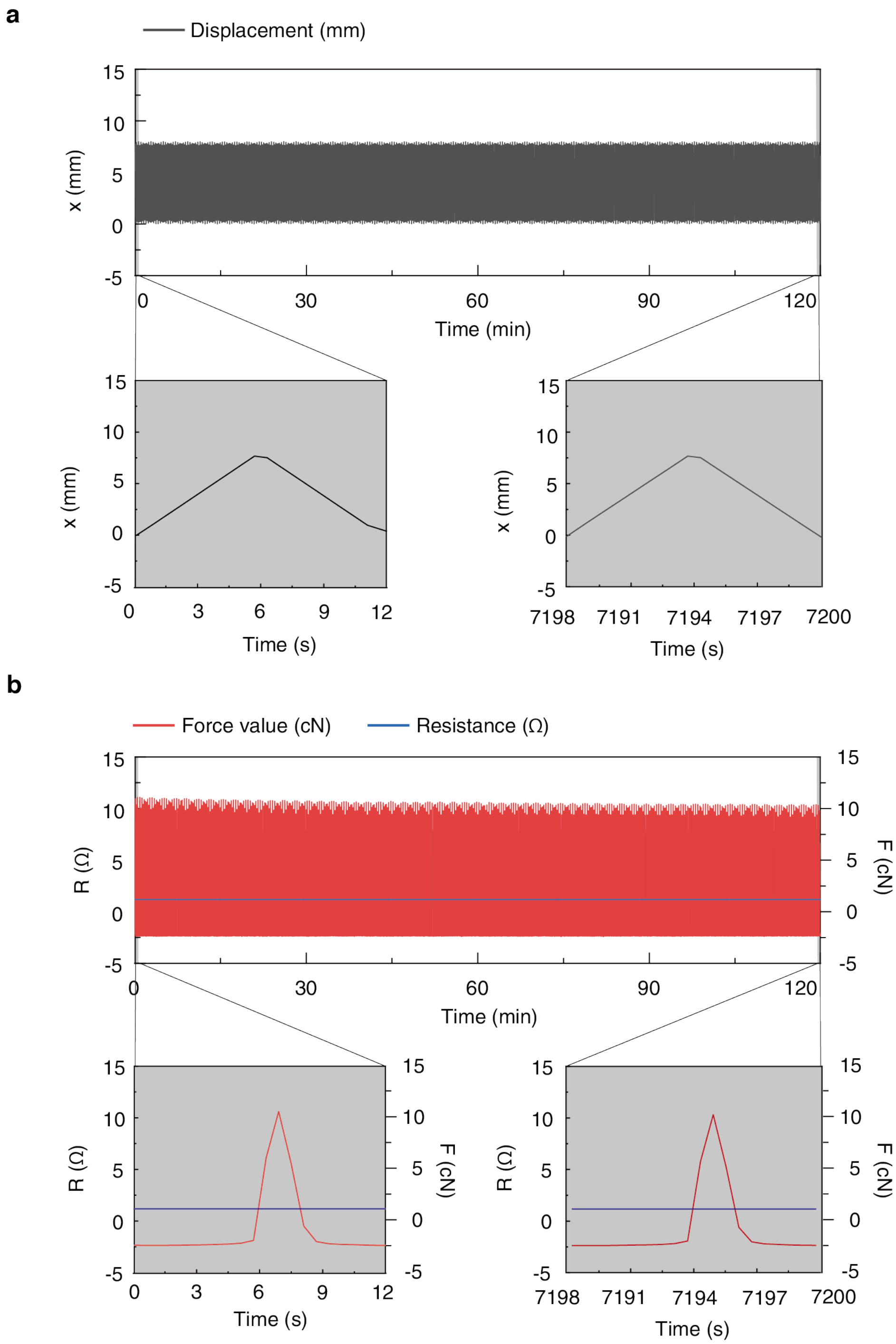

Supplementary Figure 10. **Resistance of the liquid metal fiber as a function of number of compression time.** (**a**) The variation of displacement over time. (**b**) The variations of force and resistance over time.

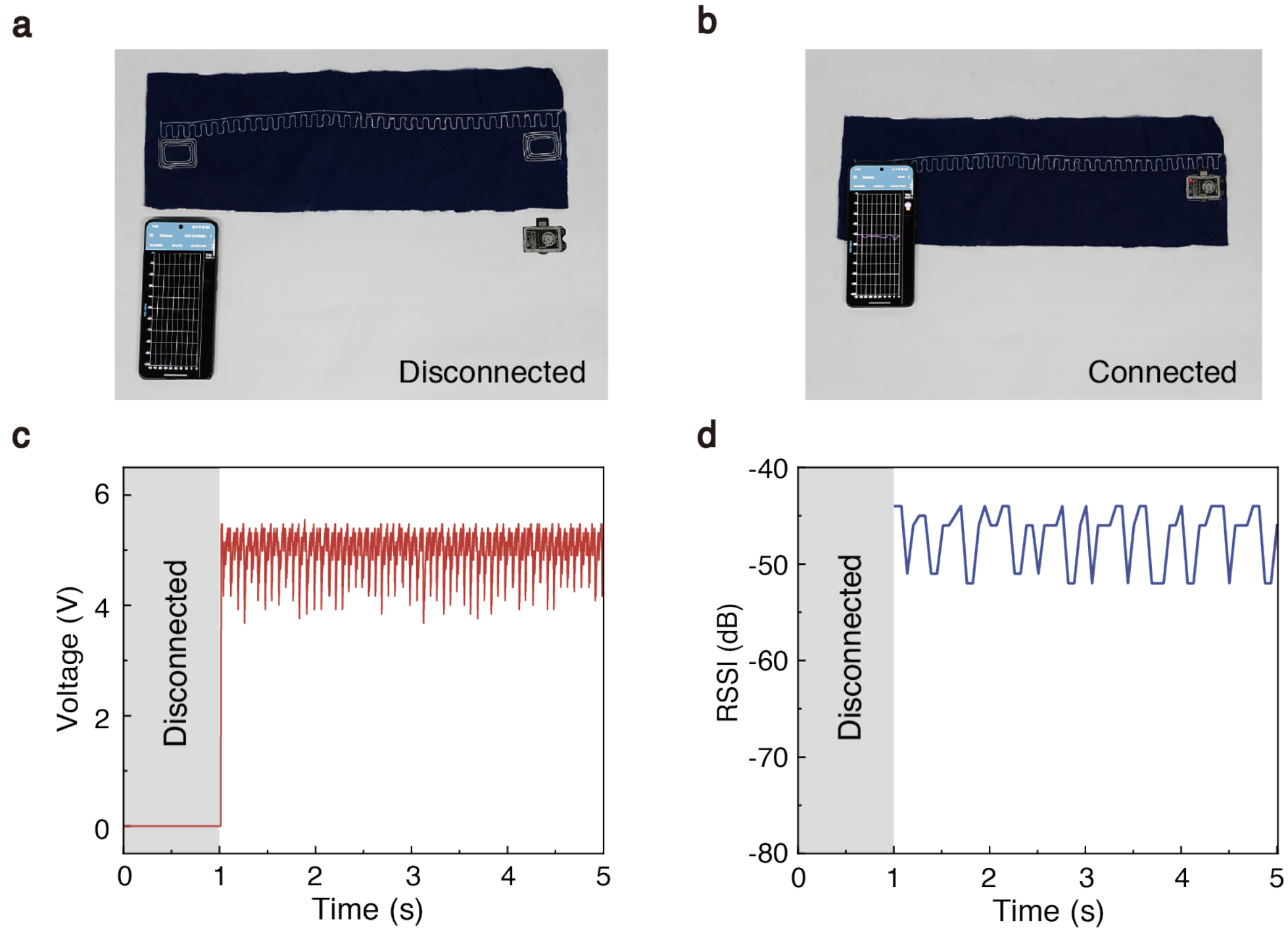

Supplementary Figure 11. **Wireless powering and communication of battery-free sensors enabled by the metamaterial textile.** (**a**) Photograph of the metamaterial textile, smartphone and sensor node which isn't powered. (**b**) Photograph of the metamaterial textile enabled powering and communication between the smartphone and sensor node. (**c**) The rectified voltage of the powered sensor node, when the wireless powering pathway is established by the metamaterial textile between the smartphone and sensor node. (**d**) The received signal strength indicator (RSSI) of the sensor node, when the wireless communication is established by the metamaterial textile between the smartphone and sensor node.

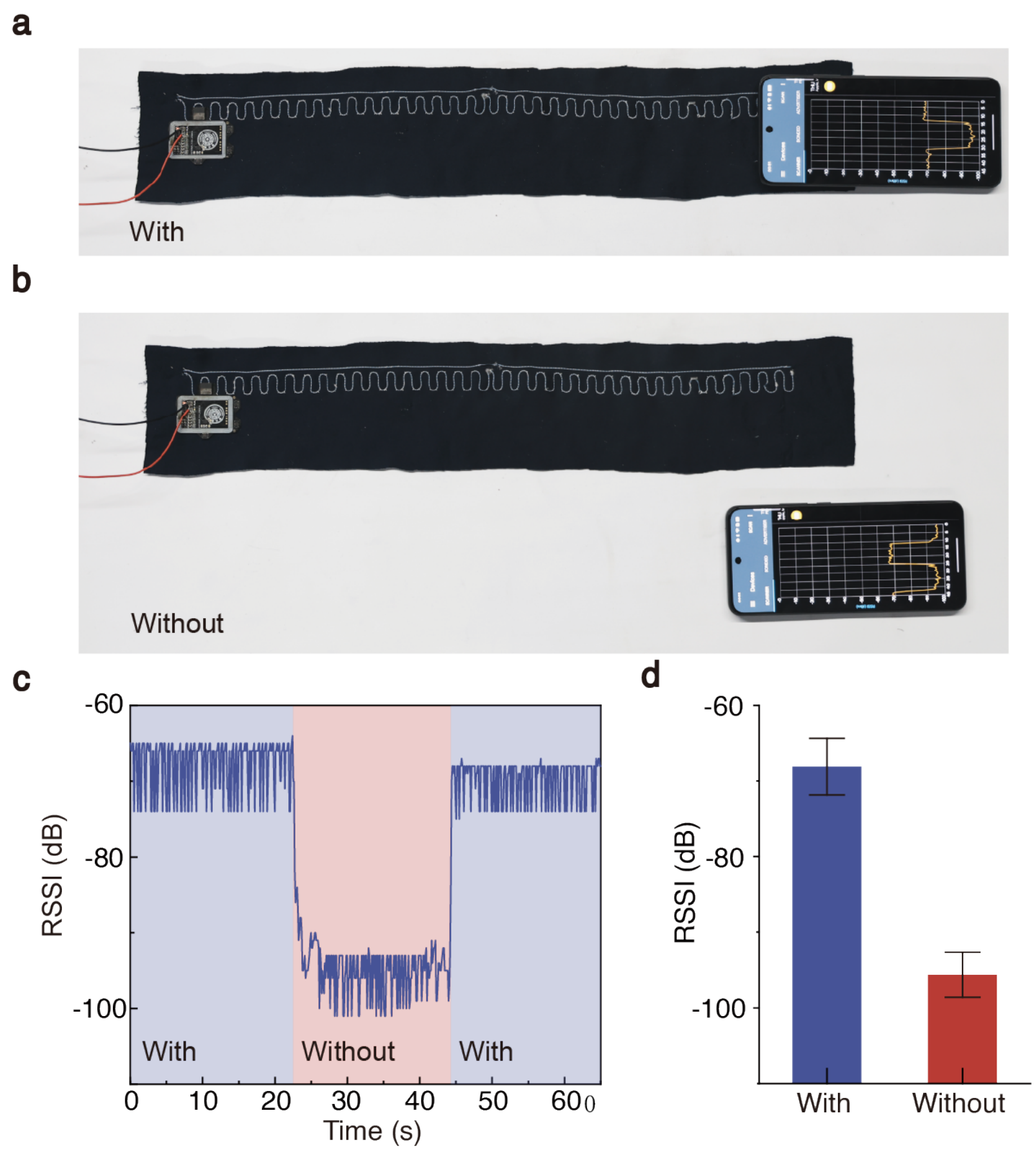

Supplementary Figure 12. **Wireless communication enhancement of metamaterial textile.** (**a**) Photograph of wireless signal transmission using metamaterial textile. (**b**) Photograph of wireless signal transmission without using metamaterial textile. (**c**) Variation in RSSI of the wireless signal with and without the metamaterial textile. (**d**) The metamaterial textile provides a wireless signal gain of approximately 28 dB. Error bars show mean ± s.d. of RSSI values calculated across the indicated period.

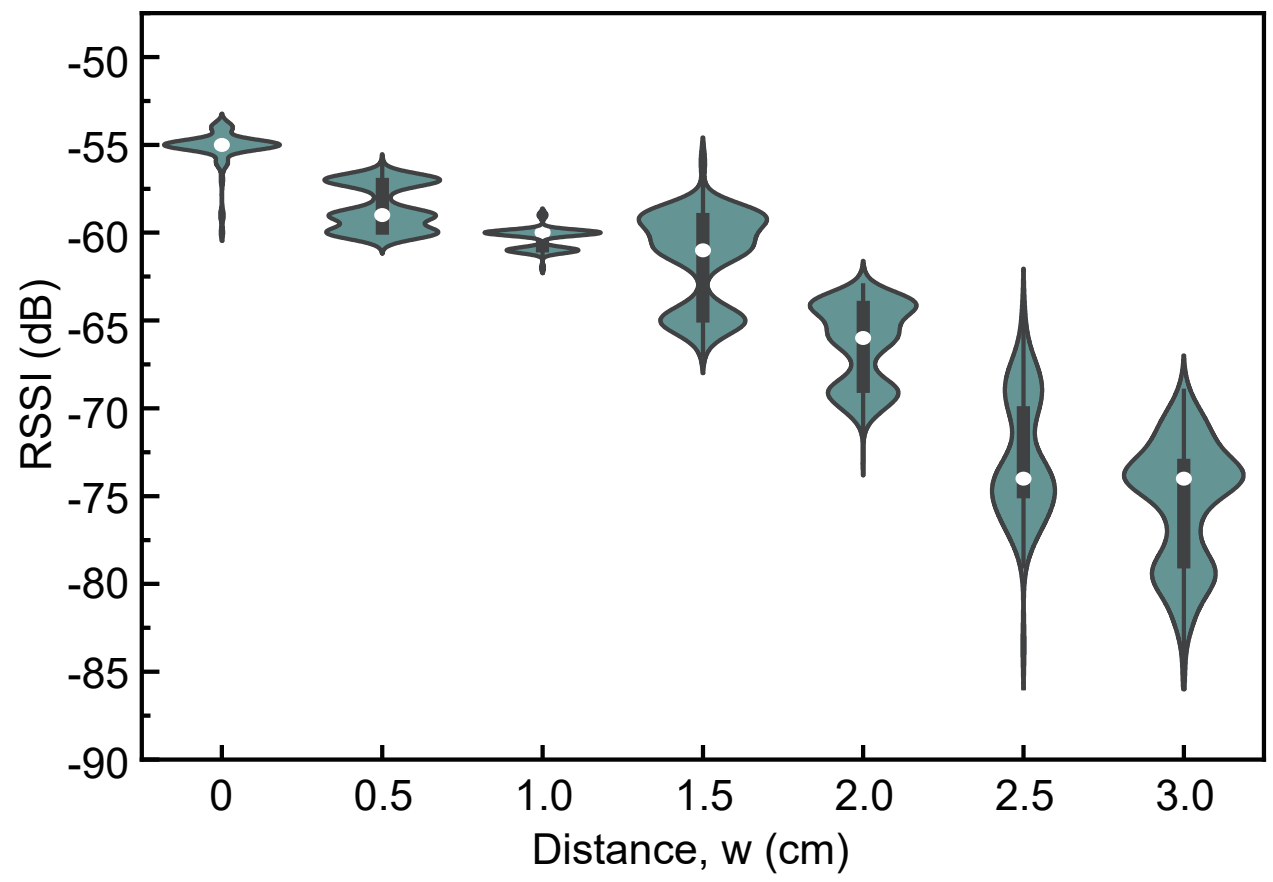

Supplementary Figure 13. **Phone position tolerance of the metamaterial textile.** The phone was placed on the SSP, and the RSSI of a single node was recorded for 1 min at each position as the phone was incrementally displaced perpendicular to the SSP structure (i.e., increasing w). The position defined as w = 0 corresponds to the location yielding the maximum RSSI, representing the optimal coupling condition. The box plots inside the violins indicate the quartiles of corresponding RSSI measurements. The white dots represent median values. The whiskers denote 1.5 times the interquartile range.

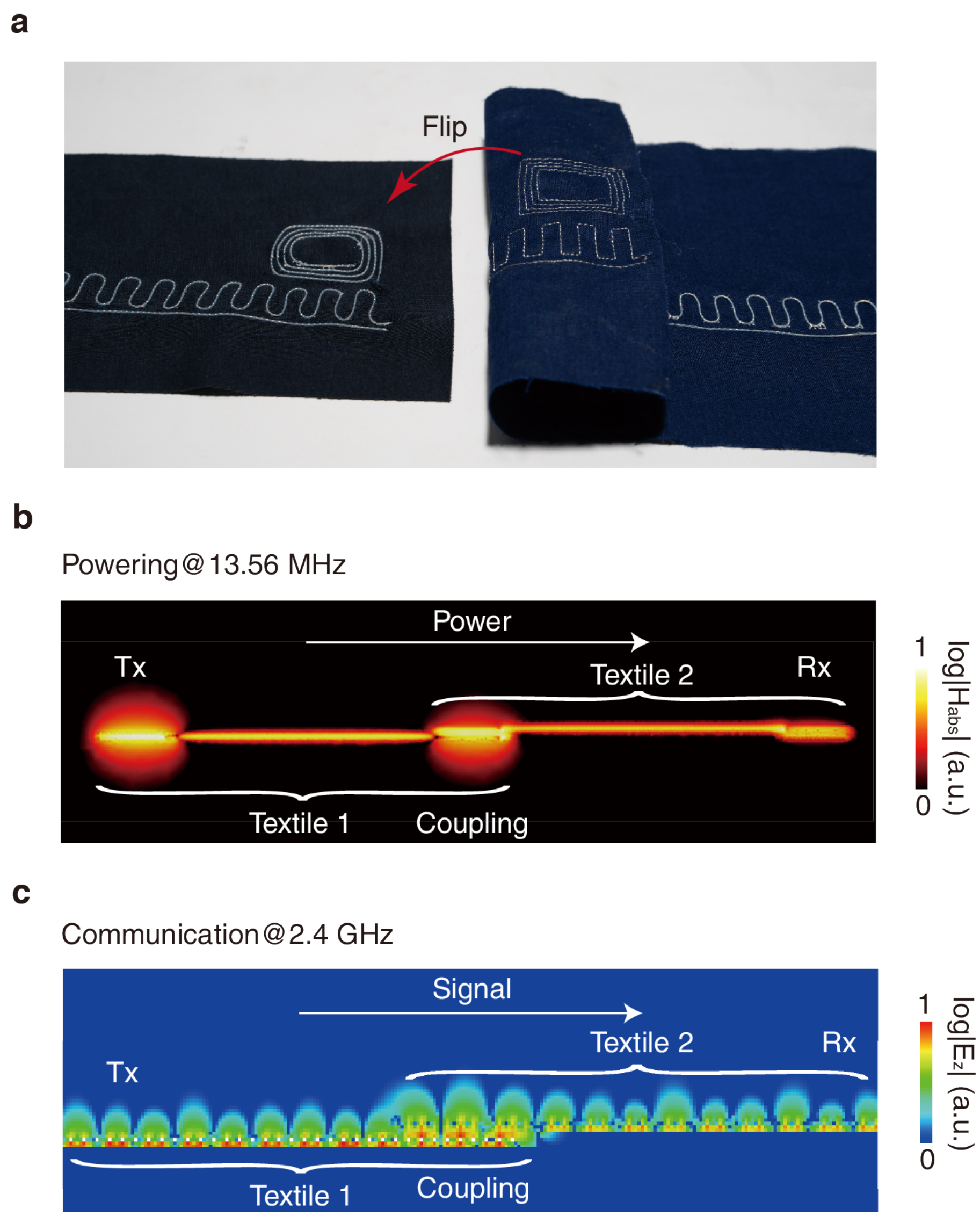

Supplementary Figure 14. **The simulation of wireless powering and data transmission across a gap between two metamaterial textiles.** (**a**) Illustration of two metamaterial textiles with a gap.(**b**) Illustration of wireless powering across a gap between two metamaterial textiles.(**c**) Illustration of wireless communication across a gap between two metamaterial textiles.

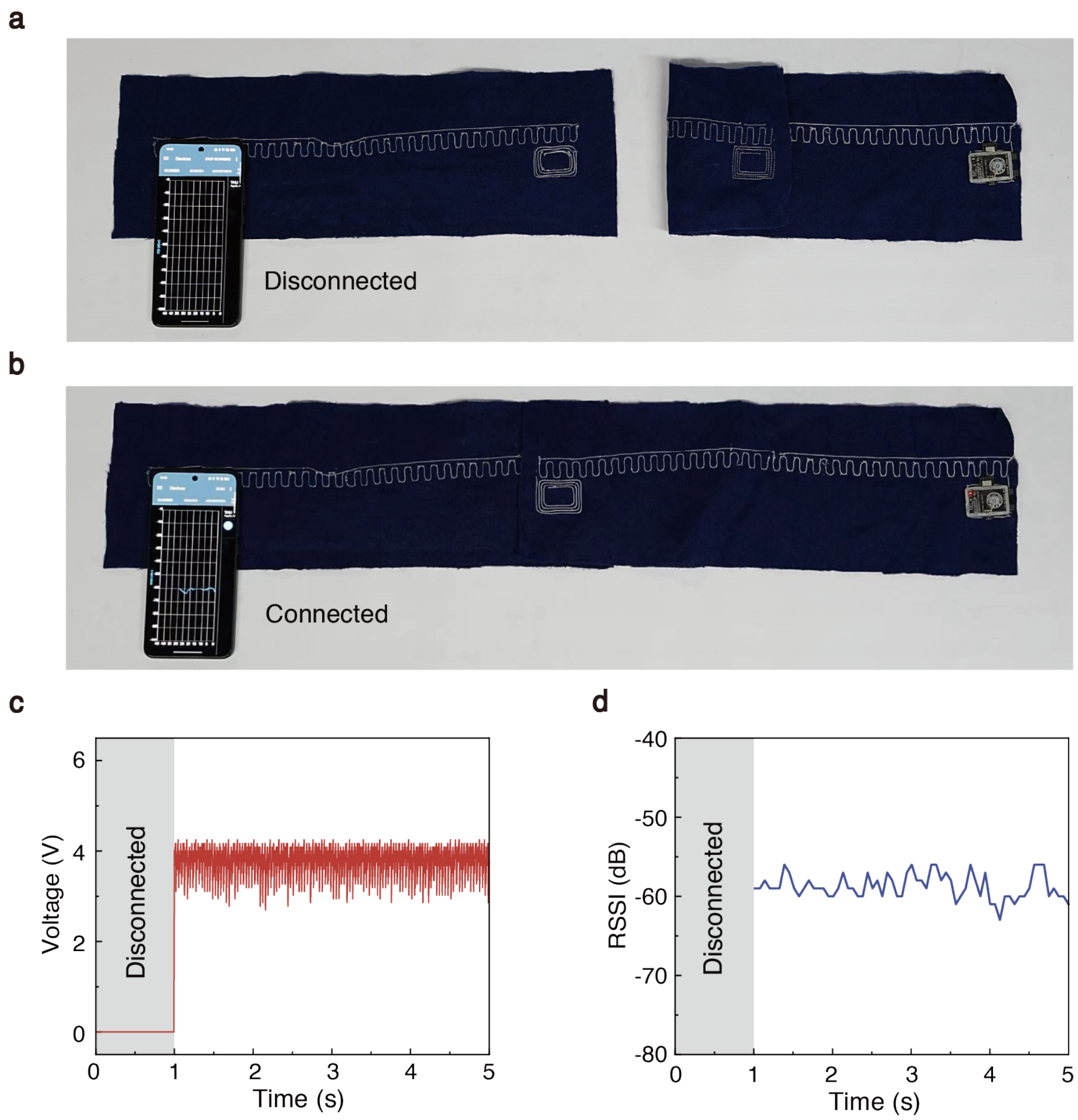

Supplementary Figure 15. **The establishment of wireless powering and communication across a gap between two metamaterial textiles.** (**a**) Photograph of two metamaterial textiles, smartphone and sensor node which isn't powered. (**b**) Photograph of two metamaterial textiles enabled powering and communication between the smartphone and sensor node. (**c**) The rectified voltage of the powered sensor node, when the wireless powering pathway is established by two metamaterial textiles between the smartphone and sensor node. (**d**) The received signal strength indicator (RSSI) of the sensor node, when the wireless communication is established by two metamaterial textiles between the smartphone and sensor node.

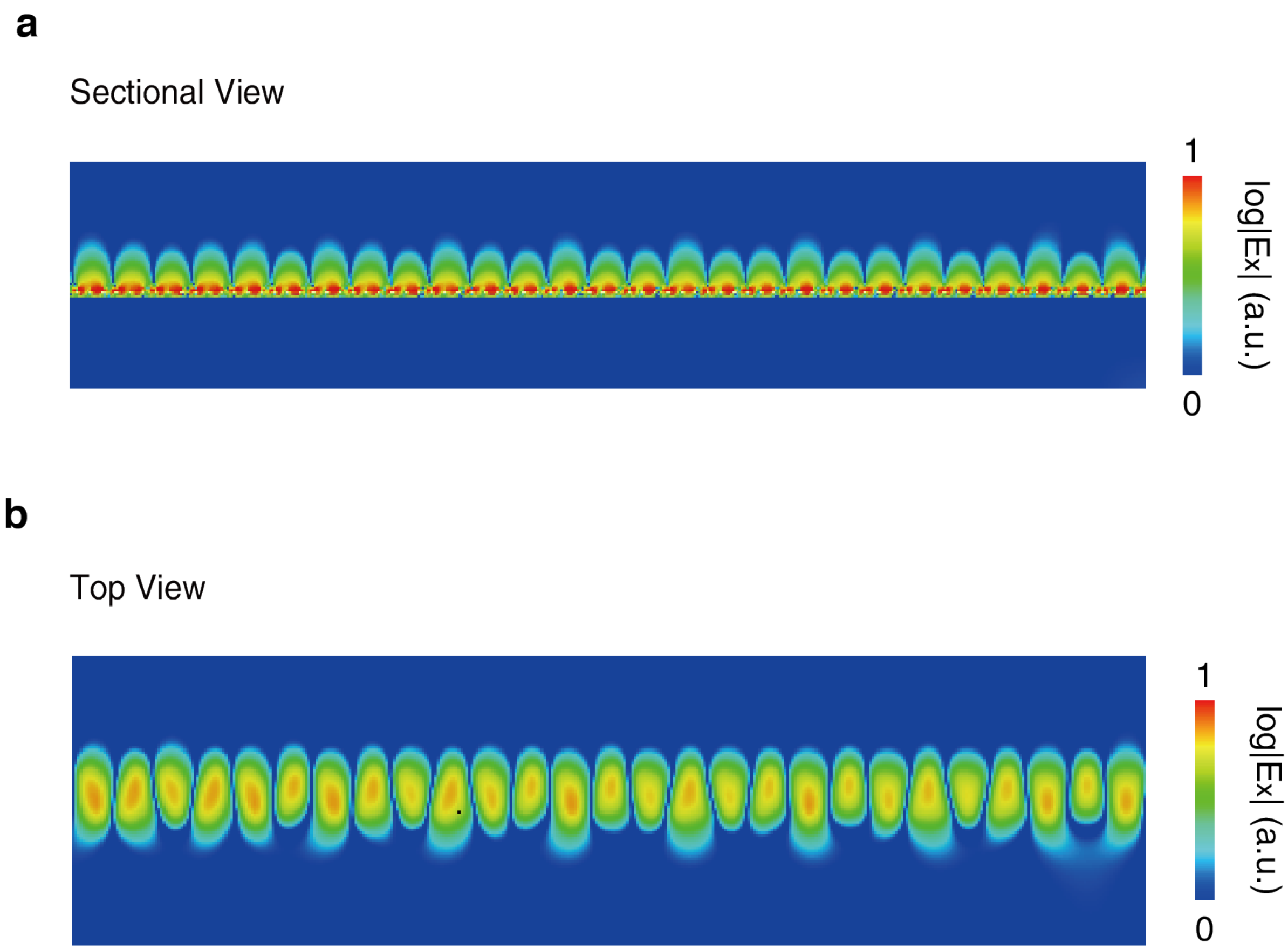

Supplementary Figure 16. **The electric field distribution of SSP.** (**a,b**) Full-wave simulations of the electric field (xy plane and xz plane) at 2.4 GHz for the SSP metamaterial textiles, the electric field is transmitted in the form of surface waves and exhibits highly localized characteristics.

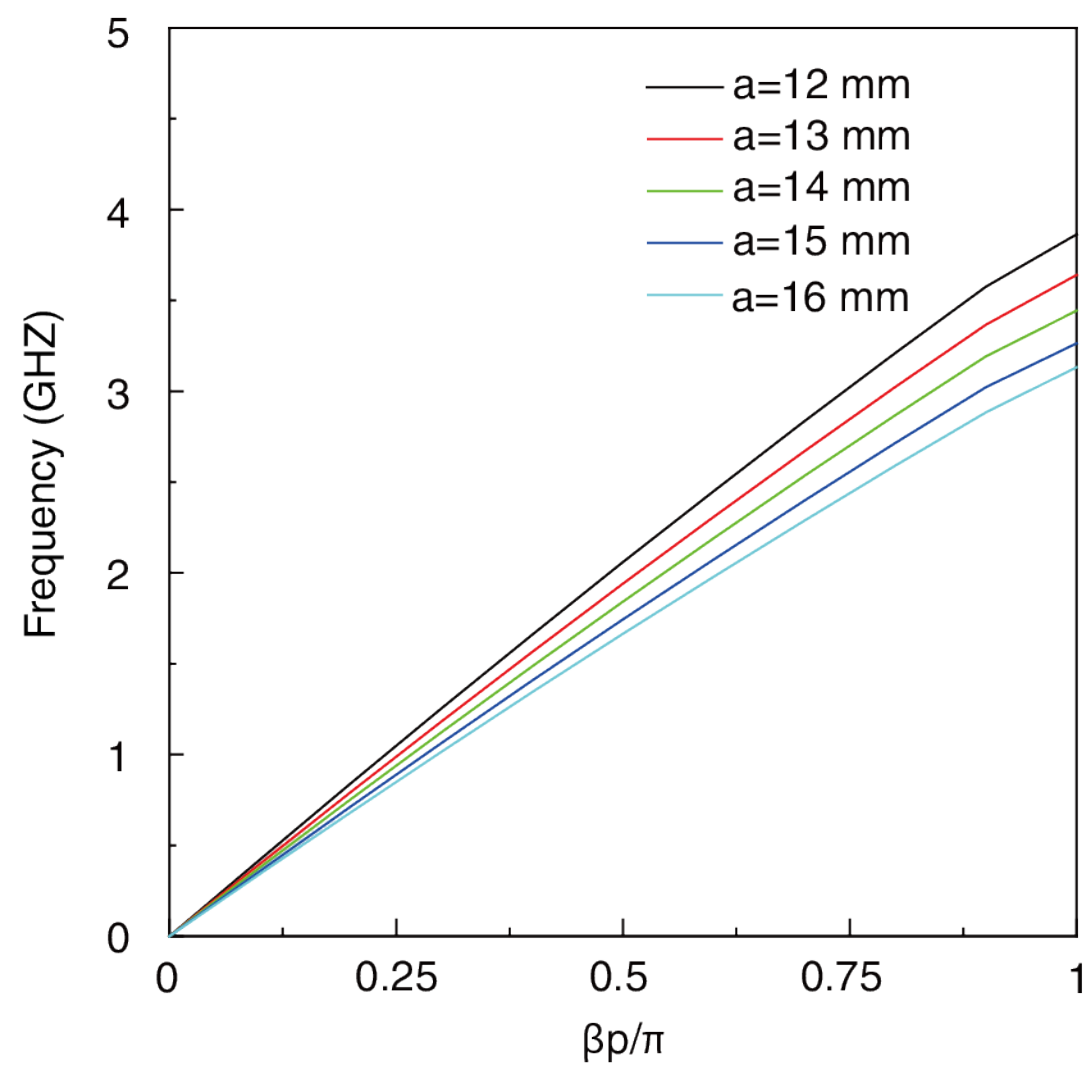

Supplementary Figure 17. **The dispersion relation of SSP.** Dispersion characteristics of the single unit cell of the SSP metamaterial textiles, as a increases, the wavenumber rises at the same frequence, which indicates a decrease in wavelength and enhanced compressibility of the field

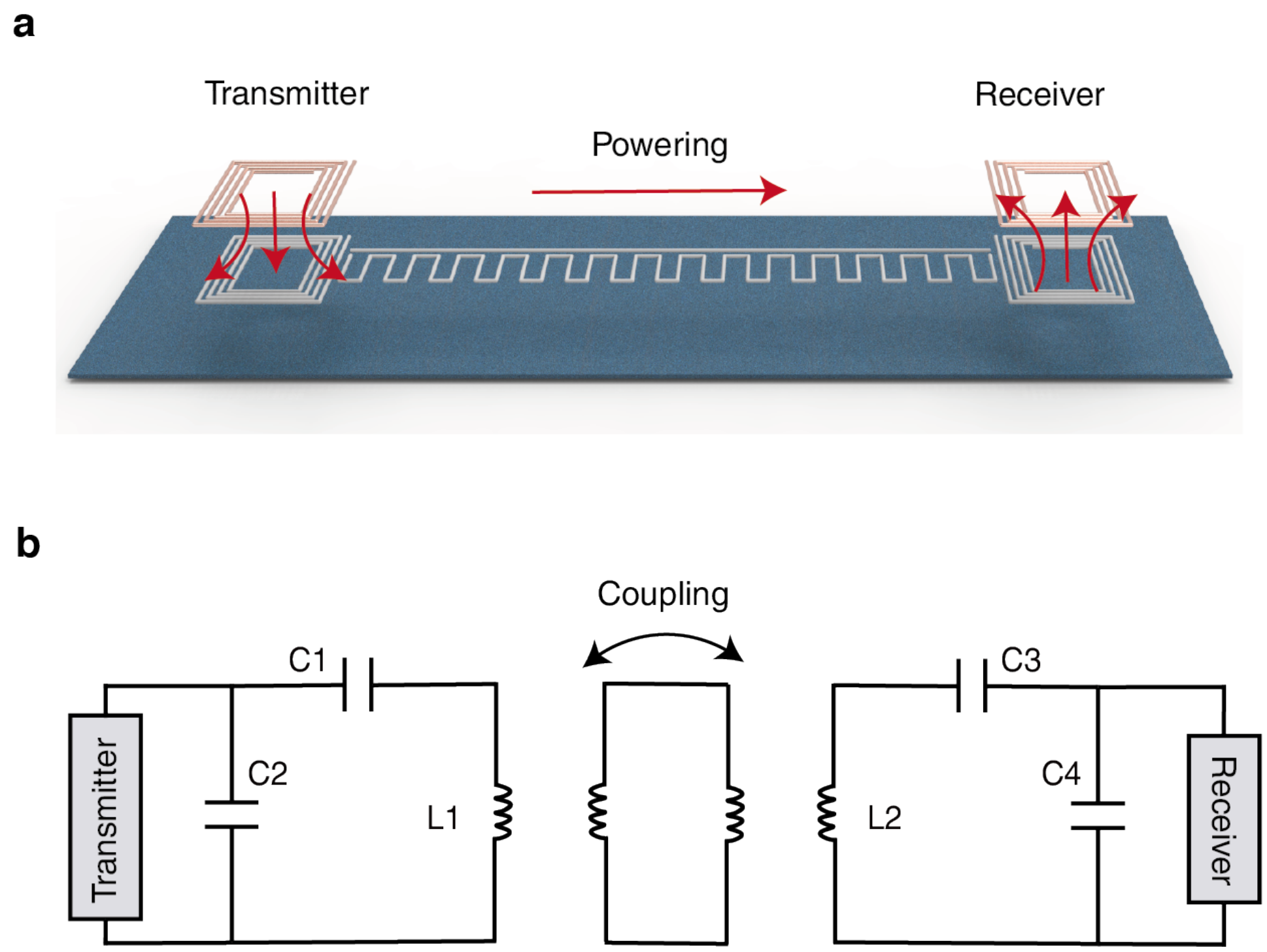

Supplementary Figure 18. **The power supply principle of two-port wireless network.** (**a**) Schematic diagram of the powering principle of the two-port network structure. (**b**) Circuit model of the wireless network system in a.

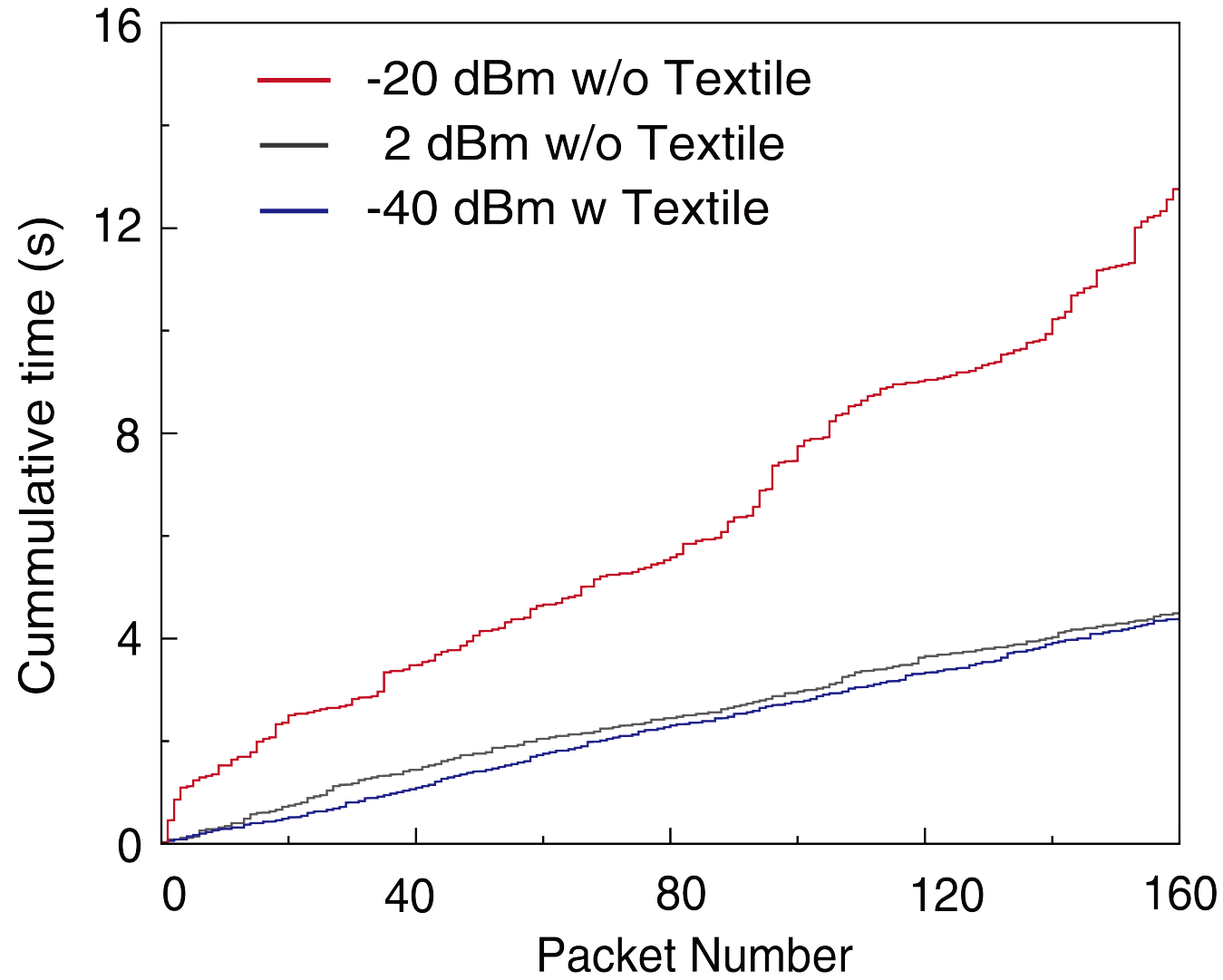

Supplementary Figure 19. **Energy-efficient wireless communication.** Cumulative transmission time as a function of number of packets, with the metamaterial textile, the high data transmission is established under a lower energy.

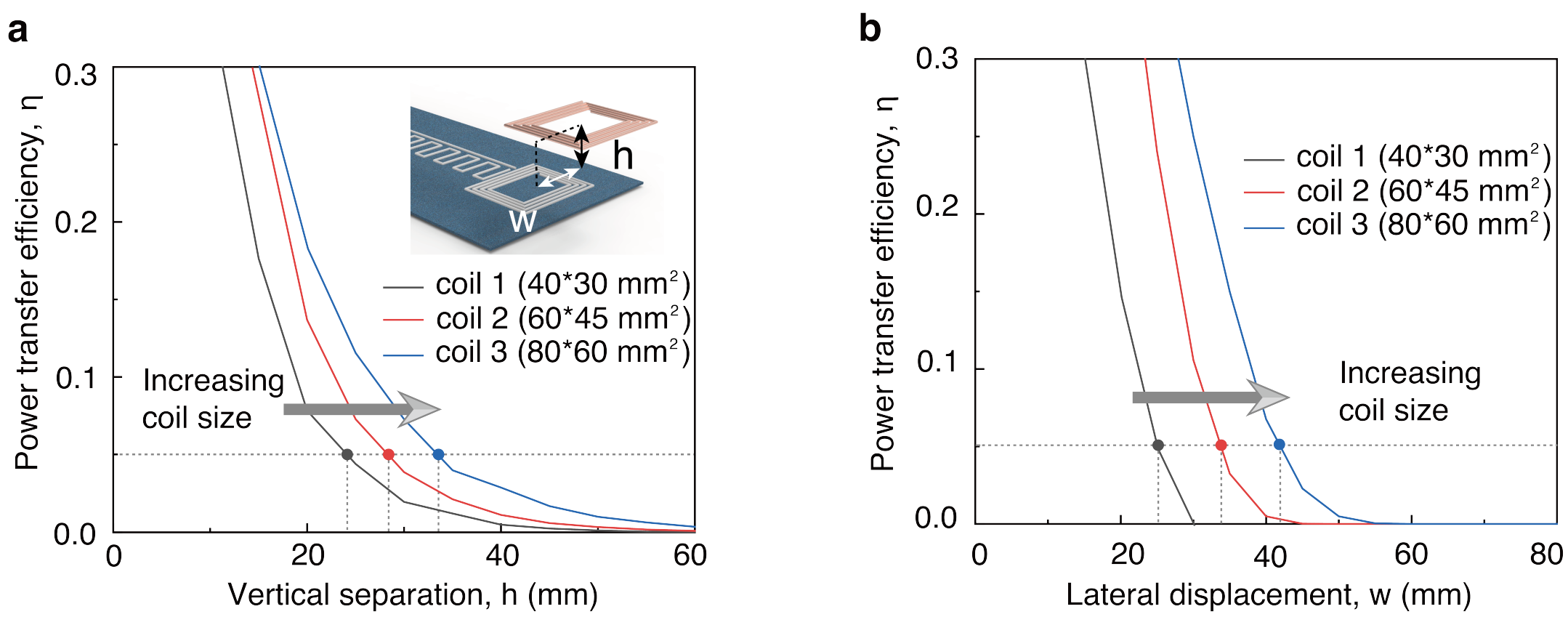

Supplementary Figure 20. **Design tunability for motion robustness.** (**a-b**) Electromagnetic simulations showing that the maximum powering distance can be increased by tuning coil geometry.

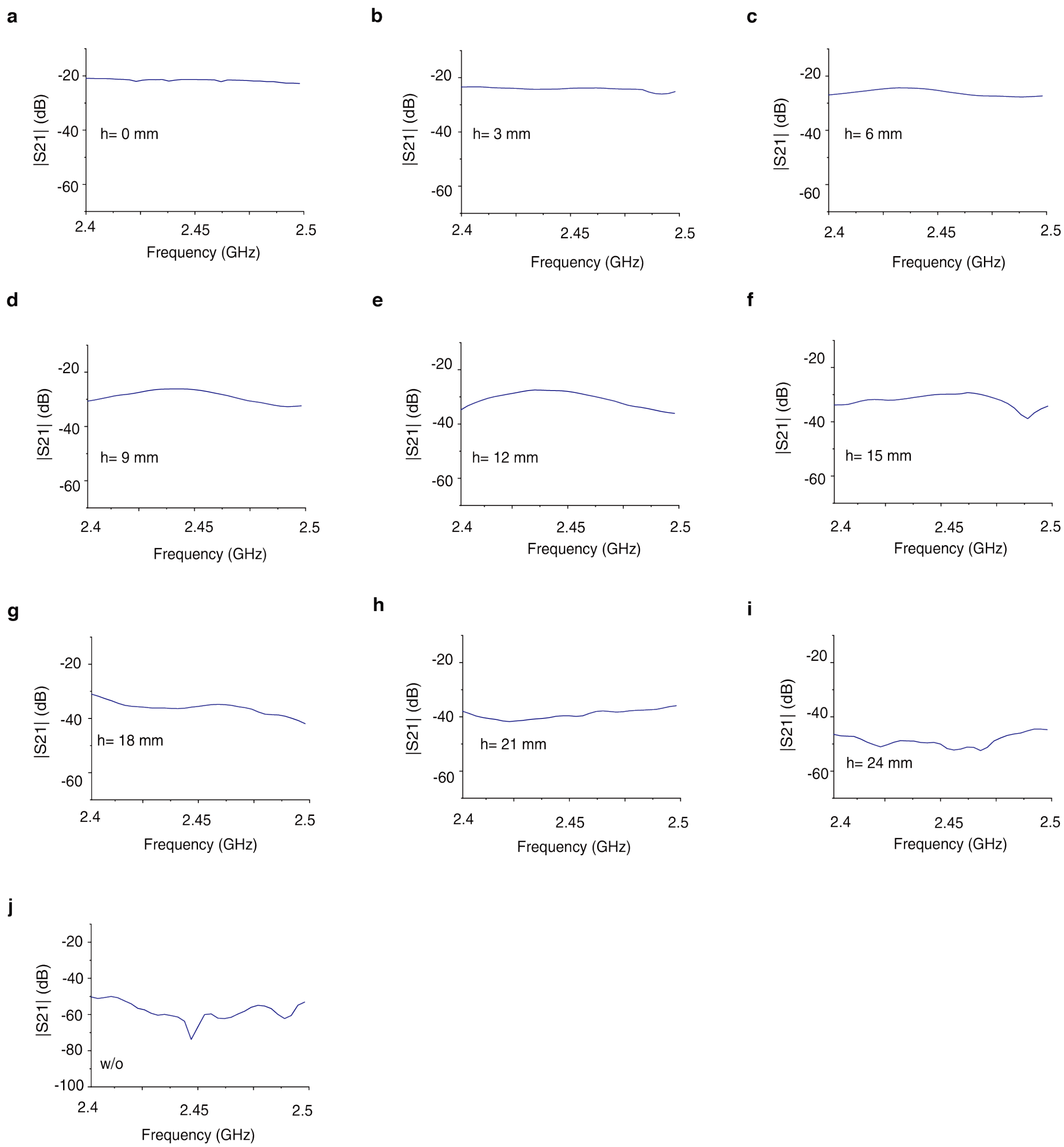

Supplementary Figure 21. **Comparison of transmission spectrums for the metamaterial textile with different vertical separations.** (**a-j**) Transmission spectrum for the metamaterial textile with different h from 2.4 GHz to 2.5 GHz and the transmission without metamaterial textile from 2.4 GHz to 2.5 GHz.

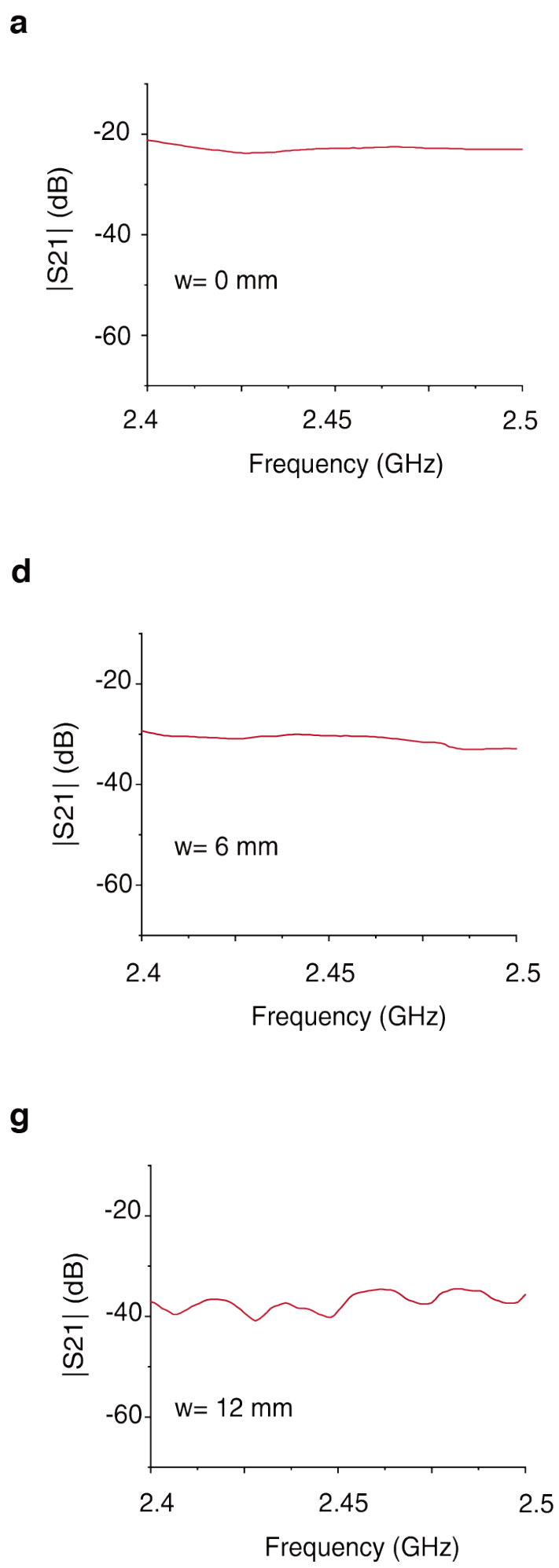

Supplementary Figure 22. **Comparison of transmission spectrums for the metamaterial textile with different displacement.** (**a-g**) Transmission spectrum for the metamaterial textile with different w from 2.4 GHz to 2.5 GHz and the transmission without metamaterial textile from 2.4 GHz to 2.5 GHz.

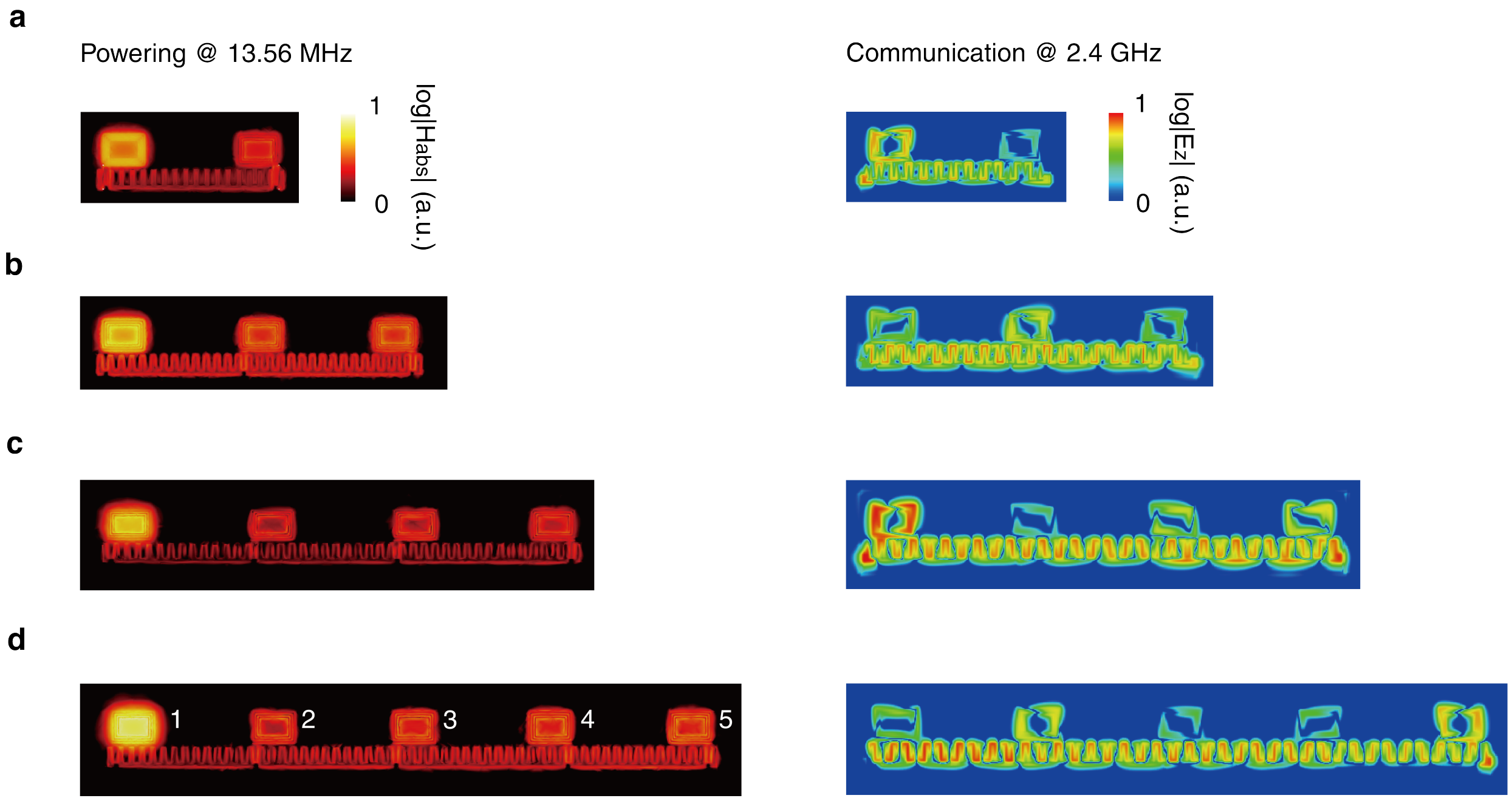

Supplementary Figure 23. **Elecric and magnetic field distribution of wireless networks in series.** (**a-d**) Electric fields in the z direction generated by a source placed on the node wirelessly interconnected to receivers at 2.45 GHz communication mode in series. Magnetic fields generated by a source placed on the node wirelessly interconnected to receivers at 13.56 MHz powering mode in series.

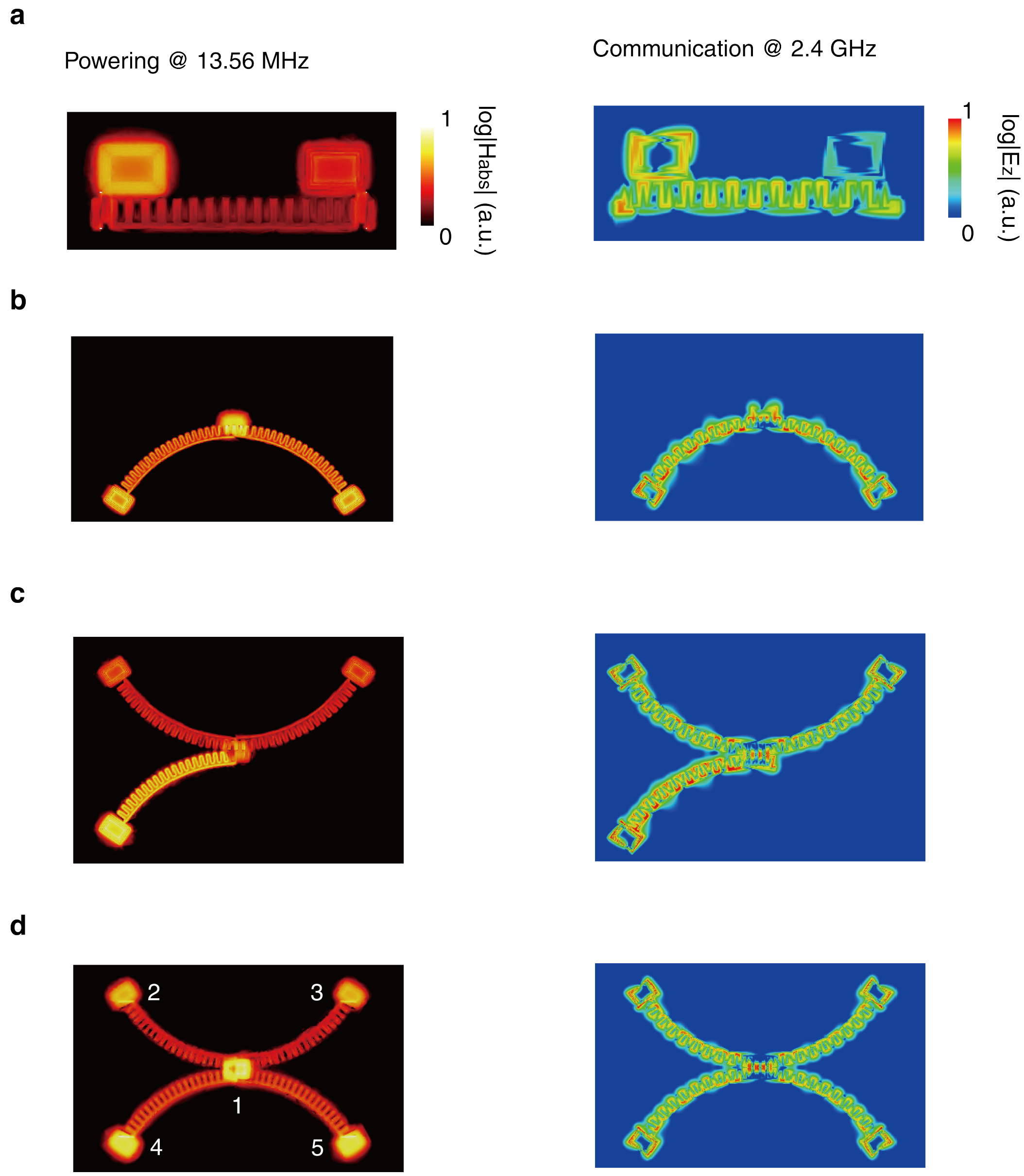

Supplementary Figure 24. **Electric and magnetic field distribution of wireless networks in parallel.** (**a-d**) Electric fields in the z direction generated by a source placed on the node wirelessly interconnected to receivers at 2.45 GHz communication mode in parallel. Magnetic fields generated by a source placed on the node wirelessly interconnected to receivers at 13.56 MHz powering mode in parallel.

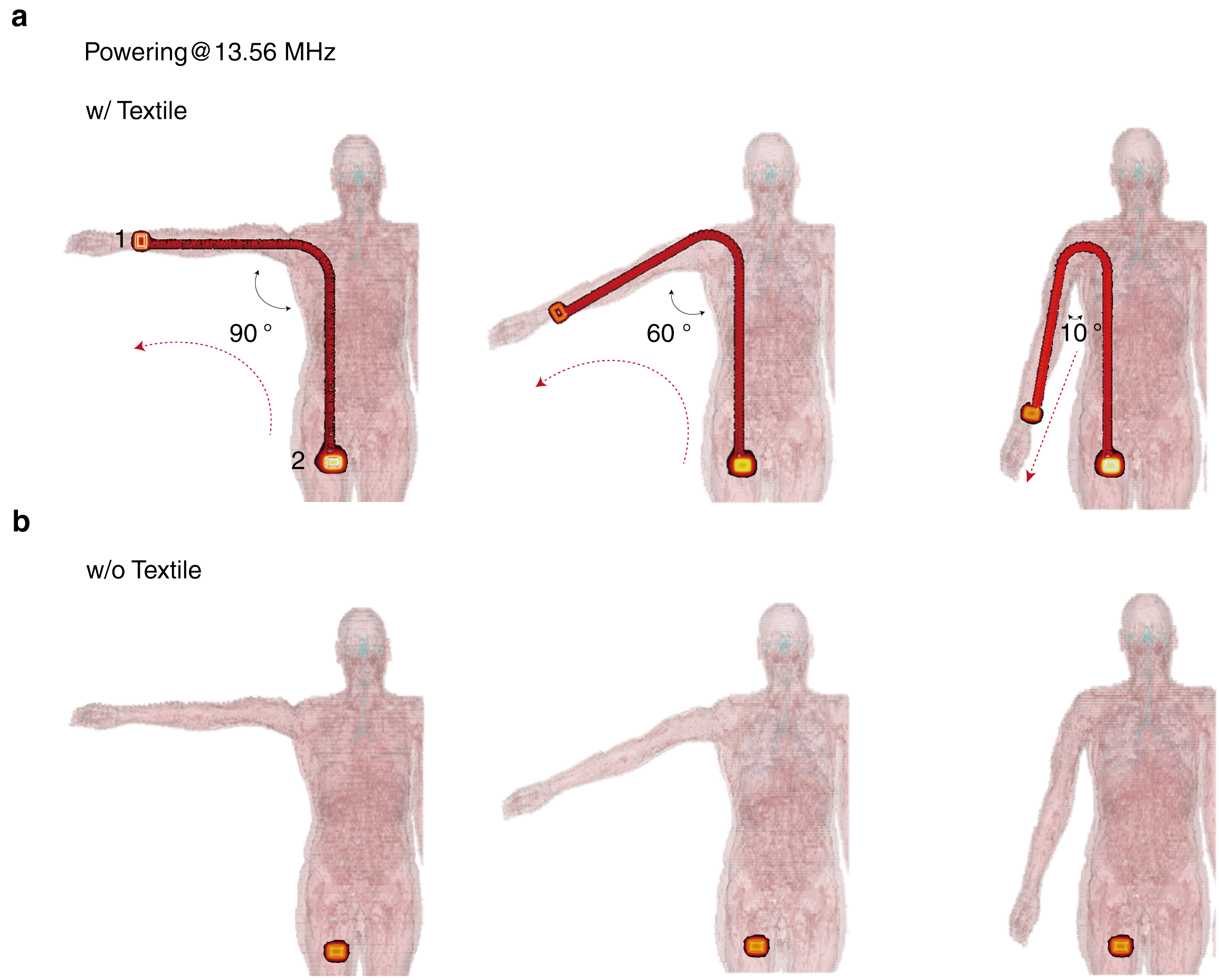

Supplementary Figure 25. **The robust wireless powering as the movement of the human body change.** (**a,b**) Wireless powering on the human body from the leg to the wrist (sensor node 1) with or without metamaterial textiles, the sensor node cannot be powered without metamaterial textiles.

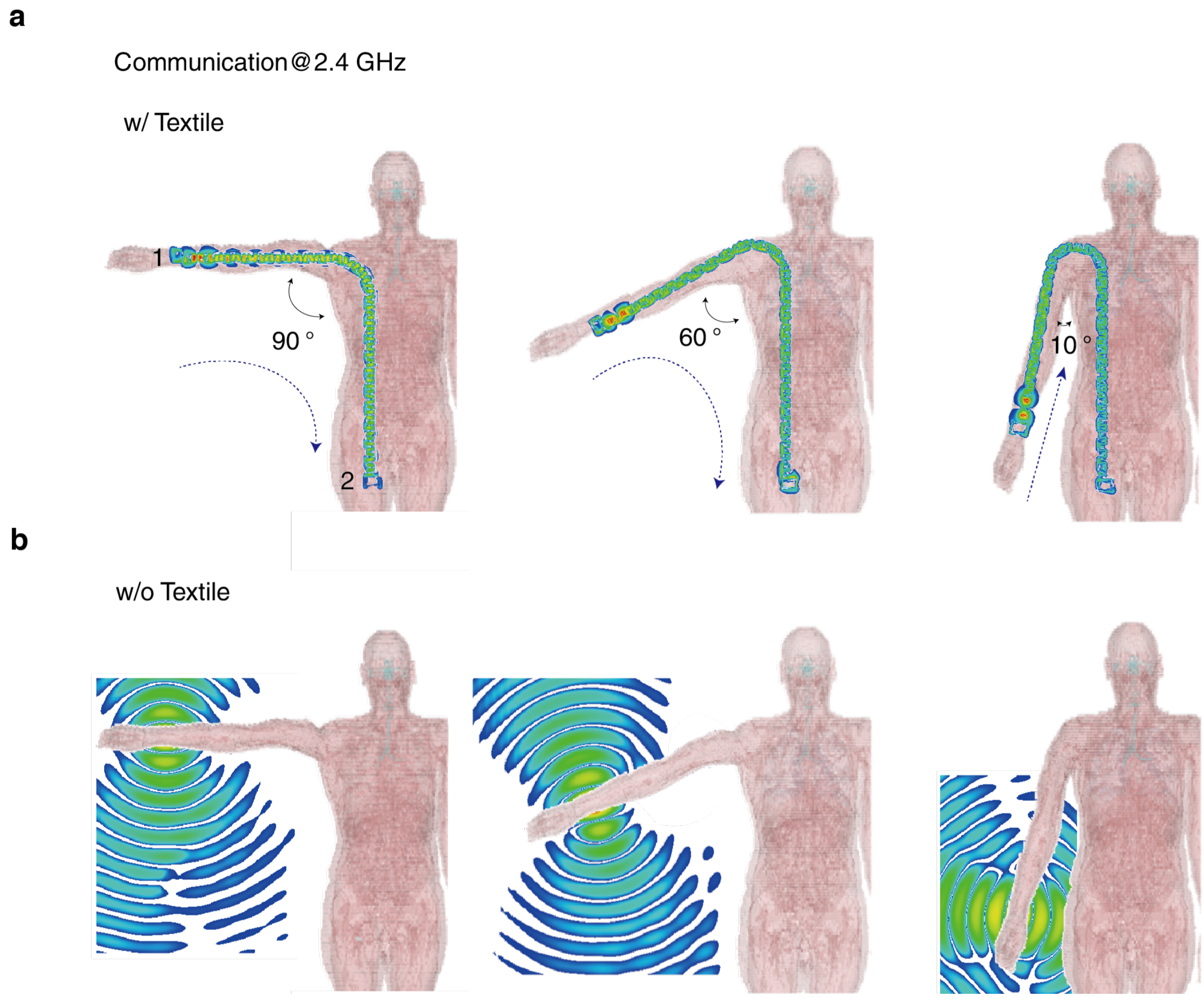

Supplementary Figure 26. **The enhanced wireless communication as the movement of the human body change.** (**a,b**) Data communication on the human body from the wrist (sensor node 1) to the leg with or without metamaterial textiles,the communication cannot be established between the smartphone and sensor node without metamaterial textiles.

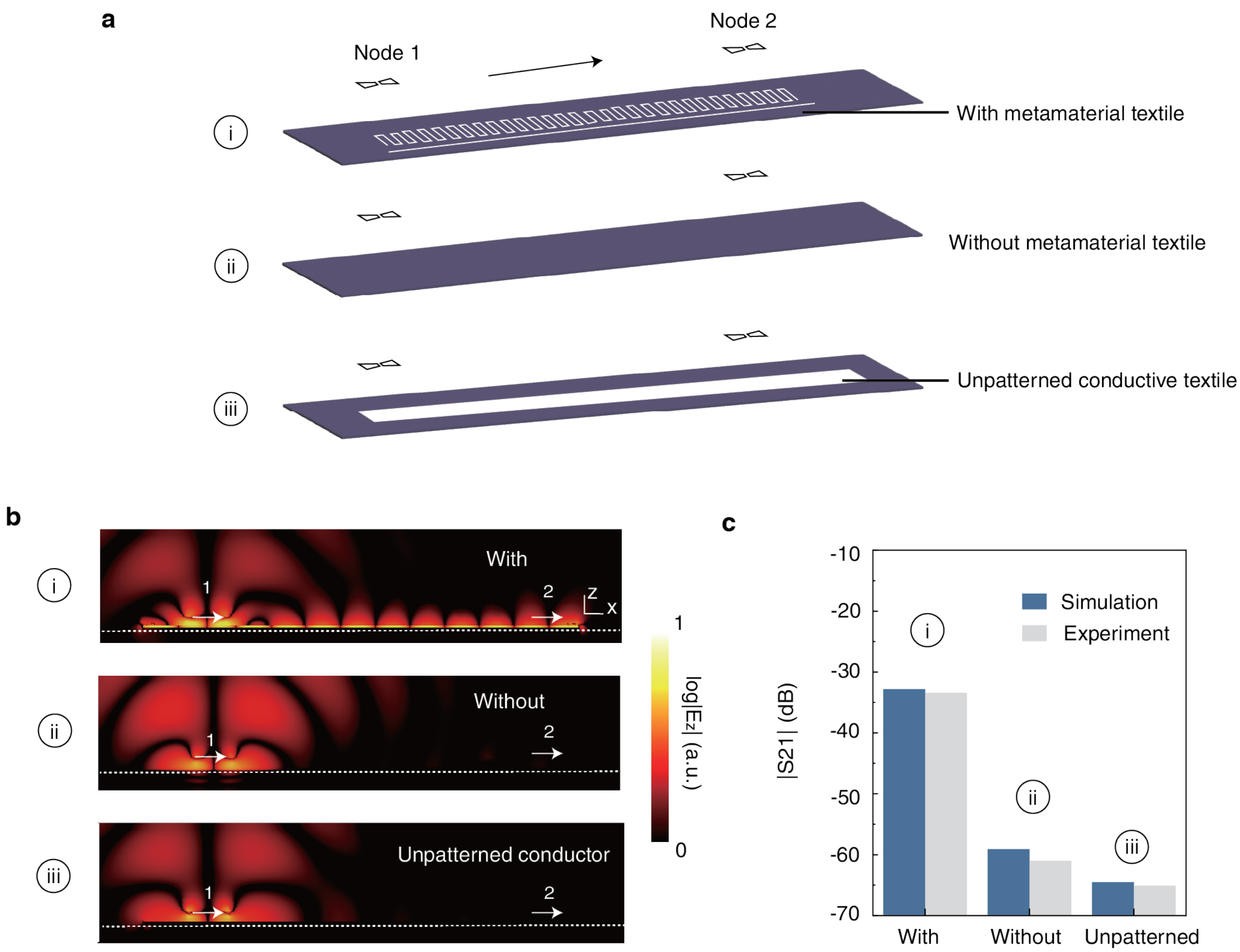

Supplementary Figure 27. **Comparison of wireless communication between different textiles.** (**a**) Schematic illustration of comparison setup. Two antennas are placed 1 cm above the textiles with a fixed separation distance of L = 50 cm. (**b**) Simulated illustration of electric field distribution emitted by a dipole above a metamaterial textile (top), normal textile (centre) and unpatterned conductor (bottom). The textile is placed on an air–body half-space. Only metamaterial textile supports the surface wave propagation. (**c**) Measured and simulated results of transmission coefficient between different textiles.

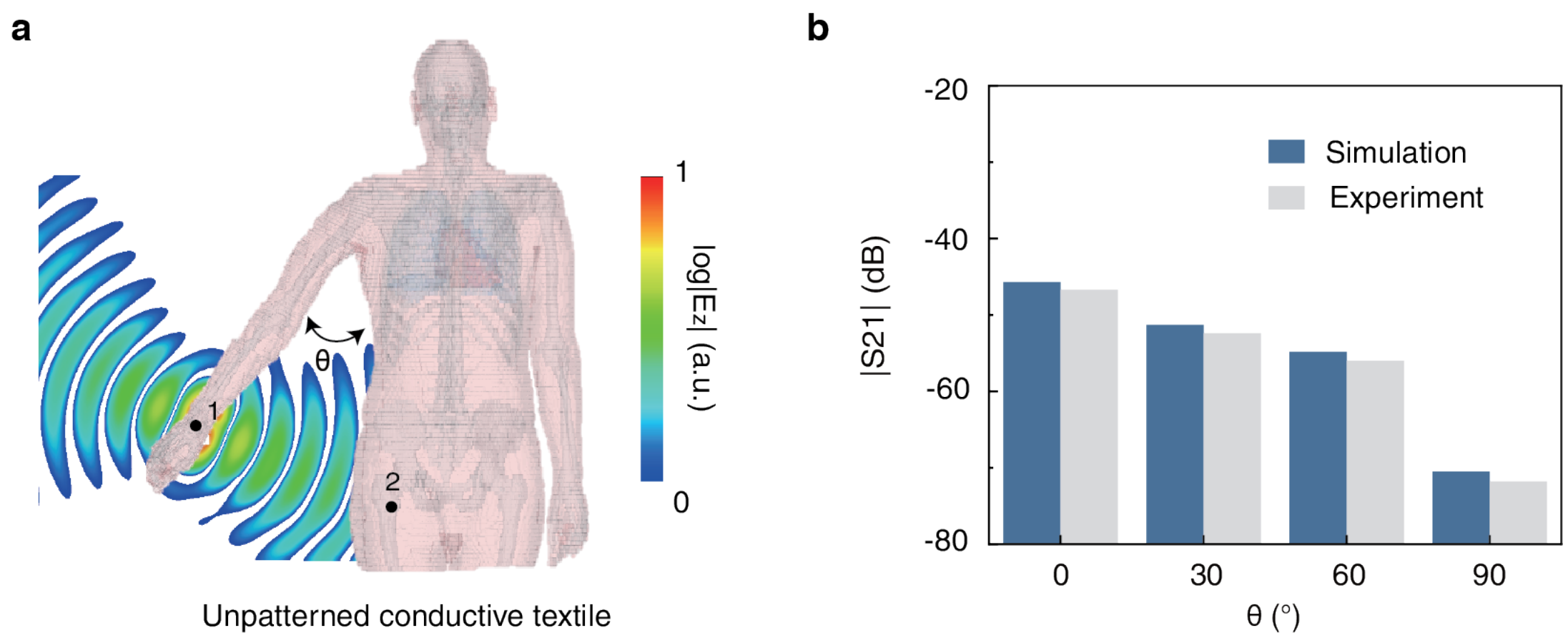

Supplementary Figure 28. **Wireless communication performance of the unpatterned conductive textile on the human body.** (**a**) Simulated electric field distribution. (**b**) Simulated and on-body measured transmission coefficient as functions of arm-raising angle, with the unpatterned conductor. The data are plotted based on single measurements.

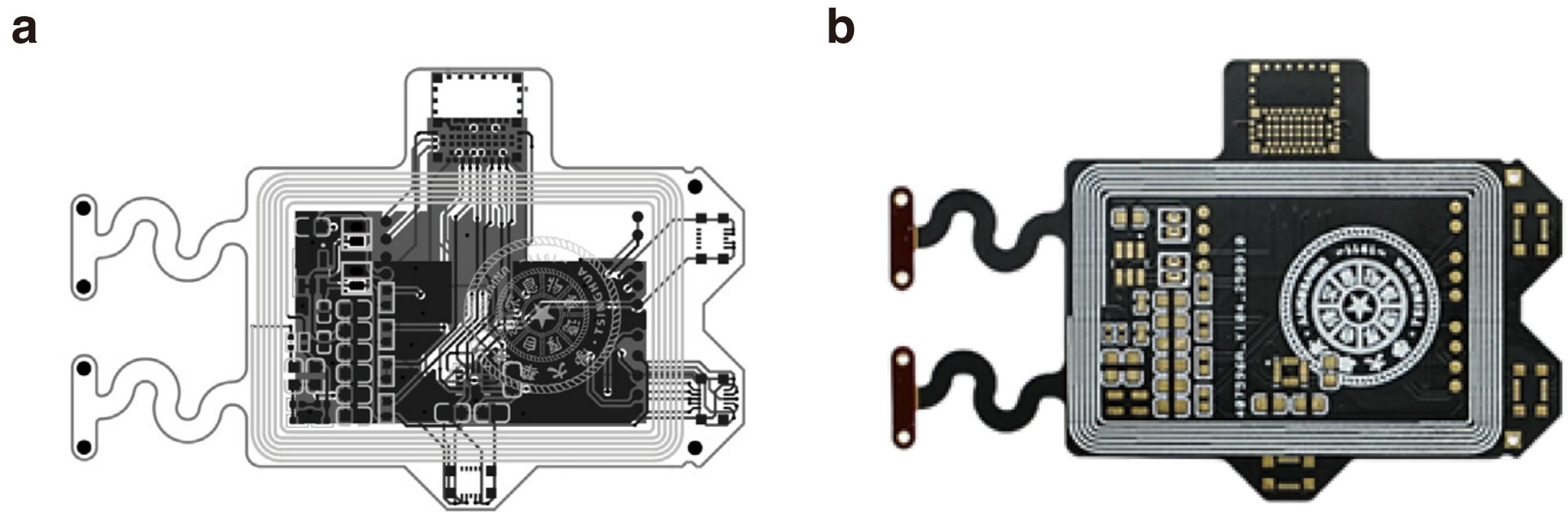

Supplementary Figure 29. **Layout diagram and photograph of the glove sensor node.** (**a**) Glove wireless sensor node PCB layout. The PCB adopts a four-layer layout, incorporating a coil, power management module, IMU, BLE MCU, and three board-to-board connectors. (**b**) Photograph of Glove wireless sensor node PCB. The serpentine traces on the left side of the PCB are designed to secure the connection with the glove while maintaining mechanical stretchability.

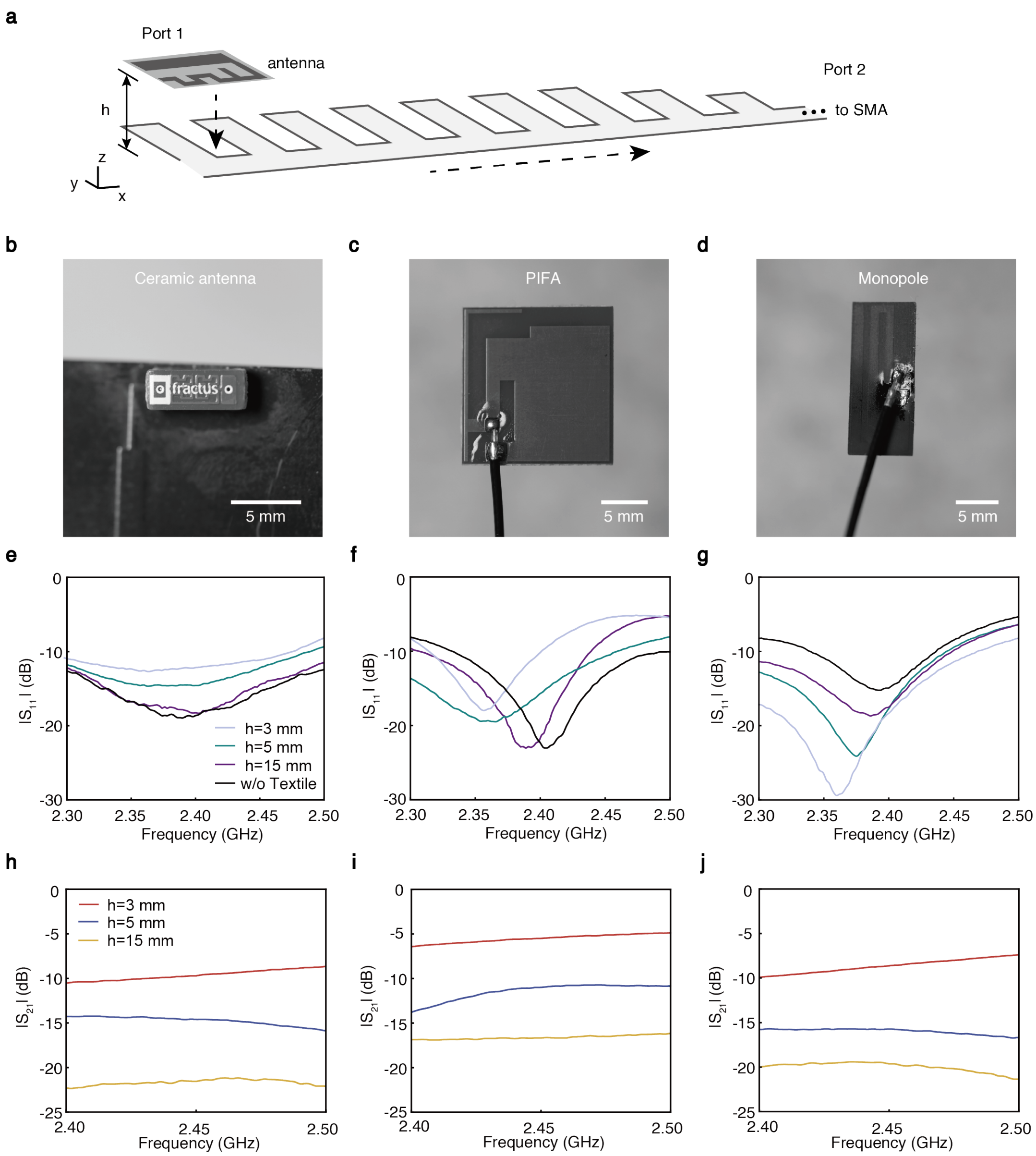

Supplementary Figure 30. **Measured reflection and transmission characteristics of different commercial antennas in proximity to an SSP metamaterial textile.** (**a**) Schematic illustration of the measurement setup, where h denotes the vertical separation between the antenna and the textile. (**b-d**) Photographs of the three transmitting antennas evaluated in this study: (**b**) ceramic antenna, (**c**) planar inverted-F antenna (PIFA), and (**d**) monopole antenna. (**e-g**) Measured reflection coefficient $|S_{11}|$ spectra of the three antennas under different separation distances (h = 3 mm, 5 mm, and 15 mm), together with the corresponding reference measurements obtained without the metamaterial textile. (**h-j**) Measured transmission coefficient $|S_{21}|$ spectra over the 2.4–2.5 GHz ISM band, characterizing the coupling efficiency between the antennas and the SSP waveguide, as well as the subsequent guided-wave transmission to the waveguide port.

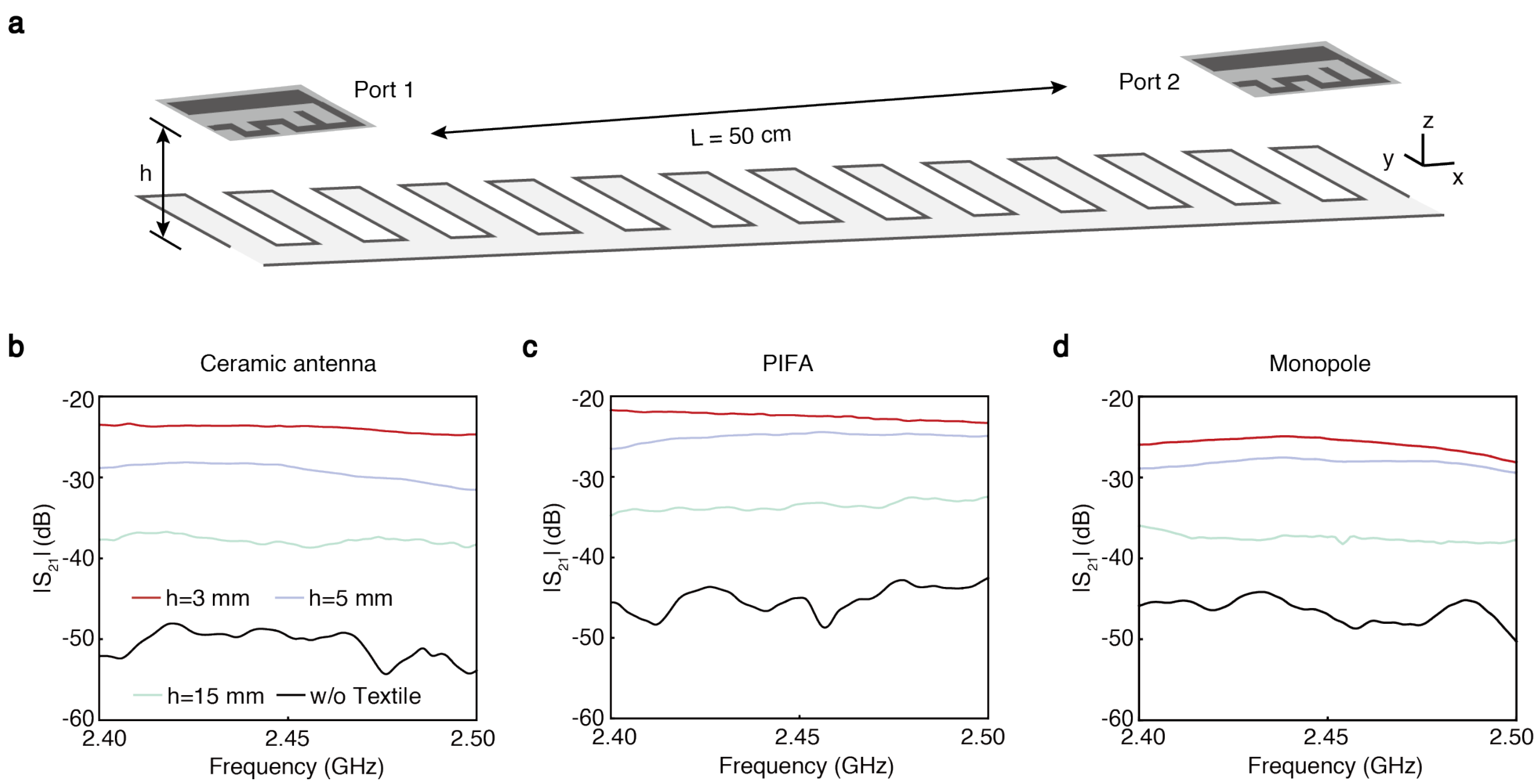

Supplementary Figure 31. **Metamaterial-enhanced wireless transmission characteristics using different commercial antennas.** (**a**) Schematic illustration of the measurement setup. Two antennas are placed above the metamaterial textile with a fixed separation distance of L = 50 cm, while the antenna-to-textile separation h is varied. (**b-d**) Measured transmission coefficient $|S_{21}|$ across the 2.40–2.50 GHz ISM band for (**b**) the ceramic antenna, (**c**) the PIFA, and (**b**) the monopole antenna.

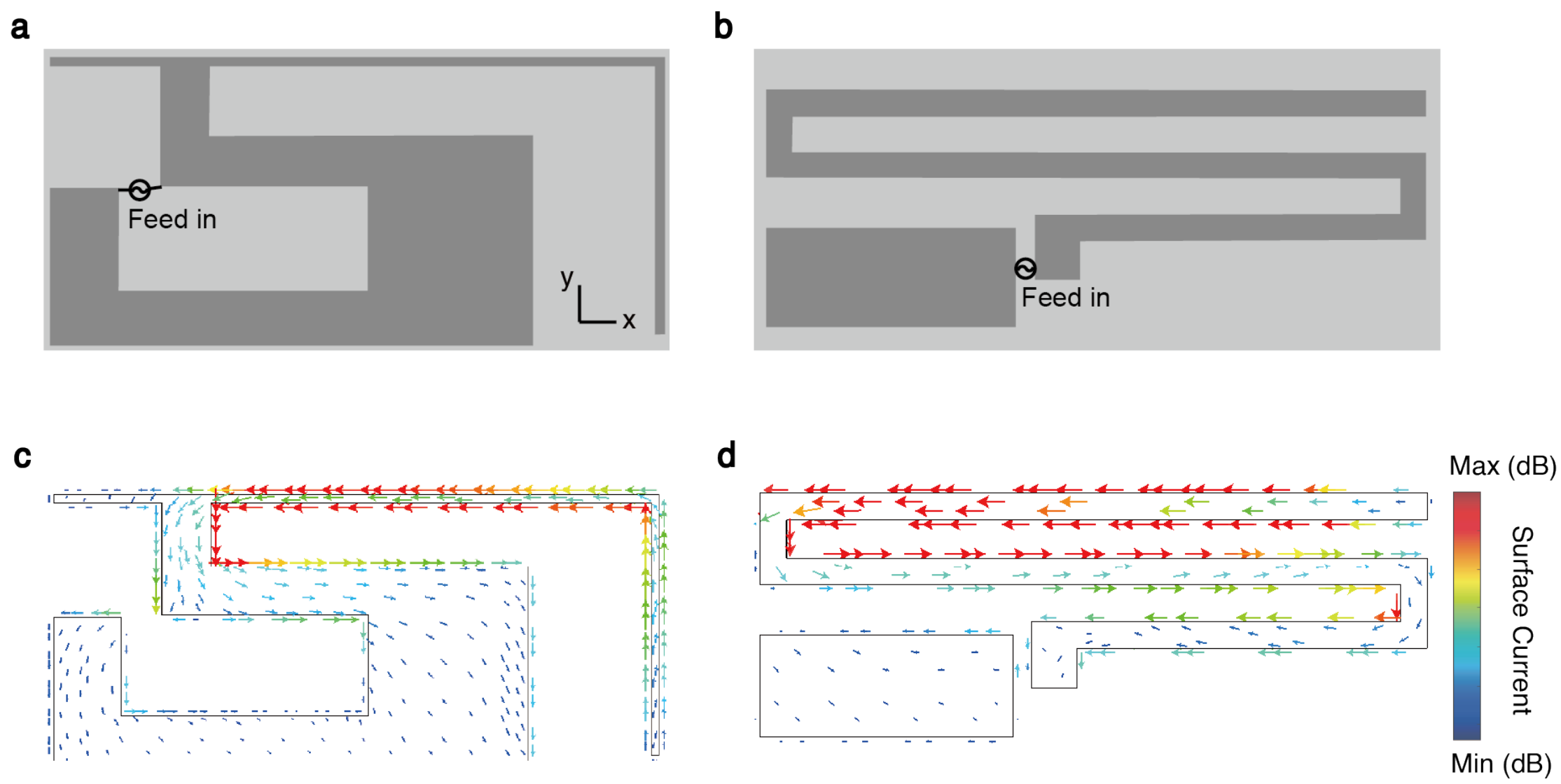

Supplementary Figure 32. **Geometric structures and simulated surface current distributions of PIFA and monopole antenna.** (**a**) Geometric structure of the PIFA. (**b**) Geometric structure of the monopole antenna. (**c**) Simulated surface current distribution on the PIFA at 2.4 GHz. (**d**) Simulated surface current distribution on the monopole antenna at 2.4 GHz.

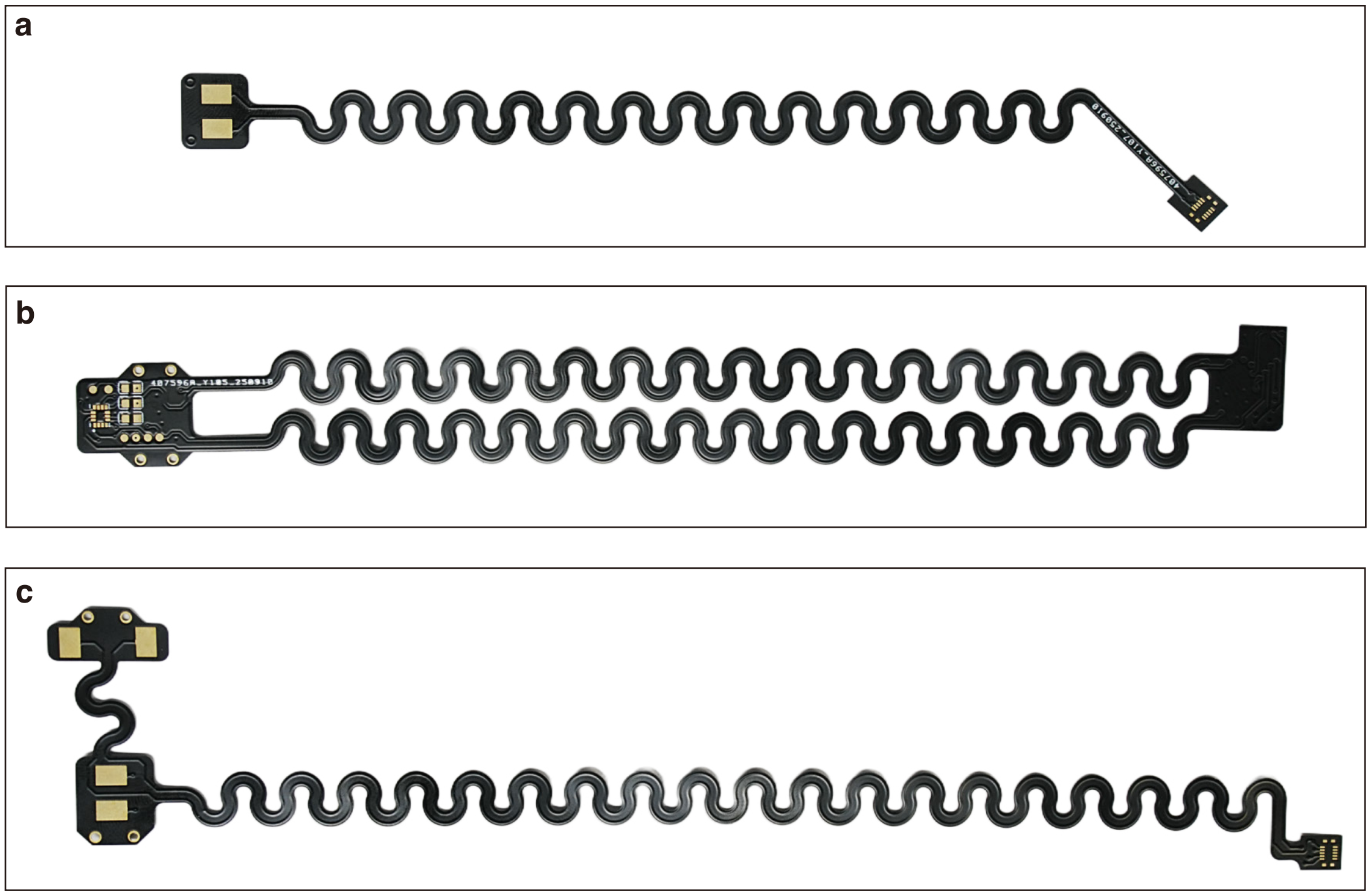

Supplementary Figure 33. **Finger circuit assemblies.** (**a**) Thumb circuit with strain-sensor interface. (**b**) Index-finger circuit with IMU and strain-sensor interface. (**c**) Middle-finger circuit with strain and pressure interfaces.

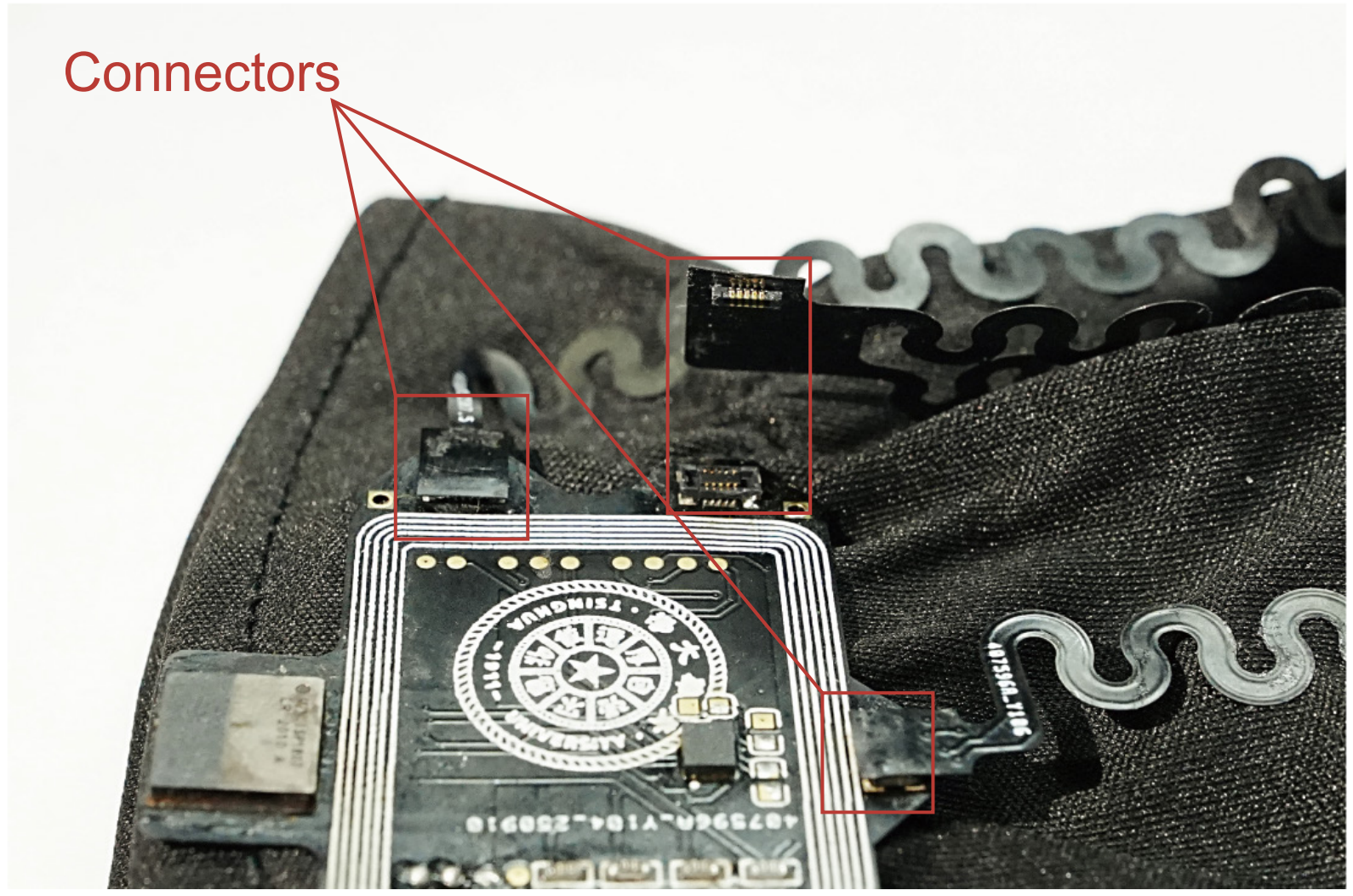

Supplementary Figure 34. **Board-to-board connector interface.** Photograph showing mechanical and electrical integration between finger circuits and the wireless sensor node.

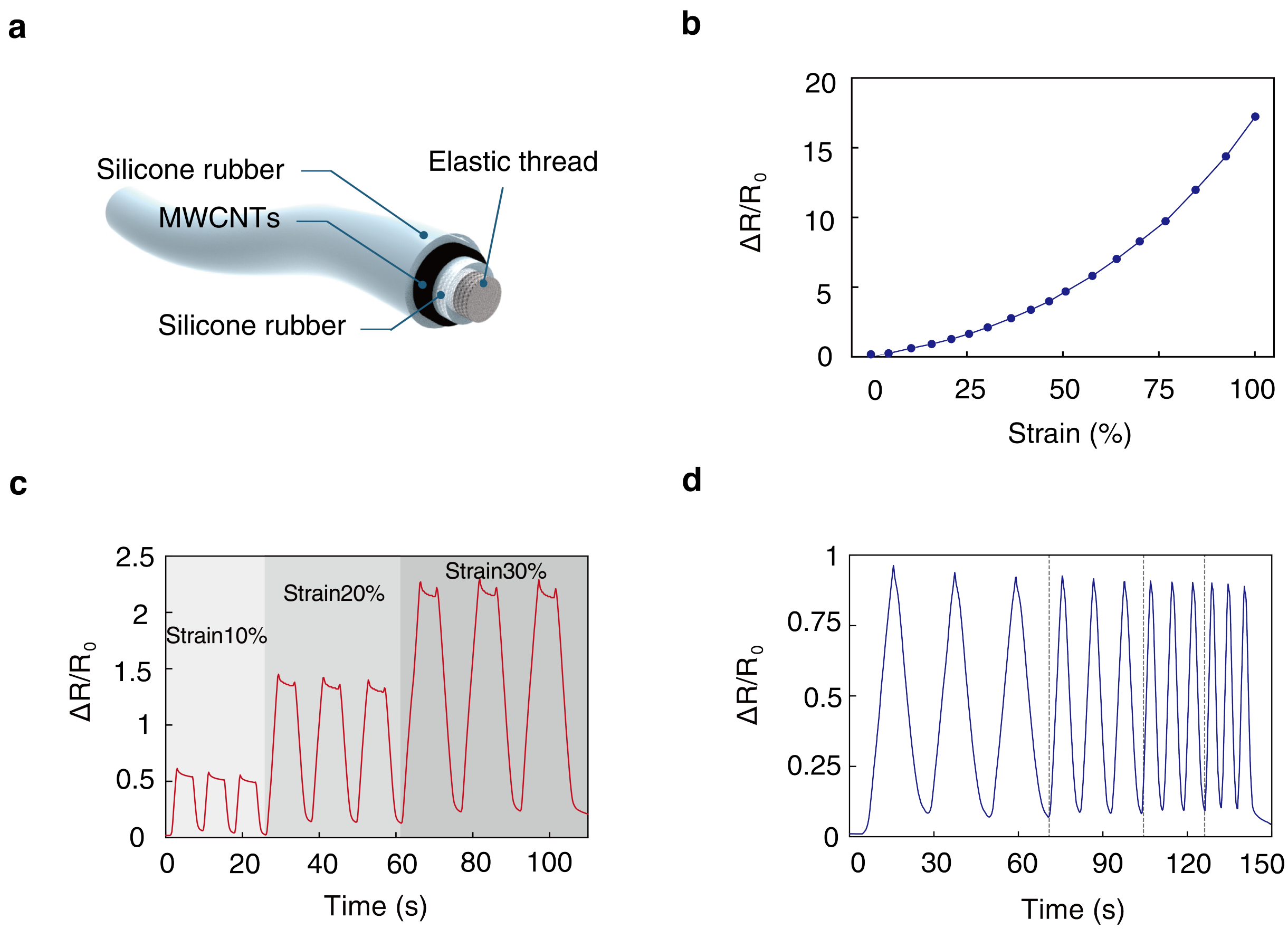

Supplementary Figure 35. **Structural configuration and electromechanical performance of the stretchable strain sensor.** (**a**) Schematic diagram of the stretchable strain sensor.(**b**) Relative resistance change of the stretchable strain sensor under different applied strains.(**c**) Dynamic response of the strain sensor under cyclic tensile strains of 10 percent, 20 percent, and 30 percent. (**d**) Dynamic electromechanical response of the strain sensor under cyclic 15 percent strain, tested at stretching rates of 100, 200, 300, and 400 mm per min.

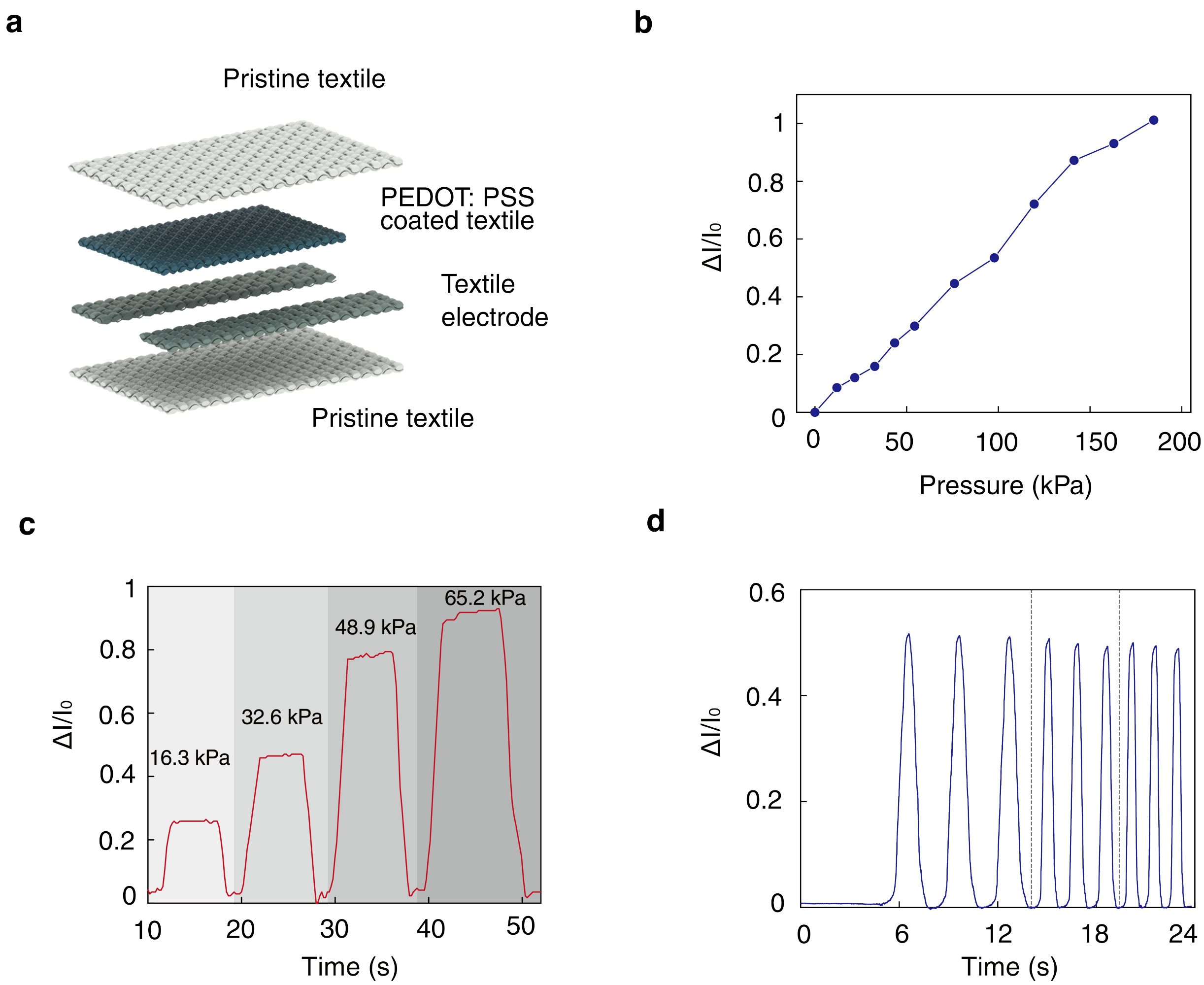

Supplementary Figure 36. **Structure design and performance of the textile-based pressure sensor.** (**a**) Exploded-view schematic illustrating the multilayer architecture of the sensor.(**b**) Relative change in current as a function of applied pressure.(**c**) Real-time current response of the pressure sensor under different applied pressure levels. (**d**) Dynamic performance under a 81.5 kPa load at different loading speeds (50, 100 and 150 mm per min.

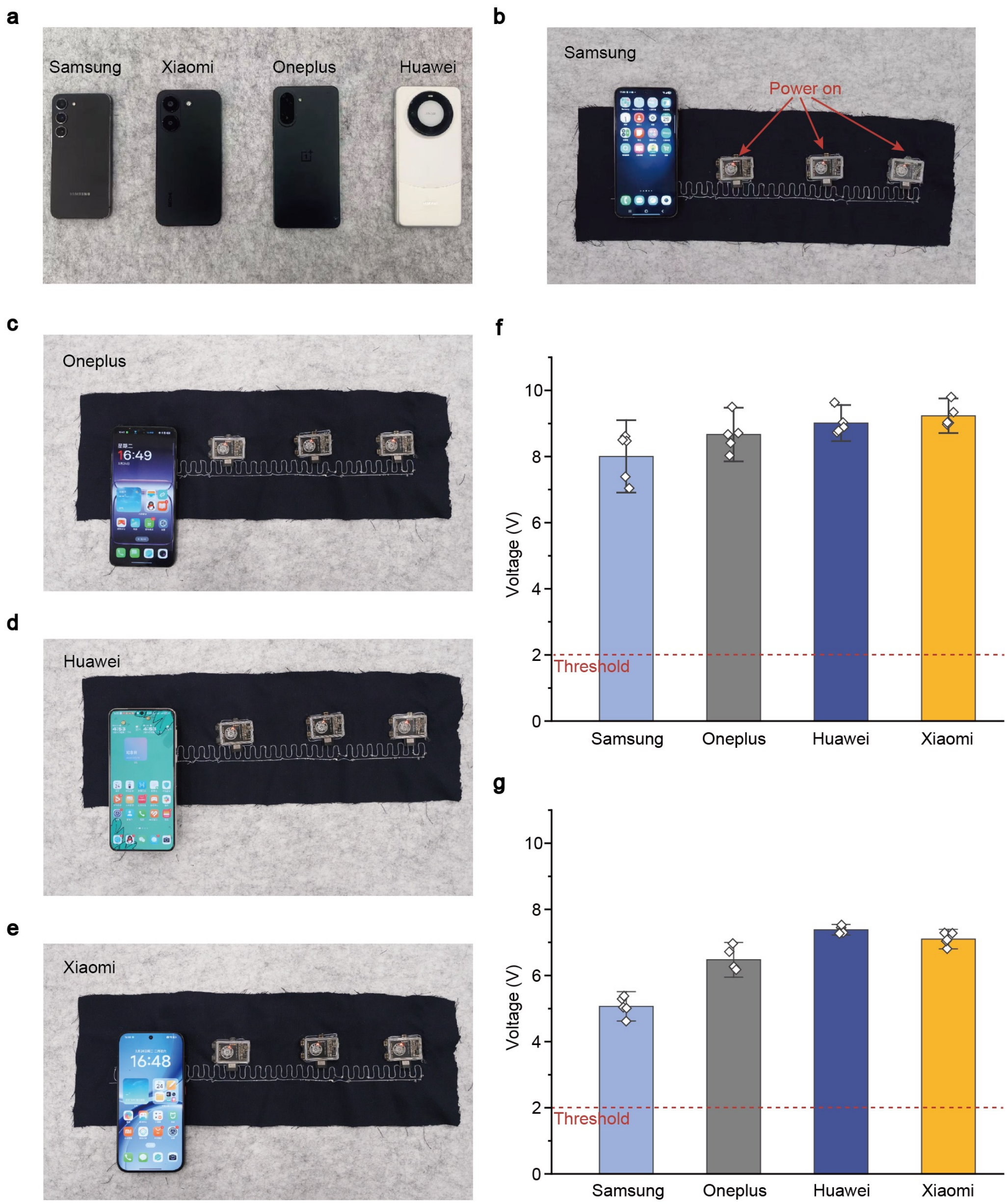

Supplementary Figure 37. **Quantitative characterization of end-to-end power transfer using commercial smartphones.** (**a**) Photographs of the four commercial smartphone models utilized for validation (Samsung S23, Xiaomi Redmi turbo5, OnePlus Ace5 and Huawei Mate60). (**b-e**) Experimental demonstrations of the smartphone-powered WBSN under a simultaneous three-node loading condition. (**f**) Measured rectified voltage of a single batter-free sensor node powered by four different smartphone models. Error bars show mean ± s.d. (n = 5 measurements) (**g**) Average rectified voltage across three concurrent battery-free sensor nodes powered by different smartphone models. Error bars show mean ± s.d. (n = 5 measurements)

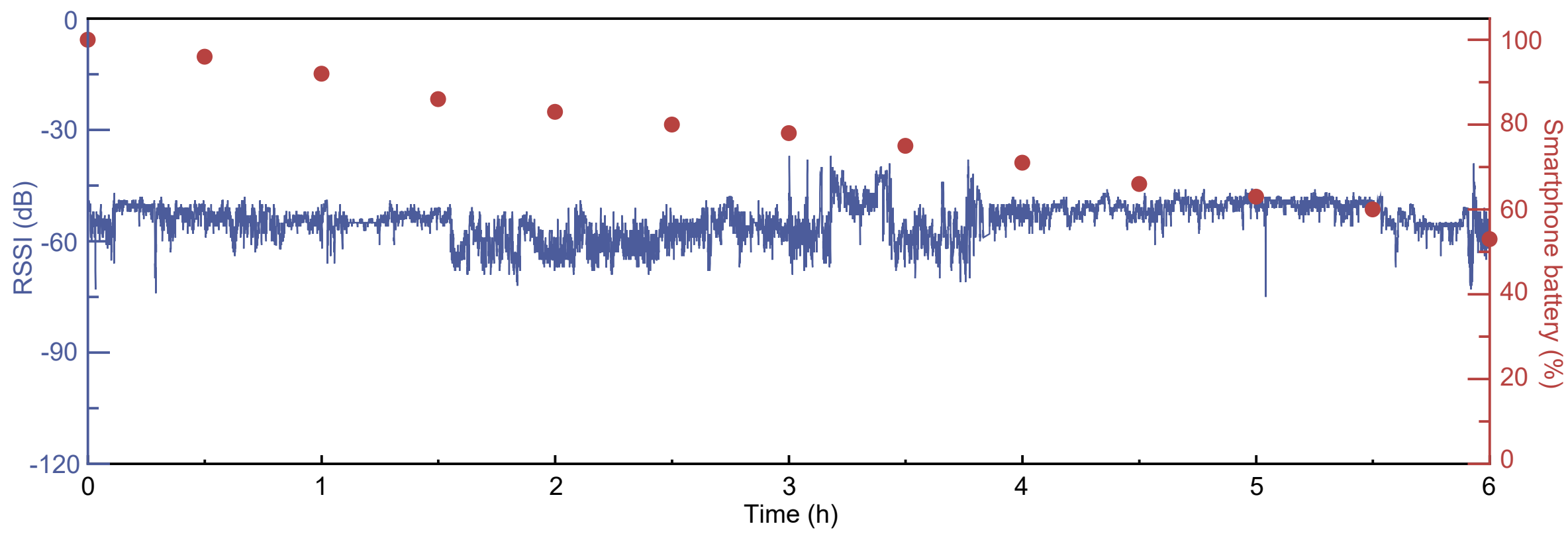

Supplementary Figure 38. **Long-term continuous operation of the smartphone-powered WBSN system.** The RSSI of a sensor node remained stable throughout the 6-hour continuous operation, with an average value of -54.34 ± 4.72 dB, demonstrating stable wireless communication performance under extended operation. Over the same period, the smartphone battery level decreased from 100% to 53%, accounting for power consumption from wireless powering (NFC), display activity, and background processes. It is noted that IMU measurements inherently suffer from time-dependent drift; after 6 hours of static operation, the accumulated angular drift reached 0.69°, 0.12°, and 0.22° for the roll, pitch, and yaw axes, respectively.

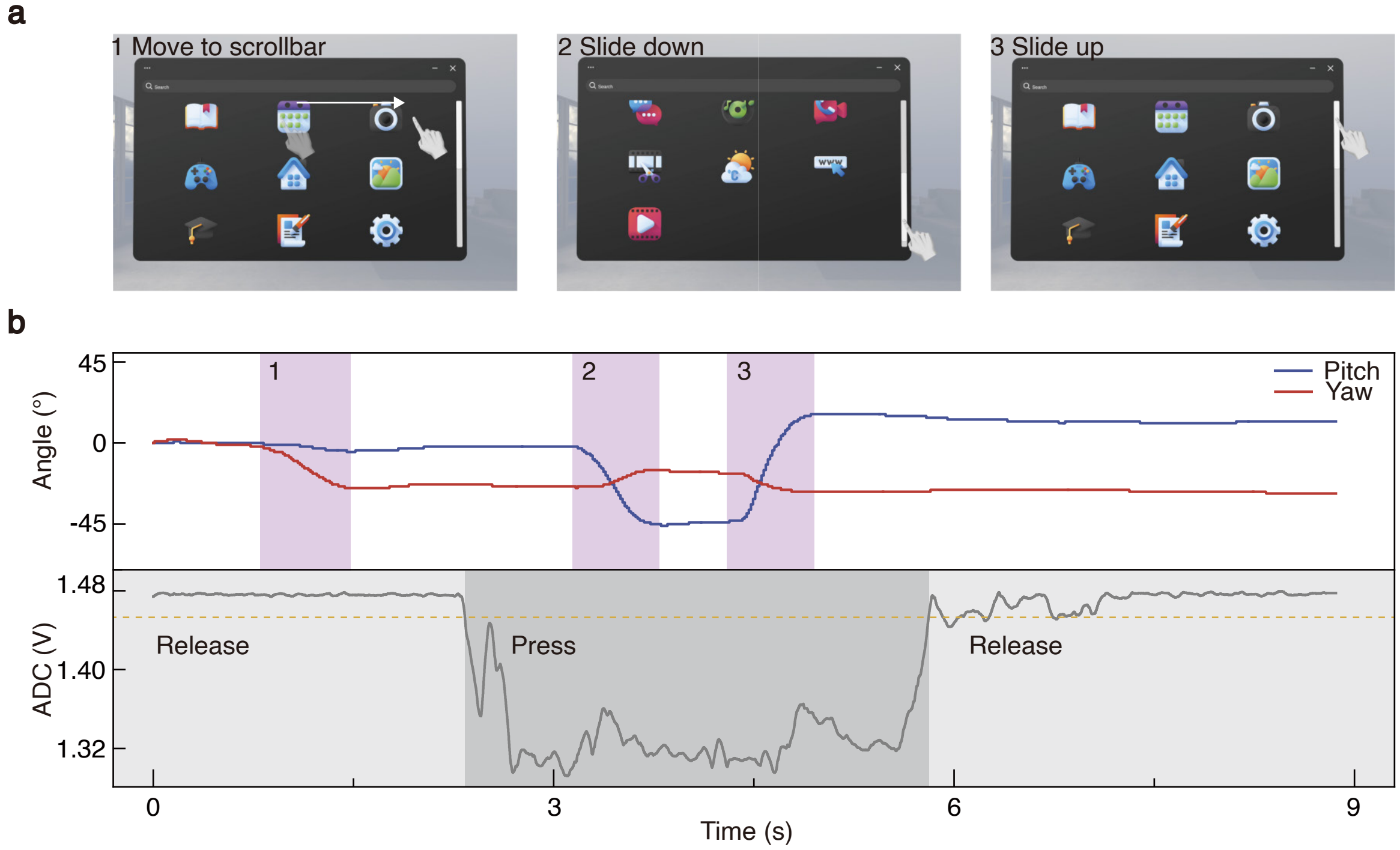

Supplementary Figure 39. **Real-time panel sliding control.** (**a**) VR viewport sliding diagram. (**b**) Real-time data display from the finger-worn IMU and pressure sensor during panel sliding. The IMU controls the cursor movement, while the pressure sensor handles the click action.

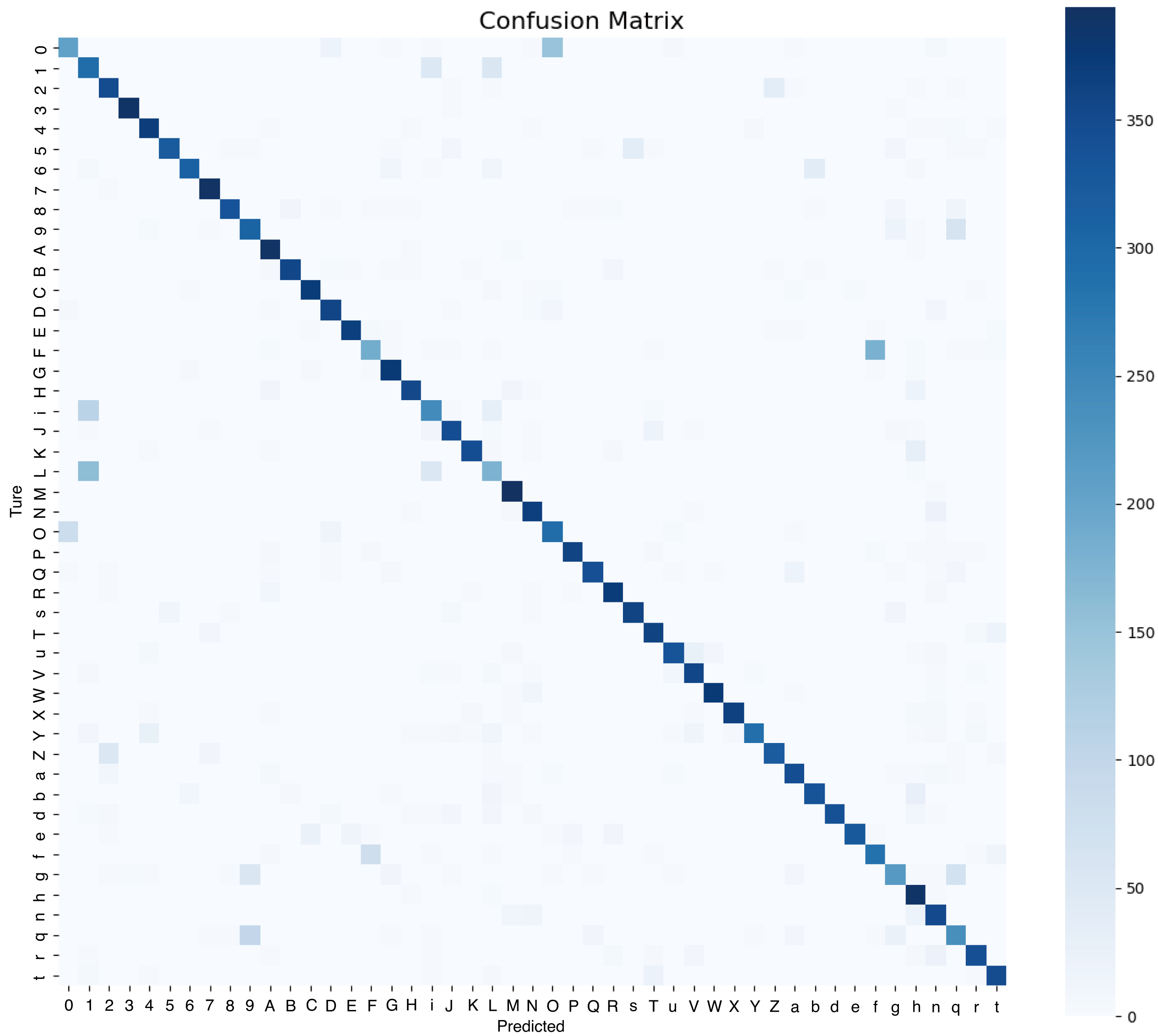

Supplementary Figure 40. **Confusion matrix for 47-character recognition.** Recognition results for 47 digits and letters (400 samples per class); visually similar upper- and lower-case letters were merged.

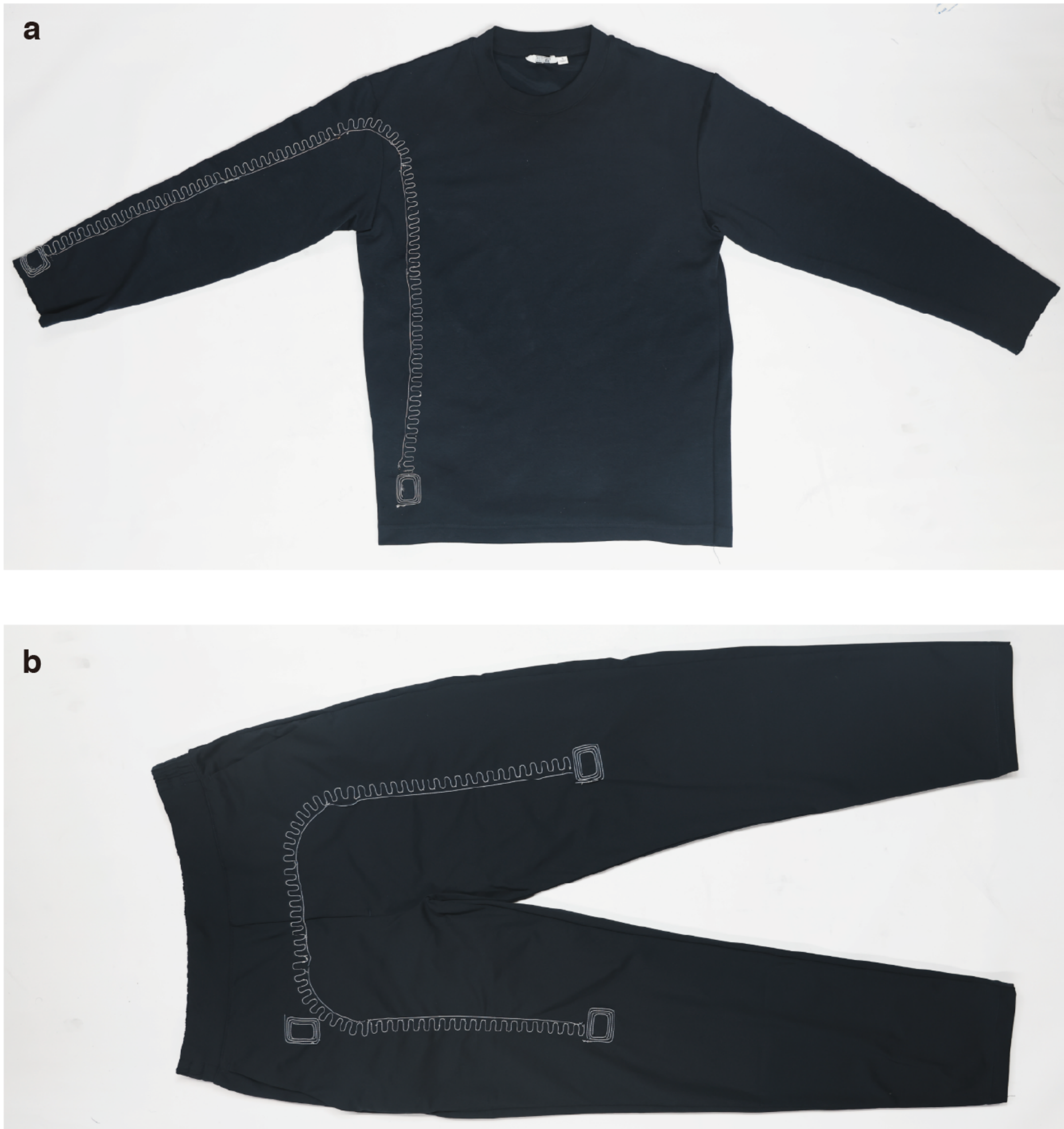

Supplementary Figure 41. **The overall architecture diagram of the full-body WBSN.** (**a**) Photograph of the upper garment WBSN. (**b**) Photograph of the lower garment WBSN.

a

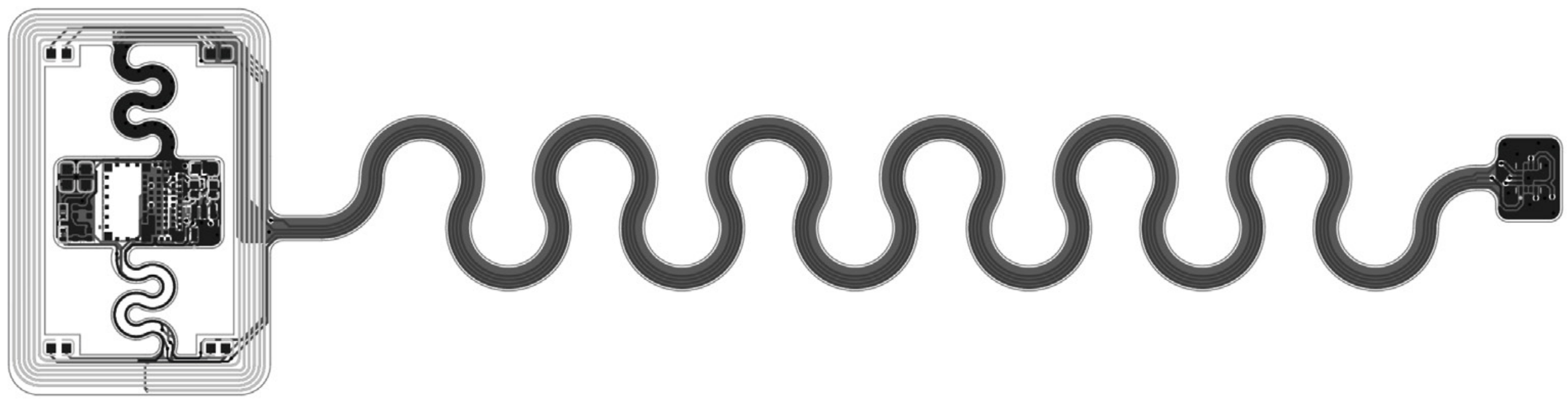

b

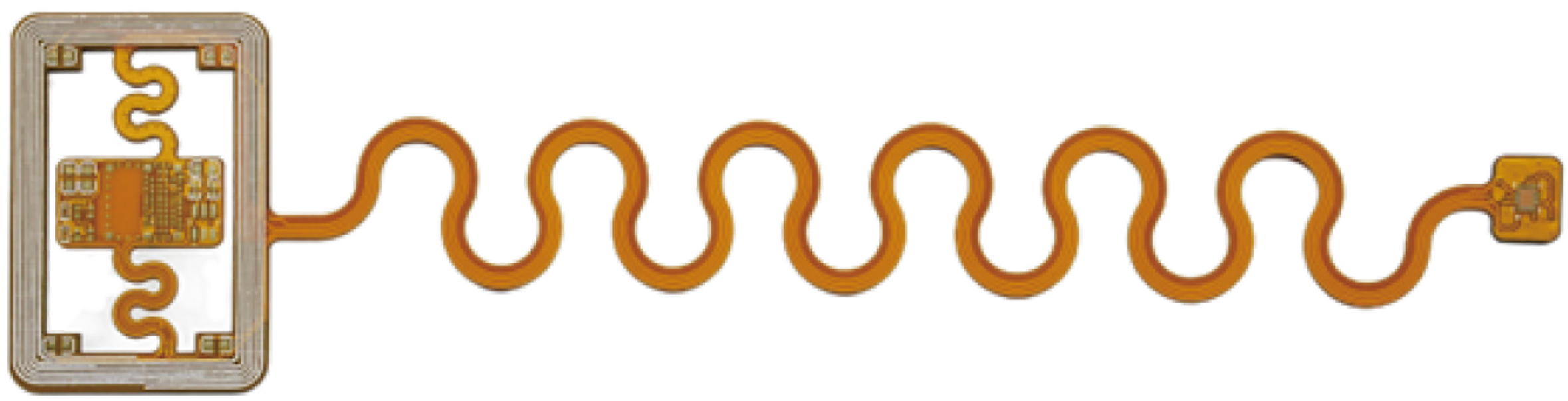

Supplementary Figure 42. **Layout diagram and photograph of the epidermal motion sensor.** (**a**) Epidermal motion sensor PCB layout. The PCB adopts a four-layer layout, incorporating a coil, a power management module, two IMUs, and a BLE MCU. (**b**) Photograph of epidermal motion sensor PCB. One of the IMUs is positioned at the end of the serpentine traces to capture motion data from the lower leg. This design ensures mechanical stretchability, allowing the sensor to maintain close contact with the leg during knee movement.

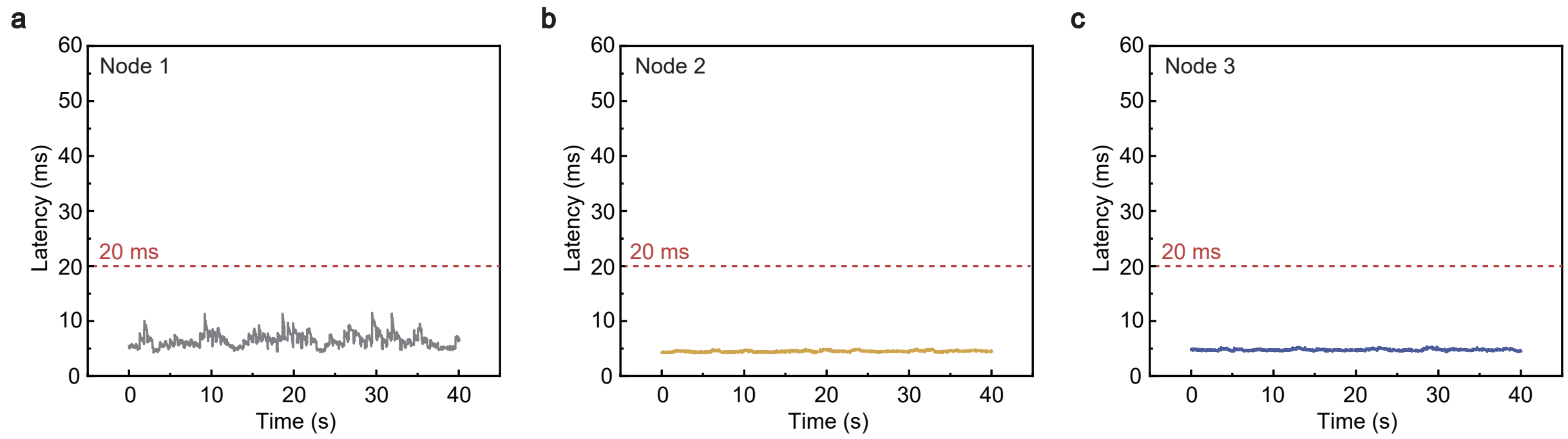

Supplementary Figure 43. **Transmission latency during high-intensity motion.** (**a**) Node 1 shows the communication latency of the battery-free glove during motion. (**b-c**) Nodes 2 and 3 show the communication latency of the battery-free epidermal sensor nodes.

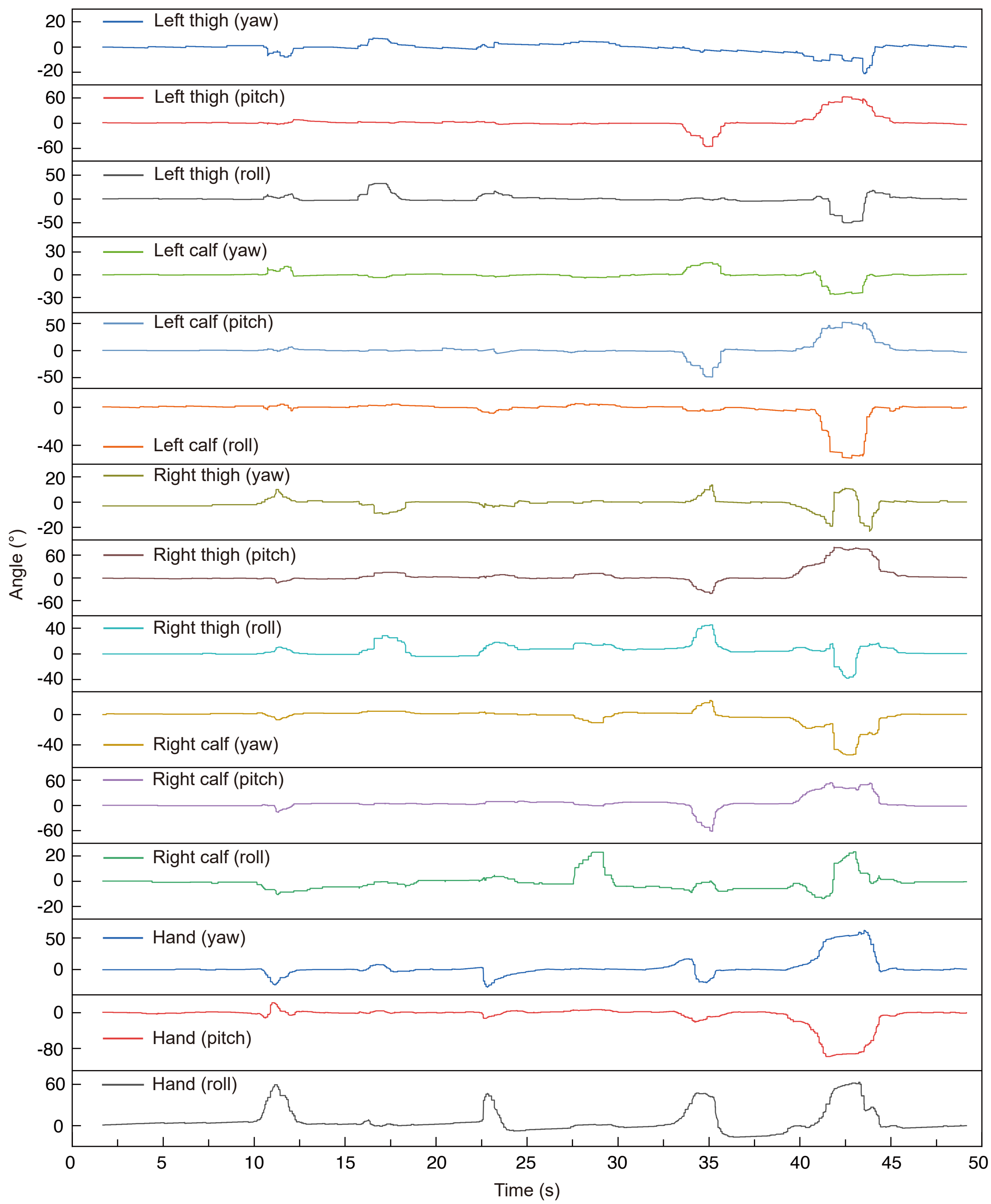

Supplementary Figure 44. **Three battery-free sensor nodes data for motion capture.** Three wireless devices were placed on the right hand and both knees, providing 15-axis IMU data for full-body motion capture.

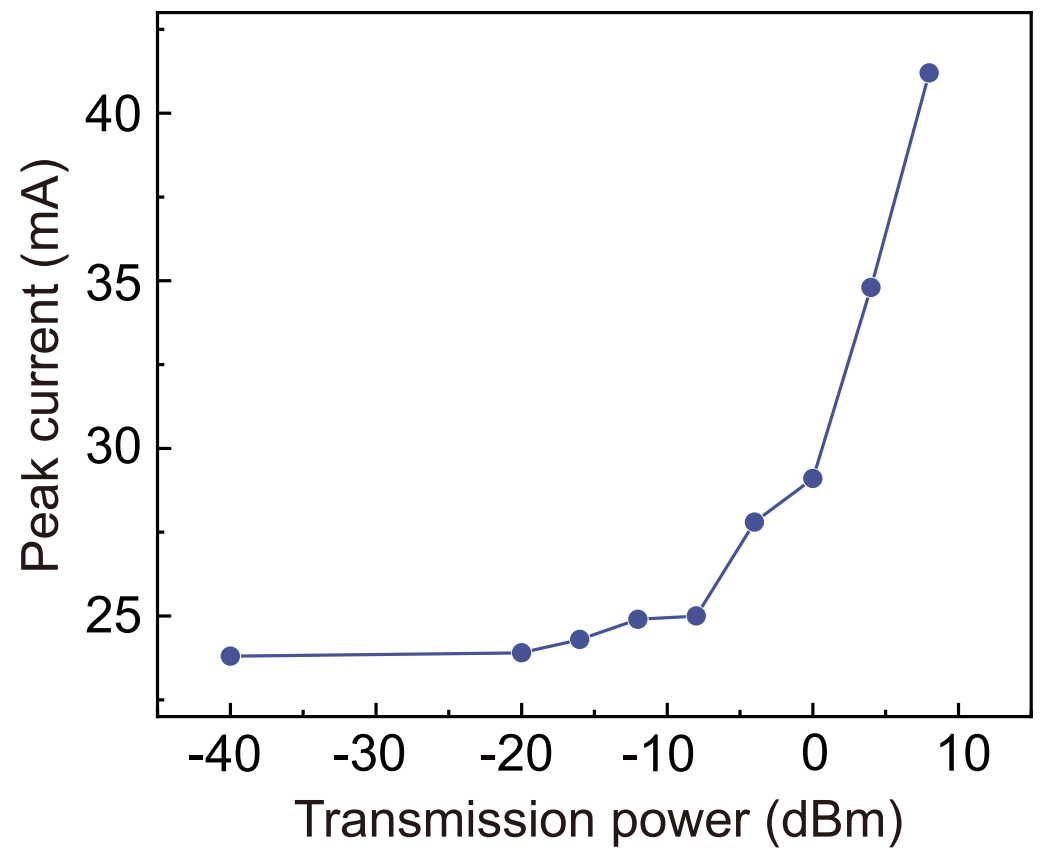

Supplementary Figure 45. **Peak current consumption at different transmit powers.** The transmission power was set to 8 dBm, 4 dBm, 0 dBm, -4 dBm, -8 dBm, -12 dBm, -16 dBm, -20 dBm, and -40 dBm, respectively, and the current variation over 500 ms was recorded. At 8 dBm, the peak current of BLE device during BLE data transmission reached nearly 40 mA, while at -40 dBm, the transmission current decreased to approximately 25 mA, indicating reduced instantaneous power consumption at lower output levels.

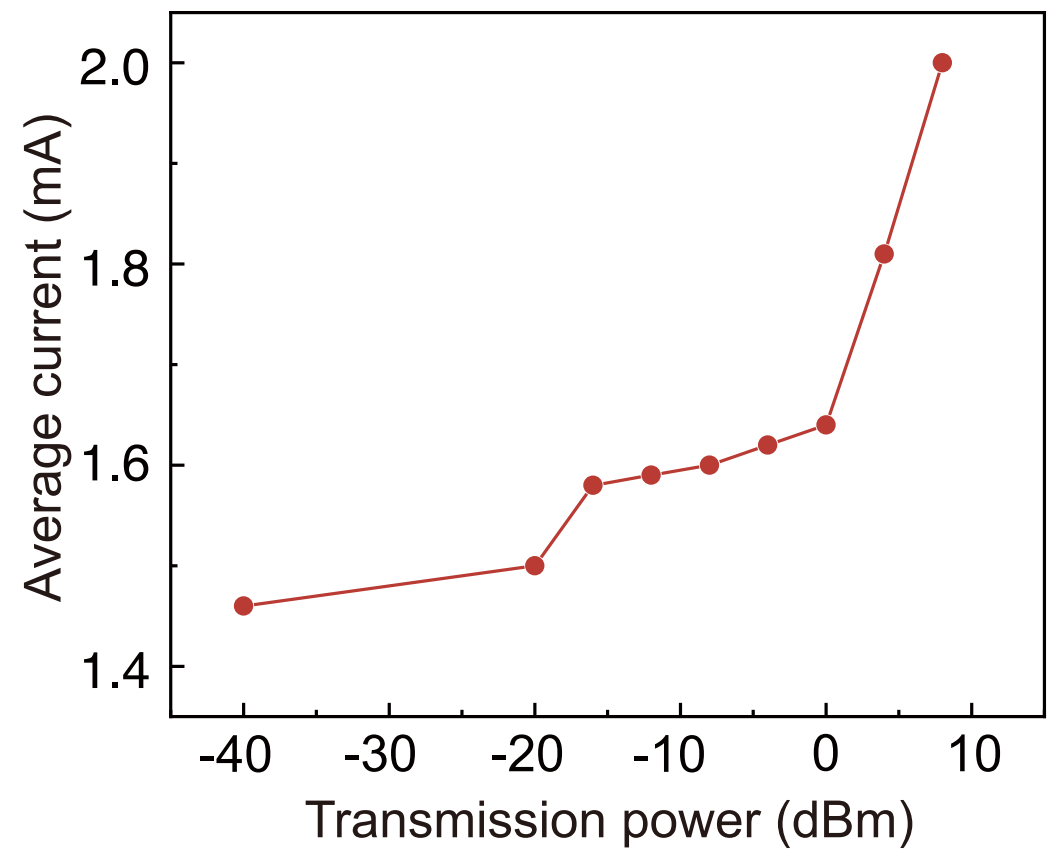

Supplementary Figure 46. **Average current consumption at different transmission powers.** Average current of BLE device was 2.00 mA at 8 dBm and 1.46 mA at -40 dBm, indicating a monotonic decrease with transmit power.

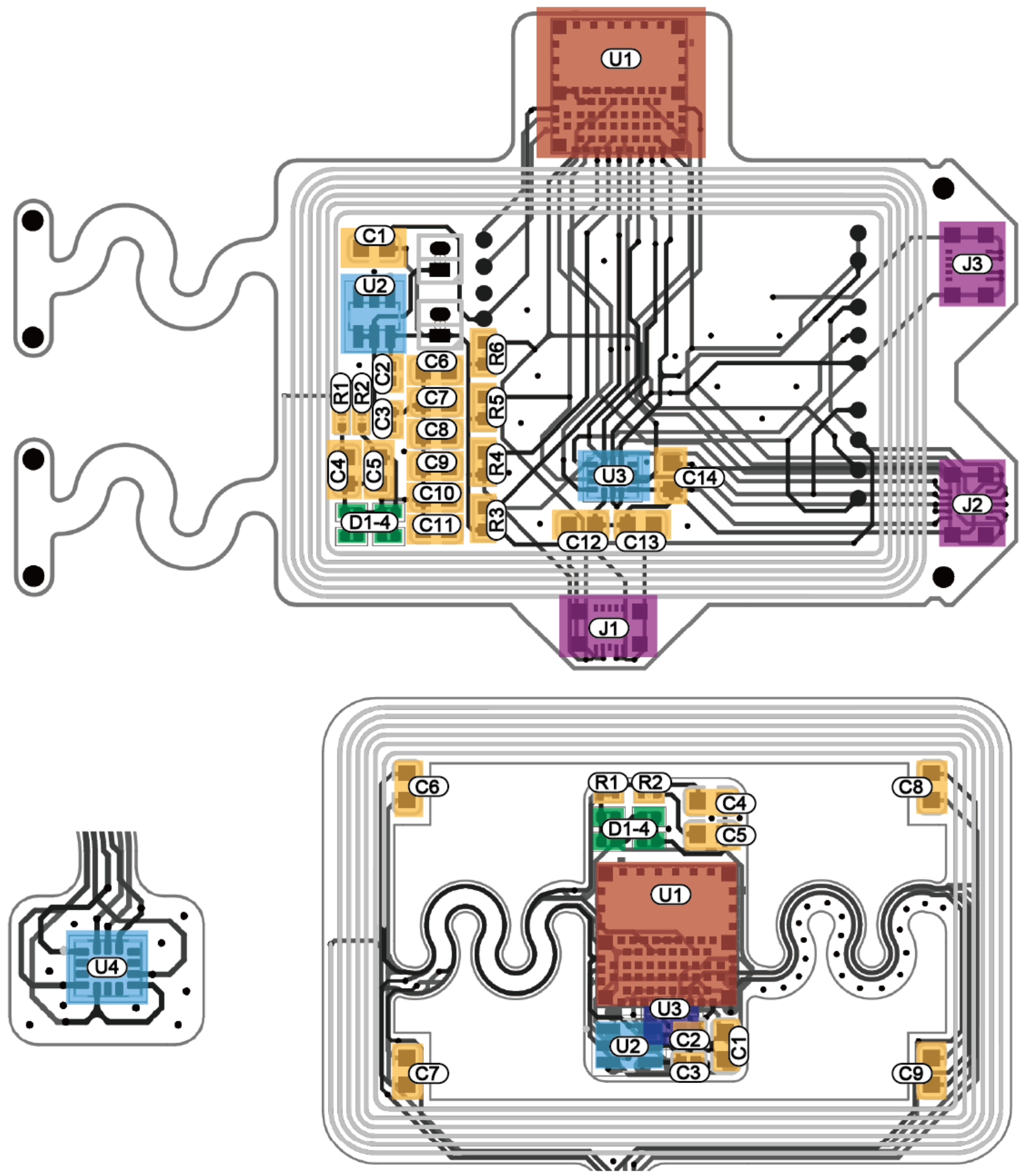

U1	ISP1807
U2	AP7312-1833
U3, U4	LSM6DSOTR
D1-4	CDBQC0130L-HF
J1-3	X0400FVS-10-LPV01
R1, R2	0R (0402)
R3-5	100K (0603)

C1, C12	100nF (0603)
C2, C3	1uF (0402)
C4, C5	100pF (0603)
C6-C11	22uF (0603)
C13	2.2uF (0603)
C14	10nF (0603)

Supplementary Figure 47. **Sensor nodes schematic.** Battery-free sensory glove and epidermal sensor node circuitry with components identified.

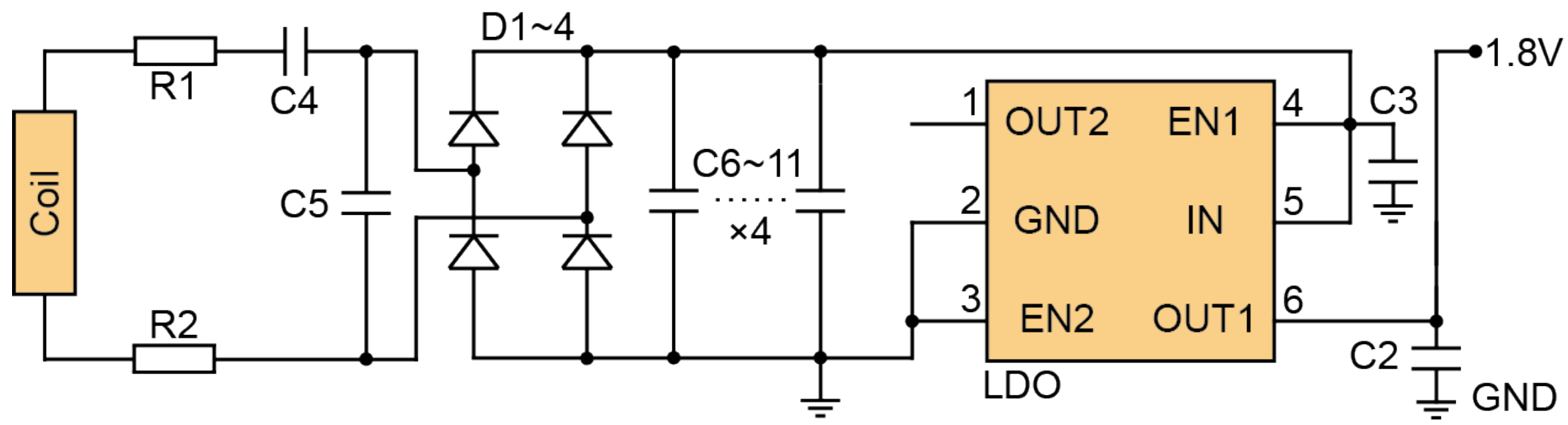

R1, R2	0 Ω	C6~11	22 uF
C2, C3	1 uF	C4, C5	100 pF

LDO	AP7312-1833
D1~4	CDBQC0130L-HF

Supplementary Figure 48. **Power management circuit.** Schematic diagram and component parameters of power management circuit.

III. SUPPLEMENTARY TABLES

Supplementary Table 1. Comparison of recent metamaterial textile-enabled WBSN technologies

Reference	Nat. Electron. 2, 243–251 (2019)	Nat. Commun. 11, 444 (2020)	Nat. Electron. 4, 808-817 (2021)	Nat. Commun. 13, 2190 (2022)	Nat. Commun. 15, 6589 (2024)	Nat. Electron. 9, 59–68 (2026)	This work
**Supporting protocal**	Single (Bluetooth, 2.4 GHz)	Single (NFC, 13.56 MHz)	Single (NFC, 13.56 MHz)	Single (NFC, 13.56 MHz)	Single (NFC, 13.56 MHz)	Single (Bluetooth, 2.4 GHz)	Dual-mode (NFC & Bluetooth, 13.56 MHz & 2.4 GHz)
**Signal and power transmission**	Surface plasmon propagation	Magnetic coupling	Magnetic coupling	Magnetic coupling	Magnetic coupling	Surface plasmon propagation	Surface plasmon propagation & Magnetic coupling
**Data rate**	High (Up to 1 Mbps)	Low (26.48 kbps)	Low (26.48 kbps)	Low (26.48 kbps)	Low (26.48 kbps)	High (Up to 1 Mbps)	High (Up to 1 Mbps)
**Sampling rate**	Unclear	1.3 Hz (Up to 8Hz)	5.6 Hz (Up to 32Hz)	8 Hz (Up to 12Hz)	5 Hz (Up to 25Hz)	50 Hz (Up to 400Hz)	100 Hz (Up to 400Hz)
**Power transfer efficiency**	Low (3.5% at 0.5m)	High (About 20% at 1m)	Low (About 1% at 0.8m)	High (48% at 0.5m)	Low (About 1% at 1m)	Low (About 4% at 0.3m)	Very high (About 70% at 1m)
**User-friendliness (whether reader/hub is smartphone)**	Smartphone (only used for communication, need another power sourse)	Specific designed reader (Smartphone only innerconnect single sensor node)	Specific designed reader	Specific designed reader (Smartphone only innerconnect single sensor node)	Specific designed reader (Smartphone only innerconnect single sensor node)	Smartphone (only used for communication, need another power sourse)	Smartphone
**Multi-clothing communication (upper to lower body)**	No	No	Yes	No	Yes	No	Yes
**Stretchability**	None	Poor	None	Good	Poor	Poor	Good
**Air permeability**	Low	High	Low	High	High	High	High
**Washability**	None	Multiple machine-washing	20-min hand-washing	Multiple machine-washing	Multiple machine-washing	None	Multiple machine-washing

*Simultaneously achieving high data rate, high sampling rate, and high power-transfer efficiency is necessary to enable unencumbered human–machine interactions.

Supplementary Table 2. Washing test of the metamaterial textile

Cycles	Transmission coefficient at 2.4 GHz (dB)	Power Efficiency at 13.56 MHz (%)	Rectified Voltage (v)	Duration (min)
0	-18.4	75.86	8.2	0
1	-18.9	74.01	8.0	45
2	-19.3	73.08	7.9	45
3	-20.1	72.16	7.2	45
Percentage change after 3 cycles	-9.24%	-4.88%	-12.20%	—

REFERENCES

[1]S. Sundaram, P. Kellnhofer, Y. Li, J.-Y. Zhu, A. Torralba, and W. Matusik, "Learning the signatures of the human grasp using a scalable tactile glove," Nature **569**, 698–702 (2019).

[2]Y. Luo, Y. Li, P. Sharma, W. Shou, K. Wu, M. Foshey, B. Li, T. Palacios, A. Torralba, and W. Matusik, "Learning human–environment interactions using conformal tactile textiles," Nature Electronics **4**, 193–201 (2021).

[3]A. Tashakori, Z. Jiang, A. Servati, S. Soltanian, H. Narayana, K. Le, C. Nakayama, C.-l. Yang, Z. J. Wang, J. J. Eng, *et al.*, "Capturing complex hand movements and object interactions using machine learning-powered stretchable smart textile gloves," Nature Machine Intelligence **6**, 106–118 (2024).

[4]A. Moin, A. Zhou, A. Rahimi, A. Menon, S. Benatti, G. Alexandrov, S. Tamakloe, J. Ting, N. Yamamoto, Y. Khan, *et al.*, "A wearable biosensing system with in-sensor adaptive machine learning for hand gesture recognition," Nature Electronics **4**, 54–63 (2021).

[5]X. Tian, P. M. Lee, Y. J. Tan, T. L. Wu, H. Yao, M. Zhang, Z. Li, K. A. Ng, B. C. Tee, and J. S. Ho, "Wireless body sensor networks based on metamaterial textiles," Nature Electronics **2**, 243–251 (2019).

[6]X. Tian, Q. Zeng, D. Nikolayev, and J. S. Ho, "Conformal propagation and near-omnidirectional radiation with surface plasmonic clothing," IEEE Transactions on Antennas and Propagation **68**, 7305–7315 (2020).
