# Supplementary material for: Metamaterial-enabled battery-free wireless sensor networks for unencumbered human-machine interactions
Source: Nat Commun. 2026 Sep 14;17:10778. doi: 10.1038/s41467-026-77600-2 (PMC13661596; doi:10.1038/s41467-026-77600-2)
Supplement: Supplementary file 3 [file 41467_2026_77600_MOESM2_ESM.pdf]

Description of Additional Supplementary Files:

Supplementary Movie 1: Wireless powering and communication enabled by metamaterial textile.

Supplementary Movie 2: Wireless signal enhancement by metamaterial textile.

Supplementary Movie 3: Battery-free wireless sensory glove enabled by metamaterial textiles.

Supplementary Movie 4: Human-robot collaboration by battery-free WBSN.

Supplementary Movie 5: Unencumbered and intuitive VR interaction by battery-free WBSN.

Supplementary Movie 6: Untethered full body motion digital twin by battery-free WBSN.

Supplementary Movie 7: Washing protocol.
